# Cholecystokinin coordinates gonadotropin-dependent and independent pathways to orchestrate zebrafish gonadal development

**DOI:** 10.1038/s41467-026-72039-x

**Published:** 2026-04-18

**Authors:** Hangyu Li, Faming Yuan, Hongwei Liang, Xiang Li, Shuo Zheng, Linlin Wang, Xiangchen Wu, Changyan Li, Xiangtong Zeng, Xuewen Rao, Dandong Huang, Zhixiu Liang, Tianyi Cai, Shaohua Xu, Xiangfeng Qin, Xiangjiang Liu, Zhan Yin, Guangfu Hu

**Affiliations:** 1 College of Fisheries, Huazhong Agricultural University, Wuhan, China; 2 Shenzhen Research Institute, Ocean University of China, Shenzhen, China; 3 Key Lab of Freshwater Biodiversity Conservation Ministry of Agriculture, Yangtze River Fisheries Research Institute, The Chinese Academy of Fisheries Sciences, Wuhan, China; 4 Institute of Hydrobiology, Chinese Academy of Sciences, Wuhan, China

**Keywords:** Reproductive biology, Ichthyology, Neurophysiology

## Abstract

Cholecystokinin (CCK) is established as a critical regulator of teleost gonadal development, functioning not only as a follicle-stimulating-hormone-releasing hormone (FSH-RH) but also stimulating gonadal development through three distinct mechanisms. Specifically, (1) CCK directly activates pituitary *lhb* transcription via CCKBRb in a specific bi-hormonal subpopulation, and indirectly enhances hypothalamic *gnrh3* expression to regulate LH synthesis and secretion. However, CCK’s physiological role in LH regulation requires further investigation, and its effect may involve both direct and indirect mechanisms, including targeting a specific bi-hormonal subpopulation. (2) CCK exerts gonadotropin-independent control over primordial germ cell (PGC) proliferation during embryogenesis, evidenced by significantly reduced PGC numbers in *cck1*^*−/−*^*;cck2*^*−/−*^ and *cckbrb*^*−/−*^ mutants, a phenotype absent in *fshb*^*−/−*^ and *lhb*^*−/−*^*;fshb*^*−/−*^ zebrafish. (3) CCK initiates meiosis and maintains gonadal somatic cell survival by suppressing apoptosis; these processes are abrogated in CCK-deficient mutants but remain intact in gonadotropin-deficient lines. Collectively, hypothalamic and peripheral CCK cells regulate gonadal function through gonadotropin-dependent and gonadotropin-independent pathways, respectively, thereby coordinating gonadal development from germline establishment to maturation.

## Abstract (web summary)

In zebrafish, cholecystokinin drives gonadal development via two parallel pathways: direct FSH stimulation in the pituitary and local regulation of germ cell proliferation and survival within the gonad.

## Introduction

Historically, the classical neuropeptide cholecystokinin (CCK) was recognized as an enteric hormone predominantly involved in digestive regulation^1^. Subsequent research revealed its widespread presence in the central nervous system and its roles in various physiological processes, including appetite, energy metabolism, mood, pain, and reproduction^2–7^. Notably, recent studies have identified CCK as a follicle-stimulating hormone-releasing hormone (FSH-RH) in teleosts^8–10^. In these species, the pituitary gland secretes two key gonadotropins: luteinizing hormone (LH) and follicle-stimulating hormone (FSH). FSH is crucial for early gonadal development, particularly germ cell proliferation and differentiation, while LH is essential for oocyte maturation and ovulation^11–14^. Thus, understanding the regulation of these gonadotropins is vital for elucidating the endocrine control of teleost reproduction. Although luteinizing hormone-releasing hormone (LH-RH), discovered in 1971, is well-established as the main regulator of gonadotropin release in mammals and most vertebrates^15^, this model has been challenged in teleosts. In teleosts, hypothalamic neurons directly innervate the pituitary pars distalis. This anatomical arrangement facilitates the direct delivery of diverse neuroendocrine factors to gonadotropes, which express a repertoire of receptors to integrate these signals. Consistent with this multi-factorial control system, in GnRH-deficient models like medaka and zebrafish, FSH synthesis and function remain largely unaffected, indicating the presence of alternative FSH-releasing factors^16,17^. A comprehensive neuropeptide screen identified CCK as a strong inducer of *fshb* transcription and FSH secretion in vitro^18,19^, and its physiological relevance was confirmed in both medaka^8^ and zebrafish^9^, establishing CCK as an FSH-RH in teleosts. However, while ligand-level analyses have been performed in medaka^8^, studies in zebrafish have primarily focused on receptor activation and knockout models^9^. The identity of the physiologically relevant endogenous ligand(s) within the CCK/gastrin family remains unclear, and whether CCK itself or other peptides such as gastrin serve as the physiologically relevant endogenous ligand(s) in zebrafish has not been fully determined. Therefore, a broader consensus regarding CCK regulation of gonadotropins across teleost species has yet to be established.

Beyond its role in gonadotropin regulation, emerging evidence from genetic ablation models suggests additional roles for CCK in gonadal development. Zebrafish mutants lacking CCK receptor *cckbrb*^*−/−*^ develop exclusively as infertile males^9^. This severe phenotype starkly contrasts with that of *fshb*^*−/−*^ and *lhb*^*−/−*^*;fshb*^*−/−*^ mutants, which retain partial fertility and lack comparable testicular development defects^14^. These observations imply that CCK may govern early germline development through previously unrecognized, gonadotropin-independent pathways.

Gonadal development in teleosts involves four major stages: primordial gonad formation, differentiation, growth, and final maturation^20^. Early developmental events, particularly primordial germ cell (PGC) migration and proliferation during 0-10 days post fertilization (dpf)^21^, as well as meiosis initiation around 13–14 dpf, represent critical checkpoints^22^. The entry of PGCs into meiosis is considered to be a gonadotropin-independent stage^23^. Disruption of these processes often leads to masculinization and subsequent testicular degeneration^24–27^. While FSH and LH primarily function during later development stages, the potential role of CCK in regulating earlier events such as PGC dynamics and meiosis initiation has remained unexplored.

In this study, we examined the critical roles of CCK across all four stages of zebrafish gonadal development, revealing its four pivotal regulatory functions. First, CCK promotes PGC proliferation through gonadotropin-independent pathways, thereby influencing sex determination. Second, it facilitates meiotic initiation and sustains gonadal somatic cells viability during differentiation, also through gonadotropin-independent mechanisms. Third, CCK directly stimulates pituitary FSH synthesis and secretion, driving folliculogenesis and spermatogenesis. Finally, CCK is involved in the regulation of oocyte maturation and ovulation. Collectively, these findings highlight CCK as a central regulator of zebrafish gonadal development, from germ cell initiation to final maturation.

## Results

### Loss of CCK or its receptor CCKBRB leads to male-biased sex differentiation and infertility

**Fig. 1:**
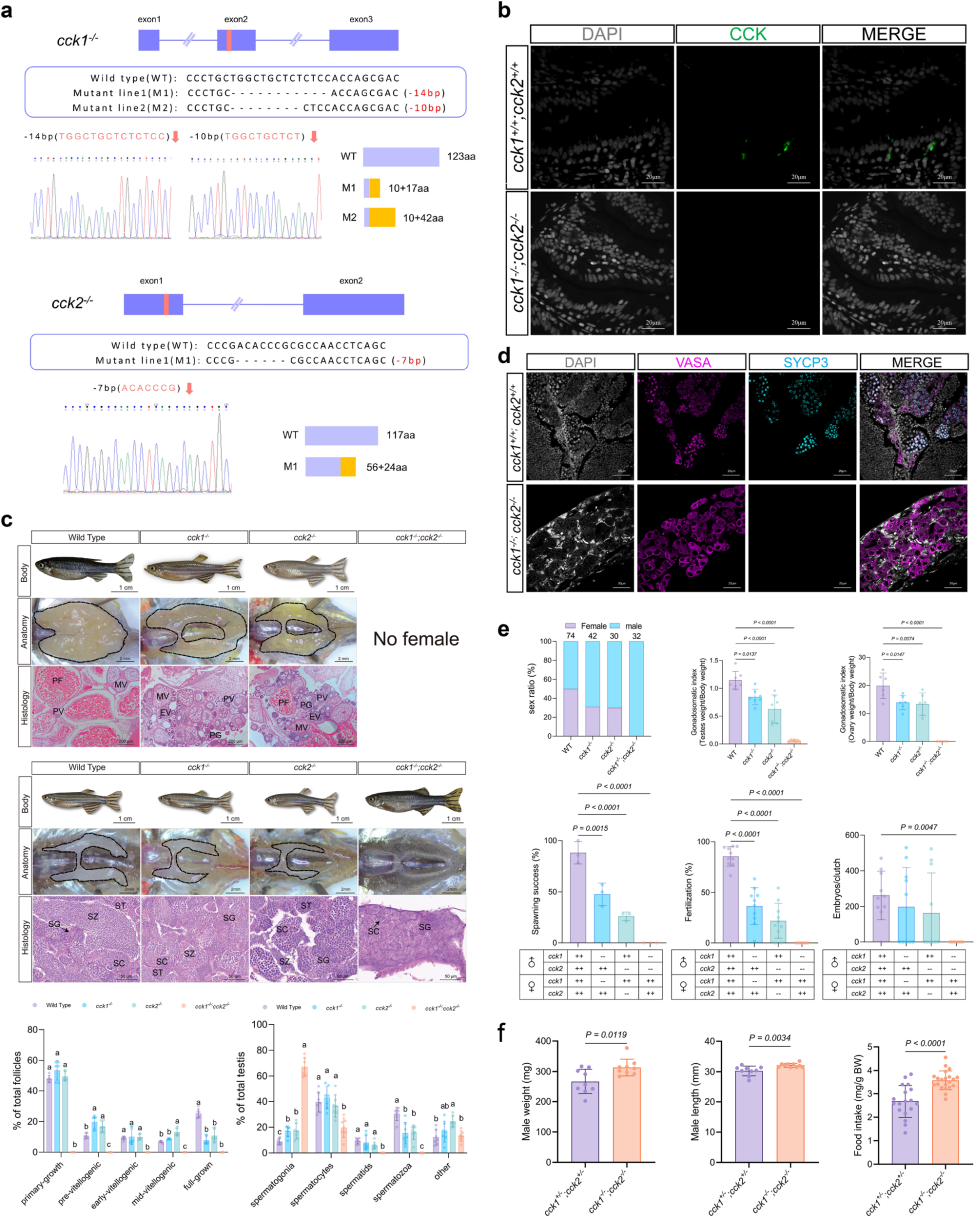
Generation and reproductive phenotype validation of *cck1* and *cck2* knockout zebrafish models. **a** Schematic diagram of the CRISPR/Cas9 targeting strategy and the resulting deletion mutations in *cck1* and *cck2*. **b** Immunostaining of CCK protein in the intestine of *cck1*^+/+^;*cck2*^+/+^ and *cck1*^−/−^;*cck2*^−/−^ zebrafish. **c** Gonadal morphology and histology of 3-month-old zebrafish. Ovarian stages: PG, primary growth; PV, pre-vitellogenic; EV, early vitellogenic; MV, mid-vitellogenic; FG, full-grown. Testicular cells: SG, spermatogonia; SC, spermatocytes; ST, spermatids; SZ, spermatozoa. Bar charts show histological quantification (ovaries, *n* = 6; testes, *n* = 7). Different letters indicate statistically significant differences among groups as determined by one-way ANOVA followed by Tukey’s multiple-comparisons test; groups sharing at least one letter are not significantly different. **d** VASA and SYCP3 immunofluorescence staining in testes of 3-month-old male zebrafish. **e** Reproductive phenotypes including sex ratio, gonadosomatic index (GSI) (female, *n* = 6; male, *n* = 7), spawning success (*n* = 3 groups of 12 pairs), fertilization rates (*n* = 10), and clutch size (*n* = 10). Statistical significance was determined by one-way ANOVA followed by Dunnett’s multiple-comparisons test. **f** Comparisons of body weight (*n* = 9), body length (*n* = 9), and food intake (*n* = 18). Statistical significance was determined by two-sided unpaired Student’s *t* test. Data are presented as mean ± s.e.m. For (**b**, **d**), representative images from three independent experiments with similar results are shown.

In zebrafish, two cholecystokinin genes, *cck1* and *cck2*, have been identified. Quantitative expression analysis indicated that both ligands are predominantly expressed in the brain and intestine (Supplementary Fig. 1). To investigate CCK’s role in gonadal development, we generated *cck1*^*−/−*^*;cck2*^*−/−*^ double knockout (DKO) zebrafish using CRISPR/Cas9 technology (Fig. 1a, b). Strikingly, all DKO individuals developed into males with underdeveloped gonads and were completely infertile (Fig. 1e). This infertility was evidenced by reproductive failure in mating trials and impaired mating behavior (Fig. 1e). Histological examination at three months revealed gamete arrest at the pre-meiotic stage, indicating impaired spermatogenesis (Fig. 1c, d). Furthermore, compared to wild-type controls, *cck1*^*−/−*^*;cck2*^*−/−*^ mutants exhibited significantly increased feeding rates, body length, and weight (Fig. 1f). These results demonstrate that CCK is essential for regulating feeding behavior, somatic growth, and gonadal development in zebrafish.

**Fig. 2:**
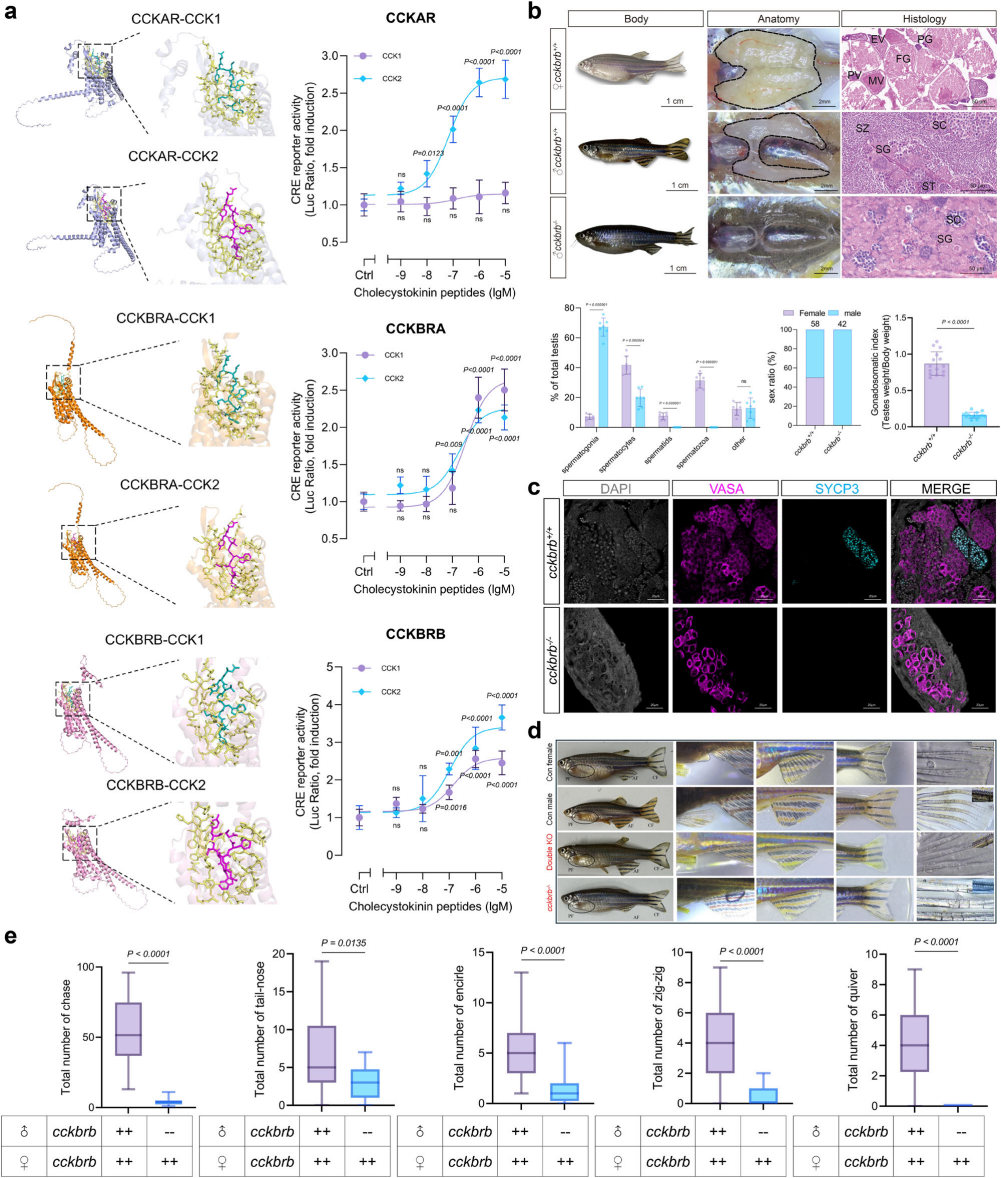
Generation and phenotypic characterization of the *cckbrb* knockout zebrafish model. **a** Ligand-receptor binding analysis. Structural prediction of zebrafish CCK interacting with its receptors (CCKAR, CCKBRA, and CCKBRB) via AlphaFold3 and functional validation using a dual-luciferase reporter assay (*n* = 4). Statistical significance was determined by one-way ANOVA followed by Dunnett’s multiple-comparisons test. ns indicates no significant difference where applicable. Data are presented as mean ± s.e.m. **b** Gonadal morphology and histology in 5-month-old *cckbrb*^+/+^ and *cckbrb*^−/−^ zebrafish. Quantification includes the distribution of gonadal cell stages (*n* = 7), sex ratio, and gonadosomatic index (GSI) (*n* = 15). Statistical significance was determined by two-sided unpaired Student’s *t* test. ns indicates no significant difference where applicable. Data are presented as mean ± s.e.m. **c** VASA and SYCP3 immunofluorescence in testes of *cckbrb*^*+/+*^ and *cckbrb*^−/−^ zebrafish. Representative images from three independent experiments with similar results are shown. **d** Analysis of secondary sexual characteristics (SSC) in 4-month-old zebrafish. Representative images comparing the anal fin (AF), caudal fin (CF), and pectoral fin (PF) phenotypes in wild-type, *cck1*^*−/−*^;*cck2*^*−/−*^, and *cckbrb*^*−/−*^ zebrafish. **e** Reproductive behavior analysis assessing courtship frequency in *cckbrb*^*−/−*^ zebrafish (*n* = 24 independent pairings). Statistical significance was determined by two-sided unpaired Student’s *t* test. In the box plots, the center line indicates the median, the box bounds indicate the first and third quartiles, and the whiskers indicate the minimum and maximum values.

Among the three zebrafish CCK receptors, ligand-receptor binding assays identified CCKBRB as having the highest affinity for both CCK1 and CCK2 peptides (Fig. 2a and Supplementary Table 1). Consistent with a reproductive role, *cckbrb* was predominantly expressed in the pituitary gland (Supplementary Fig. 1). Subsequently, we generated a *cckbrb*^*−/−*^ zebrafish model (Supplementary Fig. 2). These mutants phenocopied the *cck1*^*−/−*^*;cck2*^*−/−*^ double knockouts, developing exclusively into males with arrested testicular development at the pre-meiotic stage (Fig. 2b, c). Although these mutants displayed normal male secondary sexual characteristics (Fig. 2d), they exhibited a profound deficit in courtship behaviors (Fig. 2e, Supplementary Fig. 3 and Supplementary Movie 1, 2), leading to reproductive failure. This behavioral defect was not due to motor impairment, as feeding-driven locomotor activity remained normal in both *cck1*^*−/−*^*;cck2*^*−/−*^ and *cckbrb*^*−/−*^ mutants (Supplementary Fig. 4). To investigate the underlying mechanism, metabolomic profiling was performed, revealing significantly reduced testicular testosterone and androstenedione levels in *cckbrb*^*−/−*^ males (Supplementary Fig. 5). Crucially, exogenous testosterone treatment successfully rescued the courtship defects (Supplementary Fig. 6), establishing that CCK signaling governs reproductive behavior by maintaining androgen levels.

### CCK promotes pituitary gonadotropin synthesis and secretion via distinct mechanisms

**Fig. 3:**
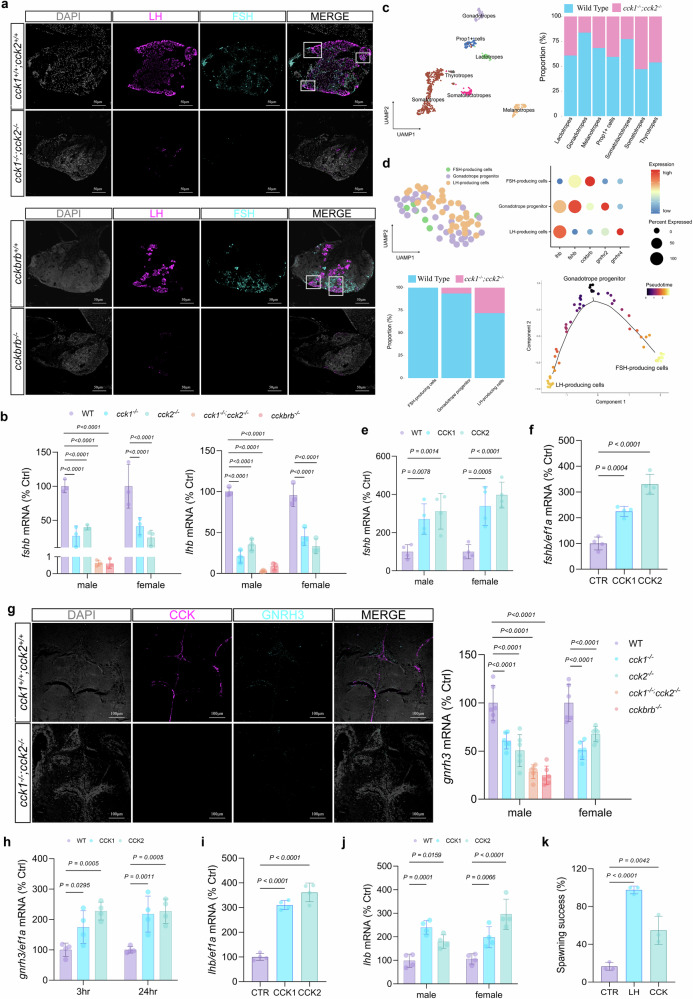
CCK regulates the synthesis and function of pituitary FSH and LH in zebrafish. **a** Representative immunohistochemical staining of LH and FSH proteins in the pituitary of WT, *cck1*^*−/−*^*;cck2*^*−/−*^, and *cckbrb*^*−/−*^ zebrafish. Representative images from three independent experiments with similar results. **b** Relative mRNA expression of *lhb* and *fshb* in pituitary tissue from different knockout models compared to WT. Data are derived from *n* = 3 independent biological replicates, where each replicate consists of a pool of 3 pituitaries. **c** Single-cell RNA sequencing (scRNA-seq) visualization of zebrafish pituitary cell clusters. **d** Sub-clustering and trajectory analysis of the gonadotrope cell population. **e**, **f** Regulation of *fshb* expression. **e** Pituitary *fshb* mRNA levels measured 24 hours after intraperitoneal (i.p.) injection of CCK1 or CCK2. Data represent *n* = 4 independent biological replicates, each consisting of a pool of 4 pituitaries. **f** *fshb* mRNA levels in primary pituitary cells treated with CCK1 or CCK2 for 24 hours (*n* = 4). **g**, **h**, Analysis of the upstream hypothalamic gnrh3 response. **g** Hypothalamic gnrh3 expression in CCK ligand and receptor knockout models (*n* = 6). **h** gnrh3 mRNA levels in hypothalamic cells treated with CCK1 or CCK2 for 3 h and 24 h (*n* = 4). **i**, **j** Regulation of *lhb* expression. **i** *lhb* mRNA levels in primary pituitary cells treated with CCK1 or CCK2 for 24 h. Data represent *n* = 4 independent biological replicates, each consisting of a pool of 4 pituitaries. **j** Pituitary *lhb* mRNA levels measured 24 hours after i.p. injection of CCK1 or CCK2. Data represent *n* = 4 independent biological replicates, each consisting of a pool of 3 pituitaries. **k** Functional assessment of ovulation success rate (%) in female zebrafish 24 hours after i.p. injection of LH or CCK (*n* = 14 biologically independent animals from 3 independent experiments). Statistical significance was determined by one-way ANOVA followed by Dunnett’s multiple-comparisons test for (**b**, **e**–**k**). Data are presented as mean ± s.e.m.

Previous studies identified CCK as a principal FSH-releasing factor in fish pituitary glands^8,9^. Here, we elucidate its mechanisms in regulating both FSH and LH synthesis. Immunofluorescence analysis revealed that FSH protein signals were almost undetectable in the pituitaries of *cck1*^*−/−*^*;cck2*^*−/−*^ and *cckbrb*^*−/−*^ zebrafish compared to wild-type controls (Fig. 3a). Consistent with this, qPCR confirmed a significant reduction in *fshb* transcript levels in these mutants (Fig. 3b). To dissect the cellular basis of this regulation, we performed single-cell RNA sequencing (scRNA-seq) on zebrafish pituitaries (Fig. 3c; Supplementary Fig. 7). Trajectory analysis of the gonadotrope lineage revealed a common progenitor population expressing stemness markers, which subsequently diverges into distinct FSH and LH differentiation paths (Supplementary Fig. 8). This developmental trajectory supports the existence of a common precursor, consistent with the *lhb/fshb* co-expressing cells identified in our model (Fig. 3d). Notably, *cckbrb* expression was specifically enriched in FSH-producing cells and their precursors (Fig. 3d). Consequently, *cck1*^*−/−*^*;cck2*^*−/−*^ mutant pituitaries exhibited a marked reduction in the number of FSH-producing cells (Fig. 3d), suggesting that CCK directly activates CCKBRB to stimulate FSH synthesis and cell maintenance.

To validate the direct regulation of FSH, we performed gain-of-function experiments. Intraperitoneal injection of CCK peptides significantly induced pituitary *fshb* mRNA expression in vivo (Fig. 3e), and CCK treatment robustly induced *fshb* expression in primary cultured pituitary cells in vitro (Fig. 3f), confirming a direct pituitary action. The efficacy of systemic CCK delivery was corroborated by elevated plasma CCK levels and pituitary response following feeding (Supplementary Fig. 9). Functionally, long-term dietary CCK supplementation promoted ovarian development in wild-type fish but failed to do so in *fshb*^*−/−*^ mutants, confirming that CCK promotes gonadal development specifically through FSH (Supplementary Fig. 10).

In contrast to the direct regulation of FSH, LH synthesis is modulated by CCK through a dual mechanism involving both direct pituitary actions and indirect hypothalamic pathways. Loss of CCK signaling led to significantly reduced LH protein and *lhb* transcript levels in mutant pituitaries (Fig. 3a, b). We first investigated the indirect pathway via the hypothalamus. Expression of *gnrh3*, a key upstream regulator of LH, was significantly reduced in the hypothalamus of both ligand and receptor mutants. Immunofluorescence revealed close anatomical proximity between GnRH3 neurons and CCK-expressing fibers in the hypothalamus (Fig. 3g; Supplementary Fig. 11). Furthermore, scRNA-seq of the hypothalamus detected *cckbrb* expression within GnRH3 neurons (Supplementary Fig. 12). Consistently, CCK treatment significantly upregulated *gnrh3* expression in primary hypothalamic cell cultures (Fig. 3h). We confirmed the functionality of this axis, as GnRH3 treatment significantly upregulated *lhb* expression in primary pituitary cells (Supplementary Fig. 13), supporting an indirect pathway where CCK stimulates GnRH3 to drive LH synthesis.

However, an indirect pathway alone could not fully explain the observed regulation. Direct administration of CCK also increased *lhb* transcript levels in vivo (Fig. 3j) and, critically, in primary pituitary cells in vitro (Fig. 3i), demonstrating a direct action independent of hypothalamic input. To definitively dissect the contribution of each pathway, we employed a hypothalamic-pituitary co-culture system. Blocking GnRH3 signaling with a neutralizing antibody only partially attenuated the CCK-induced *lhb* upregulation (Supplementary Fig. 14), confirming the existence of a parallel direct pituitary mechanism. The cellular basis for this direct regulation was identified through scRNA-seq, which revealed a population of gonadotropes co-expressing both *fshb* and *lhb* (Fig. 3d). Within this dual-hormone cell population, *cckbrb* expression was specifically detected (Fig. 3d). High-resolution co-localization analysis further confirmed that *cckbrb* mRNA is present in LH-protein-positive cells (Supplementary Fig. 15). These data establish that CCKBRB is positioned to directly regulate *lhb* transcription in a specific subset of gonadotropes. Functionally, this dual regulation is physiologically relevant, as injection of either CCK or LH significantly restored ovulation rates in female zebrafish (Fig. 3k).

Finally, we explored the evolutionary conservation of this mechanism. In contrast to zebrafish, intraperitoneal injection of CCK-8 into adult male mice failed to alter pituitary *lhb* or *fshb* levels (Supplementary Fig. 16a, b). Moreover, scRNA-seq of the mouse pituitary showed that while LH and FSH are co-expressed, these gonadotropes do not express CCK receptors (*cckar* or *cckbr*) (Supplementary Fig. 16c). This highlights a significant species-specific divergence in the neuroendocrine control of reproduction.

### CCK promotes primordial germ cell proliferation and migration independent of gonadotropins

To determine if CCK regulates gonadal development exclusively via gonadotropins, we generated *lhb*^*−/−*^, *fshb*^−/−^ and *lhb*^*−/−*^*;fshb*^*−/−*^ zebrafish mutants. In *lhb*^*−/−*^ mutants, gonadal development and sex ratios were comparable to wild-type controls, although homozygous adults were infertile (Supplementary Figs. 17, 19a, b). Similarly, *fshb*^−/−^ males exhibited normal testicular morphology and fertility, while females showed impaired ovarian development (Supplementary Figs. 17, 19). In both single mutants, testicular SYCP3 protein levels remained normal (Supplementary Fig. 18). Even in *lhb*^*−/−*^*;fshb*^*−/−*^ double mutants, which displayed severe reproductive impairment and a male-biased sex ratio, female individuals were still present, and mutant testes contained mature spermatozoa and detectable SYCP3 signals (Supplementary Figs. 17, 18, 19). Critically, none of the gonadotropin-deficient models recapitulated the complete male bias or the severe pre-meiotic arrest characteristic of *cck1*^−/−^;*cck2*^−/−^ and *cckbrb*^**−/−**^ mutants. These phenotypic discrepancies suggest that CCK regulates gonadal development through additional, gonadotropin-independent pathways.

**Fig. 4:**
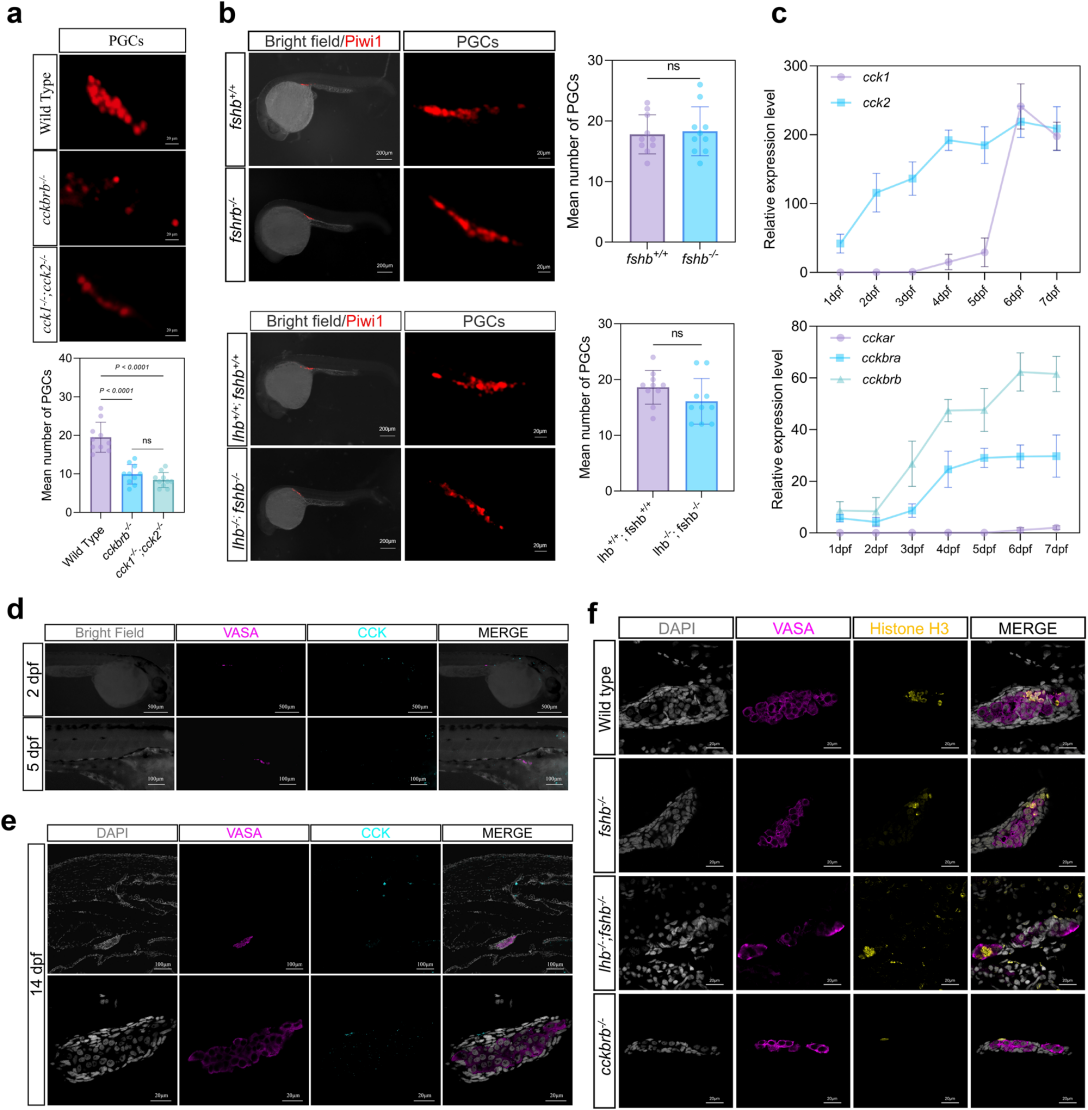
Impact of CCK signaling on primordial germ cell (PGC) proliferation and migration in zebrafish. **a**, **b** Analysis of PGC number and distribution in zebrafish embryos at 24 hours post-fertilization (hpf). PGCs were visualized using Piwi1 immunofluorescence staining. **a** Representative images and quantification of PGCs in wild-type (WT), *cckbrb*^*−/−*^, and *cck1*^*−/−*^*;cck2*^*−/−*^ embryos. Statistical significance was determined by one-way ANOVA followed by Tukey’s multiple-comparisons test. ns indicates no significant difference where applicable. Data are presented as mean ± s.e.m. **b** Representative images and quantification of PGCs in *fshb*^*+/+*^, *fshb*^*−/−*^, *lhb*^*+/+*^*;fshb*^*+/+*^ and *lhb*^*−/−*^*;fshb*^*−/−*^ embryos. Statistical significance was determined by two-sided unpaired Student’s *t* test. ns indicates no significant difference where applicable. Data are presented as mean ± s.e.m. (*n* = 10 embryos per group). **c** Temporal expression profiles of CCK ligands and receptors during early development. Relative mRNA levels of ligands (*cck1, cck2*) and receptors (*cckar, cckbra, cckbrb*) were quantified by qPCR from 1 to 7 days post-fertilization (dpf). Data are presented as mean ± s.e.m. for *n* = 3 independent replicates. Each replicate represents a pooled sample of 20–30 embryos or larvae. **d** Whole-mount immunofluorescence co-staining of VASA (germ cell marker) and CCK in zebrafish larvae at 2 dpf and 5 dpf. Merged images show the spatial relationship between CCK-expressing cells and migrating PGCs. **e** Immunofluorescence of CCK and VASA in 14 dpf gonads reveals CCK expression in somatic cells surrounding germ cells. **f** Immunofluorescence co-expression analysis of germ cells (VASA) and mitotic cells (Histone H3) in gonadal tissue of WT, *fshb*^*−/−*^*, lhb*^*−/−*^*;fshb*^*−/−*^, and *cckbrb*^*−/−*^ zebrafish at 14 dpf. For (**d**, **f**), representative images from three independent experiments with similar results are shown.

To identify these mechanisms, we investigated CCK expression dynamics and its role in early primordial germ cell (PGC) development. Quantitative PCR profiling revealed that the expression levels of CCK ligands and receptors exhibited a progressive increase throughout early embryonic development (Fig. 4c). Immunofluorescence co-staining performed at 2 and 5 dpf revealed that CCK protein is expressed along the migration route of VASA-positive PGCs (Fig. 4d). By 14 dpf, CCK expression was specifically localized to gonadal somatic cells surrounding the germ cells (Fig. 4e), suggesting a direct paracrine interaction. We next assessed PGC migration and number. At 24 hpf, *cck1*^−/−^;*cck2*^−/−^ and *cckbrb*^−/−^ embryos exhibited significantly fewer PGCs with aberrant dispersion compared to wild-type controls, indicating impaired migration (Fig. 4a). In contrast, PGC number and distribution were unaffected in *fshb*^−/−^ and *lhb*^*−/−*^*;fshb*^*−/−*^ mutants (Fig. 4b).

We further evaluated germ cell proliferation using VASA (germ cells) and Histone H3 (mitotic marker) co-staining. At 14 dpf, wild-type and *fshb*^*−/−*^ gonads showed robust mitotic activity (Fig. 4f). While *lhb*^*−/−*^*;fshb*^*−/−*^ mutants exhibited a modest reduction in mitosis, *cckbrb*^*−/−*^ mutants displayed a severe proliferation defect, with mitotic signals predominantly confined to a few somatic cells (Fig. 4f). This defect persisted at later stages; by 45 dpf, while mitotic activity increased in wild-type gonads, it remained minimal in *cckbrb*^*−/−*^ mutants (Supplementary Fig. 20, 21). Collectively, these findings establish that CCK signaling is critical for PGC migration and proliferation during primordial gonad formation, acting through a local, gonadotropin-independent mechanism.

### CCK signaling regulates germ cell meiosis and inhibits gonadal somatic cell apoptosis

**Fig. 5:**
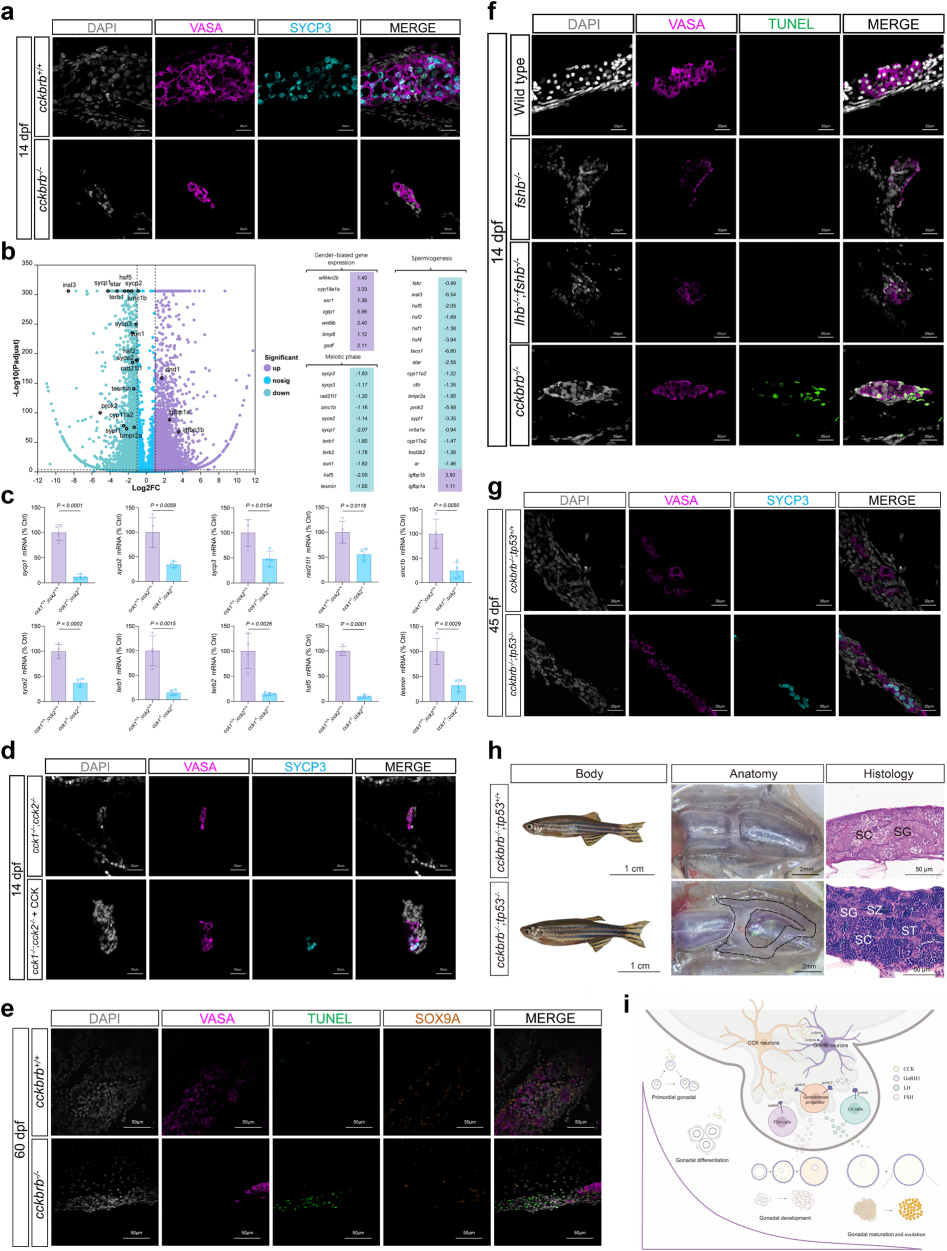
CCK signaling regulates germ cell meiosis and gonadal somatic cell survival. **a** Immunofluorescence showing the expression of VASA and SYCP3 in gonadal tissue of 14 dpf WT and *cckbrb*^*−/−*^ zebrafish. **b** RNA-seq analysis of testis tissue from WT and *cck1*^*−/−*^*;cck2*^*−/−*^ zebrafish. Volcano plot (left) and gene lists (right) display differentially expressed genes related to meiosis and spermiogenesis. Differential expression analysis was performed as described in the Methods. **c** qPCR analysis of meiosis-related gene expression in testes of WT and *cck1*^*−/−*^*;cck2*^*−/−*^ zebrafish. Data are presented as mean ± s.d. (*n* = 4). Statistical significance was determined by two-sided unpaired Student’s *t* test. **d** VASA and SYCP3 co-staining in gonadal sections of 14 dpf *cck1*^*−/−*^*;cck2*^*−/−*^ larvae following exogenous CCK immersion treatment starting from fertilization. **e** Co-staining of VASA, TUNEL, and SOX9a in testes of 60 dpf zebrafish. **f** TUNEL and VASA staining in 14 dpf gonads of WT, *fshb*^*−/−*^, *lhb*^*−/−*^*;fshb*^*−/−*^, and *cckbrb*^*−/−*^ zebrafish. **g**, **h** Analysis of *cckbrb*^*−/−*^*;tp53*^*−/−*^ zebrafish. **g** Immunofluorescence of SYCP3 and VASA in testes at 45 dpf. **h** Gross morphology and histological analysis (H&E) of gonads. **i** Schematic diagram depicting the stage-specific regulatory network of CCK signaling during gonadal development. The model illustrates CCK involvement in primordial germ cell (PGC) proliferation during early formation, the maintenance of somatic cell integrity (via apoptosis inhibition) and meiotic initiation during differentiation, and the regulation of pituitary FSH and LH synthesis (involving both direct pituitary action and indirect hypothalamic *gnrh3* modulation) to support gametogenesis and ovulation. Created in BioRender. Li, H. (2026) https://biorender.com/o68vxgm. For (**a**, **d**–**h**), representative images from three independent experiments with similar results are shown.

To determine whether cckbrb deficiency affects germ cell meiosis during gonadal differentiation, we performed immunofluorescence co-staining for VASA (germ cell marker) and SYCP3 (meiotic marker) on gonadal tissues of wild-type and *cckbrb*^*−/−*^ zebrafish at 14 dpf. Robust SYCP3 signals in wild-type gonads indicated active meiotic initiation, whereas *cckbrb*^*−/−*^ mutants exhibited markedly reduced SYCP3 expression, suggesting a failure to initiate meiosis (Fig. 5a). To assess whether this regulation is independent of gonadotropins, we analyzed SYCP3 expression in *fshb*^*−/−*^, *lhb*^*−/−*^*;fshb*^*−/−*^ mutants at 20 and 45 dpf. SYCP3-positive cells were readily detected in both wild-type and gonadotropin-deficient testes, whereas *cckbrb*^*−/−*^ mutants showed a near-complete loss of SYCP3 signals (Supplementary Figs. 22, 23). This indicates that CCK regulates meiotic initiation through a mechanism distinct from the classical hypothalamus-pituitary-gonad axis. Longitudinal analysis of *cck1*^*−/−*^*;cck2*^*−/−*^ mutants at 60, 120, and 150 dpf revealed a persistent absence of SYCP3 expression compared to sustained signals in wild-type gonads (Supplementary Fig. 24), confirming a sustained arrest of germ cell differentiation at the pre-meiotic stage.

To elucidate the molecular basis of this defect, we performed RNA-seq analysis on testes from wild-type and *cck1*^*−/−*^*;cck2*^*−/−*^ zebrafish. The transcriptomic profile revealed significant downregulation of meiosis- and spermatogenesis-related genes in mutants (Fig. 5b), a finding validated by quantitative PCR (Fig. 5c). To test whether CCK could functionally rescue this defect, we treated fertilized eggs from *cck1*^*−/−*^*;cck2*^*−/−*^ mutants with exogenous CCK solution for 14 days. We first confirmed the efficacy of the immersion protocol by detecting elevated internal CCK concentrations in treated larvae (Supplementary Fig. 25). Notably, SYCP3 expression was partially restored in germ cells from treated embryos, supporting the conclusion that CCK signaling actively promotes meiotic initiation (Fig. 5d).

Given that gonadal somatic cell integrity is essential for germ cell development, we examined whether CCK signaling supports somatic cell survival. VASA/TUNEL co-staining at 14 dpf revealed minimal apoptosis in wild-type and gonadotropin-deficient gonads (*fshb*^*−/−*^ and *lhb*^*−/−*^*;fshb*^*−/−*^) (Fig. 5f). In stark contrast, *cckbrb*^*−/−*^ mutants exhibited extensive apoptosis in somatic cells surrounding the germ cells (Fig. 5f). Further characterization at 60 dpf using the Sertoli cell marker SOX9a revealed that apoptosis was not co-localized with SOX9a, indicating that cell death occurs primarily in Leydig cells (Fig. 5e). To determine if this somatic apoptosis was the primary cause of the meiotic failure, we generated *cckbrb*^*−/−*^*;tp53*^*−/−*^ double mutants to block apoptosis. Loss of tp53 successfully rescued the meiotic defect, restoring SYCP3 expression in *cckbrb*^*−/−*^ testes (Fig. 5g; Supplementary Fig. 26). Furthermore, anatomical and histological analysis showed that *cckbrb*^*−/−*^*;tp53*^*−/−*^ mutants developed observable gonads with active sperm production, rescuing the severe gonadal atrophy seen in single mutants (Fig. 5h). Collectively, these results indicate that CCK suppresses gonadal somatic cell apoptosis independently of FSH and LH, thereby preserving the gonadal microenvironment required for germ cell meiosis.

## Discussion

Gonadal maturation in teleosts proceeds through four distinct stages: primordial gonad formation, gonadal differentiation, development, and final maturation^20^. Our findings demonstrate that CCK signaling exerts critical, stage-specific regulatory effects across this entire ontogeny, acting through hierarchically distinct gonadotropin-independent and dependent mechanisms. During the primordial formation stage (0–10 dpf in zebrafish), PGCs migrate to the genital ridge and proliferate to establish the gonadal primordium^21,28–30^. Critically, disruption of PGC migration or proliferation at this stage typically leads to all-male phenotypes and arrested gonadal development^24,25,31,32^. In the present study, both *cck1*^*−/−*^*;cck2*^*−/−*^ and *cckbrb*^*−/−*^ zebrafish exhibited significantly fewer PGCs and mitotic arrest. Notably, these defects were absent in *fshb*^*−/−*^ and *fshb*^*−/−*^*;lhb*^*−/−*^ mutants. This phenotypic distinction reveals a gonadotropin-independent function for CCK in promoting PGC proliferation, identifying CCK as a key regulator at the very onset of gonadal establishment that likely sets the trajectory for sex determination.

As development progresses to the gonadal differentiation (13–14 dpf), germ cells initiate meiosis, a process essential for both oogenesis and spermatogenesis. Failure of meiotic entry universally induces complete masculinization and, if sustained, causes testicular degeneration^22,26,27,31,33–36^. In *cck1*^*−/−*^*;cck2*^*−/−*^ and *cckbrb*^*−/−*^ mutants, meiotic initiation was consistently blocked across developmental stages (14, 45, and 150 dpf). Concurrently, gonadal somatic cells (GSCs), which support meiosis through nutrients provision and regulatory signals, underwent extensive apoptosis at 14 and 45 dpf. Given that GSC depletion disrupts meiosis and gametogenesis^37–39^, this causality is further substantiated by our genetic rescue data: *cckbrb*^*−/−*^*;tp53*^*−/−*^ double mutants exhibited suppressed apoptosis and restored spermatogenesis (fertility). However, remarkably, female individuals were not observed in the rescued cohort. This dissociation supports a “two-phase” model: the initial reduction in PGC numbers (Phase I) likely locks the gonad into a male trajectory—a defect that *tp53* deletion cannot reverse—whereas the subsequent somatic apoptosis (Phase II) drives the meiotic arrest and sterility. Thus, while *tp53* deficiency rescues gonadal integrity and fertility, it is insufficient to overturn the sex determination bias established by early PGC loss. Crucially, neither somatic apoptosis nor meiotic arrest occurred in *fshb*^*−/−*^ or *fshb*^*−/−*^;*lhb*^*−/−*^ mutants, establishing that CCK maintains gonadal somatic cell integrity and promotes meiosis via gonadotropin-independent mechanisms during early gonadal development.

Following gonadal differentiation, zebrafish undergo an extended developmental phase characterized by follicle transition from Stage IB to Stage II, marking female puberty onset^40,41^, where FSH plays a central role. Our observation that loss of CCK signaling leads to a severe reduction in *fshb* synthesis, which is rescuable by exogenous CCK, aligns robustly with recent breakthroughs in the field. Specifically, seminal studies in medaka^8^ and zebrafish^9^ have identified CCK as a potent FSH-RH. Our data, derived from comprehensive genetic knockout models, provide independent confirmation of this conserved physiological axis, reinforcing the consensus that CCK directly modulates FSH output to drive gametogenesis in teleosts. Evolutionarily, teleosts exhibit a direct CCK-FSH regulatory axis, wherein FSH-producing gonadotropes express functional CCKRs^8,42,43^. This architecture allows the satiety hormone CCK to directly modulate FSH output, representing a key adaptation in broadcast spawners like zebrafish that require sustained energy investment into oogenesis. In contrast, mammals exhibit a different strategy: FSH and LH are co-produced by the same gonadotropes, which lack CCKRs, implying that FSH regulation in these groups is indirect and likely mediated through upstream hypothalamic signals^44–46^.

Upon completion of gametogenesis, the gonads enter the final maturation stage, during which LH plays a critical role in promoting oocyte maturation and ovulation. In *lhb*^*−/*^^−^ zebrafish, oocyte growth proceeds normally, but final maturation and ovulation are significantly impaired^11,12,47^. Building upon the established role of CCK as a major FSH-RH, our study reveals a significant expansion of its neuroendocrine repertoire: CCK also functions as a critical regulator of LH in zebrafish. Both *cck1*^*−/−*^*;cck2*^*−/−*^ and *cckbrb*^*−/−*^ mutants exhibited markedly reduced pituitary LH expression, implicating CCK in LH synthesis and secretion. The apparent discrepancy with earlier studies emphasizing a specific FSH-RH role may reflect differences in experimental focus and interpretation. Our data show that CCKBRb is enriched in a subpopulation of pituitary gonadotropes co-expressing *fshb* and *lhb* transcripts^43^, suggesting that CCK may directly influence *lhb* expression in a subset of responsive cells. This interpretation aligns with previous calcium imaging studies in zebrafish, which reported that CCK elicits robust calcium responses in FSH cells, whereas responses in LH cells were described as “weaker” and “varied widely” (20–100% of cells responding)^9^. Such variability supports our model that CCK does not universally activate all LH gonadotropes, but instead targets a specific subpopulation—likely the bi-hormonal (FSH/LH co-expressing) cells that also express *cckbrb*. In our primary pituitary culture system, the expansion of this bi-hormonal subpopulation under culture conditions may amplify the observed *lhb* response, offering a plausible explanation for the discrepancy between the robust in vitro induction and the more variable in vivo calcium signals. Meanwhile, the marked in vivo suppression of LH-related phenotypes may partially result from indirect effects due to impaired gonadal function and loss of gonadal feedback. Furthermore, while primary pituitary culture demonstrates that CCK has the intrinsic capacity to stimulate *lhb* expression, the magnitude of this response in vitro may be influenced by culture-induced cellular plasticity. Together, these findings support a cautious interpretation: CCK contributes to LH regulation, but the extent of its physiological role in vivo remains to be fully clarified.

In summary, our findings demonstrate that CCK signaling exerts stage-specific and multi-level regulatory effects on zebrafish gonadal development (Fig. 5i). During the embryonic stage of primordial gonad formation, CCK ensures sufficient germ cell pools via gonadotropin-independent PGC proliferation. At the gonadal differentiation stage, CCK maintains somatic cell integrity (preventing apoptosis) and enables timely meiotic initiation, averting masculinization. Throughout the developmental phase, CCK directly stimulates FSH synthesis through pituitary CCKBRb, driving gametogenesis. Thus, hypothalamic and peripheral CCK can act through gonadotropin-dependent and -independent pathways, respectively, in teleost reproduction.

## Methods

### Animals

Zebrafish (*Danio rerio*, AB strain) and the *tp53* mutant line (Catalog ID: CZ267) were obtained from the China Zebrafish Resource Center (CZRC) and maintained in recirculating aquaculture systems (28 ± 0.5 °C; 14:10-hour light: dark cycle). KM mice (male, 8-week-old, 35–45 g) were provided by the Laboratory Animal Center of Huazhong Agricultural University. All animal procedures were approved by the Institutional Animal Care and Use Committee of Huazhong Agricultural University (IACUC No. HZAUFI-2023-028) and complied with national ethical guidelines. For experiments performed at early developmental stages, sex could not be determined reliably and was therefore not considered in the experimental design or analysis. For adult zebrafish and mouse experiments, sex was determined and is indicated where applicable in the corresponding Methods sections and figure legends. Where sex information was collected, data were reported accordingly and disaggregated by sex where appropriate.

### Generation of zebrafish mutants

*cck1*^*−/−*^, *cck2*^*−/−*^, and *cckbrb*^*−/−*^ zebrafish mutant lines were generated via CRISPR/Cas9-mediated genome editing. The guide RNA (gRNA) target sequences were as follows: *cck1*—GTGGAGAGAGCAGCCAGCAGGG (exon 2); *cck2*—GCTGAGGTTGGCGCGGGTGTCGG (exon 1); and *cckbrb*—GGATGTGTTGCTGCGGGGTCGG (exon 5). gRNA and Cas9 protein were co-injected into zebrafish embryos at the one-cell stage by using Digital Pneumatic Microinjection Pump (DMP-300, Micrology (Wuhan) Precision Insuments, Ltd). Founders were outcrossed to WT, and F1 progeny were genotyped by Sanger sequencing. The *cck1*^*−/−*^*;cck2*^*−/−*^ double knockout line was established by crossing *cck1*^*−/−*^ and *cck2*^*−/−*^ single mutants. *lhb*^*−/−*^, *fshb*^*−/−*^ and *lhb*^*−/−*^;*fshb*^*−/−*^ lines were established and characterized as previously described^48^. To generate the *cckbrb*^*−/−*^*;tp53*^*−/−*^, *cckbrb*^*+/−*^ heterozygotes were crossed with *tp53*^*+/−*^ (CZ267). The resulting progeny were genotyped and intercrossed over multiple generations to screen for and establish the double homozygous mutant line.

### Histological analysis

Adult fish (WT and mutants) were euthanized, and gonads fixed in Bouin’s solution for a minimum of 24 h. Fixed specimens were dehydrated, embedded in paraffin, and sectioned at 5 μm thickness using a Leica microtome. Sections were stained with hematoxylin and eosin (H&E), dehydrated, mounted, and scanned using a Digital Pathology Slide Scanner (KF-PRX-040; Novelsis, Halkapınar, Izmir, Turkey). Folliculogenesis and spermatogenesis staging followed Mitchell et al.^49^ and were quantified using ImageJ v1.53. Quantitative analysis of folliculogenesis was performed using a proportional staging method. To obtain a representative profile, all follicles across a systematic, non-adjacent series of sections (e.g., every 5th section) spanning the entire ovary were counted and classified into five developmental stages: primary growth (PG), pre-vitellogenic (PV), early-vitellogenic (EV), mid-vitellogenic (MV), and full-grown (FG). The results are expressed as the percentage distribution of follicles across these stages and are designed to compare the relative composition of the follicular population between samples, not to report absolute follicle counts.

### Analysis of courtship behaviors

Courtship behaviors in male zebrafish were evaluated following the methodology described by Mitchell et al. ^49^. The following behavioral parameters were recorded: chase (following or swimming alongside the female), tail-nose contact (touching the female’s side or tail with the male’s nose or head), zig-zag (tail sweeping and circling along the female’s body), encircle (circling around or in front of the female), and quiver (rapid tail oscillation against the female’s side). Ten-minute video recordings were obtained for all four genotypes (Supplementary Movie 1 and 2 are provided as representative 2-minute clips). To rule out general motor deficits as a confounding factor, feeding-driven locomotor activity was assessed in a separate cohort (*n* = 12 per group). Fish were fasted overnight and acclimatized in observation tanks (20 × 25 × 15 cm). Following the introduction of live Artemia to the center of the tank, swimming trajectories were recorded for 1 min and analyzed using Tracker® 6.0 (Open Source Physics) to quantify swimming velocity. Finally, to determine if the courtship defects were caused by androgen deficiency, a pharmacological rescue experiment was performed. Sexually mature male zebrafish (WT and *cckbrb*^*−/−*^) were immersed in system water containing Testosterone (100 ng/L; Catalog No. T5411, Sigma-Aldrich) or vehicle control overnight (12 h). The next morning, males were rinsed, paired with size-matched wild-type females, and their courtship behaviors were recorded for 10 min and quantified as described above.

### Cell transfection and CRE-luciferase reporter assay

To investigate the functional coupling and ligand affinity of zebrafish CCKRs, we utilized a CRE-driven luciferase reporter system to monitor the cAMP/PKA signaling pathway, adapting protocols from previous studies^10^. Briefly, the open reading frames (ORFs) of zebrafish *cckar* (Gene ID: 569038), *cckbra* (Gene ID: 100137117), and *cckbrb* (Gene ID: 100331465) were subcloned into the eukaryotic expression vector pcDNA3.1/Zeo(-), generating CCKR expression constructs. These constructs were transfected into HEK-293T cells (Catalog No. WN-10257, Warner Bio, Wuhan, China) to establish stable cell lines expressing each receptor.

For the reporter assay, the stable cells were transiently transfected with the pGL4.29[luc2P/CRE/Hygro] vector (Promega) containing cAMP response elements (CRE) and the pRL-TK Renilla luciferase vector (Promega) as an internal control. After 24 hours of transfection, cells were treated with graded concentrations of synthetic CCK1 or CCK2 peptides (ranging from 10^−11^ to 10^−5 ^M) for 6 h. Luciferase activity in the lysates was measured using the Dual-Glo™ Luciferase Assay System (E2920, Promega) with a Lumat LB9507 luminometer (EG&G, Gaithersburg, MD, USA). The relative luciferase activity (Firefly/Renilla) was calculated, and EC50 values were determined using non-linear regression analysis (sigmoidal dose-response) in GraphPad Prism.

### Primary cell culture

The hypothalamus and pituitaries of zebrafish were isolated and processed using a trypsin/DNase II/EDTA digestion protocol^10^. Dissociated cells from the hypothalamus and pituitary were seeded in 24-well plates at a density of 1 × 10^5^ cells/mL per well. Initial culturing was performed in plating medium at 28 °C in a 5% CO_2_ environment. After 24 h of cultivation, cells were exposed to specific drugs for an additional 24 h. After 24 h of initial culture, cells were treated with specific peptides to test direct regulation. Primary pituitary cells were incubated with GnRH3 peptide (1 nM, 10 nM) for 24 hours. Hypothalamic cells were treated with CCK peptides (10 nM, 100 nM) for 3 or 24 h. All peptides were synthesized by GenScript (Nanjing, China) with purity >95%. The specific sequences of the non-sulfated octapeptides used in this study were: CCK peptide 1 (DYLGWMDF-NH_2_) and CCK peptide 2 (DYVGWMDF-NH_2_). Gene expression was subsequently analyzed by qRT-PCR.

### Hypothalamic-pituitary (H-P) co-culture and antibody blocking

To dissect the direct and indirect regulation of LH, a transwell co-culture system was established using the primary cells described above. Dispersed hypothalamic cells were seeded onto the semi-permeable membrane of transwell inserts (0.4 µm pore size, Corning), while pituitary cells were seeded in the lower compartment of 24-well plates. Cells were co-cultured in plating medium for 24 hours to allow intercellular communication via soluble factors. For the blocking experiment, the system was treated with: (1) Vehicle control; (2) CCK peptide alone (10 nM); or (3) CCK peptide combined with an affinity-purified GnRH3-neutralizing antibody (1:500 dilution; custom-synthesized by Dia-An, Wuhan). After 24 h of treatment, pituitary cells in the lower chamber were harvested for RNA extraction and *lhb* expression analysis via qRT-PCR. While the functional blockade of GnRH3 was performed using an affinity-purified antibody compared against a vehicle (PBS) control, we acknowledge that the absence of an identically processed non-immune IgG control limits our ability to completely exclude potential non-specific effects of co-purified serum components. Therefore, the interpretation of the GnRH blockade results should be approached with appropriate caution.

### Immunostaining, in situ hybridization, and apoptosis analysis

#### Immunostaining and apoptosis assay

To visualize PGCs in vivo, whole-mount immunostaining was performed at 24 hpf using the Piwil1 antibody (CZPA2, CZRC; 1:500) as the primary antibody and Alexa Fluor 594 goat anti-rabbit IgG (Yeasen, 1:200) as the secondary antibody, following the method described by Tao et al. ^50^. For apoptosis detection, Terminal deoxynucleotidyl transferase dUTP nick end labeling (TUNEL) analysis was conducted using the TUNEL Apoptosis Detection Kit (YSFluor 488, Yeasen, China) according to the manufacturer’s protocol. Whole-mount fluorescent images were captured using a fluorescence stereomicroscope (M205FA, Leica)^51^.

#### Tissue section immunofluorescence (IF)

For immunofluorescence analysis of the zebrafish hypothalamus, pituitary, or testes, tissue sections were fixed with 4% paraformaldehyde (PFA). After three washes with cold PBS, samples were blocked with blocking solution (PBS containing 0.1% Triton-100, 1% BSA, and 1% DMSO) for at least 1 hour. Primary antibodies were diluted in the blocking solution and incubated with the samples overnight at 4 °C. After washing with PBS, sections were incubated with secondary antibodies conjugated with Alexa Fluor® 488 or 594 (Yeasen). Nuclei were counterstained with DAPI.

#### Combined fluorescence in situ hybridization (FISH) and immunofluorescence (IHC)

To co-localize *cckbrb* mRNA with LH protein, a sequential FISH and IHC protocol was performed. First, FISH was conducted using a digoxigenin (DIG)-labeled RNA probe specific for *cckbrb* (Probe sequence provided in Supplementary Table 2). Following the FISH procedure, sections were washed and blocked for IHC. Samples were then incubated with the anti-LH primary antibody (1:500) overnight at 4 °C, followed by the appropriate secondary antibody.

#### Antibodies and Imaging

The following commercial antibodies were used: anti-CCK (C2581, Merck; 1:1000), anti-Vasa (2008, Dia-An; 1:800), anti-SYCP3 (CZPA3, CZRC; 1:1000), anti-SOX9a (ab209820, Abcam; 1:1000), anti-Histone H3 (ab183626, Abcam 1:1000), and anti-PCNA (ab29, Abcam; 1:1000). The anti-GnRH3 (1:500), anti-LH (1:500), and anti-FSH (1:500) antibodies were custom synthesized by Dia-An (Wuhan). The specificity of these antibodies was empirically validated via Western blot or antigen pre-absorption tests. Confocal images for tissue sections were captured using a confocal microscope (FV3000, Olympus).

### Embryonic CCK rescue

The rescue protocol was adapted from Zhai et al. ^52^. Briefly, embryos, generated from *cck1*^*+/−*^*;cck2*^*+/−*^ heterozygous crosses, were then incubated in E3 medium containing a CCK cocktail (0.02 µg/mL CCK1 and 0.02 µg/mL CCK2). The control group was treated with the same concentration of DMSO. All experimental groups were processed at 14 dpf. Tail fins were excised from each fish for genotyping, and gonadal cells were subjected to immunohistochemical analysis to detect the expression of SYCP3 protein in the gonadal cells.

### CCK feed supplementation

Briefly, CCK1 (1 mg) and CCK2 (1 mg) were dissolved separately in 1 mL dimethyl sulfoxide (DMSO), then diluted to 5 mL with anhydrous ethanol. The mixture was evenly sprayed onto the surface of 10 g commercial fish feed and left at room temperature for 15 min to allow for adequate adsorption. The feed was then covered with aluminum foil and incubated in a 30 °C incubator for 10 h to stabilize the active components on the feed. During the experiment, the daily feeding amount was set at 3% of the fish body weight. Feeding was carried out at regular intervals following standard aquaculture procedures to ensure stable consumption. Prior to each feeding, the treated feed was thoroughly mixed to ensure consistent dosing across all feedings.

### Quantification of CCK absorption (ELISA)

To validate the effective absorption of exogenous CCK, peptide concentrations were measured using a fish-specific Cholecystokinin (CCK) ELISA kit (Catalog No. CSB-E13054Fh-1, Cosmo Bio USA).

#### For feeding supplementation

Adult zebrafish were fed either the basal diet or the CCK-supplemented diet. Blood samples were collected 1.5 hours post-feeding. Blood from ten individual fish was pooled to constitute one biological replicate (*n* = 3). Plasma was separated by centrifugation at 861 × *g* for 10 min at 4 °C, and CCK concentration was quantified following the manufacturer’s instructions.

#### For larval immersion

Zebrafish larvae at 48 hpf were subjected to waterborne CCK immersion for 1 h. Following treatment, larvae were washed thoroughly three times with fresh E3 medium. 20–30 larvae were pooled for each biological replicate (*n* = 3) and homogenized to measure internal CCK concentration.

### Feeding rate

One day prior to the experiment, fish were individually weighed and placed into numbered transparent plastic bowls for acclimatization in a light-controlled incubator. The feeding amount was set at 6% of the fish body weight, and feeding occurred at 9:00 A.M. the following day. One hour after feeding, residual food was collected using a pipette, dried, and weighed. The feeding rate was calculated using the formula: Feeding rate (%) = [(Amount of feed provided—Amount of residual feed)/Body weight] × 100%.

### Intraperitoneal injection

#### Zebrafish experiments

CCK1 and CCK2 peptides were diluted in 0.65% fish physiological saline to a final concentration of 0.1 μg/μL. A dosage of 1 μg per fish was prepared for injection. Three-month-old wild-type zebrafish were randomly assigned to experimental groups (*n* = 32 per group), with an equal distribution of 16 males and 16 females. After 24 h of fasting, fish were anesthetized with 0.05% MS222 and received intraperitoneal injections using a microinjection syringe. Following injection, fish were immediately returned to their tanks for continued maintenance. After 24 h, fish were collected. Due to their small size, four pituitaries were pooled to constitute a single sample, which was then rapidly frozen in liquid nitrogen and stored for RNA extraction and RT-qPCR analysis.

#### Mouse experiments

Intraperitoneal injections followed a modified protocol based on Motojima et al.^53^. CCK-8 (Peptide Institute, Osaka, Japan) was dissolved in 0.9% sterile physiological saline. Mice were divided into two groups (*n* = 12 per group) receiving either saline as a control or CCK-8 (50 μg/kg body weight). Pituitary glands were collected under anesthesia at 6 and 12 h post-injection, respectively.

### Ovulation induction experiment

The experiment was adapted and optimized from Tang et al. ^54^. Healthy female zebrafish were selected and maintained in 3 L tanks at a density of 4 fish per liter under controlled conditions (28 °C, 14:10 light/dark cycle). On the experiment day, two fish were anesthetized with MS222, and their body weights were precisely measured using an analytical balance. Different treatments were administered intraperitoneally using a 32-gauge Hamilton syringe: the control group was injected with Ringer’s solution (pH 7), while the experimental groups received CCK1 (1 μg/g body weight) and LH (200 ng/g body weight). The LH utilized was recombinant zebrafish LH protein (Beijing VJTBio Co.,LTD, Beijing, China). The injection doses for CCK1 and LH were determined based on previous experiments exploring spawning induction within the range of 0.1–4 μg/g.

After injection, the fish were returned to the breeding tanks and allowed to recover for 2–3 h. Spawning was induced by gentle abdominal pressure, and successful ovulation was confirmed when eggs were expelled. A total of 14 female zebrafish were used per group, with three replicate experiments conducted. The number of successful ovulations per group was recorded to assess the effects of different treatments on reproductive characteristics.

### qRT-PCR

Total RNA was extracted from zebrafish tissue using the Trizol method (Invitrogen). cDNA was synthesized using the HiFiScript All-in-One RT Master Mix for qPCR kit (CWBIO, Beijing). Primer sequences are provided in Supplementary Table 2. PCR amplification was performed using SuperStar Universal SYBR Master Mix (CWBIO, Beijing) on an ABI 7500 Real-Time PCR System. The thermal cycling conditions were as follows: 95 °C for 5 min, followed by 45 cycles of 95 °C for 10 seconds (denaturation), 60 °C for 10 s (annealing), and 72 °C for 10 s (extension). *ef1a* was used as the internal control. Each sample was performed in triplicate technical repeats.

### Single-cell RNA-seq library construction and sequencing

For zebrafish scRNA-seq, 6-month-old male zebrafish from the *cck1*^*−/−*^*;cck2*^*−/−*^ (weight = 366 ± 35 mg) and WT (weight = 312 ± 27 mg) groups were selected (*n* = 30 per group). Pituitary and hypothalamic tissues were dissected, washed in ice-cold minimal essential medium, and dissociated using trypsin, EDTA, and DNase II. After cell counting, cells were washed twice in NeuroGro medium (T710KJ, BasalMedia, Shanghai, China) and resuspended in PBS at a concentration of 1 × 10⁶ cells per mL. Samples were sent to OE Biotech (Shanghai, China) for scRNA-seq. Following preliminary quality control using CellRanger v5.0.0, further quality control and processing were performed using the Seurat package (version 4.1.0), with specific sequencing methods and analysis described in Supplementary Table 3. The scRNA-seq data are available in NCBI’s Gene Expression Omnibus (GEO) under the accession numbers GSE296008 and GSE295139. Additionally, single-cell transcriptomics of 7-week-old adult male mouse pituitary cells from the Gene Expression Omnibus (GEO) database under the accession number GSE120410^55^.

### Data transformation and statistical analysis

Transcription levels were measured by qRT-PCR using the ABI 7500 SDS v1.5.1 software, and calibrated using a standard curve (dynamic range: 10⁵, correlation coefficient: >0.95). The housekeeping gene *ef1a* was used as the internal control, and target gene mRNA levels were normalized and expressed as percentages of the control group (“%Ctrl”). Data from 4 to 8 replicates (mean ± SEM) were analyzed by one-way ANOVA to assess significant differences between treatments. Post-hoc analysis was performed using Dunnett’s test in SPSS Statistics 26.0 (Chicago, IL, USA).

### Sample size determination

No statistical methods were used to predetermine sample size. Sample sizes were chosen based on common practice in the field, previous experience with similar zebrafish experiments, and sample availability. For each experiment, the sample size and the definition of *n* are provided in the corresponding figure legends or Methods section. Where applicable, experiments were independently repeated with similar results to ensure reproducibility.

### Reporting summary

Further information on research design is available in the Nature Portfolio Reporting Summary linked to this article.

## Supplementary information

Supplementary Information

Description of Additional Supplementary Files

Supplementary Movie 1

Supplementary Movie 2

Reporting Summary

Transparent Peer Review file

## Source data

Source Data

## Data Availability

The raw bulk RNA-seq data generated in this study have been deposited in the NCBI BioProject database under accession code PRJNA1441806. The scRNA-seq data generated in this study have been deposited in the NCBI Gene Expression Omnibus (GEO) under accession codes GSE296008 and GSE295139. The published single-cell transcriptomic dataset analysed in this study is available in GEO under accession code GSE120410. Other data supporting the findings of this study are available within the paper, its Supplementary Information, and the Source Data file. Source data are provided with this paper.
