# Supplementary material for: Cholecystokinin coordinates gonadotropin-dependent and independent pathways to orchestrate zebrafish gonadal development
Source: Nat Commun. 2026 Apr 18;17:5392. doi: 10.1038/s41467-026-72039-x (PMC13275923; doi:10.1038/s41467-026-72039-x)
Supplement: Supplementary file 1 — Supplementary Information [file 41467_2026_72039_MOESM1_ESM.pdf]

Supplementary Information for

Cholecystokinin coordinates gonadotropin-dependent and independent pathways to orchestrate zebrafish gonadal development

Supplementary Figures

Supplementary Fig. 1 | Expression profiles of zebrafish CCK ligands and CCK receptors across various tissues.

Supplementary Fig. 2 | Gene structure of *cckbrb* and CRISPR/Cas9 targeting strategy.

Supplementary Fig. 3 | Analysis of mating behaviors in *cck1*$^{-/-}$;*cck2*$^{-/-}$ zebrafish.

Supplementary Fig. 4 | Locomotor activity driven by feeding stimulus in in *cck1*$^{-/-}$;*cck2*$^{-/-}$ and *cckbrb*$^{-/-}$ zebrafish.

Supplementary Fig. 5 | Testicular androgen levels in *cckbrb*$^{-/-}$ zebrafish.

Supplementary Fig. 6 | Testosterone immersion rescues the impaired courtship behaviors in *cckbrb*$^{-/-}$ zebrafish.

Supplementary Fig. 7 | Dot plot revealing distinct marker gene expression patterns across zebrafish pituitary cell populations.

Supplementary Fig. 8 | Dynamics of stemness gene expression during gonadotrope differentiation.

Supplementary Fig. 9 | Confirmation of CCK feeding efficacy and resulting impact on pituitary *fshb* expression.

Supplementary Fig. 10 | Effects of CCK-containing diet on gonad development in wild-type and *fshb*$^{-/-}$ zebrafish.

Supplementary Fig. 11 | Immunohistochemical staining of GnRH3 protein in hypothalamic sections from male *cckbrb*$^{+/+}$ and *cckbrb*$^{-/-}$ zebrafish.

Supplementary Fig. 12 | Single-cell RNA sequencing analysis of the zebrafish hypothalamus.

Supplementary Fig. 13 | Effects of GnRH3 on gonadotropin expression in pituitary cells *in vitro*.

Supplementary Fig. 14 | Hypothalamic-Pituitary (H-P) co-culture system and functional assessment of dual CCK regulation.

Supplementary Fig. 15 | Co-localization of *cckbrb* mRNA and LH protein in the adult male zebrafish pituitary gland.

Supplementary Fig. 16 | Cholecystokinin-8 does not regulate pituitary *lhb* and *fshb* gene expression in male mice.

Supplementary Fig. 17 | Morphological and histological analysis of gonads in *lhb* and *fshb* knockout zebrafish models.

Supplementary Fig. 18 | Immunofluorescence co-expression analysis of VASA and SYCP3 proteins in the testes of *lhb* and *fshb* single and double knockout zebrafish models.

Supplementary Fig. 19 | Sex ratio and mating success in *lhb* and *fshb* knockout zebrafish models.

Supplementary Fig. 20 | Immunofluorescence analysis of germ cell proliferation in *cckbrb*$^{-/-}$ zebrafish at different developmental stages.

Supplementary Fig. 21 | Representative western blots showing the expression levels of Histone H3 in gonads from Wild-type (WT) and *cckbrb*$^{-/-}$ zebrafish at 45, 90, and 180 days post fertilization (dpf).

Supplementary Fig. 22 | Co-expression analysis of VASA and SYCP3 in gonads of WT, *fshb*$^{-/-}$, *lhb*$^{-/-}$;*fshb*$^{-/-}$, and *cckbrb*$^{-/-}$ zebrafish.

Supplementary Fig. 23 | Western blot analysis of the meiotic marker SYCP3 in WT, *fshb*$^{-/-}$, *lhb*$^{-/-}$;*fshb*$^{-/-}$, and *cckbrb*$^{-/-}$ zebrafish.

Supplementary Fig. 24 | Temporal analysis of meiosis in testicular tissue at 60, 120, and 150 dpf in WT and *cck1*$^{-/-}$;*cck2*$^{-/-}$ zebrafish using VASA/SYCP3 co-staining.

Supplementary Fig. 25 | Quantification of internal CCK concentration in larval zebrafish following waterborne immersion.

Supplementary Fig. 26 | Analysis of SYCP3 expression in *cckbrb*$^{-/-}$;*tp53*$^{+/+}$ and *cckbrb*$^{-/-}$;*tp53*$^{-/-}$ zebrafish gonads.

Supplementary Tables

Supplementary Table 1 | EC_{50} Values for Zebrafish CCK Receptors.

Supplementary Table 2 | The primers used in the study.

Supplementary Table 3 | Single-cell RNA sequencing and analysis.

Supplementary Fig. 1

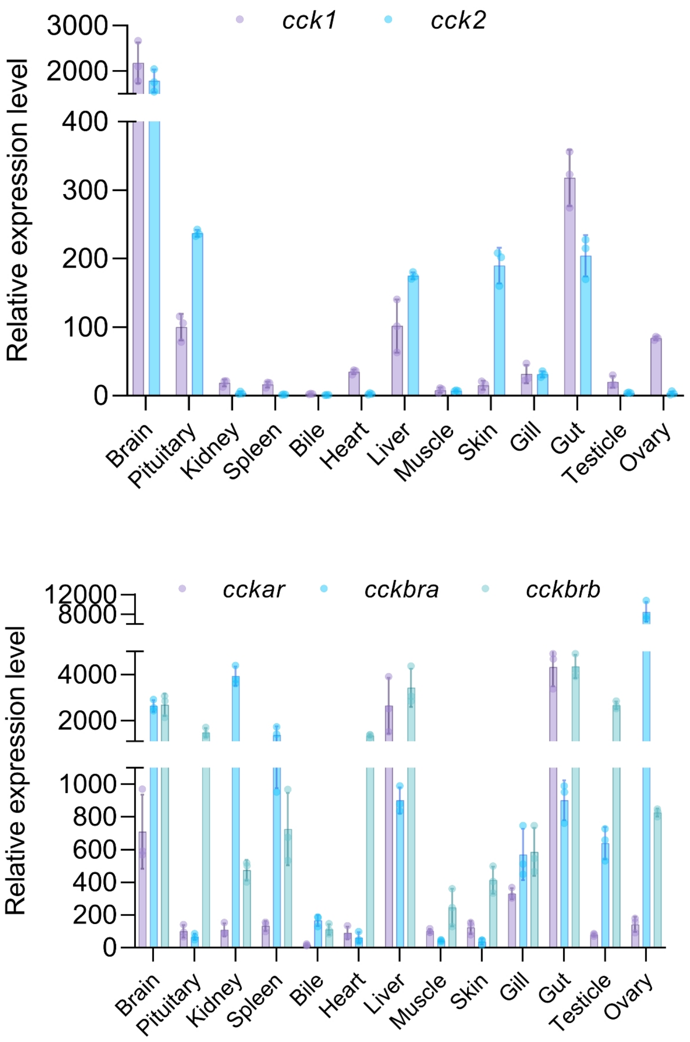

Supplementary Fig. 1 | Expression profiles of zebrafish CCK ligands and CCK receptors across various tissues. The relative mRNA expression levels of CCK ligands (*cck1, cck2*) and CCK receptors (*cckar, cckbra, cckbrb*) were quantified in adult zebrafish tissues. Data were determined by quantitative real-time PCR (qPCR) and normalized to the endogenous reference gene, elongation factor 1a (*ef1a*). Expression levels are presented as Relative Expression (Arbitrary Units). Data are shown as mean ± s.e.m. (standard error of the mean) for *n* = 3 independent biological replicates. For pituitary, gallbladder, and kidney, each replicate was composed of four pooled organs from different individuals.

Supplementary Fig. 2

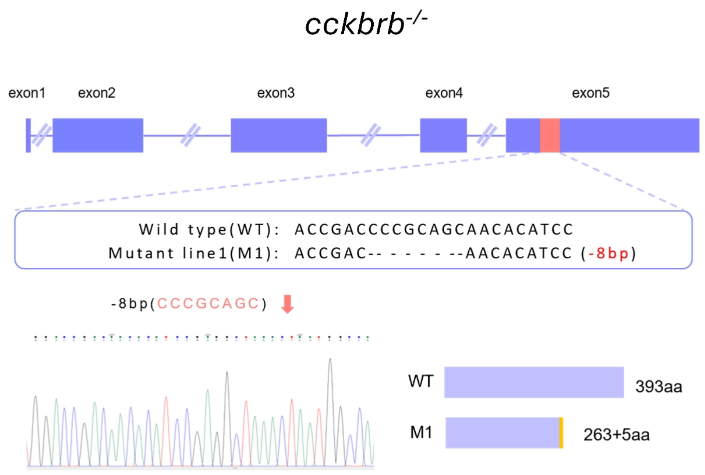

Supplementary Fig. 2 | Gene structure of *cckbrb* and CRISPR/Cas9 targeting strategy. Schematic diagram illustrating the genomic structure of the zebrafish *cckbrb* gene, which consists of five exons. The CRISPR/Cas9-mediated targeting strategy was designed using a guide RNA (gRNA) targeting Exon 5 (indicated by an arrow). This targeting resulted in an 8 base pair (bp) deletion (△8 bp) at the gRNA site. This mutation is predicted to induce a frameshift and generate a premature termination codon (PTC), leading to a functional *cckbrb* knockout line. The location of the gRNA target site and the primers used for genotyping are indicated.

Supplementary Fig. 3

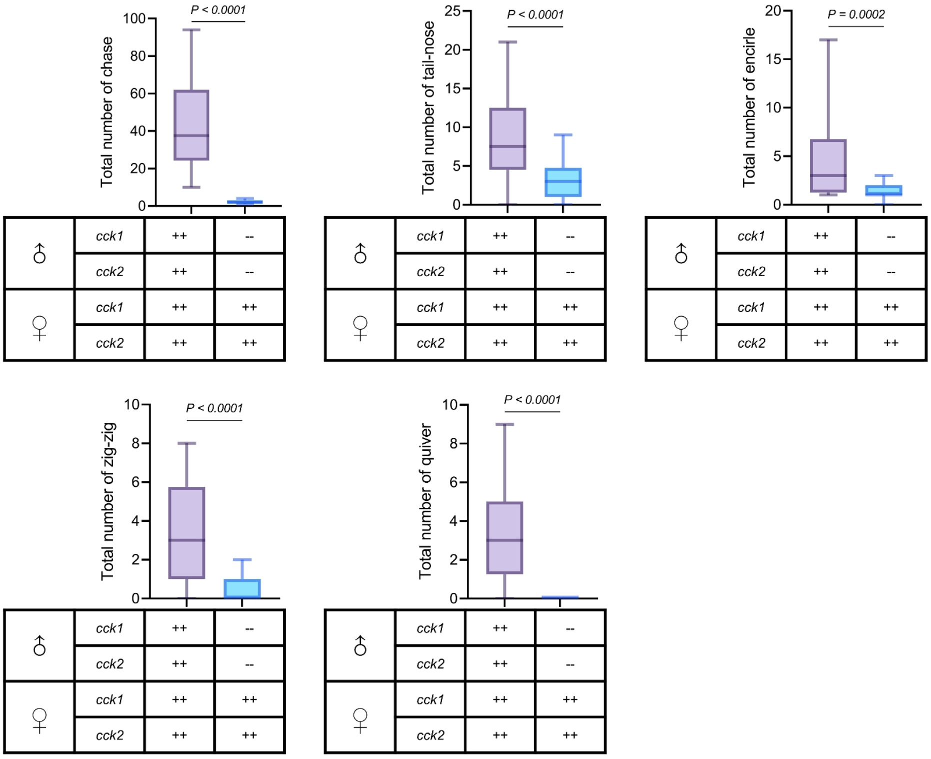

Supplementary Fig. 3 | Analysis of mating behaviors in *cck1*$^{-/-}$;*cck2*$^{-/-}$ zebrafish. Schematic representation of typical male zebrafish courtship behaviors, including Chase, Tail-nose, Encircle, Zig-zag, and Quiver. Box plots showing the frequency of each courtship behavior performed by males of different genotypes during a 10-minute observation period. Data are presented as median, interquartile range (IQR), and min-max values (*n* = 24 per group).

Supplementary Fig. 4

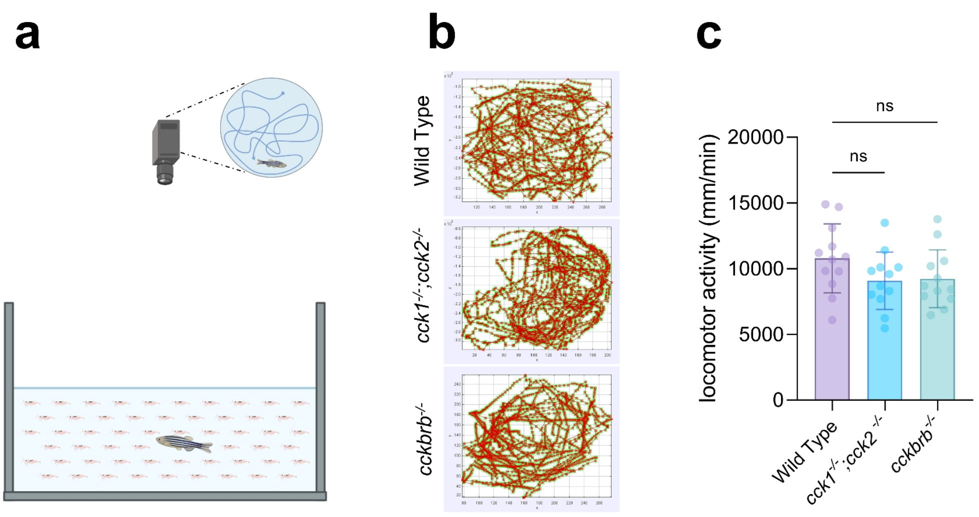

Supplementary Fig. 4 | Locomotor activity driven by feeding stimulus in *cck1*$^{-/-}$;*cck2*$^{-/-}$ and *cckbrb*$^{-/-}$ zebrafish. a Experimental scheme. Created in BioRender. Li, H. (2026) https://biorender.com/qm1v0y9. **b** Representative swimming traces of wild-type, *cck1*$^{-/-}$;*cck2*$^{-/-}$ and *cckbrb*$^{-/-}$ zebrafish. **c** Quantification of feeding-driven locomotor activity. Data are presented as mean ± s.e.m. from n = 12 independent fish per group. Statistical significance was determined by one-way ANOVA followed by Dunnett's multiple-comparisons test.

Supplementary Fig. 5

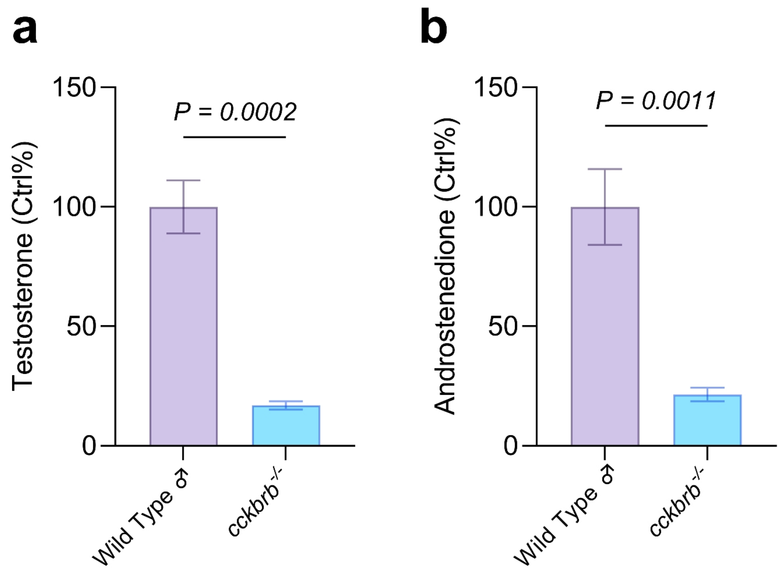

Supplementary Fig. 5 | Testicular androgen levels in *cckbrb*$^{-/-}$ zebrafish. The levels of key androgen hormones were quantified in the testes of *cckbrb*$^{-/-}$ and wild-type (WT) male zebrafish. **a** Relative levels of testosterone in WT and *cckbrb*$^{-/-}$ testes. **b** Relative levels of androstenedione in WT and *cckbrb*$^{-/-}$ testes. Androgen quantification was performed using metabolomic analysis. Data were normalized to the mean value of the Wild-type (WT) male testes group. Data are presented as the mean ± s.d. (standard deviation) for n = 3 independent biological replicates. Each biological replicate represents a pooled sample composed of the testes from ten individual male zebrafish. Statistical significance was determined by two-sided unpaired Student's t-test.

Supplementary Fig. 6

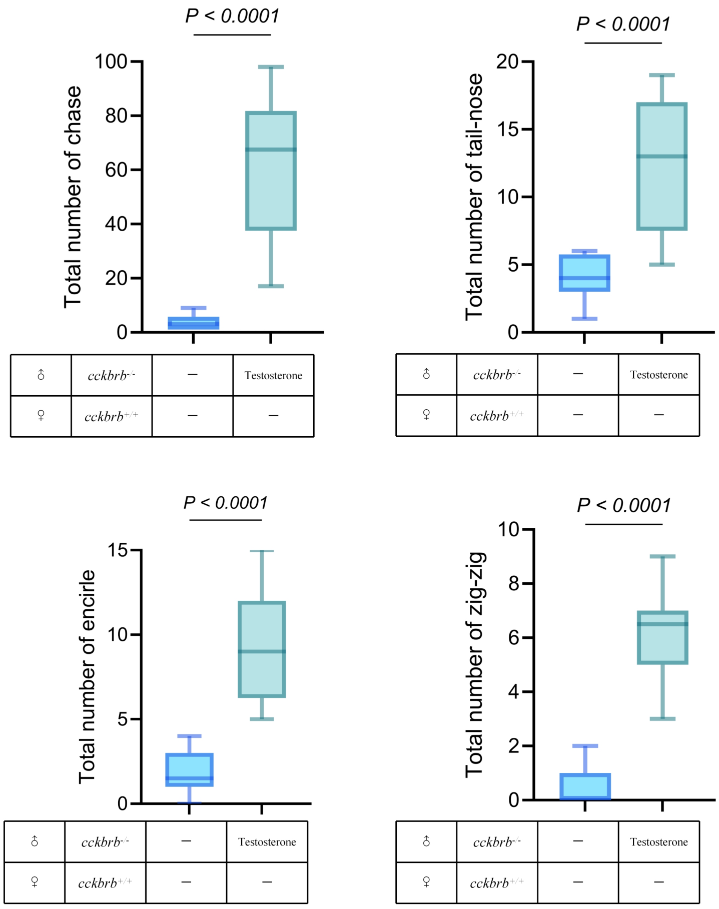

Supplementary Fig. 6 | Testosterone immersion rescues the impaired courtship behaviors in *cckbrb*$^{-/-}$ zebrafish. The effect of exogenous testosterone on male courtship behavior was assessed in wild-type (WT) and *cckbrb*$^{-/-}$ zebrafish. Male fish were treated with testosterone immersion prior to behavioral assessment. The figure shows the frequency of each courtship behavior (chase, tail-nose, encircle and zig-zag) performed by males of different genotypes and treatments during a 10-min observation period. Data are presented using box plots, where the central line indicates the median, the box boundaries represent the interquartile range (IQR), and the whiskers denote the minimum and maximum values. The sample size for each group is n = 24 independent pairings. Statistical significance was determined by two-sided unpaired Student's t-test. Source data are provided as a Source Data file.

Supplementary Fig. 7

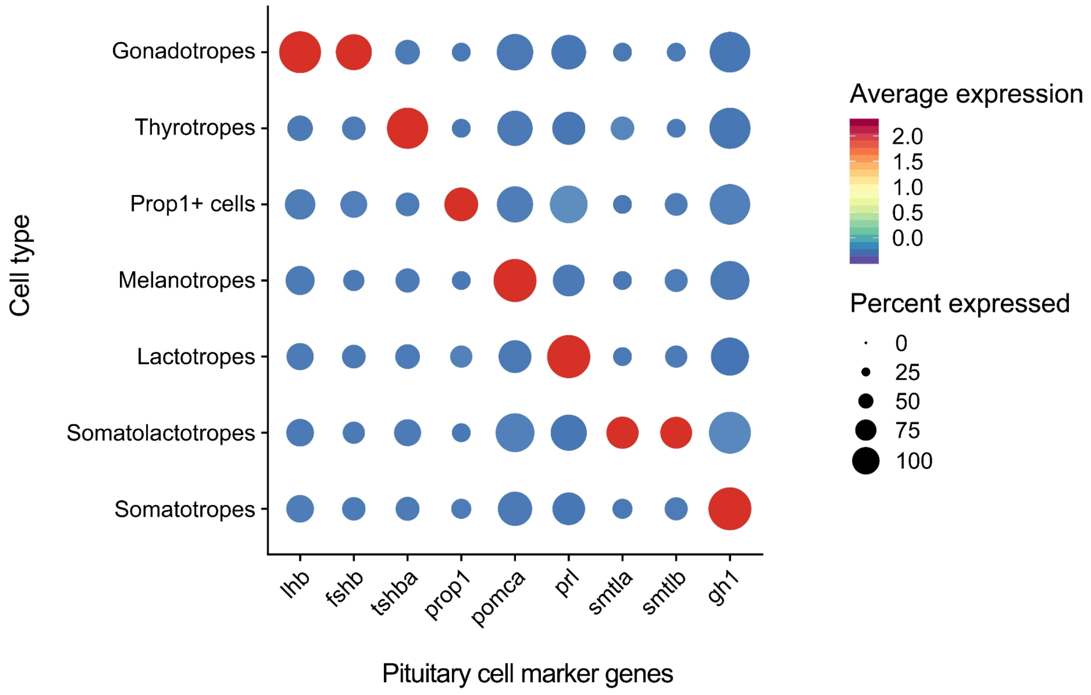

Supplementary Fig. 7 | Dot plot revealing distinct marker gene expression patterns across zebrafish pituitary cell populations. This visualization confirms the identity of seven major pituitary cell lineages based on their expression of established canonical marker genes. The color intensity of the dots represents the average gene expression level, while the size of the dots indicates the percentage of cells expressing the respective gene within each designated cell type.

Supplementary Fig. 8

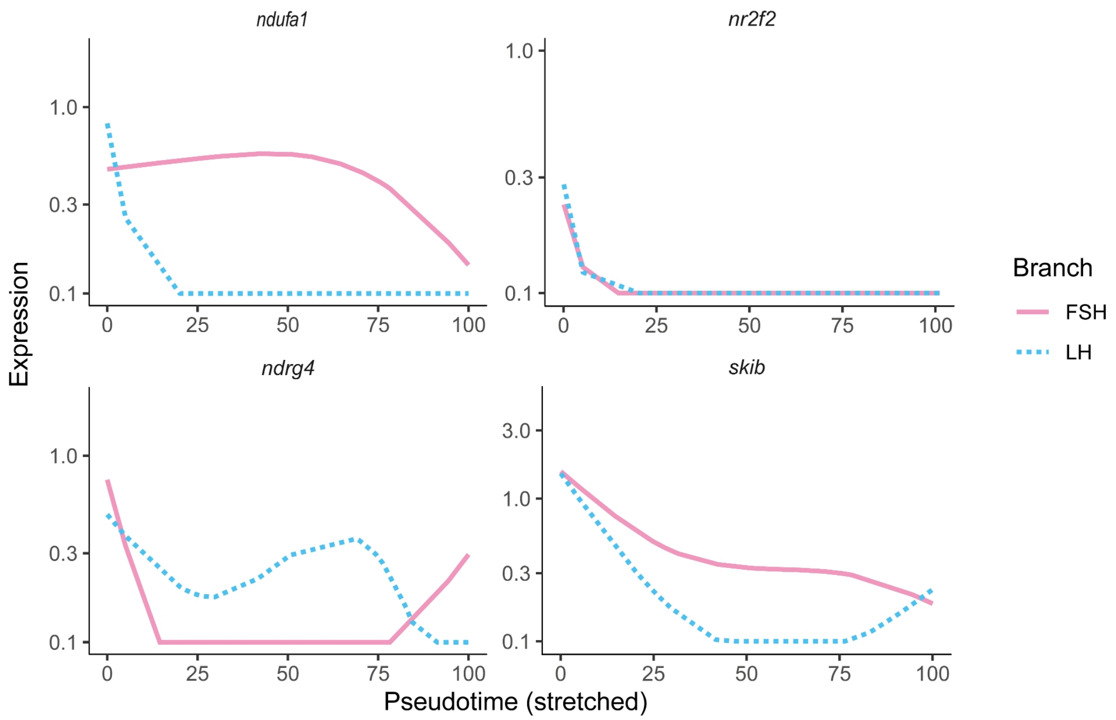

Supplementary Fig. 8 | Dynamics of stemness gene expression during gonadotrope differentiation. The figure shows the relative expression levels of the stemness-related genes *ndufa1*, *nr2f2*, *ndrg4*, and *skib* across the pseudotime trajectory of gonadotrope differentiation, determined by pseudotime analysis. The two trajectories are shown as FSH (pink solid line) and LH (blue dotted line) differentiation branches.

Supplementary Fig. 9

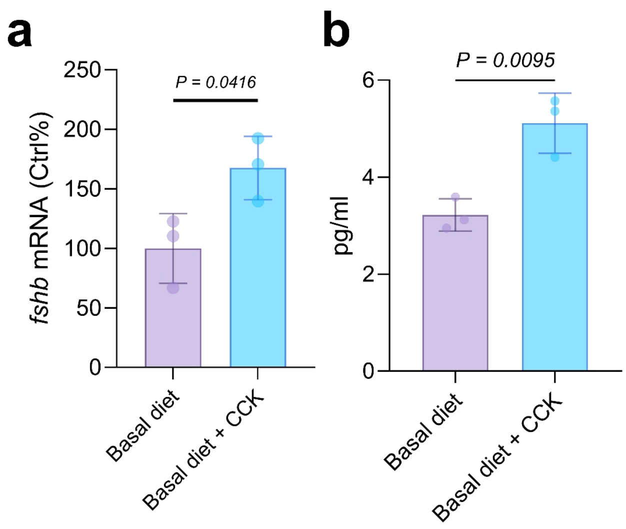

Supplementary Fig. 9 | Confirmation of CCK feeding efficacy and resulting impact on pituitary *fshb* expression. The figure confirms the effective absorption of dietary CCK and its subsequent biological effect, measured 1.5 hours post-feeding with a CCK-supplemented diet versus a normal diet. **a** Relative mRNA expression level of *fshb* in the pituitary gland (determined by qPCR). Data are presented as mean ± s.e.m. for $n = 3$ independent replicates. Each replicate is a pool of four pituitaries. **b** Plasma CCK concentration (determined by ELISA). Data are presented as mean ± s.e.m. for $n = 3$ independent replicates. Each replicate is a pool of blood from ten individual fish. Statistical significance was determined by two-sided unpaired Student's *t*-test.

Supplementary Fig. 10

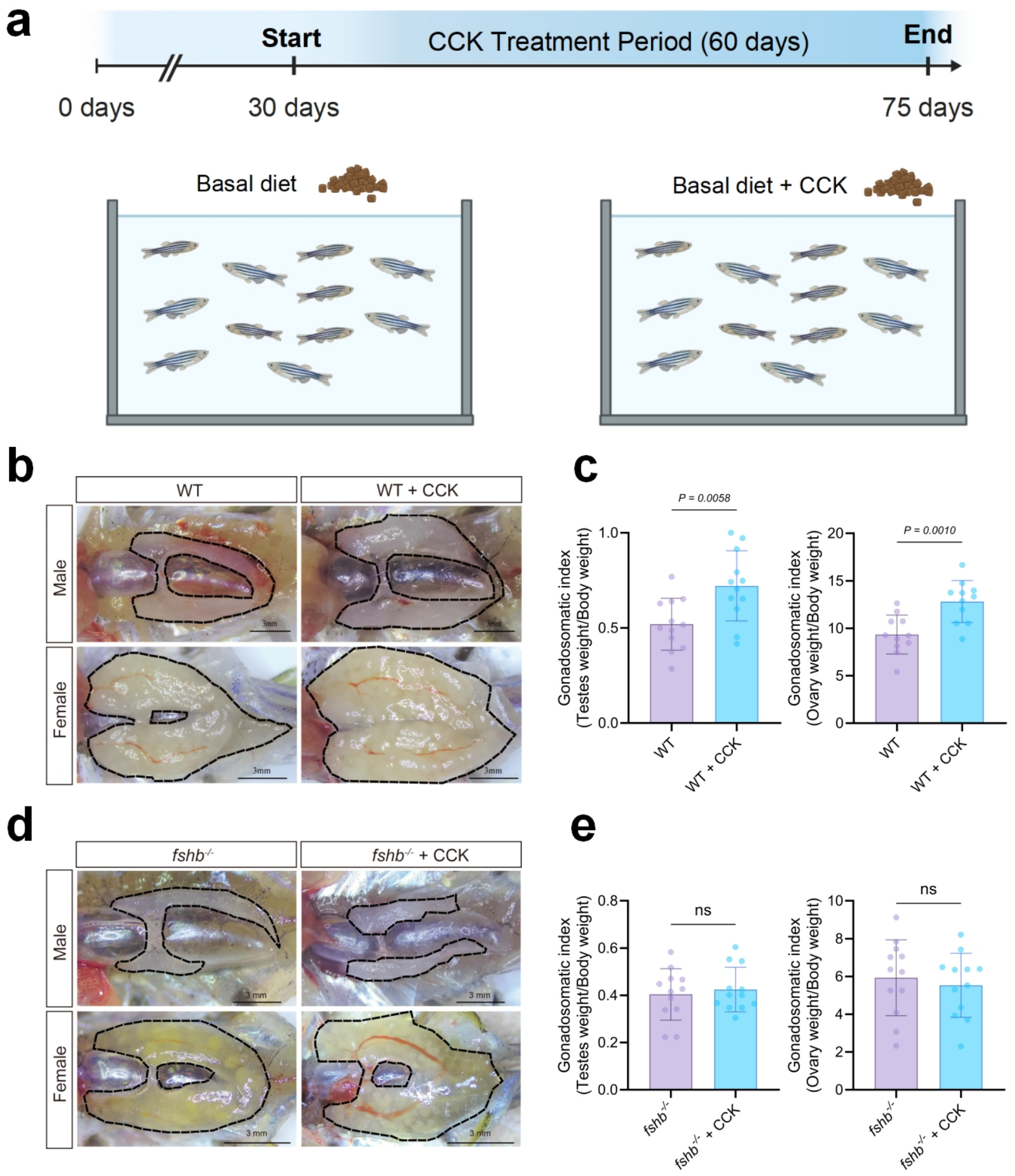

Supplementary Fig. 10 | Effects of CCK-containing diet on gonad development in wild-type and *fshb*$^{-/-}$ zebrafish. a Schematic diagram of the experimental timeline. Fish were fed the CCK diet for 45 days (from 30 to 75 dpf). Created in BioRender. Li, H. (2026) https://biorender.com/v7qg9n0. **b, d** Representative images of gonad morphology in Wild-type (WT) (**b**) and *fshb*$^{-/-}$ (**d**) zebrafish after the feeding period. Representative images from four independent experiments with similar results are shown. Scale bars, 3 mm. **c, e** Gonadosomatic Index (GSI) comparison in WT (**c**) and *fshb*$^{-/-}$ (**e**) male and female fish. Data are presented as mean ± s.e.m. for n = 12 independent fish per group. Statistical significance was determined by two-sided unpaired Student's t-test.

Supplementary Fig. 11

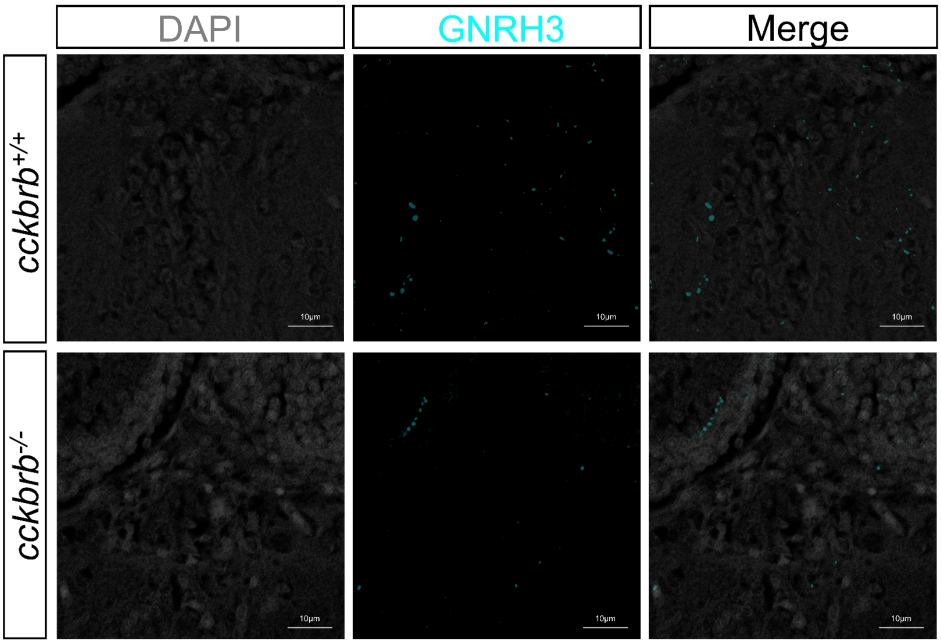

Supplementary Fig. 11 | Immunohistochemical staining of GnRH3 protein in hypothalamic sections from male *cckbrb*$^{+/+}$ and *cckbrb*$^{-/-}$ zebrafish. Representative images of Immunohistochemical (IHC) staining for GnRH3 protein in hypothalamic sections of male *cckbrb*$^{+/+}$ and *cckbrb*$^{-/-}$ zebrafish. Representative images from four independent experiments with similar results are shown.

Supplementary Fig. 12

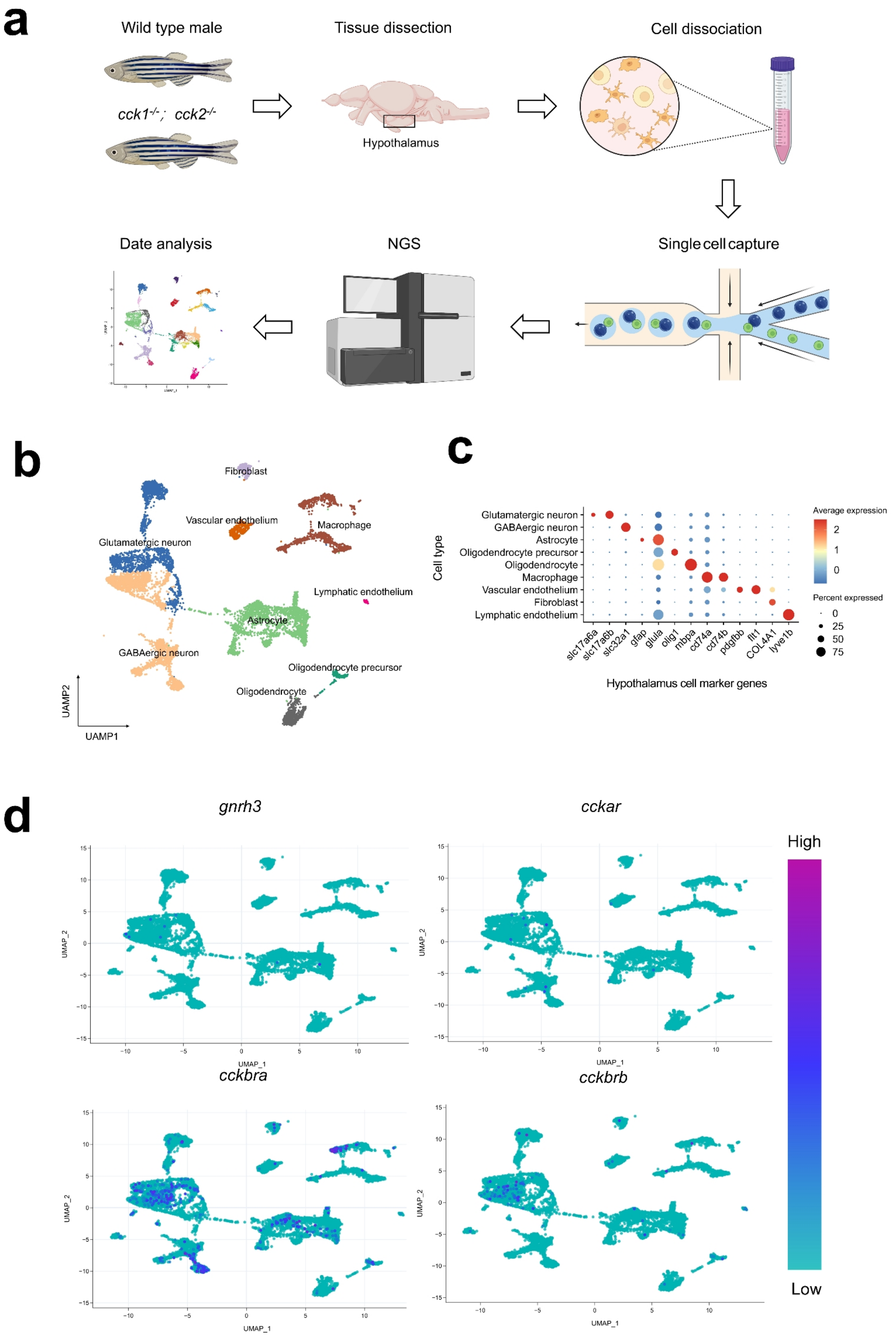

Supplementary Fig. 12 | Single-cell RNA sequencing analysis of the zebrafish hypothalamus. **a** Schematic diagram of zebrafish hypothalamus single-cell RNA sequencing (scRNA-seq) workflow. Created in BioRender. Li, H. (2026) https://biorender.com/ggl0y1d. **b** UMAP visualization of hypothalamic cells, revealing nine major cell clusters. **c** Dot plot showing expression patterns of established marker genes used to annotate cell types. **d** UMAP expression distribution of *cckar*, *cckbra*, *cckbrb*, and *gnrh3* genes in zebrafish hypothalamic cells.

Supplementary Fig. 13

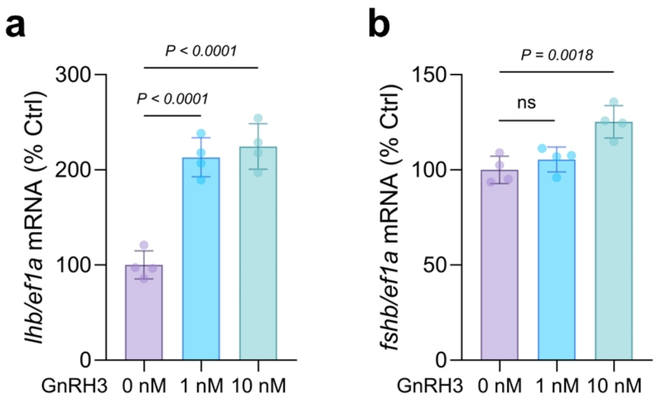

Supplementary Fig. 13 | Effects of GnRH3 on gonadotropin expression in pituitary cells *in vitro*. a qPCR analysis of relative *lhb* mRNA expression in primary cultured zebrafish pituitary cells treated with graded concentrations of GnRH3 (0, 1, and 10 nM) for 24 hours. **b** qPCR analysis of relative *fshb* mRNA expression under the same experimental conditions. Data are presented as mean ± s.d. for n = 4 independent biological replicates. Expression levels were normalized to *ef1a*. Statistical significance was determined by one-way ANOVA followed by Dunnett's multiple-comparisons test.

Supplementary Fig. 14

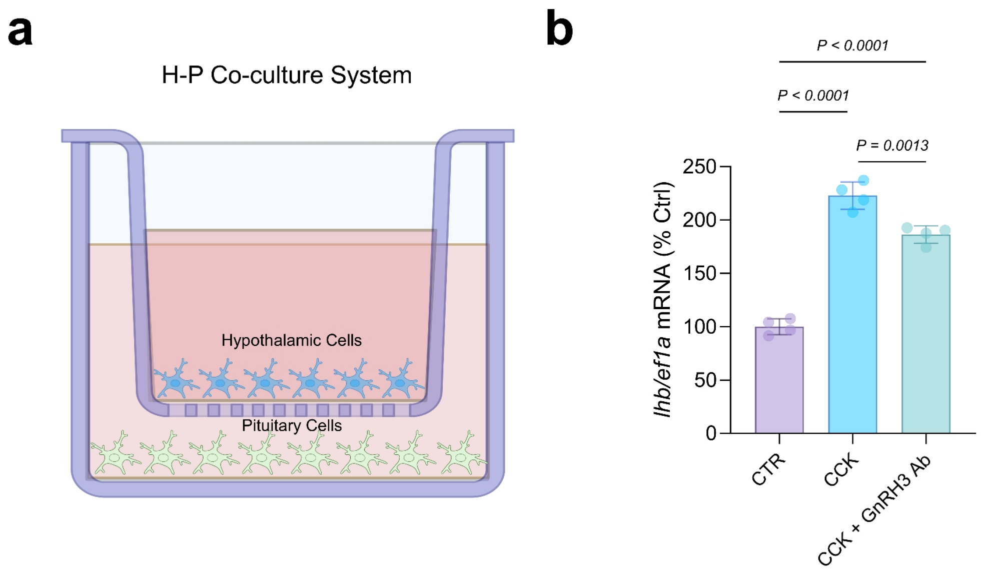

Supplementary Fig. 14 | Hypothalamic-Pituitary (H-P) co-culture system and functional assessment of dual CCK regulation. a Schematic diagram of the H-P co-culture system. Hypothalamic cells are cultured on the transwell insert membrane, and pituitary cells are cultured in the lower chamber, facilitating the transfer of soluble factors. Created in BioRender. Li, H. (2026) https://biorender.com/hgl9icn. **b** qPCR analysis of *lhb* mRNA expression in pituitary cells from the co-culture system following treatment with Control (Ctrl), CCK only (CCK), and CCK with GnRH3 blocking antibody (CCK + GnRH3 Ab). Data are presented as mean ± s.e.m. (standard error of the mean) for n = 4 independent experiments. Statistical significance was determined by one-way ANOVA followed by Tukey's multiple-comparisons test.

Supplementary Fig. 15

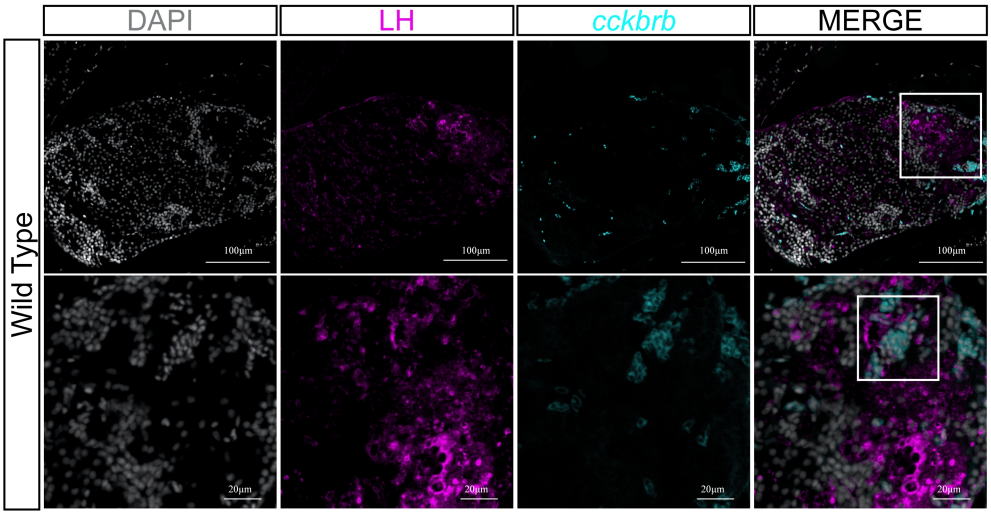

Supplementary Fig. 15 | Co-localization of *cckbrb* mRNA and LH protein in the adult male zebrafish pituitary gland. *cckbrb* mRNA was detected using a fluorescent probe (via Fluorescence *In Situ* Hybridization), and LH protein was visualized via immunohistochemistry (IHC). Representative images from three independent experiments with similar results are shown.

Supplementary Fig. 16

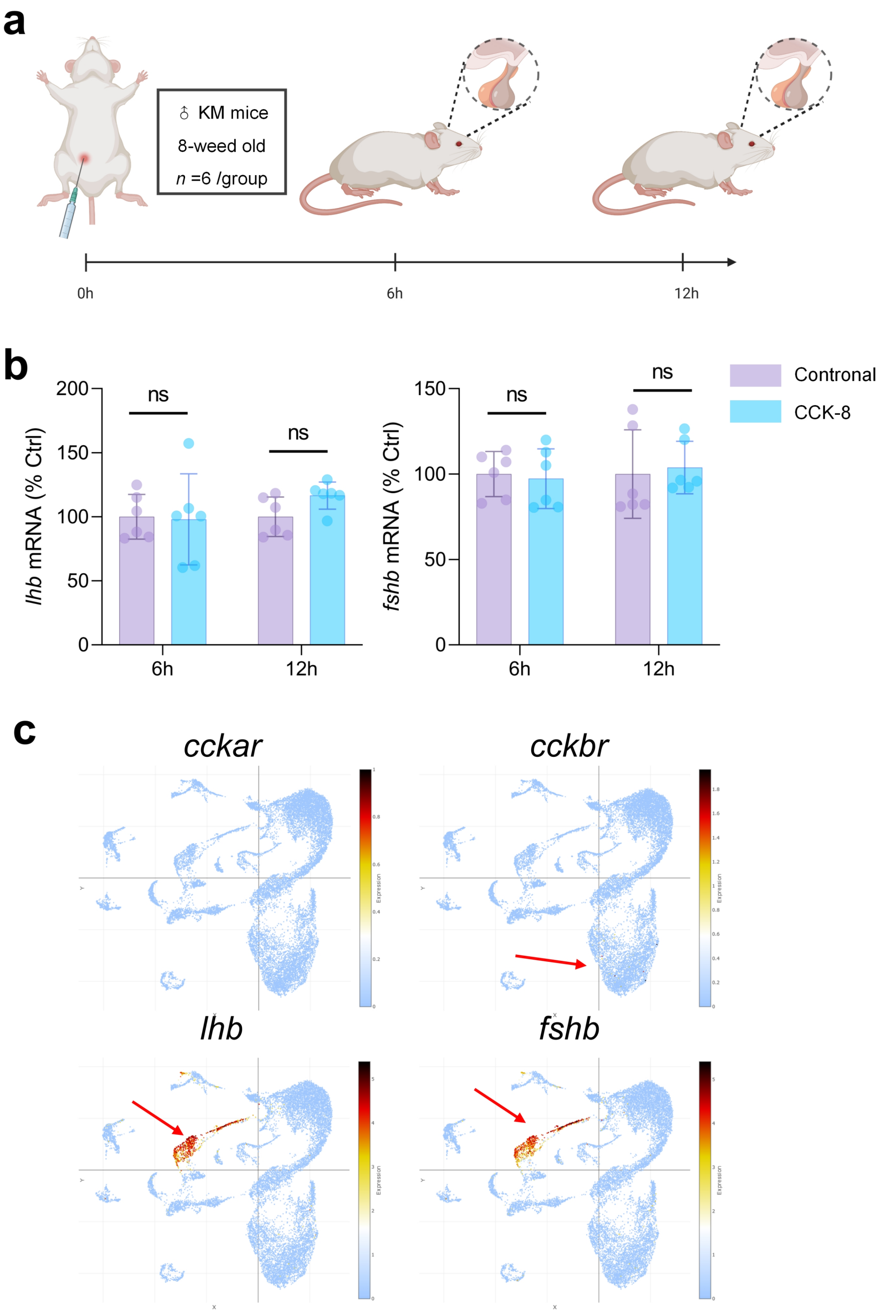

Supplementary Fig. 16 | Cholecystokinin-8 does not regulate pituitary *lhb* and *fshb* gene expression in male mice. a Schematic illustrating pituitary sampling at 6 and 12 hours after cholecystokinin-8 (CCK-8) or saline injection in male mice. Created in BioRender. Li, H. (2026) https://biorender.com/rfgfg10. **b** Pituitary *lhb* and *fshb* mRNA levels quantified by RT-qPCR 6 and 12 hours post-injection of CCK-8 or saline (*n* = 6 biologically independent male mice per group). Data are presented as mean ± s.e.m. Statistical significance was determined by two-sided unpaired Student's *t*-test. **c** UMAP visualization of *cckar*, *cckbr*, *lhb*, and *fshb* gene expression distribution in pituitary cells from 7-week-old adult male mice.

Supplementary Fig. 17

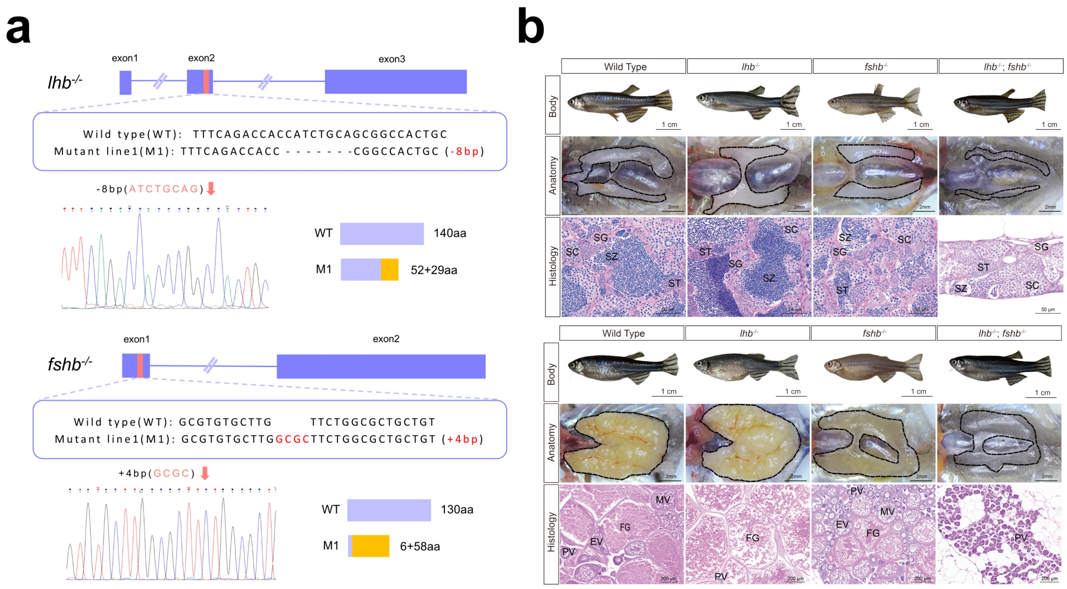

Supplementary Fig. 17 | Morphological and histological analysis of gonads in *lhb* and *fshb* knockout zebrafish models. a Schematic diagram showing the CRISPR/Cas9 targeting locations within the *lhb* and *fshb* genes. **b** Morphological and histological analysis of gonads in *lhb* and *fshb* single and double knockout zebrafish models compared to wild-type (WT) controls. Representative images illustrate the gross morphology and histological sections of the gonads. The developmental stages of oocytes and spermatogenesis are indicated by the following abbreviations: FG, fully mature oocytes; MV, mid-vitellogenic stage; EV, early vitellogenic stage; PV, pre-vitellogenic stage; PG, primary growth stage; SG, spermatogonia; SC, spermatocytes; ST, spermatids; SZ, mature sperm. Scale bars are indicated on the images. Representative images from five independent experiments with similar results are shown.

Supplementary Fig. 18

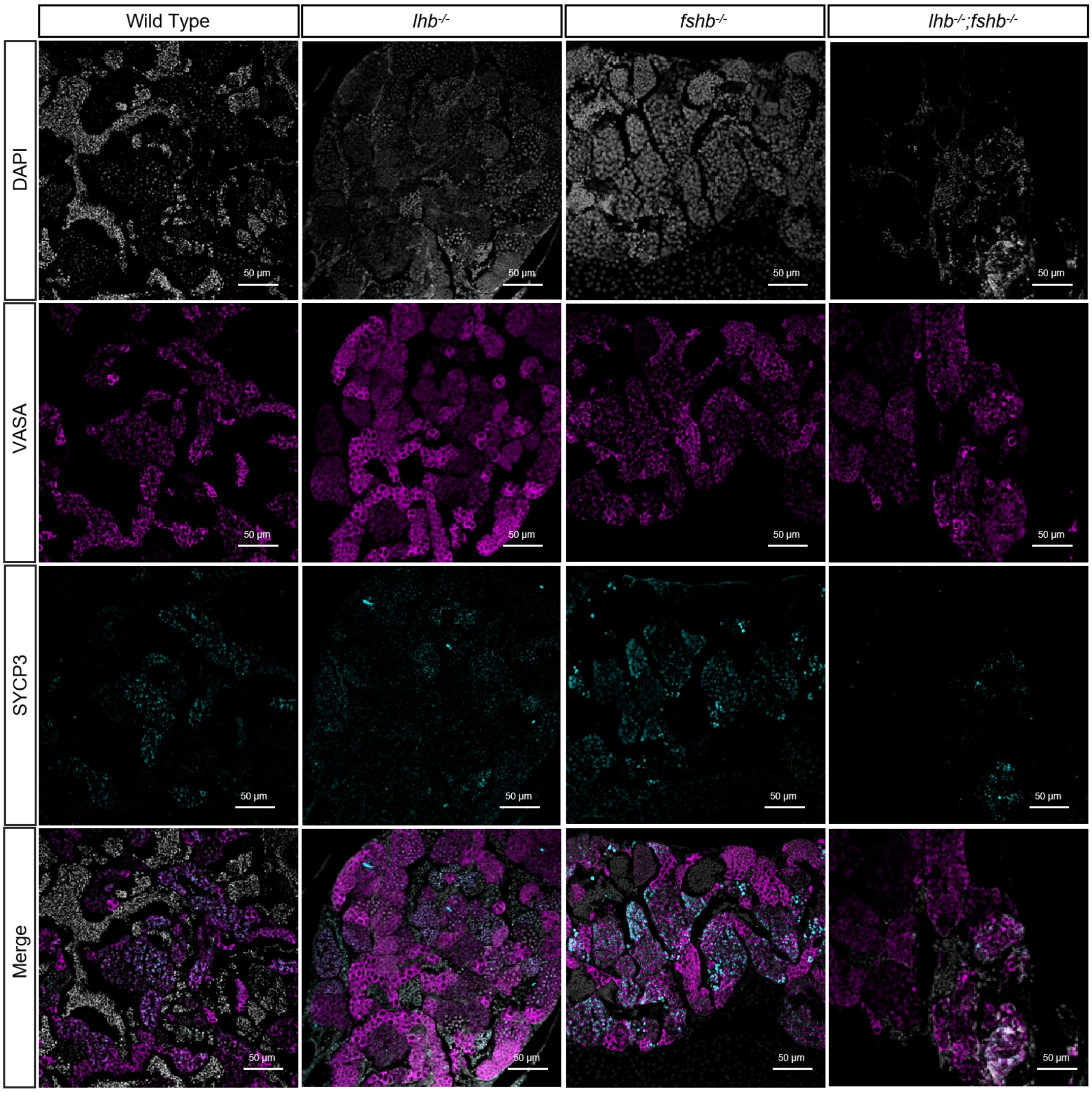

Supplementary Fig. 18 | Immunofluorescence co-expression analysis of VASA and SYCP3 proteins in the testes of *lhb* and *fshb* single and double knockout zebrafish models. Representative images from three independent experiments with similar results are shown.

Supplementary Fig. 19

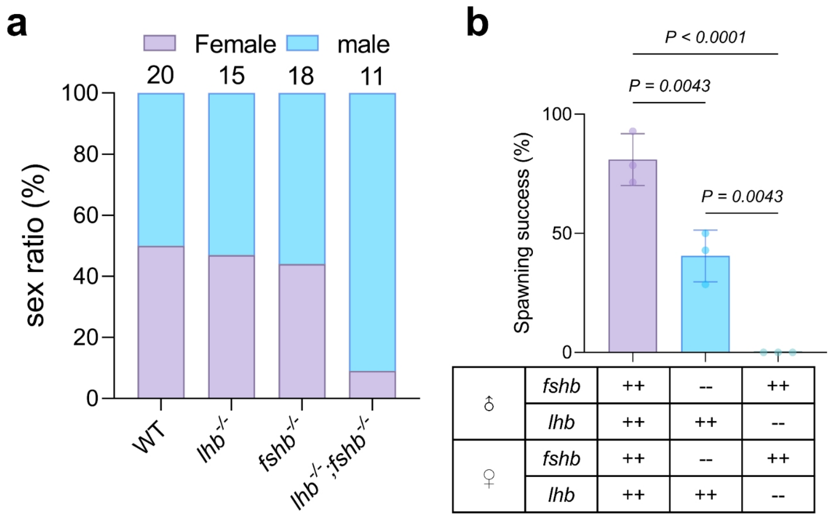

Supplementary Fig. 19 | Sex ratio and mating success in *lhb* and *fshb* knockout zebrafish models. a Analysis of sex ratios in *lhb* and *fshb* knockout zebrafish. The number of fish per group is indicated on the graph. **b** Comparison of spawning success rates (%) across different paired male and female genotypes. Data are presented as mean ± s.e.m. for *n* =12 independent mating pairs (experiment repeated in three sets). Statistical significance was determined by two-sided unpaired Student's *t*-test.

Supplementary Fig. 20

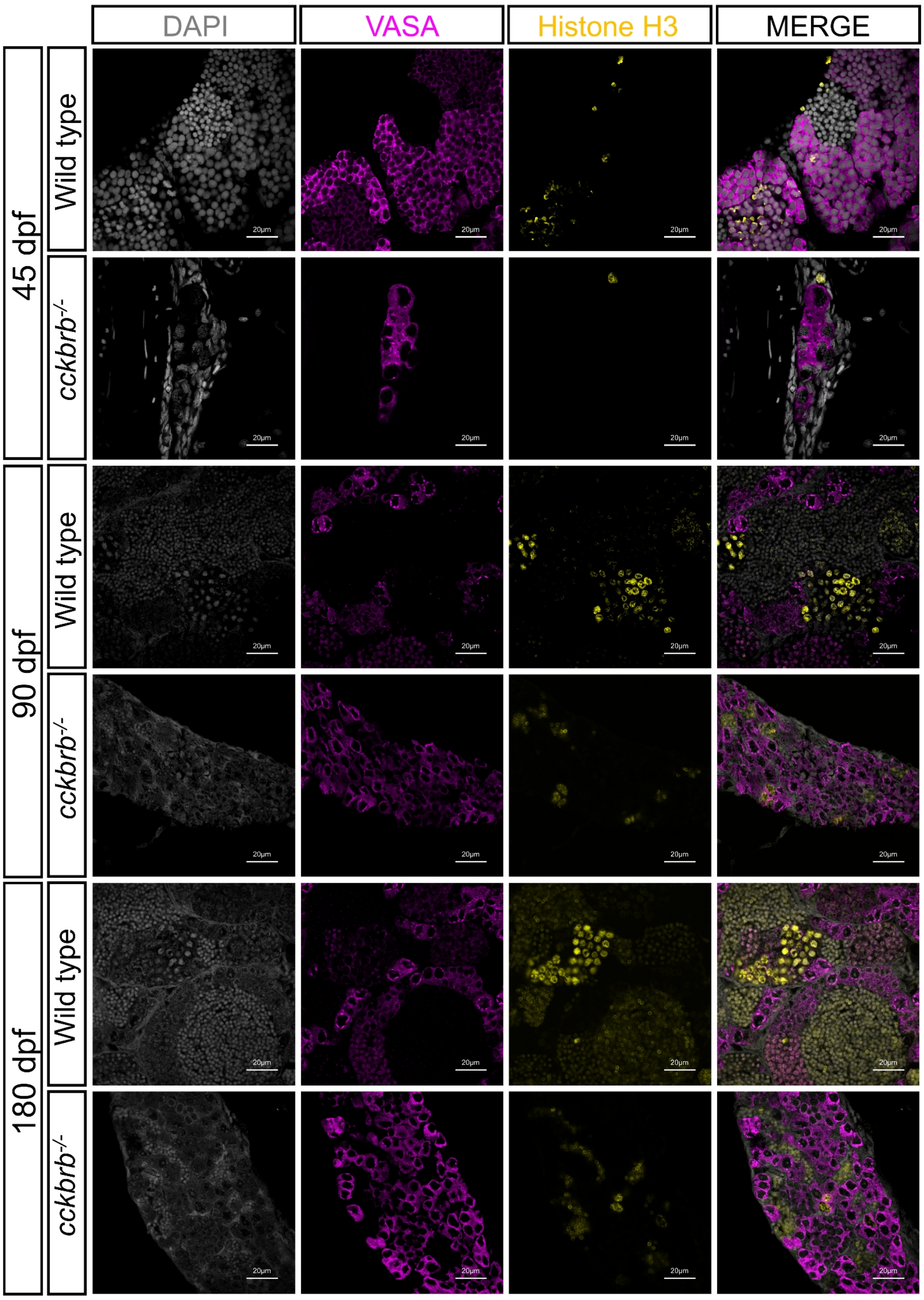

Supplementary Fig. 20 | Immunofluorescence analysis of germ cell proliferation in *cckbrb*$^{-/-}$ zebrafish at different developmental stages. The figure displays Immunofluorescence (IF) co-staining of gonadal sections from Wild-type (WT) and *cckbrb*$^{-/-}$ zebrafish at 45 dpf, 90 dpf, and 180 dpf. The sections were stained for VASA (a germ cell marker) and Histone H3 (H3) (a mitotic cell marker) to assess germ cell proliferation. Scale bars are indicated on the images. Representative images from three independent experiments with similar results are shown.

Supplementary Fig. 21

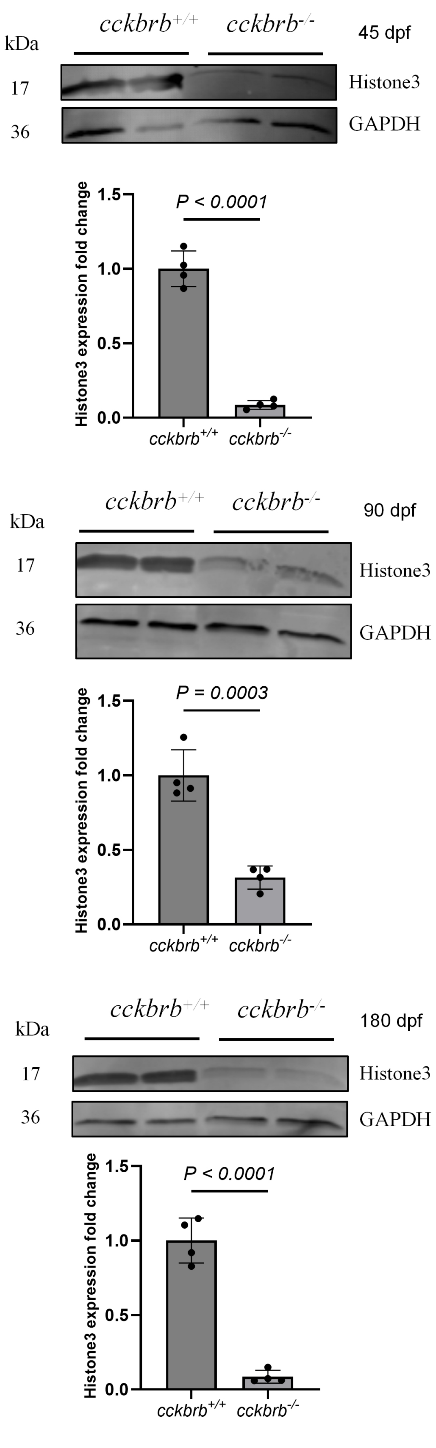

Supplementary Fig. 21 | Representative western blots showing the expression levels of Histone H3 in gonads from Wild-type (WT) and *cckbrb*$^{-/-}$ zebrafish at 45, 90, and 180 days post fertilization (dpf). GAPDH served as a loading control. Quantification of Histone H3 expression is shown below each blot. Data are presented as mean ± s.e.m. for n = 4 independent biological replicates. Statistical significance was determined by two-sided unpaired Student’s t-test. The experiment was repeated three times independently with similar results. Source data are provided as a Source Data file.

Supplementary Fig. 22

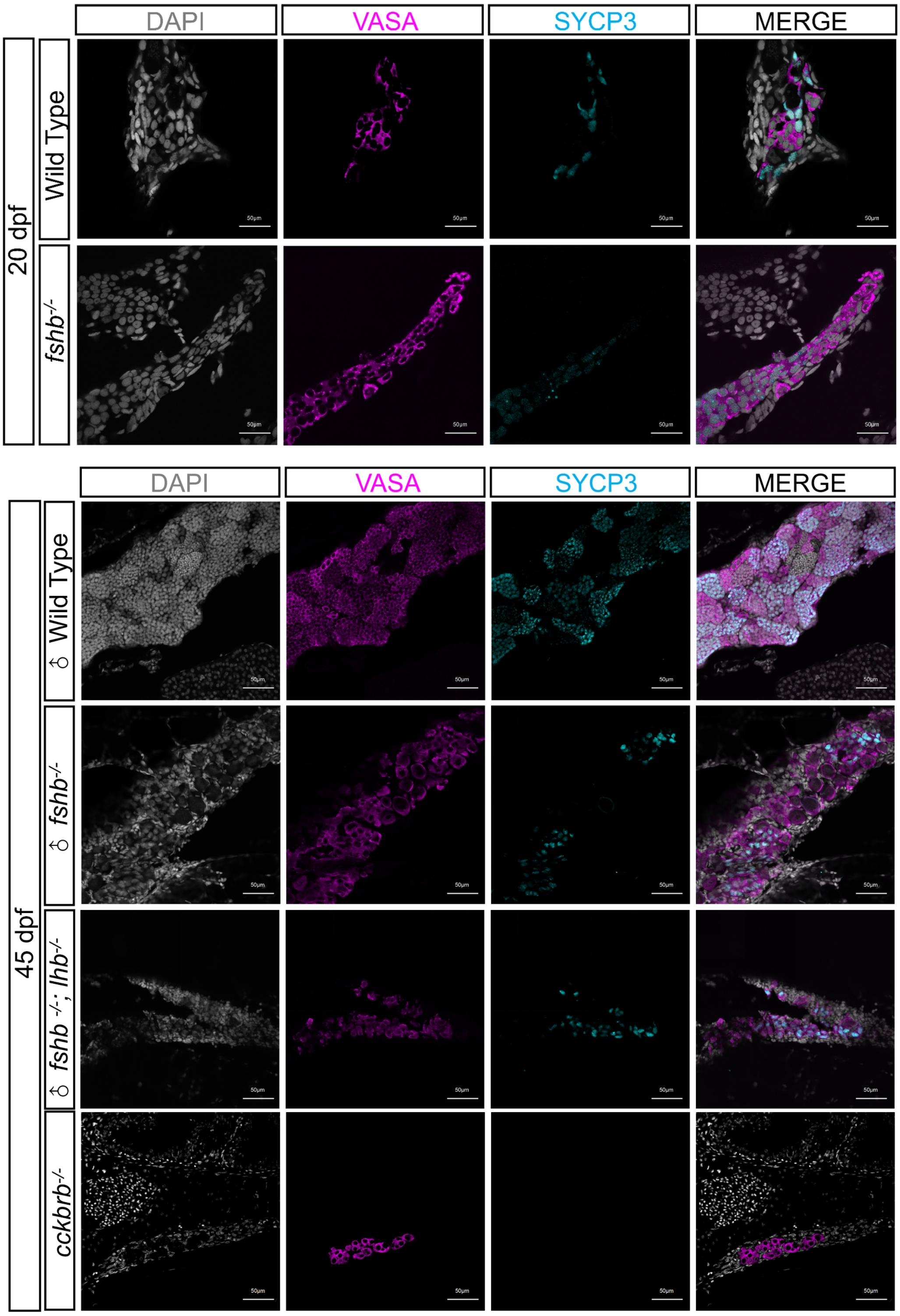

Supplementary Fig. 22 | Co-expression analysis of VASA and SYCP3 in gonads of WT, *fshb*$^{-/-}$, *lhb*$^{-/-}$;*fshb*$^{-/-}$, and *cckbrb*$^{-/-}$ zebrafish. Representative images from three independent experiments with similar results are shown.

Supplementary Fig. 23

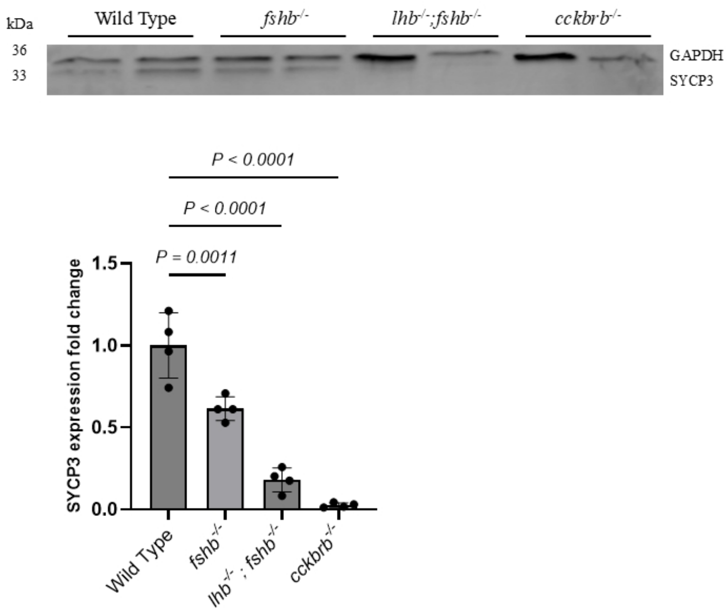

Supplementary Fig. 23 | Western blot analysis of the meiotic marker SYCP3 in WT, *fshb*$^{-/-}$, *lhb*$^{-/-}$;*fshb*$^{-/-}$, and *cckbrb*$^{-/-}$ zebrafish. Representative western blots showing SYCP3 protein expression in gonads from WT, *fshb*$^{-/-}$, *lhb*$^{-/-}$;*fshb*$^{-/-}$, and *cckbrb*$^{-/-}$ zebrafish at 45 days post fertilization (dpf). GAPDH served as a loading control. Quantification of SYCP3 expression is shown below the blot. Data are presented as mean ± s.e.m. for n = 4 independent biological replicates. Statistical significance was determined by one-way ANOVA followed by Dunnett's multiple-comparisons test. The experiment was repeated three times independently with similar results. Source data are provided as a Source Data file.

Supplementary Fig. 24

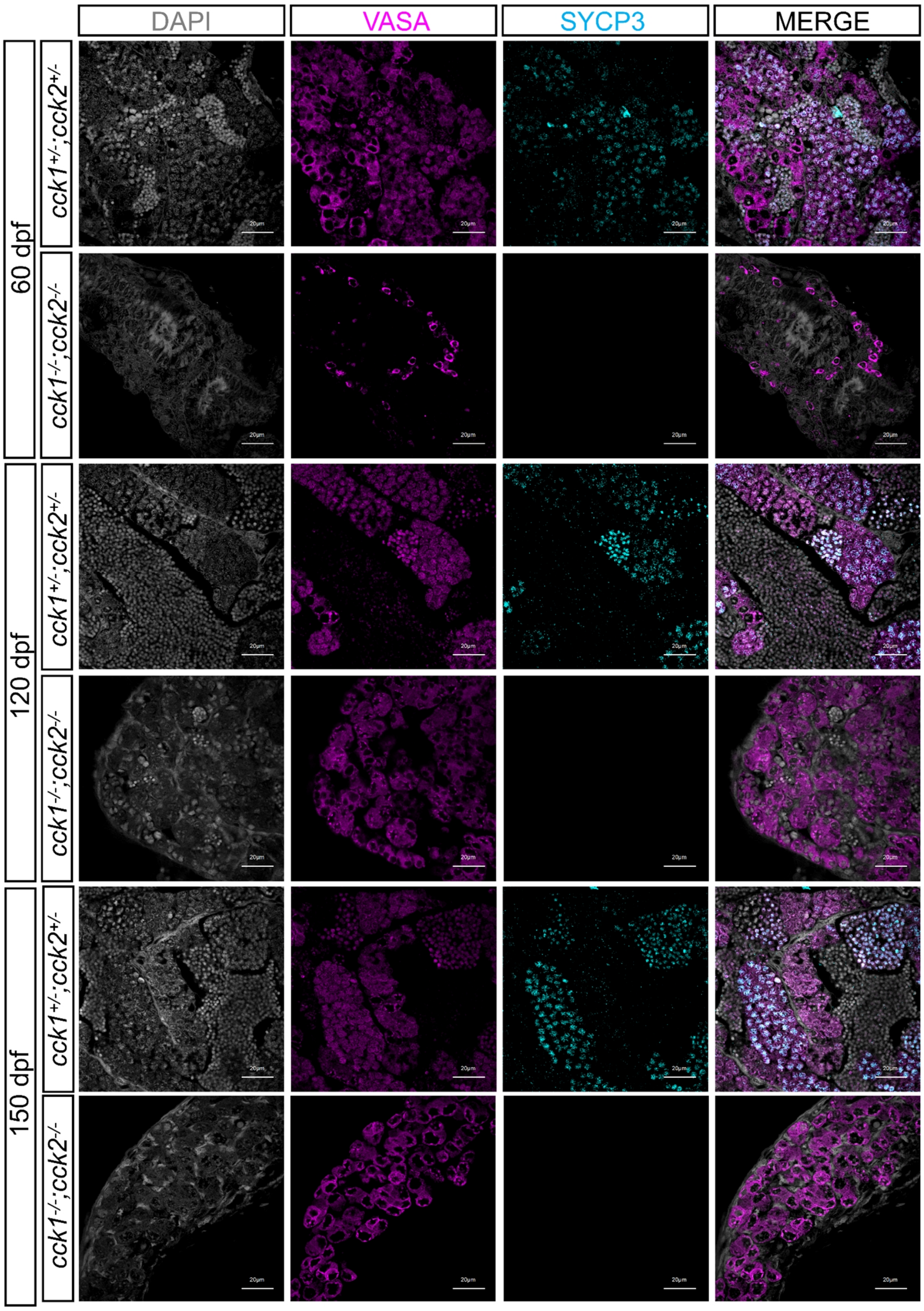

Supplementary Fig. 24 | Temporal analysis of meiosis in testicular tissue at 60, 120, and 150 dpf in WT and *cck1*$^{-/-}$;*cck2*$^{-/-}$ zebrafish using VASA/SYCP3 co-staining. Representative images from three independent experiments with similar results are shown.

Supplementary Fig. 25

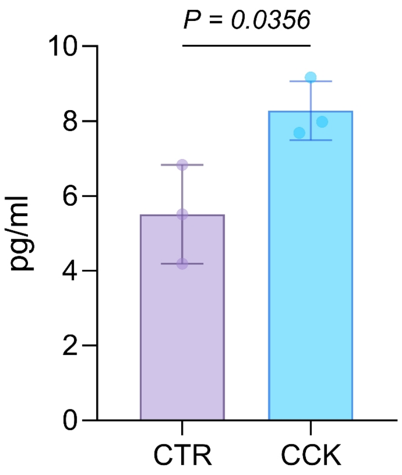

Supplementary Fig. 25 | Quantification of internal CCK concentration in larval zebrafish following waterborne immersion. Internal CCK concentration (pg/ml) was measured by ELISA in 48 hpf zebrafish larvae from Control (CTR) and CCK immersion groups after 1 hour treatment. Data are presented as mean ± s.e.m. for $n = 3$ independent biological replicates. Each replicate is a pooled sample of 20-30 individual larvae. Statistical significance was determined by two-sided unpaired Student's *t*-test.

Supplementary Fig. 26

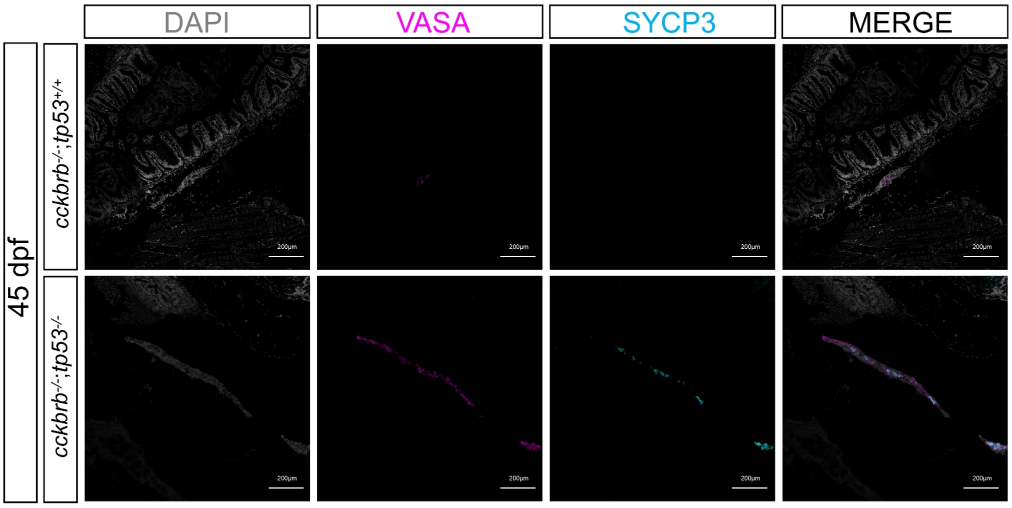

Supplementary Fig. 26 | Analysis of SYCP3 expression in *cckbrb$^{-/-}$;tp53$^{+/+}$* and *cckbrb$^{-/-}$;tp53$^{-/-}$* zebrafish gonads. The figure displays low-magnification Immunofluorescence (IF) staining of SYCP3 protein (a meiotic marker) in gonadal sections. The analysis compares *cckbrb$^{-/-}$;tp53$^{+/+}$*, and *cckbrb$^{-/-}$;tp53$^{-/-}$* zebrafish. Scale bars are indicated on the images. Representative images from three independent experiments with similar results are shown.

Supplementary Table 1 | EC_{50} Values for Zebrafish CCK Receptors

Receptor	CCK1 EC_{50} (nM)	CCK2 EC_{50} (nM)
CCKAR	-	68.4 ± 33.1
CCKBRA	240.8 ± 128.1	135.9 ± 108.6
CCKBRB	8.3 ± 5.7	4.9 ± 2.3

The values represent the effective concentration (EC_{50}) of zebrafish CCK1 and CCK2 ligands. Data are presented as the mean ± s.e.m. $n = 4$.

Supplementary Table 2 The primers used in the study

	Forward primer（5’-3’）	Reverse primer（5’-3’）
Genotyping		
CCK1-C	AGCAGTTTCACTCACTAATCCTC	TGCGGAGCCGACATTAGAC
CCK2-C	TGCTCTCCTCCTCAGGTCTC	TCAGTGTCGTGTGCGTCTC
CCKBRb-C	ACGGAGTAGACAGGCATCCA	TGGTATAAGCGCCACTGTCTT
FSHb-C	TTCACTTCGTAACCCCAGGC	AGCTGCACCTGCATTCTGAT
LHb-C	CATTCTCCAGGATGTTATTGGCTG	TAGCGCATGTGTATGTGACAGTG
RT-qPCR		
cck1	CGCTGGACTCTGTGTATG	ATCGGCTAATCGGATGGA
cck2	GCCGGAACTCCTCGATGAAC	CAGCCCACATAATCCCGGTC
gnrh3	CAGCACTGGTCATACGGTTGG	TTCAGCATCCACCTCATTCACT
lhb	GGTGTCTTCTTTCTCTTCTC	CGGGCTCTTGTAAACGGGAT
fshb	TTGTTCTGGCGCTGCTGTTGC	TTCTGGGTGTGCTGTGCCAT
cckar	ACTTCATGGGCACCTCTGTG	CCAGGTAGCAGCAATCACCT
cckrbra	TGGTGGATTGTCATCTTGCCAT	CTTCCAGGTGTTGGCAGAGT
cckrbrb	TAGCGAGGTGGAGAAGGCAT	CTCCGTCGTCGCTGCTAAT
sycp1	GCAGAGCTGAGTGAAAAACG	TGACACTGGTGTCTTCAGGA
sycp2	AGTTCTCAGAGGTTGGAGAAAGT	TCTCTTGGTAGGTGTGGAAGG
sycp3	GCTGAGCAAGAAAAGATCTGC	TTTTGGTGAGAACCTCCAG
rad21l1	CGCCGAGACATGTTTTATGCCC	TCAAACACGTGGGCTTTGGT
smc1b	AGAAACGCCAGCGCTCAG	GTCCAGAGTCAGAAGACGGC
syce2	CATACATCAGCAAAGGTGCCAG	CACCAAAAGGTTCCAGGGCT
terb1	GTGACTTTCACTTGGCGCTG	GCTGTATGTTTCACGCGGTC
terb2	ACTGCATGGTTCTCCGACAG	TCTGACGAGGTTGACATGCT
hsf5	GGCAGCTCAACCTCTACGG	TCAGCCTCTTCAGATGGACC
tesmin	CCCCATGACCAGCACAAGAT	CATGGGGTGAGTGGTGTTCA
lhb-mice	TCTGGCCGCAGAGAATGAGT	ACCATGCTAGGACAGTAGCC
fshb-mice	ACGAGACCGTAAGATTGCCT	GTGCTGTCGCTGTCACACTT
In Situ Hybridization		
cckbrb-FISH	AACAGATGCTGCGTAAGCCT	
Reference gene		
ef1a	AACTCTGCTGCTCTTGAAT	GCGTAACTGTGGATGTGA

Supplementary Table 3. Single-cell RNA sequencing and analysis.

**Single-cell RNA sequencing (scRNA-seq)**	Tissues were dissected into single-cell suspensions and sent to OE Biotech (Shanghai, China) for scRNA-seq. The 10X Genomics was used to generate single-cell Gel Beads-in-emulsion (GEMs). Immediately after GEM generation, the gel bead was dissolved, primers including an Illumina TruSeq Read 1 (read 1 sequencing primer), 16 nt 10x barcode, 12 nt unique molecular identifier (UMI), 30 nt poly(dT) sequence were released and mixed with cell lysate and a master mix containing reverse transcription (RT) reagents. Silane magnetic beads were used to purify first-strand cDNA from the post-GEM-RT reaction mixture, which contained residual biochemical reagents and primers. Barcoded full length cDNA was amplified by PCR to generate sufficient mass for library construction.
**Single-cell RNA analysis by Seurat**	Raw reads generated from high-throughput sequencing were sequenced in fastq format. The CellRanger software package (version. 5.0.0, 10x Genomics, California, USA) was used to perform data quality statistics on the original data and to quantify the high-throughput single-cell transcriptome against the reference genome to obtain quality control statistics such as the number of high-quality cells. The Seurat software package (version. 4.1.0) was then used for further quality control and processing based on the CellRanger preliminary quality control results. The principal component analysis (PCA) was performed using gene expression levels, and the results were visualised in two-dimensional space using tSNE. The FindAllMarkers function in Seurat was used to identify marker genes and to identify genes that are differentially up-regulated in each cell category compared to other cell groups. The marker genes were visualised using the VlnPlot and FeaturePlot functions.
