# Supplementary material for: Cholecystokinin coordinates gonadotropin-dependent and independent pathways to orchestrate zebrafish gonadal development
Source: Nat Commun. 2026 Apr 18;17:5392. doi: 10.1038/s41467-026-72039-x (PMC13275923; doi:10.1038/s41467-026-72039-x)
Supplement: Supplementary file 2 — Description of Additional Supplementary Files [file 41467_2026_72039_MOESM2_ESM.pdf]

Description of Additional Supplementary Files

File Name: Supplementary Movie 1
Description: Representative 2-min video clip showing the courtship behaviors of wild-type and *cck1*$^{-/-}$;*cck2*$^{-/-}$ male zebrafish during the 10-min observation period.

File Name: Supplementary Movie 2
Description: Representative 2-min video clip showing the courtship behaviors of wild-type and *cckbrb*$^{-/-}$ male zebrafish during the 10-min observation period.
