# Supplementary material for: Cholecystokinin coordinates gonadotropin-dependent and independent pathways to orchestrate zebrafish gonadal development
Source: Nat Commun. 2026 Apr 18;17:5392. doi: 10.1038/s41467-026-72039-x (PMC13275923; doi:10.1038/s41467-026-72039-x)
Supplement: Supplementary file 5 — Reporting Summary [file 41467_2026_72039_MOESM5_ESM.pdf]

nature portfolio

Corresponding author(s): Guangfu Hu

Last updated by author(s): Mar 24, 2026

Reporting Summary

Nature Portfolio wishes to improve the reproducibility of the work that we publish. This form provides structure for consistency and transparency in reporting. For further information on Nature Portfolio policies, see our Editorial Policies and the Editorial Policy Checklist.

Statistics

For all statistical analyses, confirm that the following items are present in the figure legend, table legend, main text, or Methods section.

n/a	Confirmed	
☐	☒	The exact sample size (*n*) for each experimental group/condition, given as a discrete number and unit of measurement
☐	☒	A statement on whether measurements were taken from distinct samples or whether the same sample was measured repeatedly
☐	☒	The statistical test(s) used AND whether they are one- or two-sided<br>*Only common tests should be described solely by name; describe more complex techniques in the Methods section.*
☐	☒	A description of all covariates tested
☐	☒	A description of any assumptions or corrections, such as tests of normality and adjustment for multiple comparisons
☐	☒	A full description of the statistical parameters including central tendency (e.g. means) or other basic estimates (e.g. regression coefficient) AND variation (e.g. standard deviation) or associated estimates of uncertainty (e.g. confidence intervals)
☒	☐	For null hypothesis testing, the test statistic (e.g. *F*, *t*, *r*) with confidence intervals, effect sizes, degrees of freedom and *P* value noted<br>*Give P values as exact values whenever suitable.*
☒	☐	For Bayesian analysis, information on the choice of priors and Markov chain Monte Carlo settings
☒	☐	For hierarchical and complex designs, identification of the appropriate level for tests and full reporting of outcomes
☒	☐	Estimates of effect sizes (e.g. Cohen's *d*, Pearson's *r*), indicating how they were calculated

Our web collection on statistics for biologists contains articles on many of the points above.

Software and code

Policy information about availability of computer code

Data collection	Data collection was performed using ABI 7500 SDS v1.5.1 for qRT-PCR measurements. No custom code was generated or used in this study.
Data analysis	Data analysis was performed using ImageJ v1.53 for image quantification, Tracker 6.0 for swimming trajectory analysis, SPSS Statistics 26.0 for statistical analyses, CellRanger v5.0.0 for preliminary scRNA-seq processing, and Seurat v4.1.0 for downstream single-cell RNA-seq analyses. No custom code was generated or used in this study.

For manuscripts utilizing custom algorithms or software that are central to the research but not yet described in published literature, software must be made available to editors and reviewers. We strongly encourage code deposition in a community repository (e.g. GitHub). See the Nature Portfolio guidelines for submitting code & software for further information.

Data

Policy information about availability of data

All manuscripts must include a data availability statement. This statement should provide the following information, where applicable:

- Accession codes, unique identifiers, or web links for publicly available datasets
- A description of any restrictions on data availability
- For clinical datasets or third party data, please ensure that the statement adheres to our policy

PRJNA1441806 [https://www.ncbi.nlm.nih.gov/bioproject/PRJNA1441806] (raw bulk RNA-seq data generated in this study). GSE296008 [https://www.ncbi.nlm.nih.gov/geo/query/acc.cgi?acc=GSE296008] (scRNA-seq data generated in this study). GSE295139 [https://www.ncbi.nlm.nih.gov/geo/query/acc.cgi?

acc=GSE295139] (scRNA-seq data generated in this study). GSE120410 [https://www.ncbi.nlm.nih.gov/geo/query/acc.cgi?acc=GSE120410] (published single-cell transcriptomic dataset analysed in this study). Other data supporting the findings of this study are available within the paper, its Supplementary Information, and the Source Data file. Source data are provided with this paper.

Research involving human participants, their data, or biological material

Policy information about studies with human participants or human data. See also policy information about sex, gender (identity/presentation), and sexual orientation and race, ethnicity and racism.

Reporting on sex and gender	*Use the terms sex (biological attribute) and gender (shaped by social and cultural circumstances) carefully in order to avoid confusing both terms. Indicate if findings apply to only one sex or gender; describe whether sex and gender were considered in study design; whether sex and/or gender was determined based on self-reporting or assigned and methods used. Provide in the source data disaggregated sex and gender data, where this information has been collected, and if consent has been obtained for sharing of individual-level data; provide overall numbers in this Reporting Summary. Please state if this information has not been collected. Report sex- and gender-based analyses where performed, justify reasons for lack of sex- and gender-based analysis.*
Reporting on race, ethnicity, or other socially relevant groupings	*Please specify the socially constructed or socially relevant categorization variable(s) used in your manuscript and explain why they were used. Please note that such variables should not be used as proxies for other socially constructed/relevant variables (for example, race or ethnicity should not be used as a proxy for socioeconomic status). Provide clear definitions of the relevant terms used, how they were provided (by the participants/respondents, the researchers, or third parties), and the method(s) used to classify people into the different categories (e.g. self-report, census or administrative data, social media data, etc.) Please provide details about how you controlled for confounding variables in your analyses.*
Population characteristics	*Describe the covariate-relevant population characteristics of the human research participants (e.g. age, genotypic information, past and current diagnosis and treatment categories). If you filled out the behavioural & social sciences study design questions and have nothing to add here, write "See above."*
Recruitment	*Describe how participants were recruited. Outline any potential self-selection bias or other biases that may be present and how these are likely to impact results.*
Ethics oversight	*Identify the organization(s) that approved the study protocol.*

Note that full information on the approval of the study protocol must also be provided in the manuscript.

Field-specific reporting

Please select the one below that is the best fit for your research. If you are not sure, read the appropriate sections before making your selection.

[x] Life sciences [ ] Behavioural & social sciences [ ] Ecological, evolutionary & environmental sciences

For a reference copy of the document with all sections, see nature.com/documents/nr-reporting-summary-flat.pdf

Life sciences study design

All studies must disclose on these points even when the disclosure is negative.

Sample size	No statistical methods were used to predetermine sample size. Sample sizes were chosen based on common practice in the field, previous experience with similar zebrafish experiments, and sample availability.
Data exclusions	No data were excluded from the analyses.
Replication	Experiments were independently repeated where applicable, and the number of independent repeats is stated in the figure legends or Methods section.
Randomization	Animals/samples/cultures were randomly assigned to experimental and control groups upon enrollment to minimize bias.
Blinding	Investigators were blinded to group allocation during both data collection and outcome assessment/statistical analysis to prevent observer bias.

Reporting for specific materials, systems and methods

We require information from authors about some types of materials, experimental systems and methods used in many studies. Here, indicate whether each material, system or method listed is relevant to your study. If you are not sure if a list item applies to your research, read the appropriate section before selecting a response.

Materials & experimental systems

n/a	Involved in the study
☐	☒ Antibodies
☐	☒ Eukaryotic cell lines
☒	☐ Palaeontology and archaeology
☐	☒ Animals and other organisms
☒	☐ Clinical data
☒	☐ Dual use research of concern
☒	☐ Plants

Methods

n/a	Involved in the study
☒	☐ ChIP-seq
☒	☐ Flow cytometry
☒	☐ MRI-based neuroimaging

Antibodies

Antibodies used	The following antibodies were used in this study: anti-Piwil1 (CZPA2, China Zebrafish Resource Center (CZRC), 1:500), Alexa Fluor 594 goat anti-rabbit IgG (Yeasen, 1:200), anti-CCK (C2581, Merck, 1:1000), anti-Vasa (2008, Dia-An, 1:800), anti-SYCP3 (CZPA3, CZRC, 1:1000), anti-SOX9a (ab209820, Abcam, 1:1000), anti-Histone H3 (ab183626, Abcam, 1:1000), and anti-PCNA (ab29, Abcam, 1:1000). Custom antibodies included anti-GnRH3 (Dia-An, Wuhan, 1:500), anti-LH (Dia-An, Wuhan, 1:500), anti-FSH (Dia-An, Wuhan, 1:500), and a GnRH3-neutralizing antibody (Dia-An, Wuhan, 1:500).
Validation	The specificity of the anti-GnRH3, anti-LH, and anti-FSH antibodies was empirically validated by Western blot or antigen pre-absorption tests, as described in the Methods. Commercial antibodies were used according to the manufacturers' recommendations and the applications described in the manuscript.

Eukaryotic cell lines

Policy information about cell lines and Sex and Gender in Research

Cell line source(s)	HEK-293T cells were obtained from Warner Bio (Wuhan, China; Catalog No. WN-10257).
Authentication	The HEK-293T cell line used in this study was authenticated by short tandem repeat (STR) profiling.
Mycoplasma contamination	The HEK-293T cells tested negative for mycoplasma contamination.
Commonly misidentified lines (See ICLAC register)	No commonly misidentified cell lines from the ICLAC register were used.

Animals and other research organisms

Policy information about studies involving animals; ARRIVE guidelines recommended for reporting animal research, and Sex and Gender in Research

Laboratory animals	Laboratory animals used in this study included zebrafish (Danio rerio, AB strain) and the tp53 mutant zebrafish line (Catalog ID: CZ267), obtained from the China Zebrafish Resource Center (CZRC), and KM mice (male, 8-week-old, 35–45 g) obtained from the Laboratory Animal Center of Huazhong Agricultural University.
Wild animals	This study did not involve wild animals.
Reporting on sex	For experiments performed at early developmental stages, sex could not be determined reliably and was therefore not considered in the study design or analysis. For adult zebrafish and mouse experiments, sex was determined and is indicated where applicable in the corresponding Methods sections and figure legends. Where sex information was collected, data were reported accordingly and disaggregated by sex where appropriate.
Field-collected samples	This study did not involve field-collected samples.
Ethics oversight	All animal procedures were approved by the Institutional Animal Care and Use Committee of Huazhong Agricultural University (IACUC No. HZAUFI-2023-028) and complied with national ethical guidelines.

Note that full information on the approval of the study protocol must also be provided in the manuscript.

Plants

Seed stocks	*Report on the source of all seed stocks or other plant material used. If applicable, state the seed stock centre and catalogue number. If plant specimens were collected from the field, describe the collection location, date and sampling procedures.*
Novel plant genotypes	*Describe the methods by which all novel plant genotypes were produced. This includes those generated by transgenic approaches, gene editing, chemical/radiation-based mutagenesis and hybridization. For transgenic lines, describe the transformation method, the number of independent lines analyzed and the generation upon which experiments were performed. For gene-edited lines, describe the editor used, the endogenous sequence targeted for editing, the targeting guide RNA sequence (if applicable) and how the editor was applied.*
Authentication	*Describe any authentication procedures for each seed stock used or novel genotype generated. Describe any experiments used to assess the effect of a mutation and, where applicable, how potential secondary effects (e.g. second site T-DNA insertions, mosiacism, off-target gene editing) were examined.*
