# Supplementary material for: Cholecystokinin coordinates gonadotropin-dependent and independent pathways to orchestrate zebrafish gonadal development
Source: Nat Commun. 2026 Apr 18;17:5392. doi: 10.1038/s41467-026-72039-x (PMC13275923; doi:10.1038/s41467-026-72039-x)
Supplement: Supplementary file 6 — Transparent Peer Review file [file 41467_2026_72039_MOESM6_ESM.pdf]

nature portfolio

Peer Review File

Cholecystokinin coordinates gonadotropin-dependent and independent pathways to orchestrate zebrafish gonadal development

Corresponding Author: Professor Guangfu Hu

Parts of this Peer Review File have been redacted as indicated to remove third-party material.

This file contains all reviewer reports in order by version, followed by all author rebuttals in order by version.

Version 0:

Reviewer comments:

Reviewer #1

(Remarks to the Author)
Using a combination of gene editing (cck1/cck2 double knockout and cckbrb receptor knockout), comparative analysis with gonadotropin knockouts (fshb, lhb, and double knockouts), single-cell RNA sequencing, cell culture experiments, and rescue assays, this study investigated the role of cholecystokinin (CCK) in zebrafish gonadal development. In addition to confirming recent findings that CCK acts as a follicle-stimulating hormone-releasing hormone (FSH-RH) in teleosts, the authors aimed to demonstrate additional roles of CCK in zebrafish reproduction, both gonadotropin-dependent and independent. The major conclusion of the study is that CCK is not merely an FSH-RH, but also a LH-RH; and it not only regulates gonadotropins, but functions as a master regulator that orchestrates gonadal development. The study is overall well planned and performed with substantial amount of data supporting the conclusions. My questions are organized around these two major aspects of proposed CCK functions.
CCK as FSH-RH and LH-RH:
1. The finding that CCK functions as an FSH-releasing hormone in zebrafish is not novel, as this has been well established in recent studies in both zebrafish (Hollander-Cohen et al. 2024) and medaka (Uehara et al. 2024). This study essentially corroborates these previous results; however, the manuscript does not provide sufficient comparison or discussion of these earlier investigations.
2. The manuscript provided evidence that CCK also functions as an LH-releasing hormone in addition to its role in stimulating FSH. This observation agrees well with the previous report by Hollander-Cohen et al. in zebrafish that LH secretion was shut down in CCK receptor knockout. This study went further by proposing two mechanisms underlying such regulation: direct regulation at pituitary level and indirect regulation via GnRH3 in the hypothalamus. However, further evidence is needed to validate this regulation as commented below.
3. L153: direct pituitary actions: Is there any evidence for expression of CCK receptor(s) in the LH cells? The single cell expression data did not provide such information. The previous study by Hollander-Cohen et al. reported that zebrafish pituitary only expresses cckbra, not cckbrb, in contrast to the present study. This issue was not touched at all in the manuscript. In medaka, knockout of CCK signaling also resulted in reduced expression of lhb; however, this reduction was proven to be due to the secondary effects of steroid deficiency, not CCK.
4. L157: Any evidence for CCK acting via its receptor cckbrb in regulating LH? A simple reduction of lhb expression in the mutants does not sufficiently support its signalling mechanisms in LH cells. The authors do provide data showing CCK stimulation of lhb expression in vitro; however, this should be interpreted with caution because it has been reported that pituitary hormones may regulate each other via paracrine pathways.
5. The paper proposes that CCK may indirectly control LH biosynthesis via hypothalamic GnRH3. However, the importance of GnRH system (both GnRH2 and 3) in controlling pituitary gonadotropins has been rejected by recent genetic studies. Given this, the functional importance and physiological relevance of CCK regulation of LH via GnRH3 is questionable.
6. L167: This statement is not supported by strong evidence. The best way to demonstrate such mechanism is to perform the analysis in gnrh3 mutant.
7. L172: ScRNA-seq analysis revealed a considerable subset of pituitary cells expressing both fshb and lhb. This finding should be interpreted with caution. Firstly, the data presented in Figure 1a do not demonstrate co-localization of LH and FSH at the protein level. Secondly, multiple studies in fish, including zebrafish, have established that FSH and LH are

synthesized in two distinct populations of pituitary cells, in contrast to the co-expression observed in mammals. Therefore, the actual identity of cells co-expressing lhb and fshb requires further validation. Until such confirmation is available, it is premature to use evidence of cckbrb expression in these cells to support the argument for direct CCK regulation of LH. Moreover, there is no current evidence indicating co-expression of cckbrb specifically within lhb-expressing cells. The previous study by Hollander-Cohen et al. showed little or no expression of cckbra in the LH cells and no expression of cckbrb in the pituitary, which disagrees with the finding of the present study. The authors should carefully and thoroughly address this issue.

8. L178-180: Concluding that CCK promotes gonadal maturation and ovulation through its regulation of LH (either directly or indirectly) is premature, as this inference is based solely on their common ability to increase ovulation rates following injection. It is important to note that numerous hormones and factors have demonstrated similar effects, and we cannot conclude that they mediate actions of each other.
9. L344-345: To demonstrate that CCK promotes gonadal development through FSH, the best way would be using fshb mutant.
10. L369: This statement “GnRH3 is the primary factor inducing LH expression in zebrafish” may not be true as recent genetic studies have rejected the involvement of GnRH system in controlling gonadotropin biosynthesis.
11. L375-377: The involvement of GnRH3 in CCK actions should be tested in gnrh3 mutant.
12. The existence and mechanism of CCK-FSH and GnRH-LH axes has been reported recently in both zebrafish and medaka. However, there is a complete lack of comparative discussions about these earlier studies, especially the study in medaka story, in the present manuscript.

Gonadotropin-independent regulation of germline and gonadal development:

13. It is concluded in the manuscript that CCK promotes PGC proliferation and facilitate meiotic division, independently of gonadotropins. However, the study did not provide any information about where the CCK molecule comes from; in particular, the results from the present study clearly show that CCK genes are expressed in multiple organs including the gonads. The working model indicates that CCK comes from the hypothalamus as a neuroendocrine hormone (Figure 6); however, this needs to be validated by evidence.
14. To demonstrate that CCK can directly promote PGC proliferation, the authors need to provide two pieces of critical evidence: first, the expression of CCK receptor(s) in PGCs, and second, in vitro effects of CCK on cultured PGCs. The second one may be technically challenging, but it is worth trying. As a neuroendocrine hormone, CCK may indirectly influence PGC proliferation and migration via other endocrine and paracrine pathways.
15. L382-383: There is no evidence for roles of somatic cell integrity in controlling meiosis.

Other questions:

16. The authors used commercially available antibody to stain for CCK in the hypothalamus. Was the antibody specificity validated by Western blotting?
17. L108-110: It is stated that CCKBRb exhibited the highest binding affinity for CCK1/2. To evaluate and compare ligand-receptor affinity, one would need to construct a full dose response curve for ED50, which is the critical parameter for comparing binding affinity. However, most dose response curves presented (Fig. 2a) are not complete, making it inappropriate to make conclusions regarding affinity.
18. L118-119: replace “-“ with commas.
19. L141-142 and L148: Statistical analysis is needed to make the conclusion “marked reduction”, and “significantly promoted”.
20. L177: What LH was used to induce ovulation? This information is important because it is known that heterologous hormones especially those from mammals may have different receptor specificity and potency in fish species.
21. Figure 4b: the label “fshrb-/-” should be “fshb-/-”.
22. L273: “donadotropin” should be “gonadotropin”.
23. L274: In Figure 1, although the mutant males show deficient testis development, spermatocytes are obvious, indicating meiotic activity. How can the expression of cycp3 be completely missing?
24. L279: What does “permanent block” mean? If it indicates no meiotic activity, why are spermatocytes present in the mutant testis (Figure 1)? Without meiosis, gonads should be undifferentiated, then how is sex determined?
25. L286: Administering CCK through incubation is an interesting method. Any information about how efficiently the peptide is absorbed and which pathway(s) it may follow?
26. L409: The manuscript should include comprehensive details about the fshb and lhb mutants generated and utilized in this study. Given that fshb and lhb knockout models have also been reported by several other laboratories, such information would be helpful for comparative analysis, particularly when discrepancies in phenotypes are observed.
27. L430-431: How was total number of follicles counted for each female?
28. L434: How was area occupied by spermatogonia quantified as they are often scattered individually across testis sections?
29. L496: Embryos cannot be fertilized.
30. L502: Feed supplementation is an interesting approach for peptide delivery. However, this should be validated for the following: (1) doses used; (2) efficiency of absorption; and (3) serum or tissue concentrations.
31. The manuscript is very well written, clear and easy to follow. It appears that the authors used AI tools to assist in writing, which is perfectly fine and demonstrate the value of AI technology. However, the authors should disclose the use and indicate which tools were used. Also, the authors should try to humanize the final version to remove signs of AI writing, including the words commonly over-used by AI tools, such as Concurrently, Crucially, Notably, etc.

Reviewer #2

(Remarks to the Author)

Summary or Overall assessment

The authors present a comprehensive genetic study demonstrating that the CCK signaling pathway serves as a distinct

regulator of FSH and LH expression in teleosts, independent of GnRH—a finding of notable academic value that challenges classical models. To my knowledge, this is the third independent group to validate this concept using a fish model, following studies published in Nature Communications (Shun Kenny Uehara et al., 2024) and eLife (Lian Hollander-Cohen et al., 2024). The consistency of the present findings with prior work further enhances their credibility. Through detailed phenotypic analyses, the authors outline multiple roles of CCK across key stages of gametogenesis, including germ cell proliferation, meiotic initiation, somatic cell survival, sex determination, and the regulation of both FSH and LH. While phenotypic descriptions are thorough, mechanistic insights remain limited, which somewhat constrains the study's depth and broader impact. Nonetheless, given the strength of the dataset and the importance of the topic, the manuscript holds significant potential if mechanistic aspects are further developed. Below are my specific comments on the manuscript.

Major Concerns：
1.In Figure 1c, please indicate the age of the fish used and specify the experimental groups represented by the different colored bars in the quantification plot. While the data can be interpreted by an expert reader, it is essential to include a clear and complete legend to ensure rigorous and reproducible reporting. More broadly, figure legends involving animal experiments should report the animal's age, sample size, data format (e.g., mean ± s.e.m.), and statistical test used to ensure transparency and reproducibility. In addition, this issue is not limited to Figure 1 (Such as Fig. 2 et al). Throughout the manuscript, the age of animals used in all experiments—including histological analyses, immunofluorescence, and other animal-based assays—should be clearly stated.
2.The authors report that CCK regulates both FSH and LH, which contrasts with prior studies (Uehara et al., Nature Communications, 2024; Hollander-Cohen et al., eLife, 2024) that concluded CCK specifically regulates FSH, not LH. This discrepancy should be addressed in the discussion with direct comparison and mechanistic context. If confirmed, dual regulation of FSH and LH by CCK would be a significant advance. However, current evidence for LH regulation is preliminary—mainly based on spatial co-expression and indirect induction. Stronger functional validation or clearer discussion of the limitations is needed, especially as this may represent the study's most novel contribution.
3.Line 215-216: The manuscript compares the phenotypes of fshb-/-, lh-/-, and double mutants with those of cck-deficient zebrafish to support the gonadotropin-independent function of CCK. Please clarify whether these lhb/fshb mutants were newly generated in this study. If so, are their phenotypes consistent with previous reports? Additional details on their construction, genotyping, and phenotypic validation would strengthen the interpretation.
4.Fig 4: The authors assess PGC proliferation at 14 dpf using VASA and H3P staining and report a marked reduction in cckbrb mutants. However, have the authors considered the possibility of developmental delay in mutants, which may confound the assessment of proliferation at a fixed time point? Including a comparison of general developmental timing or discussing this potential bias would be helpful.
5.The authors attribute the all-male phenotype in CCK pathway mutants to disrupted PGC migration, proliferation, meiosis, and somatic cell survival. However, this remains correlative. In zebrafish, PGC loss may be a consequence—not a cause—of male development, and the possibility that CCK directly affects sex determination cannot be excluded. More critically, this model is inconsistent with findings in medaka: cck2rb knockout medaka still develop females (Uehara et al., Nature Communications, 2024). If CCK directly reduced PGCs, a similar male bias would be expected, which was not observed. This suggests that CCK may not directly control PGC number. The authors should interpret this mechanism more cautiously and consider alternative explanations. Additional molecular evidence demonstrating how CCK directly regulates PGC number—or PGC rescue experiments—would help clarify the causality and strengthen the conclusions.
6.The authors propose a central role for CCK in integrating metabolic and reproductive regulation in zebrafish. However, the potential species specificity, crosstalk with other metabolic regulators (e.g., NPY, ghrelin), and indirect effects through altered energy status remain underexplored. The discussion would benefit from acknowledging these limitations and outlining future directions, such as investigating feedback mechanisms or inter-organ communication pathways.

Minor Comments：
7.Line 114-116 (Fig 2d): The authors show that male double mutants exhibit normal secondary sexual characteristics, an important observation suggesting intact androgen signaling. This finding effectively rules out androgen deficiency as a cause of reproductive failure. Given that fish reproductive behavior is jointly regulated by LH and androgens, this result provides a critical foundation for interpreting subsequent behavioral and hormonal defects. However, the manuscript neither states the rationale behind this experiment nor discusses its biological relevance. As a result, the data are presented in isolation, making it difficult for readers—especially those outside the field—to appreciate its relevance. I recommend that the authors provide background context, clarify the intent of the experiment, and discuss its implications within the framework of sex hormone signaling and reproductive behavior.
8.Line 199: Please note that there should be a space between numerical values and units (e.g., "5 dpf" instead of "5dpf"). This formatting issue appears multiple times throughout the manuscript, such as in Line 199. A thorough check and correction are recommended to ensure consistency and adherence to journal style.
9.Fig. 3: Again, for panels j-m in, each should include its own legend indicating the group represented by each bar, even if the groups are the same. Currently, only panel j provides this information. Similar omissions are found elsewhere in the manuscript. Please ensure all figure panels are self-contained, with clear labeling of group identities, to allow interpretation without cross-referencing.
10.Fig. 3: Although the manuscript does not explicitly define "N" and "n", it appears that "n" refers to the number of biological replicates per experiment and "N" denotes the number of independent experimental repeats. However, there seems to be inconsistency in this usage. For instance, in Figure 3b, "N = 3, n = 4" is stated, yet only three data points are shown. In contrast, Figure 3l uses the same notation ("N = 3, n = 4"), but displays four data points. This discrepancy suggests inconsistent interpretation or presentation. Please clarify and, if necessary, correct the description to ensure accuracy and consistency.
11.Fig 3i: The labels "40X" and "100X" refer to objective magnifications and are not typically included directly in figure panels. Relative image size should instead be indicated using clearly visible scale bars. In the current manuscript, the scale

bars in several histological and immunofluorescence images (e.g., H&E and IF) are too small to be easily read. Please enlarge the scale bars to a legible size and ensure they are clearly visible across all relevant figures.
12.Immunofluorescence (IF) results should be quantified and supported with statistical analysis, particularly in figures such as Fig. S12, S13, and 4c. Quantification would enhance the rigor and interpretability of the data.
13.Line 235 and 472: The abbreviation "hpf" (hours post-fertilization) is defined more than once in the manuscript. Please retain the definition only at its first occurrence and use the abbreviation consistently thereafter to avoid redundancy.
14.Line 442-451: The study reports impaired mating behavior in cck mutants but does not clarify whether their general locomotor activity is intact. It would strengthen the interpretation to confirm that reduced mating behavior is not due to non-specific motor deficits.

Reviewer #3

(Remarks to the Author)
In this manuscript, the authors provide a detailed analysis of the effects of knocking out the CCK system on reproductive functions in zebrafish. While Hollander-Cohen previously performed knockouts in zebrafish, their study focused only on CCK receptors. In contrast, this manuscript includes ligand knockouts and thus provides a more detailed analysis of the effects on FSH.
In addition, the manuscript proposes a completely new perspective in which CCK regulates reproduction via gonadotropin-independent pathways. However, this analysis is limited to knockouts and single-cell RNA-seq expression profiling, with only minimal IHC, and does not investigate which CCK neurons act on which cell expressing CCK receptor. As such, the conclusion that hypothalamic CCK directly affects the gonads, as depicted in the schematic, is not supported by the evidence.
Furthermore, the authors' claim that CCK regulates LH contradicts previously published findings, but the experimental evidence provided in this manuscript is insufficient to support this assertion.
In conclusion, the reviewer considers that the manuscript lacks sufficient evidence to support many of its claims. Specifically, the main story relies on whole-body knockouts, which could have multiple indirect effects, and co-expression results from single-cell RNA-seq, which are not definitive. Without more detailed morphological analyses or in vitro physiological experiments, these conclusions cannot be drawn. To make definitive claims from single-cell RNA-seq, reproducibility should be confirmed using more conclusive methods such as double in situ hybridization. Single-cell RNA-seq does not necessarily reflect in vivo expression accurately, and using it alone to support conclusions risks error.

Technical issues:
• All RNA co-localization is discussed based solely on single-cell RNA-seq data. This can be misleading, as dissociation alters expression profiles and may not reflect physiological conditions. For example, in Figure 3a, histological data show distinct localization of LH and FSH cells, but single-cell RNA-seq suggests substantial co-localization. As reported in medaka (Fontaine et al., 2020, J. Endocrinol.), dissociation changes lh and fsh expression profiles. Thus, double in situ hybridization or other methods are needed to confirm these results. The cells labeled as "progenitors" in Figure 3d may not be progenitors, but rather artifacts caused by dissociation for single-cell RNA-seq. The authors should carefully compare these data with double-label histology results and consider that in vivo bihormonal cells are likely very rare.

Comments on CCK regulation of LH and GnRH:
• The claim that CCK regulates LH contradicts previous experiments in the same zebrafish line (Hollander-Cohen, 2024, eLife), where LH cells did not express cckbr, and Ca^{2+} imaging or release experiments showed no significant effect.
• Knockouts reduce FSH and consequently gonadal estrogen, which could indirectly attenuate the GnRH-LH pathway. Gonadal estrogen is established to stimulate this pathway (Huggard-Nelson et al., 2002; Li et al., 2018). Uehara et al., 2024 observed similar LH reductions in intact fish in medaka, while knockout of cckbr in gonadectomized fish did not affect LH. If the authors claim that CCK regulates LH, they must show this effect persists in gonadectomized fish.
• The assertion that GnRH neurons express cckbr is based on hypothalamic single-cell RNA-seq, but the main population of GnRH3 neurons resides in the POA. Even if double in situ hybridization confirmed co-localization, simply having receptors does not establish functional importance; targeted KO or electrophysiological analysis of GnRH3 neurons would be required.
• The claim that CCK injection induces ovulation likely reflects indirect effects via FSH-stimulated estrogen, rather than a direct action on the gonads, and cannot be stated as "CCK facilitates gonadal maturation and ovulation via its dual regulation (direct and indirect) of LH synthesis and secretion (Line 179-180)." These conclusions should be led by more careful observations.

Comments on gonadotropin independent pathway:
• It is interesting that CCK system KO produces more severe reproductive phenotypes than FSH KO. Hollander-Cohen (2024, eLife) also reported this, showing consistency. The authors conclude that CCK KO reduces PGC migration, proliferation, and sycp3 expression, thereby impairing meiosis. However, the source of CCK and its pathway are entirely unexplored, and the only clear conclusion is that the effect is there is something gonadotropin-independent. Furthermore, fish PGCs are known to proliferate even in adults (Nakamura et al., 2010; Zupa et al., 2023), so initial migration deficits do not necessarily explain adult germ cell numbers. Beyond reduced sycp3 expression, the mechanism remains completely unexplored, and further analysis is essential. Without mechanistic explanation for why CCK KO is more severe than FSH KO, no additional conclusions beyond previously reported facts (Hollander-Cohen, 2024) can be drawn.

Version 1:

Reviewer comments:

Reviewer #1

(Remarks to the Author)
The authors have responded to all of my questions and concerns and have extensively revised the manuscript with addition of a substantial amount of new data. This reviewer appreciates the considerable effort involved. However, some questions and concerns remain and should be further addressed or clarified.

1. The authors tested the effects of FSH and GH on the expression of lhb for possible paracrine regulation in the pituitary. This is good. However, what FSH and GH were used in the experiment? Recombinant proteins from zebrafish or other species? If the hormones are heterologous, then the authors need to be cautious in interpreting the data as hormones from different species may not act as specifically as homologous ones. The manuscript also mentioned using recombinant zebrafish LH obtained from a commercial company (Beijing VJTBio). However, no information was given about how the recombinant hormone is produced and how the activity is validated. Also, no information about this hormone is available at the company's website. The authors should add this information in the manuscript.

2. "Unlike mammals, teleosts lack a median eminence. Instead, hypothalamic neurons directly innervate the pituitary, enabling multiple neuropeptides to directly regulate gonadotropes." This statement is questionable as direct innervation is not the condition for multiple neuropeptides to directly regulate gonadotropes. The median eminence allows more neuropeptides or neurotransmitters to directly regulate pituitary cells.
3.
3. Figure S13 examined the effect of GnRH3 on lhb expression. Why not looking at fshb expression as well in the samples?

4. Regarding the use of a gnrh3 knockout to demonstrate the indirect CCK–GnRH3–LH pathway, the authors tried to argue against this approach by invoking possible developmental compensation. This is not a convincing argument. While developmental compensation can occur in stable mutant lines, genetic loss-of-function is still the most appropriate and direct way to test this pathway. By the same logic, the authors would not have performed the experiment using fshb-/- mutant to demonstrate FSH involvement in CCK action. Therefore, developmental compensation alone is insufficient justification to dismiss the gnrh3 knockout approach. It is understandable that gnrh3 mutant line was not available and generating it would take a long time, longer than the time allowed for the revision. This explanation would be good and reasonable enough.

5. The authors performed immunohistochemical staining for LH and FSH. However, the antibodies used were raised by the same group before against grass carp hormones. The specificity of these antibodies in zebrafish is questionable and needs to be validated.

Reviewer #2

(Remarks to the Author)
Overall assessment：
The authors have responded to all of my questions and suggestions and have supplemented the manuscript with a substantial amount of new experimental data. Overall, the revised version represents a marked improvement over the original submission. This manuscript provides a comprehensive and technically impressive analysis of the role of CCK signaling in zebrafish gonadal development. By integrating multiple genetic models and functional assays, the study substantially advances our understanding of CCK as a regulator of both gonadotropin-dependent and -independent processes during early germline development. The overall framework and conclusions are of clear interest for clarifying the physiological roles of CCK in teleost reproduction.
1、 However, several questions and concerns still remain and need to be further addressed or clarified.I do not question the reported reduction in PGC number and the all-male phenotype in CCK mutants. However, PGC number and migration defects occur at early embryonic stages, whereas the somatic cell apoptosis proposed as a mechanism is observed mainly after ~14 dpf, making a direct causal link unclear. How CCK regulates PGC number during early development therefore remains unresolved.
2、 Related to the above point, the authors show that tp53 knockout rescues the meiotic defects caused by loss of CCK (this genetic rescue is very elegant), and the mechanism is attributed to suppression of somatic cell apoptosis. However, the manuscript does not report the sex composition of the cck;tp53 double mutants. The data presented indicate restoration of spermatogenesis, but female development does not appear to be recovered. If the all-male phenotype in CCK mutants is primarily driven by somatic apoptosis, why is female fate not restored when apoptosis is rescued? This suggests that meiotic rescue and sexual fate determination may be separable processes, and further implies that CCK's effects on early PGC behavior cannot be fully explained by somatic apoptosis alone.

Reviewer #3

(Remarks to the Author)
The reviewer would like to acknowledge the considerable effort made by the authors in adding a large number of experiments within a relatively short revision period. These new data are valuable in that they move the study away from the original, incorrect hypothesis that hypothalamic CCK simultaneously regulates both the pituitary and the gonads, and

instead support an independent regulation model, which is likely a more plausible conclusion. This shift demonstrates that the additional experiments have generated important information.
On the other hand, this shift completely changed the concept, and the title “Cholecystokinin integrates gonadotropin-dependent and -independent pathways to orchestrate zebrafish gonadal development” cannot be applied.

Also, even with many additional experiments, the evidence remains insufficient to overturn previously published studies showing little or no direct effect of CCK on LH regulation, and the newly proposed model of local regulation of CCK in the gonads is still not fully supported. In addition, for several of the newly added experiments, validation of the experimental systems and key antibody appears insufficient, leaving uncertainties in data interpretation. Please refer to the specific comments below.

To "Response to Comment 1:"
>We acknowledge the initial concerns regarding the mechanistic depth of our study and the reliance on correlative data from whole-body knockouts and scRNA-seq. In direct response, we have undertaken an extensive series of new experiments designed to transform key correlative observations into causally supported conclusions. The revisions below address each major concern with decisive new evidence.
>(1). Upgrading methodological rigor and externally validating key cell populations

The authors state that cckbrb is expressed in LH cells with the additional data of double labeling of cckbrb mRNA and LH protein. However, when viewed at low magnification, this co-localization is very minor (Supplementary Fig. S15). Even if present, the degree of co-localization is extremely low, which is in clear contrast to the situation in FSH cells, where cckbrb expression is almost complete, as reported previously (Hollander-Cohen et al., 2024; Uehara et al., 2024). Also, if CCK indeed exerts a direct effect on LH, responses comparable to those observed for FSH should have been detected in LH in Hollander-Cohen et al. (2024) (see absence of effect in Hollander-Cohen et al., 2024, Figure4). The present study does not provide sufficient data to overturn the clear difference in responsiveness between FSH and LH reported in prior studies. Therefore, based on previous studies and the data presented here, the current model implying that CCK acts on FSH and LH in a similar manner is not objectively supported.

>(2). Providing mechanistic proof for LH regulation
In your data, only a very small subset of LH cells is reported to express cckbrb. Given this, how can the authors explain a nearly threefold increase in overall LH expression? It is difficult to reconcile these two observations.
Importantly, cckbrb is a receptor that can signal through both Gs and Gq pathways. Therefore, if CCK truly increases LH expression under physiological conditions via a Gs-mediated mechanism, it should also activate Gq signaling and consequently elevate intracellular Ca^{2+} levels. However, as noted above, in freshly prepared pituitary tissues, LH cells show little to no response to CCK peptide (Hollander-Cohen et al., 2024). In other words, the results reported by Hollander-Cohen et al. indicate that, under physiological conditions, CCK does not directly act on LH cells, and likewise do not support a direct increase in LH expression under physiological conditions.
This interpretation is also consistent with the low degree of cckbrb co-localization in LH cells observed in the present study.
As mentioned in my previous comments, considering the findings of Romain et al. (2020), it is well established that culturing pituitary cells alters FSH and LH expression patterns, often leading to increased co-expression of FSH and LH. Thus, the authors may be observing the effects of CCK under these non-physiological, culture-specific conditions.
Unless the authors adequately explain why Hollander-Cohen et al. failed to observe Calcium responses in LH cells upon CCK application, this hypothesis cannot revise the current understanding.

>Functional validation of the GnRH3 pathway:

I acknowledge to establish this new experiment (Supplementary Fig.S14) . However, the experimental design relies on the use of antibodies, and the appropriate control in this case should be normal serum. Without such a control, it is not possible to exclude the possibility that other components present in the serum contributed to the observed reduction in LH levels. Therefore, the experimental system itself requires reconsideration.
In addition, adding immunized rabbit serum to the culture medium at a dilution of 1:500 is too much and may introduce unintended side effects, and these potential confounding effects should be carefully considered and controlled for.
More broadly, for all experiments added during the revision that rely on antibodies, proper validation of the antibodies used is essential. For example, it should be demonstrated whether the effect of GnRH can be abolished by this antibody. Without such validation, the specificity and physiological relevance of the observed effects remain uncertain.

>Precise source of CCK for early gonadal action: In response to the valid criticism regarding the schematic, spatial expression analysis revealed that during early development, the CCK influencing the gonad originates from local tissue, not the hypothalamus (Fig. 4d,e; Fig. R5). We have therefore revised our working model (Fig. 5i) to accurately distinguish between CCK's neuroendocrine role (hypothalamus-pituitary) and its local paracrine role (local source-gonad).

This is a very reasonable set of data. If feasible, demonstrating whether this local source is truly important through a tissue- or cell-type–specific knockout would be necessary to reach a solid conclusion.

>This lack of robust expression in the PGC lineage supports the hypothesis that CCK regulation of PGCs is likely indirect.

Which cell types mediate this effect? At a minimum, tissue-level in situ hybridization is required to address this point.

Overall, in the original version, a working hypothesis was proposed without sufficient evidence, namely that hypothalamic CCK is required for PGC proliferation, and this hypothesis was subsequently reversed in the revision. I believe that interpreting what is not directly observed and elevating such interpretations into hypotheses is potentially dangerous. In this regard, it is appropriate that the authors did not retain the original conclusion in the revised version. In this sense, the all conclusions, including the title and abstract should be carefully reviewed.

To "Response to Comment 3:"
>(1). Discrepancy with Hollander-Cohen et al. (2024):

As stated above, the fact that little to no response is observed in LH:GCaMP recordings is a serious concern. Even if a small response were detected in a very limited subset of LH cells, it would not be reasonable to discuss this effect as operating in parallel with the robust action of CCK on FSH, nor to draw the two as equivalent pathways in a schematic model. Such a representation does not reflect the relative strength or reliability of the underlying data.

[Concerns about the newly added data]
(1) In Fig. S9, it is unclear how the peptide is taken up when delivered through food because peptides are digested in the stomach or gut. Moreover, the reported increase in mRNA expression within 1.5 hours appears unusual and requires further explanation. Given the small sample size (n = 3), it is questionable whether the observed difference is sufficiently robust and clear to support a firm conclusion.

(2) In addition, it is not specified which CCK peptide sequence was used in these experiments. The peptide sequence is not described anywhere in the manuscript and should be explicitly provided.

(3) Information regarding the antibodies and related reagents is insufficient, and their validation is inadequate. For both the neutralizing antibody and the antibodies used for staining, a dilution of 1:500 appears excessively concentrated. Moreover, beyond the statement that the antibodies were "custom-synthesized by Dia-An, Wuhan," no additional information is provided. As a result, the specificity of these antibodies remains unclear.

(4) In Fig. S11, the authors refer to the staining as GnRH immunohistochemistry, but the anatomical location of the signal is unclear. Moreover, GnRH3 neurons are not expected to be present in such large numbers, raising the possibility that the signal represents something else. This raises concerns about whether a specific antibody was used at an appropriate concentration.
Please refer to Spicer et al. (2016) for the expected distribution and characteristics of GnRH3 neurons.
https://doi.org/10.1371/journal.pone.0158141

(5) In lines 587–589, the manuscript states:
"The anti-GnRH3, anti-LH, and anti-FSH antibodies were custom synthesized by Dia-An (Wuhan) following the method of Feng et al. (24) and validated in previous studies (10)."
However, reference 24 describes the generation of a GnRH3 antibody, whereas reference 10 does not provide validation of these antibodies, nor does it clearly describe their origin or specificity. Therefore, the claim that these antibodies were "validated in previous studies" is not supported by the cited references. Moreover, the wording suggests that the antibodies were newly generated for the present study, which is inconsistent with the statement that they have already been validated. As a result, both the provenance and specificity of these antibodies remain unclear.

Version 2:

Reviewer comments:

Reviewer #1

(Remarks to the Author)

Reviewer #2

(Remarks to the Author)
The authors have satisfactorily addressed all of my previous concerns. The revised manuscript shows clear improvements in logic, rigor, and clarity of presentation. Congratulations to the authors on this solid work, and I recommend publication of this manuscript in Nature Communications.

Reviewer #3

(Remarks to the Author)
Through the course of the discussion with the reviewers, it has become clear that the current dataset does not provide decisive evidence supporting a strong direct action on LH. In particular, no critical data have been presented in the revised manuscript that would overturn the previous Ca2+ imaging findings, in which no clear response was observed in LH cells. The rationale underlying the authors' initial conclusion that CCK functions as an LH-releasing hormone, based on culture and knockout experiments, is addressed in detail in the specific comments. However, it is also evident that, throughout the revision process, the authors' interpretation has shifted from asserting a "direct action on LH" toward acknowledging the existence of indirect effects. This shift itself represents the difficulty of positioning CCK as a strong direct regulatory axis for LH.
Crucially, it must be emphasized that the GnRH peptide—the established LH-releasing hormone (LH-RH)—demonstrates a robust induction of both Ca2+ transients and lhb expression in LH cells in zebrafish and other teleosts. While CCK fulfills these criteria for the FSH system, the evidence presented for the LH system remains fundamentally different and insufficient to warrant the designation of "LH-RH."
In light of these considerations, I strongly recommend that the interpretation regarding LH, as well as the overall description throughout the manuscript, be revised in a direction strictly supported by the data or previous studies.
Please see the specific comments below for detailed explanations and suggestions.

>1. Response to "Title and Concept Shift"
>Previous Comment:
>The reviewer noted that the title "integrates" is no longer applicable given the shift to an independent regulation model.
> Authors' Response:
>We fully agree. To accurately reflect this distinction while highlighting that these pathways work in concert to achieve gonadal development, we have modified the title to:
>"Cholecystokinin coordinates gonadotropin-dependent and -independent pathways to orchestrate zebrafish gonadal development"
>We believe "coordinates" better captures the physiological reality: while the pathways are mechanistically distinct and spatially separated, CCK signaling acts as a synchronizing factor to ensure both germ cell establishment and hormonal support.

Reviewer's Comment:

The authors state that they "fully agree" with the concern regarding the independent nature of the pathways; however, no substantive conceptual revision is evident. The revised title replaces "integrate" with "coordinate" and "orchestrate," yet all three terms presuppose a single unified regulatory system—the very premise that has been questioned.
More fundamentally, these terms imply the existence of a central regulatory entity or master-switch mechanism. In contrast, the present study identifies two physiologically distinct systems that utilize the same signaling molecule:
• Hypothalamic CCK acting on the pituitary (gonadotropin-dependent)
• Gonadal CCK acting locally within the gonad (gonadotropin-independent)

Using the term "coordinate" in this context is conceptually problematic. By analogy, acetylcholine is utilized by both parasympathetic neurons regulating cardiac function and motor neurons controlling skeletal muscle contraction. However, no physiologist would describe these distinct systems as being "orchestrated" by acetylcholine merely because they share a common neurotransmitter. They represent independent physiological processes initiated by different neuronal populations under different functional contexts.
Similarly, gonadal growth during early development mediated by local CCK signaling and hypothalamic CCK stimulation of the pituitary during reproductive activation represent distinct physiological events. The mere use of the same signaling molecule does not justify framing them as components of a single coordinated regulatory system.

Unless you can provide evidence of a shared upstream trigger that simultaneously regulates both the hypothalamic and gonadal CCK pools to synchronize these pathways, the terms "coordinate" and "orchestrate" are inappropriate.

The title must be revised to accurately reflect these parallel, independent pathways, such as:
"Hypothalamic and peripheral cholecystokinin stimulate zebrafish gonadal development through gonadotropin-dependent and -independent pathways."

>2. Response to "Mechanistic Proof for LH Regulation"
>Response to LH Regulation & Hollander-Cohen Discrepancy:
>Previous Comment:
>The reviewer questioned the physiological relevance of CCK on LH, citing low cckbrb co-localization and the lack of robust Ca2+ response in Hollander-Cohen et al. (2024). They also suggested that our in vitro results might be culture artifacts (altered expression patterns)
> Authors' Response:
>Regarding Hollander-Cohen et al. (2024): We respectfully note that Hollander-Cohen et al. actually reported that CCK does elicit a calcium response in LH cells, albeit "weaker" and "varied widely" (20%-100% of cells responding). This perfectly

aligns with the well-established existence of a bi-hormonal subpopulation (cells co-expressing FSH and LH) in teleosts. We propose that the responses observed in our study are driven by CCK acting on these cckbrb-positive bi-hormonal cells.
>Regarding Culture Artifacts: We acknowledge the reviewer's concern that primary culture conditions can influence gene expression patterns. However, we respectfully submit that our conclusion is not based solely on in vitro data. The most critical evidence comes from our in vivo genetic models: cckbrb-/- mutants exhibit a marked reduction in pituitary lhb mRNA expression in the intact animal. This in vivo phenotype definitively proves that CCK signaling is physiologically required for maintaining basal LH synthesis. If the CCK-LH regulation were merely a culture artifact, the loss of the CCK receptor in vivo would not result in such a profound suppression of lhb levels. Our in vitro data merely serves to dissect the mechanism (direct vs. indirect) of this established in vivo phenomenon.

Reviewer’s Comment:

The concerns regarding the interpretation of the in vivo data were already raised in the initial review, but are reiterated here for clarity. It is indeed true that lhb expression was reduced in vivo. However, it is important to emphasize that these experiments were conducted in intact animals. In teleosts, gonadal estrogen is widely accepted to stimulate the GnRH–LH axis (Huggard-Nelson et al., 2002; Li et al., 2018). Therefore, it is entirely plausible that disruption of the CCK system reduces FSH levels, leading to impaired gonadal function, which in turn secondarily suppresses LH expression.
Moreover, as the authors themselves show, CCK knockout produces more severe defects in gonadal development than FSH knockout. Such profound gonadal impairment would itself be expected to reduce LH levels. In Fig. 1C, the $ccka^{-/-}$;$cckb^{-/-}$ animals are all male and exhibit markedly deficient testicular development and spermatogenesis. These severe gonadal abnormalities are sufficient to account for reduced LH expression. Therefore, the results obtained in $ccka^{-/-}$;$cckb^{-/-}$ animals cannot be interpreted as evidence that CCK directly regulates LH.
Consistent with this interpretation, in medaka, CCK receptor knockout reduces LH expression in intact animals, but this reduction is not observed in ovariectomized individuals (Uehara et al., 2024; Fig. 1i vs. Fig. 1l). This clearly indicates the presence of a gonad-dependent indirect mechanism. In the zebrafish in vivo data cited in the present manuscript, the possibility of such gonad-dependent indirect effects has not been excluded. By observing the medaka findings, an indirect interpretation appears more likely.

On the Interpretation of the Bi-hormonal Cell Data
The authors argue that the response is limited to a small subpopulation of bi-hormonal cells and suggest that either extreme upregulation within a small number of cells or culture-related effects may account for the “profound” change in whole-pituitary lhb expression. However, drawing the conclusion that CCK strongly regulates LH under such unstable and artificial conditions represents a logical leap (related to the next question).
In the “culture conditions” where LH expression increases, the proportion of bi-hormonal cells (which express cckbr) is also elevated. Stimulation of LH expression in these bi-hormonal cells in vitro does not necessarily imply that such regulation occurs in vivo, where these cells constitute a very minor population. Furthermore, because the authors interpret bi-hormonal cells as progenitor-like cells, it is inappropriate to designate CCK as an “LH-RH” based on effects observed in this limited and potentially transitional cell population.

Inconsistency with Previous Zebrafish Studies
A more critical issue is the insufficient discussion of consistency with existing literature. In zebrafish, Hollander-Cohen et al. reported using Ca2+ imaging that CCK robustly increases Ca2+ signals in FSH cells, whereas no significant response was observed in LH cells. This finding indicates that, at least in zebrafish, the primary direct target of CCK is the FSH system.
To argue that CCK functions as both FSH-RH and LH-RH on the basis of an undetectable effect on a minor FSH/LH co-expressing progenitor subpopulation is not justified. If the effect on LH cells cannot be detected by Ca2+ imaging and is confined to a minor progenitor-like population, it cannot reasonably be equated with the clearly demonstrated FSH-releasing activity.
Although the present manuscript concludes that CCK directly regulates LH, it does not clearly explain how these findings reconcile with previous zebrafish studies. Without resolving inconsistencies across studies—including those conducted in the same species—it is premature to conclude a direct action on LH. Furthermore, increasing in culture condition does not mean “releasing activity”.

Required Discussion Points
At a minimum, the authors should explicitly discuss:
• The zebrafish Ca2+ imaging data
• The medaka ovariectomy experiments
and carefully consider the possibility that the in vitro system does not fully reflect physiological conditions, as well as the likelihood that the observed effects on LH represent indirect mechanisms in a broad sense.
While the authors demonstrate the presence of CCK receptors in LH-expressing cells, these receptors are restricted to a minor bi-hormonal population, and this limitation must be interpreted cautiously.
At present, the only consistently and robustly demonstrated effect is the direct action on the FSH system. In contrast, for the LH system, the available evidence is insufficient to conclusively support a direct regulatory role, and this distinction should be clearly articulated in the manuscript.

>Response to Mechanistic Proof for LH Regulation (Explaining the 3-fold Increase in vitro):

>Previous Comment:
>The reviewer rightly questioned how a "very small subset" of cckbrb-expressing LH cells could sustain a "nearly threefold increase" in total LH expression in primary pituitary culture.

>Authors’ Response:
>We appreciate the reviewer’s keen observation regarding the quantitative discrepancy between the sparse receptor distribution and the robust transcriptional response. We propose that the observed 3-fold increase in our in vitro system is driven by two synergistic factors, one of which aligns with the reviewer's own insight:
>High Transcriptional Amplitude in Target Cells: While the cckbrb-positive subpopulation (bi-hormonal cells) is numerically small, the activation of the Gq-calcium signaling pathway in these cells is likely highly potent. A massive transcriptional induction (e.g., >20-fold) within a small specific subset of cells can mathematically result in a significant (3-fold) increase in the total mRNA pool of the entire culture.
>Culture-Induced Cellular Plasticity (As noted by the reviewer): Importantly, we agree with the reviewer’s suggestion that "culturing pituitary cells alters FSH and LH expression patterns, often leading to increased co-expression." It is highly probable that in our primary culture system, the population of bi-hormonal (FSH+LH / CCK-responsive) cells expands or becomes more sensitive compared to the in vivo state. This culture-specific enrichment of the responsive population provides a logical explanation for why the direct CCK-induced lhb upregulation is more pronounced in vitro (3-fold) than might be predicted solely from in situ staining of fixed tissue.
>Conclusion: Therefore, the in vitro data accurately reflects the intrinsic capability of CCK to drive lhb expression directly in responsive gonadotropes. However, we fully agree that in vivo, this direct pathway operates in concert with the powerful indirect GnRH3 pathway to achieve full physiological regulation.

Reviewer’s Comment:
Thank you for your reconsideration and detailed responses. The authors propose two hypotheses to explain the pronounced induction of lhb observed in vitro:
(1) high-amplitude transcriptional induction in a small subset of cells, and
(2) increased cellular plasticity under culture conditions.

These are important considerations and should be explicitly incorporated into the main text. In particular, the possibility that culture-specific changes in cellular composition influenced the results must be clearly distinguished from interpretations of in vivo physiology.
Furthermore, the authors now state that, in vivo, CCK acts in coordination with a “powerful indirect GnRH3 pathway.” However, given this clarification, the current description of CCK as “acting as a luteinizing-hormone-releasing hormone (LH-RH)” creates an internal inconsistency with the authors’ own revised interpretation.
At present, the data suggests at most the possibility or capacity for a direct action on LH. This is qualitatively different from the well-established and robust direct regulatory axis demonstrated for FSH. It would therefore be inappropriate to position LH regulation at the same conceptual level as FSH control. I strongly recommend revising the use of the term “LH-RH” and clearly distinguishing between limited direct effects and indirect pathways.
In Hollander-Cohen, GnRH action on LH cells is clearly shown in Ca^{2+} imaging; this is “LH-RH”.

On the Proposed CCK → GnRH3 Pathway
The assertion of a “powerful indirect GnRH3 pathway” is based primarily on the culture experiment shown in Fig. S14. However, GnRH3 blockade explains only a small fraction (approximately ~20%) of the CCK-induced increase in LH expression. This is fundamentally different from the data in Fig. R6, where exogenous GnRH3-induced responses are almost completely abolished by GnRH3 antibody blockade.
These results indicate that the CCK → GnRH3 pathway accounts for only a minor component of the observed LH induction. Indeed, the majority of the LH increase persists even when GnRH3 signaling is blocked. When considered together with the authors’ own acknowledgment that long-term culture markedly increases the proportion of bi-hormonal cells, the most parsimonious interpretation is that the observed LH upregulation largely reflects culture-induced changes in cellular composition rather than a dominant physiological CCK → GnRH3 axis.
Moreover, this proposed pathway is supported by a single experimental approach and lacks independent validation, such as receptor co-localization by in situ hybridization. The evidentiary support is therefore limited. At this stage, positioning the CCK → GnRH3 pathway as a major regulatory axis leaves substantial room for alternative interpretations.

Conclusion and Recommendation
Taken together, the current description of CCK as “acting as a luteinizing-hormone-releasing hormone (LH-RH)” exceeds what is supported by the presented data and lacks appropriate caution. The data demonstrate, at most, the possibility of a direct influence on LH. This does not yet warrant placing LH regulation on the same level as the more robustly supported FSH regulatory axis.
The conclusions that are clearly supported by the present study are:
• Hypothalamic CCK is involved in FSH regulation.
• CCK may influence germ cell proliferation at the gonadal level.
These findings are substantial and valuable in their own right.
However, the proposed LH regulatory mechanism would represent a revision of the existing framework in the field, and the current evidence is insufficient to justify such a strong claim. Drawing definitive conclusions without adequate empirical support is premature.
Accordingly, I strongly recommend a substantial tone-down of the conclusions regarding LH regulation, along with explicit acknowledgment of culture-specific effects and unverified pathways as limitations of the study.

Revisions Required to Ensure Consistency with the Consensus Reached During the Review Process
Several sections of the manuscript deviate from the interpretation that has been refined and clarified through the review process. These should be revised accordingly.

Line 8
“orchestrating” → “stimulating”
The term “orchestrating” implies a central and integrative regulatory role that exceeds the scope of the current evidence. A more neutral term such as “stimulating” is appropriate.

Line 9
“(1) Acting as a luteinizing-hormone-releasing hormone (LH-RH), CCK directly activates pituitary lhb transcription via CCKBRb and indirectly enhances hypothalamic gnrh3 expression to regulate LH synthesis and secretion.”
As discussed throughout the review process, the evidence supporting CCK action on LH is not comparable in strength to that for the FSH system (or GnRH action on LH). Based on the current data, it is not appropriate to position CCK as a luteinizing-hormone-releasing hormone (LH-RH). The present wording conveys a stronger conclusion than is warranted by the available evidence.
The increase in lhb expression observed under culture conditions may reflect culture-specific changes in cellular composition, including expansion of bi-hormonal cells. Therefore, extrapolating these findings to in vivo physiology requires caution.
Furthermore, it is well established that changes in gonadal function can secondarily influence LH expression. The in vivo data presented here do not allow a clear distinction between direct pituitary effects and indirect gonad-mediated mechanisms.
Accordingly, this statement should either be deleted or revised in a substantially more conservative manner. For example:
“CCK may influence LH synthesis through indirect mechanisms.”
This wording remains within the limits of the current data and avoids overstating a direct regulatory role.

Line 19
The terms “central integrator” and “synchronizing” imply unified central control by a single CCK system. However, the present study suggests that hypothalamic and peripheral CCK may regulate reproductive function through distinct pathways. Thus, describing CCK as a “central integrator” is inconsistent with both the current data and the interpretive consensus reached during review.
I recommend revising to:
“Hypothalamic and peripheral CCK cells regulate gonadal function via gonadotropin-dependent and -independent pathways, respectively.”

Line 23
Keyword: “LH-releasing hormone” should be deleted.

Lines 50–53
The statement that CCK action on LH “has not been previously explored” is inconsistent with existing literature. Indirect effects of CCK on LH via the gonad have already been discussed in Uehara et al., 2024. Moreover, Hollander-Cohen et al., 2024 reported LH-related analyses in cckr knockout zebrafish, including Ca2+ imaging in LH cells. Therefore, it is inappropriate to suggest that LH regulation has not been examined.
However, understanding of endogenous CCK ligands remains limited. Ligand-level experimental validation has thus far been performed only in medaka, whereas zebrafish studies have primarily focused on receptor-level analyses. It remains unclear whether CCK itself or other peptides such as gastrin serve as the physiologically relevant endogenous ligand(s) in zebrafish.
The current findings are significant in demonstrating that CCK functions as a ligand in zebrafish. Therefore, I recommend reframing the question as follows:
“However, while ligand-level analyses have been performed in medaka, studies in zebrafish have primarily focused on receptor activation and knockout models, and the identity of the physiologically relevant endogenous ligand(s) within the CCK/gastrin family remains unclear. Therefore, a broader consensus regarding CCK regulation of gonadotropins across teleost species has yet to be established.”

Line 79
“Finally, CCK acts as a LH-RH to induce oocyte maturation and ovulation.”
This statement again designates CCK as LH-RH. Given the high likelihood of indirect mechanisms, this exceeds the scope of the evidence and should be deleted.

Lines 430–447
This paragraph contains interpretations that deviate from the consensus established during review. In particular, describing CCK as a “dual gonadotropin-releasing factor” or “master coordinator” represents a conceptual model that is not supported by the current data.
I strongly recommend deletion of this entire paragraph.
Instead, the discussion should acknowledge that the apparent stimulation of LH may reflect secondary effects mediated through FSH and gonadal function, and should be interpreted cautiously within that framework.

Lines 455–458
“Finally, during the final maturation and ovulation phase, CCK enhances LH release via dual hypothalamic (gnrh3 upregulation) and pituitary (lhb induction) mechanisms to facilitate ovulation.”
This statement is not consistent with the consensus reached during review. Neither prior studies nor the present study demonstrate that CCK increases LH specifically during the ovulation phase. This constitutes speculation beyond the presented data and should be deleted.

Lines 458–460
“Thus, CCK emerges as a central integrator of metabolic and reproductive signals, synchronizing gonadotropin-independent and -dependent pathways across gonadal ontogeny.”
The terms “central integrator” and “synchronizing” overstate the conclusions relative to the evidence. A more neutral and accurate description would be:
“Thus, hypothalamic and peripheral CCK can act through gonadotropin-dependent and -independent pathways, respectively, in teleost reproduction.”

Author’s response
>3. Response to Antibody Specificity & Validation (Crucial Revisions)
>Control for Functional Assays (Response to "Serum Control"): The reviewer correctly pointed out that normal serum is the appropriate control for whole serum antibodies. However, we clarify that all custom antibodies used in functional blocking experiments were affinity-purified, not whole serum. Therefore, the vehicle (PBS) is the appropriate control, as non-specific serum proteins are absent. This eliminates the confounding effects of serum components.

Reviewer’s Comment:
In the GnRH antibody experiment, only the GnRH-Ab group involved immunoglobulin purified from serum, which inevitably contains contaminating serum-derived components and, at minimum, non-specific IgG fractions. This introduces a potential confounding factor that is not present in the other experimental groups.
Ideally, the experiment should have included a control antibody preparation purified from serum in the same manner (e.g., non-immune IgG or pre-immune serum processed identically), in order to control for possible effects of co-purified serum proteins or IgG itself. Without such a control, it cannot be excluded that part of the observed effect reflects non-specific influences of serum-derived components rather than specific GnRH neutralization.
Therefore, this methodological limitation should be explicitly described in the manuscript, and the interpretation of the GnRH blockade results should be presented with appropriate caution.

Open Access This Peer Review File is licensed under a Creative Commons Attribution 4.0 International License, which permits use, sharing, adaptation, distribution and reproduction in any medium or format, as long as you give appropriate credit to the original author(s) and the source, provide a link to the Creative Commons license, and indicate if changes were made.
In cases where reviewers are anonymous, credit should be given to 'Anonymous Referee' and the source.
The images or other third party material in this Peer Review File are included in the article’s Creative Commons license, unless indicated otherwise in a credit line to the material. If material is not included in the article’s Creative Commons

license and your intended use is not permitted by statutory regulation or exceeds the permitted use, you will need to obtain permission directly from the copyright holder.
To view a copy of this license, visit https://creativecommons.org/licenses/by/4.0/

Responses to the Comments from Editor and the three Reviewers

Cholecystokinin integrates gonadotropin-dependent and independent pathways to orchestrate zebrafish gonadal development

Contents

Reviewer #1 (Remarks to the Author): 1
Response to Comment 1: 2
Response to Comment 2: 4
Response to Comment 3: 5
Response to Comment 4: 7
Response to Comment 5: 9
Response to Comment 6: 14
Response to Comment 7: 18
Response to Comment 8: 22
Response to Comment 9: 25
Response to Comment 10: 27
Response to Comment 11: 28
Response to Comment 12: 29
Response to Comment 13: 31
Response to Comment 14: 33
Response to Comment 15: 36
Response to Comment 16: 40
Response to Comment 17: 41
Response to Comment 18: 43
Response to Comment 19: 43
Response to Comment 20: 45
Response to Comment 21: 45
Response to Comment 22: 45
Response to Comment 23: 46
Response to Comment 24: 47
Response to Comment 25: 49
Response to Comment 26: 51
Response to Comment 27: 53
Response to Comment 28: 54
Response to Comment 29: 54
Response to Comment 30: 55
Response to Comment 31: 56
Reviewer #2 (Remarks to the Author): 58
Response to Comment 1: 59
Response to Comment 2: 60
Response to Comment 3: 62
Response to Comment 4: 63
Response to Comment 5: 66
Response to Comment 6: 71
Response to Comment 7: 74

Response to Comment 8: 77
Response to Comment 9: 78
Response to Comment 10: 79
Response to Comment 11: 80
Response to Comment 12: 82
Response to Comment 13: 84
Response to Comment 14: 85
Reviewer #3 (Remarks to the Author): 87
Response to Comment 1: 87
Response to Comment 2: 96
Response to Comment 3: 100

Reviewer #1 (Remarks to the Author):

Using a combination of gene editing (cck1/cck2 double knockout and cckbrb receptor knockout), comparative analysis with gonadotropin knockouts (fshb, lhb, and double knockouts), single-cell RNA sequencing, cell culture experiments, and rescue assays, this study investigated the role of cholecystokinin (CCK) in zebrafish gonadal development. In addition to confirming recent findings that CCK acts as a follicle-stimulating hormone-releasing hormone (FSH-RH) in teleosts, the authors aimed to demonstrate additional roles of CCK in zebrafish reproduction, both gonadotropin-dependent and independent. The major conclusion of the study is that CCK is not merely an FSH-RH, but also a LH-RH; and it not only regulates gonadotropins, but functions as a master regulator that orchestrates gonadal development. The study is overall well planned and performed with substantial amount of data supporting the conclusions. My questions are organized around these two major aspects of proposed CCK functions.

Response:

We sincerely thank the reviewer for this highly thoughtful and positive summary of our work, and for recognizing the robustness of our experimental design and the substantial dataset provided. We are encouraged by your acknowledgement of our central conclusions: that CCK functions not only as an FSH-RH but also as an LH-RH (dual gonadotropin regulation), acting as a master regulator that orchestrates both gonadotropin-dependent and -independent aspects of gonadal development. Below, we provide a point-by-point response to your specific questions regarding these two major functional aspects, supported by extensive new experimental evidence that significantly strengthens the mechanistic insights of our study.

CCK as FSH-RH and LH-RH:

1. The finding that CCK functions as an FSH-releasing hormone in zebrafish is not novel, as this has been well established in recent studies in both zebrafish (Hollander-Cohen et al. 2024) and medaka (Uehara et al. 2024). This study essentially corroborates these previous results; however, the manuscript does not provide sufficient comparison or discussion of these earlier investigations.

Response to Comment 1:

We acknowledge that our original discussion did not sufficiently contextualize our findings against these pivotal studies. To address this comprehensively, we have extensively revised the Discussion sections to explicitly cite Hollander-Cohen et al. and Uehara et al. as the foundational studies establishing the CCK-FSH axis. In the revised text, we now clearly delineate between results that corroborate existing models (CCK-FSH) and those that advance the field (CCK-LH and CCK-Gonad/Soma). Furthermore, we have added a dedicated section in the Discussion to position our findings within the current landscape. This section highlights that while the FSH-RH role is conserved, the LH-RH function and the direct, early gonadal regulation we report represent a major expansion of CCK's known neuroendocrine repertoire. We have revised the Discussion section to explicitly compare our work with recent literature and highlight our novel contributions:

Revised Discussion Excerpt 1: Confirmation of FSH-RH Role

"Following gonadal differentiation, zebrafish undergo an extended developmental phase characterized by follicle transition from Stage IB to Stage II, marking female puberty onset[40, 41]. where FSH plays a central role. Our observation that loss of CCK signaling leads to a severe reduction in *fshb* synthesis, which is rescuable by exogenous CCK, aligns robustly with recent breakthroughs in the field. Specifically, seminal studies in medaka[8] and zebrafish[9] have identified CCK as a potent FSH-RH. Our data, derived from comprehensive genetic knockout models, provide independent confirmation of this conserved physiological axis, reinforcing the consensus that CCK directly modulates FSH output to drive gametogenesis in teleosts."

Revised Discussion Excerpt 2: Confirmation of LH-RH Role

"Upon completion of gametogenesis, the gonads enter the final maturation stage, during which LH plays a critical role in promoting oocyte maturation and ovulation. In *lhb*$^{-/-}$ zebrafish, oocyte growth proceeds normally, but final maturation and ovulation are significantly impaired[11, 12, 47]. Building upon the established role of CCK as a major FSH-RH, our study reveals a significant expansion of its neuroendocrine repertoire: CCK also functions as a critical regulator of LH in zebrafish. Both *cck1*$^{-/-}$;*cck2*$^{-/-}$ and *cckbrb*$^{-/-}$ mutants

exhibited markedly reduced pituitary LH expression, implicating CCK in LH synthesis and secretion. The apparent discrepancy with earlier studies concluding a specific FSH-RH role likely stems from their primary focus on establishing that novel function, and from our identification of a specific cellular substrate: a subpopulation of pituitary gonadotropes that co-express *fshb* and *lhb* transcripts[43] and are enriched for the *cckbrb* receptor, providing a basis for dual regulation.

Mechanistically, CCK regulates LH through a dual pathway. It directly stimulates *lhb* expression in pituitary cells, an action supported by the presence of CCKBRb in cells co-expressing FSH and LH. Additionally, CCK operates via an indirect, GnRH3-mediated route, significantly upregulating hypothalamic *gnrh3* expression. This dual mechanism ensures robust LH output. Functionally, the physiological relevance of this regulation is confirmed by the effective induction of ovulation in female zebrafish following exogenous administration of either CCK or LH. Therefore, our findings extend the existing paradigm, positioning CCK not as a solo FSH-RH, but as a dual gonadotropin-releasing factor that integrates the control of gametogenesis (via FSH) and final maturation/ovulation (via LH), underscoring its role as a master coordinator within the teleost reproductive neuroendocrine system."

2. The manuscript provided evidence that CCK also functions as an LH-releasing hormone in addition to its role in stimulating FSH. This observation agrees well with the previous report by Hollander-Cohen et al. in zebrafish that LH secretion was shut down in CCK receptor knockout. This study went further by proposing two mechanisms underlying such regulation: direct regulation at pituitary level and indirect regulation via GnRH3 in the hypothalamus. However, further evidence is needed to validate this regulation as commented below.

Response to Comment 2:

We sincerely appreciate the reviewer's insightful summary and recognition that our findings on CCK-mediated LH regulation align with the recent phenotypic observations by Hollander-Cohen et al. We fully agree that establishing the precise mechanisms—specifically the dual direct and indirect pathways—requires rigorous validation. To address this, we have performed extensive additional experiments, including dual FISH/IHC co-localization and functional explant assays with pathway blockers, to definitively substantiate this model. We provide the detailed evidence supporting both regulatory pathways in our point-by-point responses to the specific comments below.

3. L153: direct pituitary actions: Is there any evidence for expression of CCK receptor(s) in the LH cells? The single cell expression data did not provide such information. The previous study by Hollander-Cohen et al. reported that zebrafish pituitary only expresses cckbra, not cckbrb, in contrast to the present study. This issue was not touched at all in the manuscript. In medaka, knockout of CCK signaling also resulted in reduced expression of lhb; however, this reduction was proven to be due to the secondary effects of steroid deficiency, not CCK.

Response to Comment 3:

We sincerely thank the reviewer for raising these critical points, which allow us to comprehensively clarify the mechanism of LH regulation and reconcile our findings with prior literature. Regarding the gene nomenclature, we appreciate the reviewer's attention to this detail, as the annotation of teleost gene duplicates has evolved in public databases. To ensure consistency with current standards, we strictly adhered to the official NCBI and ZFIN nomenclature for our target gene, *cckbrb* (Accession: XM_017357750.4). Importantly, sequence alignment confirms that this gene corresponds to the exact same locus referred to as *cckbra* in the study by Hollander-Cohen et al. Thus, the discrepancy is purely terminological due to database updates, while the biological target investigated in both studies is identical.

To address the pivotal question of cellular localization and reconcile our findings with those of Hollander-Cohen et al. (who localized the receptor to FSH cells), we integrated single-cell transcriptomics with high-resolution imaging. First, our single-cell RNA sequencing (scRNA-seq) analysis identified a distinct subpopulation of gonadotropes that co-express both *fshb* and *lhb* transcripts. Notably, we detected specific expression of *cckbrb* within this dual-hormone cell population (Fig. 3d). To validate this finding spatially, we performed dual labeling using fluorescence *in situ* hybridization (FISH) for *cckbrb* mRNA and immunohistochemistry (IHC) for LH protein. This confirmed that *cckbrb* is indeed expressed in LH-producing cells (Supplementary Fig. S15). Taken together, these data suggest that the *cckbrb* expression previously observed in "FSH cells" (Hollander-Cohen et al.) likely corresponds to this specific FSH/LH dual-expressing subpopulation. Our data confirms that these cells actively produce LH, providing the structural basis for direct LH regulation.

Finally, to validate this direct mechanism and exclude secondary steroid effects (as observed in medaka), we performed primary pituitary cell culture experiments. We observed that CCK treatment robustly upregulates *lhb* mRNA expression *in vitro* (Fig. 3i). Since this *in vitro* system is devoid of gonads and systemic steroid feedback, the upregulation of *lhb* cannot be a secondary effect of steroid deficiency. Collectively, these anatomical and functional data confirm that CCK acts directly on pituitary gonadotropes to drive LH synthesis.

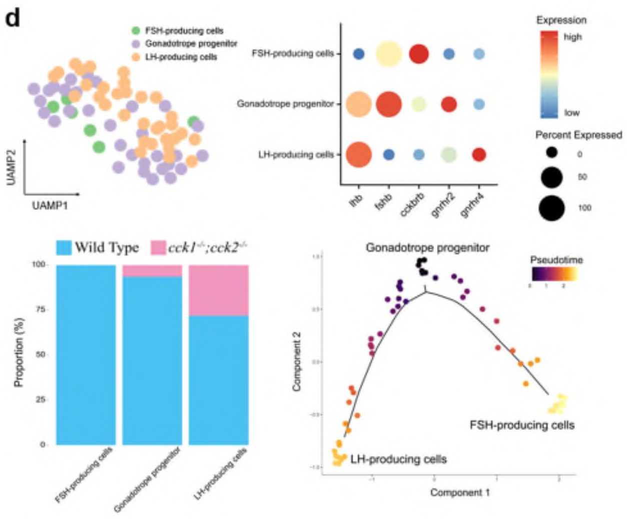

Fig. 3d Sub-clustering and trajectory analysis of the gonadotrope cell population.

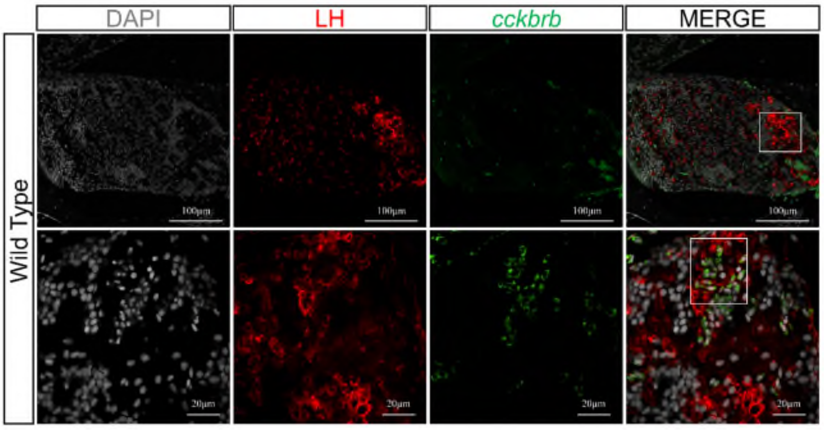

Supplementary Fig. S15 | Co-localization of *cckbrb* mRNA and LH protein in the adult male zebrafish pituitary gland. *cckbrb* mRNA was detected using a fluorescent probe (via Fluorescence *In Situ* Hybridization), and LH protein was visualized via immunohistochemistry (IHC).

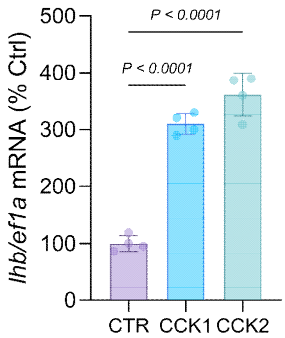

Fig. 3i. qPCR analysis of *lhb* gene expression in pituitary cells treated with CCK1 or CCK2 for 24 hours (n= 4)

4. L157: Any evidence for CCK acting via its receptor cckbrb in regulating LH? A simple reduction of lhb expression in the mutants does not sufficiently support its signaling mechanisms in LH cells. The authors do provide data showing CCK stimulation of lhb expression in vitro; however, this should be interpreted with caution because it has been reported that pituitary hormones may regulate each other via paracrine pathways.

Response to Comment 4:

We thank the reviewer for this highly insightful comment regarding the distinction between direct receptor activation and secondary paracrine signaling. We agree that rigorous evidence is required to definitively attribute the LH upregulation to direct CCKBRb signaling. Based on our single-cell transcriptome data and phenotypic observations, we propose a precise "Dual-Hormone Cell" mechanism that explains both the direct regulation and the specific phenotypic outcomes.

Our single-cell RNA sequencing (scRNA-seq) data reveals that *cckbrb* is expressed in the FSH cell lineage, specifically enriching in a subpopulation that co-expresses both FSH and LH (Fig. 3d). In contrast, the "LH-only" cell population lacks *cckbrb* expression. This specific cellular architecture explains the differential phenotypes observed in our knockout models (Fig. 3a): the loss of CCK signaling leads to a severe reduction in FSH (suggesting the receptor-positive population is critical for FSH maintenance), whereas LH signals are significantly reduced but not eliminated. This partial LH loss corresponds to the specific depletion of the CCK-dependent "dual-hormone" population, while the receptor-negative "LH-only" population continues to produce LH.

To functionally rule out the possibility that this LH regulation is merely a secondary effect of paracrine signaling from other pituitary cells, we performed additional *in vitro* pituitary cell culture experiments. We specifically tested the effects of FSH and growth hormone (GH) on *lhb* expression. Our new data shows that FSH treatment actually inhibits *lhb* expression, while GH treatment has no significant effect (Fig. R1). This creates a logical exclusion: if CCK regulated LH solely via FSH release (paracrine), *lhb* levels should decrease upon CCK stimulation. The fact that CCK upregulates *lhb*, combined with the lack of induction by GH, confirms that the effect is direct and not mediated by paracrine loops involving neighboring hormone-secreting cells.

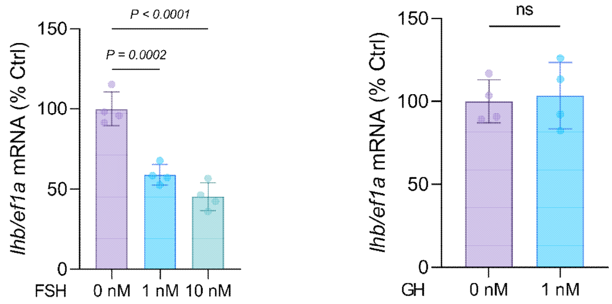

Fig. R1 | Effects of FSH and GH treatment on *lhb* expression in zebrafish primary cultured pituitary cells. qPCR analysis of *lhb* mRNA in primary pituitary cells treated with FSH (left) or GH (right) for 24 hours. Data are presented as mean ± s.d. (n= 4)

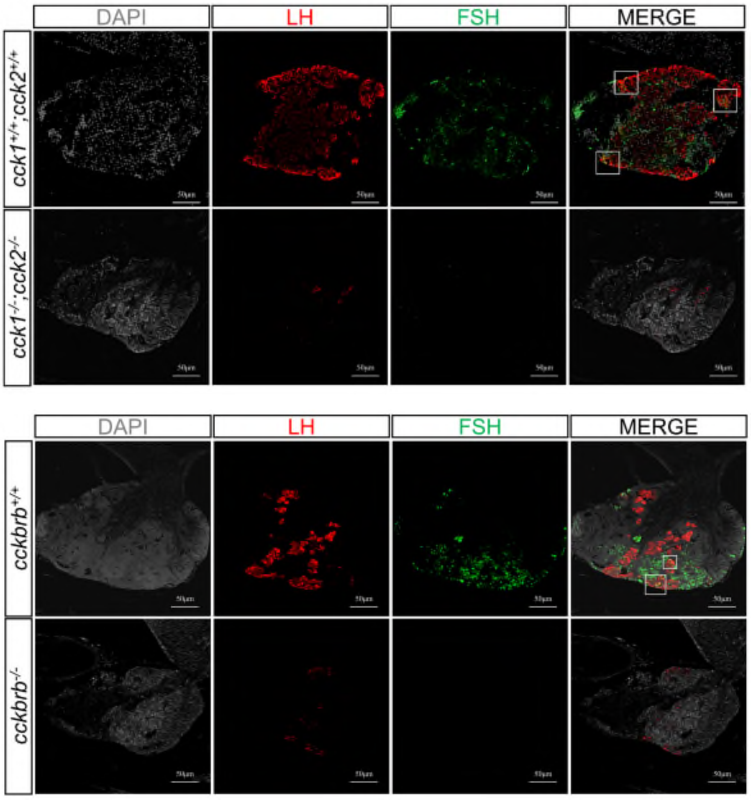

Fig. 3a Representative immunohistochemical staining of LH and FSH proteins in the pituitary of WT, *cck1*$^{-/-}$;*cck2*$^{-/-}$, and *cckbrb*$^{-/-}$ zebrafish.

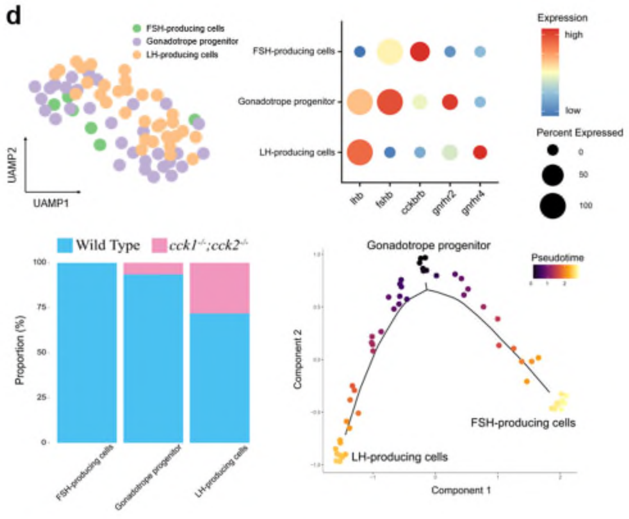

Fig. 3d Sub-clustering and trajectory analysis of the gonadotrope cell population.

5. The paper proposes that CCK may indirectly control LH biosynthesis via hypothalamic GnRH3. However, the importance of GnRH system (both GnRH2 and 3) in controlling pituitary gonadotropins has been rejected by recent genetic studies. Given this, the functional importance and physiological relevance of CCK regulation of LH via GnRH3 is questionable.

Response to Comment 5:

We thank the reviewer for raising this critical point regarding the functional importance of the proposed CCK-GnRH3-LH axis, especially in the context of recent debates on the role of GnRH in teleosts. We agree that the physiological dominance of GnRH has been questioned by genetic knockout studies where reproduction remains largely intact. However, we respectfully posit that this does not negate the role of GnRH as a key regulator of LH, but rather highlights the evolutionarily redundant and compensatory nature of the neuroendocrine system in teleosts. Our defense of the physiological relevance of the CCK-GnRH3-LH pathway is based on the following points:

（1）**Fundamental divergence in teleost neuroendocrinology:** Unlike mammals, teleosts lack a median eminence. Instead, hypothalamic neurons directly innervate the pituitary, enabling multiple neuropeptides to directly regulate gonadotropes. This anatomical difference underpins a paradigm of multi-modal and redundant control over gonadotropin secretion (Fig. R2). Thus, the absence of a severe phenotype in GnRH-deficient models can be explained by functional compensation from other direct regulators, such as Neurokinin B, Secretogranin-2, and as we demonstrate here, CCK. The system is robust, not GnRH-dependent.

[Figure Redacted]

Figure R2. Schematic digram of a lateral view onf mouse (A) and the zebrafish (B) brain. In mammals (A), the GnRH cells (red) project to the median eminence, a highly vascularized connection to the pituitary. In teleosts (B), the neurohypophysis (NeH) is reduced and the GnRH cells make neural connections with the pituitary (Whitlock et al., 2019).

（2）**Functional specialization and divergent regulation of FSH and LH validate the pathway's logic.** The reviewer's point rightly reflects a major shift in our understanding of teleost gonadotropin regulation. It is now clear that in zebrafish, FSH and LH are not

only functionally distinct but are also regulated by largely separate upstream pathways (Fig. R3), which explains the resilience of the reproductive axis to the loss of any single factor. FSH is the key hormone for gonadal growth and gametogenesis. Critically, as highlighted by the reviewer and supported by recent genetic studies, GnRH is not the primary regulator of FSH. Instead, CCK acts as a major FSH-releasing hormone (FSH-RH). LH is essential specifically for final oocyte maturation and ovulation. This dichotomy is crucial: The pathway governing final maturation (LH/ovulation) need not be the same as the pathway governing growth (FSH/development). Therefore, questioning the importance of the GnRH3-LH axis for ovulation—based on its minor role in FSH-driven development—overlooks this fundamental functional and regulatory divergence.

[Figure Redacted]

Figure R3. Functional specialization and divergent regulation of FSH and LH in the teleost reproductive axis.

（3）**Direct molecular and functional evidence for the axis:** Crucially, our study provides direct evidence supporting each step of the CCK-GnRH3-LH axis:

Step 1 (CCK-GnRH3): We show that CCK treatment significantly upregulates *gnrh3* expression in primary hypothalamic cultures (Fig. 3h).

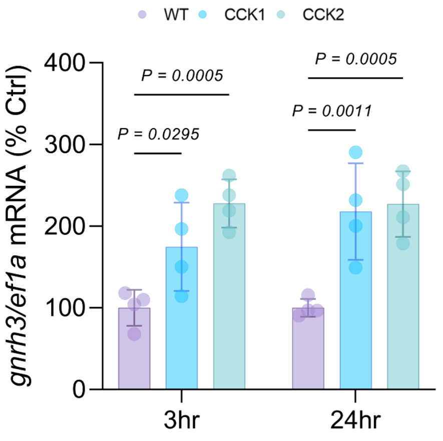

Fig. 3h *gnrh3* mRNA expression in hypothalamic cells treated with CCK1 or CCK2 for 3 hours and 24 hours (*n*= 4).

Step 2 (GnRH3 Receptor Presence): Our scRNA-seq data confirm that LH-producing cells in the zebrafish pituitary express GnRH receptors (Fig. 3d and Supplementary Fig. S7), providing the necessary molecular machinery for them to respond to GnRH3.

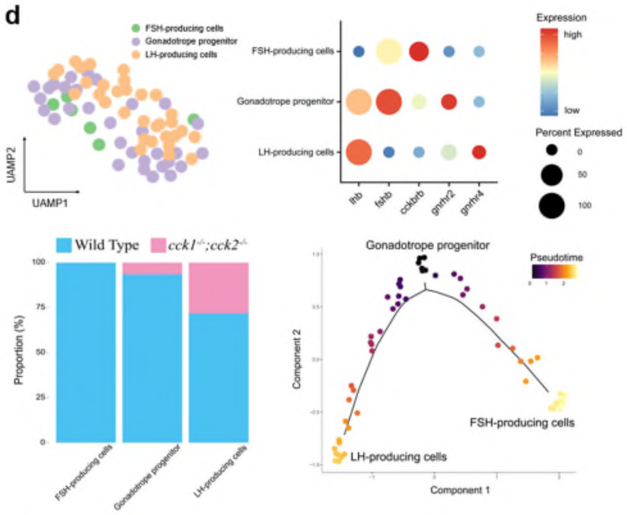

Fig. 3d Sub-clustering and trajectory analysis of the gonadotrope cell population.

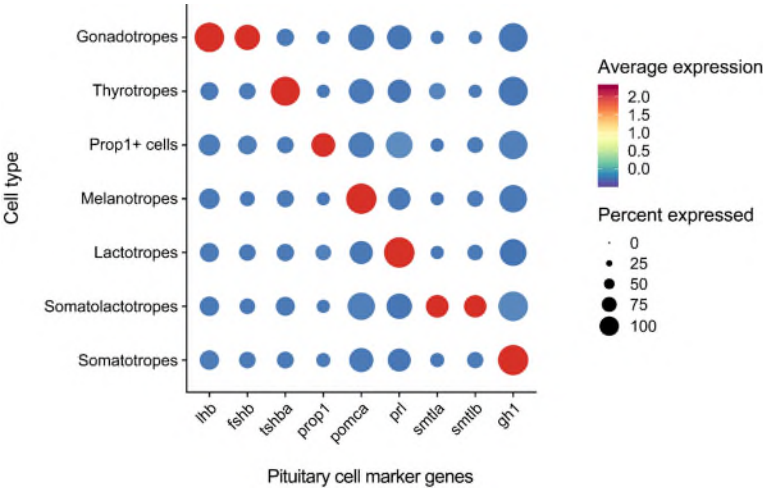

Supplementary Fig. 7. Dot plot revealing distinct marker gene expression patterns across zebrafish pituitary cell populations.

Step 3 (GnRH3-LH): In direct response to this comment, we have conducted new *in vitro* experiments treating primary pituitary cells with GnRH3. The results, now included as Supplementary Fig. S13, clearly demonstrate that GnRH3 directly stimulates *lhb* expression, functionally validating this final step of the pathway.

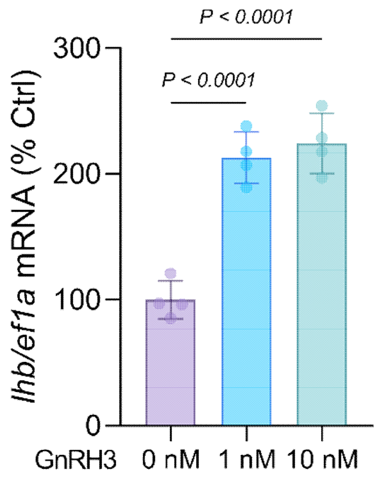

Supplementary Fig. S13 | GnRH3 stimulates *lhb* expression in pituitary cells *in vitro*. qPCR analysis of relative *lhb* mRNA expression in primary cultured zebrafish pituitary cells treated with GnRH3 for 24 hours. Data are presented as mean ± s.d. for *n*= 4 independent biological replicates. Expression levels were normalized to *ef1a*.

Step 4 (Functional Output): Finally, exogenous CCK administration successfully induces ovulation (Fig. 3k), demonstrating the physiological relevance of this integrated pathway.

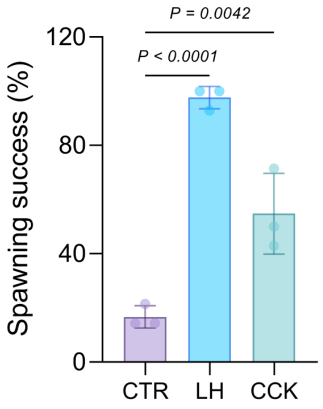

Fig. 3k Functional assessment of ovulation success rate (%) in female zebrafish 24 hours after *i.p.* injection of LH or CCK (n= 14 biologically independent animals from 3 independent experiments).

In light of this multi-layered evidence, we propose that CCK does not operate through a fragile or secondary pathway, but rather acts as an upper-level integrator that enhances the entire pro-ovulatory signal. It does so by co-activating both the canonical GnRH3 pathway and a parallel direct pituitary pathway to ensure a robust LH surge.

6. L167: This statement is not supported by strong evidence. The best way to demonstrate such mechanism is to perform the analysis in gnrh3 mutant.

Response to Comment 6:

We thank the reviewer for this suggestion. While we agree that genetic models are powerful tools, we respectfully propose that for dissecting this specific signaling node, an acute functional blockade provides clearer mechanistic insight than a stable mutant, while also being feasible within the revision timeline.

Scientific rationale for the experimental design. Our study establishes that CCK regulates LH via two parallel pathways: a direct pituitary action and an indirect GnRH3-mediated pathway. In a stable *gnrh3* knockout, the direct CCK-LH pathway would remain intact, and developmental compensation by other neuropeptides (common in zebrafish gonadotropin regulation) would likely mask the specific contribution of the indirect arm. This redundancy explains why *gnrh3* knockouts often lack severe adult phenotypes. Therefore, to specifically isolate the GnRH3-dependent component of CCK action without the confounding effects of developmental compensation, we employed an acute antibody-based blockade system.

We developed a hypothalamic-pituitary co-culture system and treated it with a GnRH3-neutralizing antibody to dissect these pathways. The results, presented in Supplementary Fig. S14, clearly demonstrate that while CCK treatment alone robustly stimulates *lhb* expression, this induction is significantly attenuated (but not abolished) when GnRH3 signaling is blocked by the antibody.

This experiment provides definitive proof of concept: the attenuation represents the GnRH3-dependent indirect pathway (validating the reviewer's query), while the residual induction confirms the existence of the direct pituitary pathway. When combined with our molecular evidence that CCK upregulates hypothalamic *gnrh3* (Fig. 3g, h), that GnRH3 neurons express CCK receptors (Supplementary Fig. S12), and the anatomical proximity of these neurons (Supplementary Fig. S11), this functional blockade offers the most compelling evidence for the physiological relevance of the CCK-GnRH3-LH axis.

Editorial Note: Supplementary Fig. S14a in this Peer Review File is reproduced with permission from BioRender, Created in BioRender. Li, H. (2026) https://BioRender.com/hgl9icn

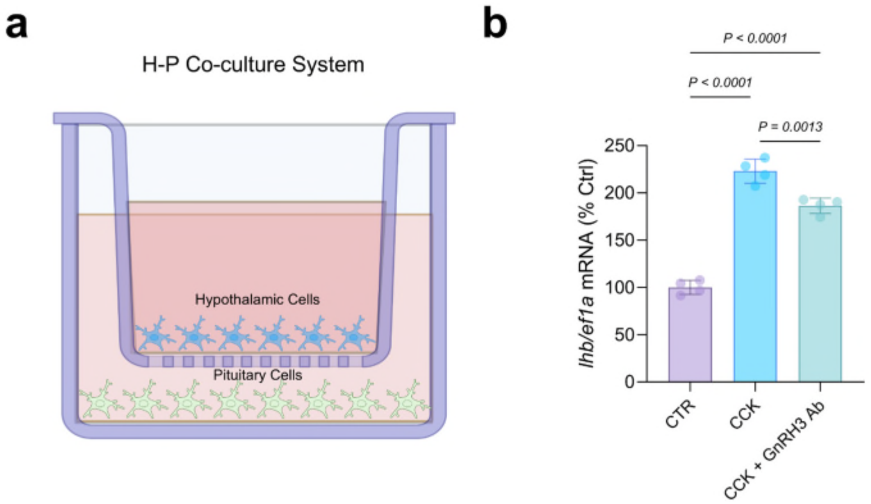

Supplementary Fig. S14 | Hypothalamic-Pituitary (H-P) co-culture system and functional assessment of dual CCK regulation. a Schematic diagram of the H-P co-culture system. Hypothalamic cells are cultured on the transwell insert membrane, and pituitary cells are cultured in the lower chamber, facilitating the transfer of soluble factors. **b** qPCR analysis of *lhb* mRNA expression in pituitary cells from the co-culture system following treatment with Control (Ctrl), CCK only (CCK), and CCK with GnRH3 blocking antibody (CCK + GnRH3 Ab). Data are presented as mean ± s.e.m. (standard error of the mean) for *n*= 4 independent experiments.

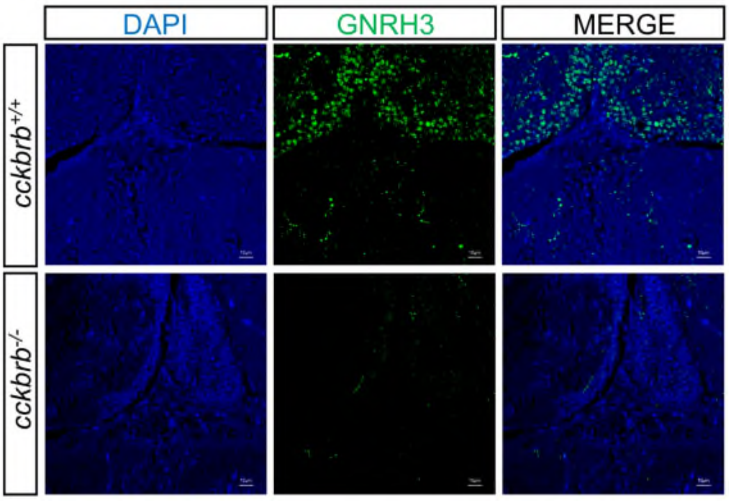

Supplementary Fig. S11 | Immunohistochemical staining of GnRH3 protein in hypothalamic sections from male *cckbrb*$^{+/+}$ and *cckbrb*$^{-/-}$ zebrafish. Representative images of Immunohistochemical (IHC) staining for GnRH3 protein in hypothalamic sections of male *cckbrb*$^{+/+}$ and *cckbrb*$^{-/-}$ zebrafish. Scale bar, 50μm.

Editorial Note: Supplementary Fig. S12a in this Peer Review File is reproduced with permission from BioRender, Created in BioRender. Li, H. (2026) https://BioRender.com/ggl0y1d

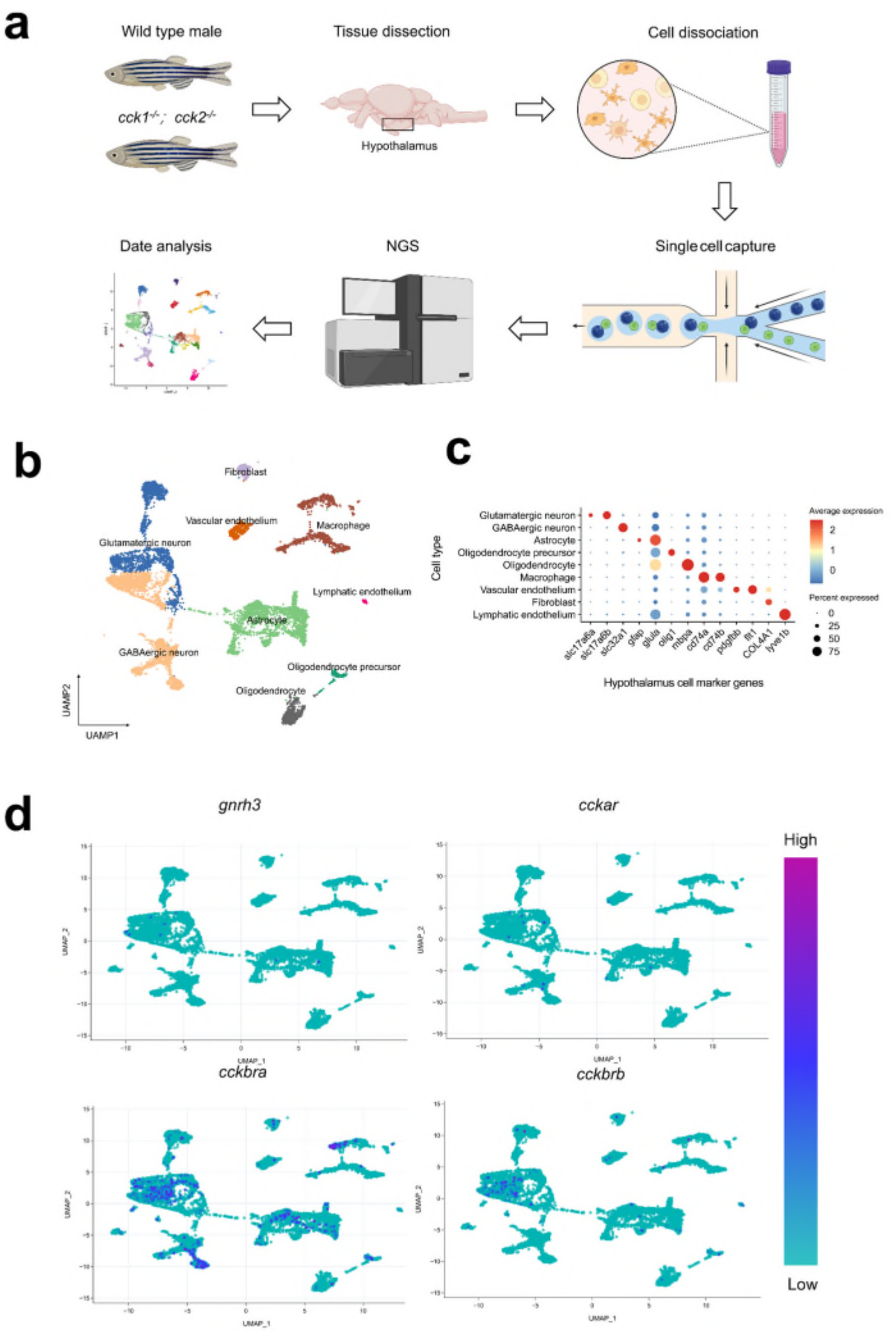

Supplementary Fig. S12 | Single-cell RNA sequencing analysis of the zebrafish hypothalamus. a Schematic diagram of zebrafish hypothalamus single-cell RNA sequencing (scRNA-seq) workflow. **b** UMAP visualization of hypothalamic cells, revealing nine major cell clusters. **c** Dot plot showing expression patterns of established marker genes used to annotate cell types. **d** UMAP expression distribution of *cckar*, *cckbra*, *cckbrb*, and *gnrh3* genes in zebrafish hypothalamic cells.

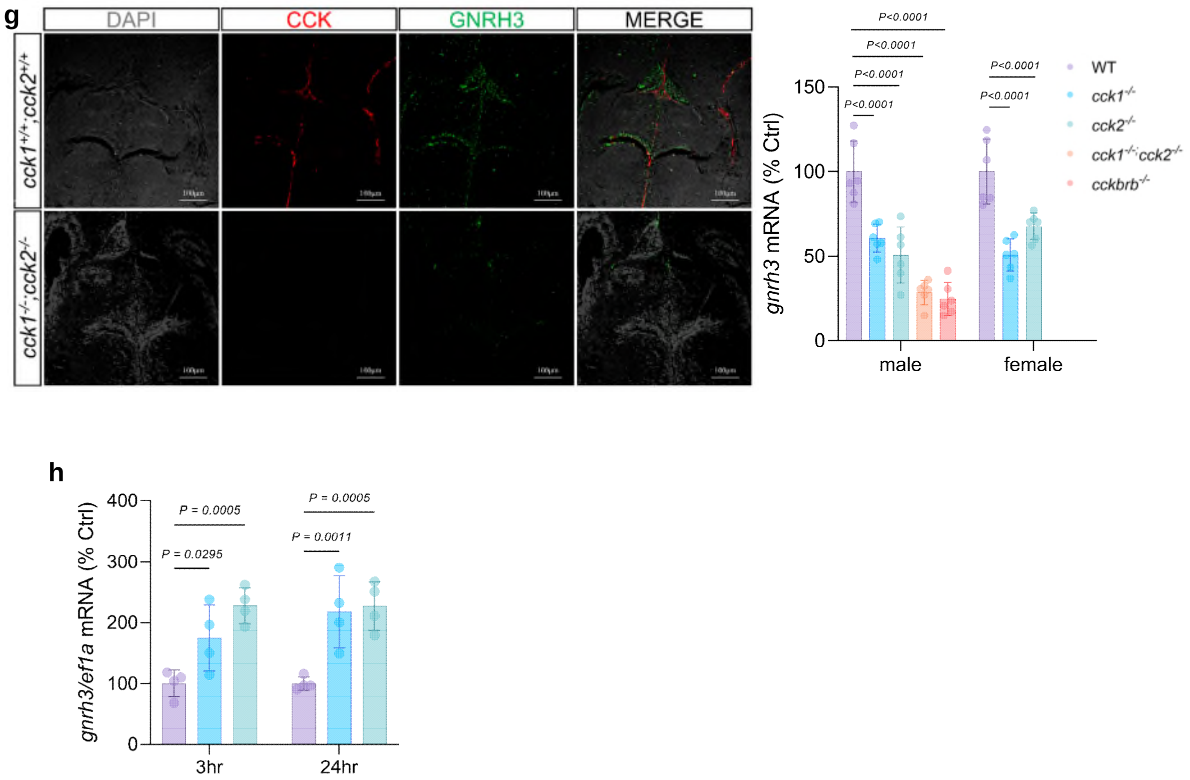

Fig. 3g, h Analysis of the upstream hypothalamic *gnrh3* response. **g** Hypothalamic *gnrh3* expression in CCK ligand and receptor knockout models (*n*= 6). **h** *gnrh3* mRNA levels in hypothalamic cells treated with CCK1 or CCK2 for 3 hours and 24 hours (*n*= 4).

7. L172: ScRNA-seq analysis revealed a considerable subset of pituitary cells expressing both fshb and lhb. This finding should be interpreted with caution. Firstly, the data presented in Figure 1a do not demonstrate co-localization of LH and FSH at the protein level. Secondly, multiple studies in fish, including zebrafish, have established that FSH and LH are synthesized in two distinct populations of pituitary cells, in contrast to the co-expression observed in mammals. Therefore, the actual identity of cells co-expressing lhb and fshb requires further validation. Until such confirmation is available, it is premature to use evidence of cckbrb expression in these cells to support the argument for direct CCK regulation of LH. Moreover, there is no current evidence indicating co-expression of cckbrb specifically within lhb-expressing cells. The previous study by Hollander-Cohen et al. showed little or no expression of cckbra in the LH cells and no expression of cckbrb in the pituitary, which disagrees with the finding of the present study. The authors should carefully and thoroughly address this issue.

Response to Comment 7:

We appreciate the reviewer's careful scrutiny of the *fshb* and *lhb* co-expressing cells. We fully acknowledge the historical view in teleosts describing FSH and LH cells as distinct populations. However, we provide decisive evidence that the co-expressing population identified in our study is a genuine biological entity.

Crucially, the existence of such dual-hormone expression is robustly supported by the very recent study by Tanaka et al. (Endocrinology, 2024). In their single-cell analysis, they notably observed that LH cells partially express *fshb* transcripts, and FSH cells partially express *lhb* (Table R1). Furthermore, they confirmed this co-expression at the protein level using immunohistochemistry, providing strong external validation for the phenomenon (Fig. R4). Building on this, our high-resolution scRNA-seq trajectory analysis places these co-expressing cells at a specific developmental branch point, leading us to define them as "gonadotrope progenitors" (progonadotropes) (Fig. 3d). To independently verify this at the protein level in our own samples, we have provided clearer, high-magnification confocal microscopy images in the revised Fig. 3a. These optimized images unambiguously demonstrate the co-localization of LH and FSH proteins *in vivo*.

Regarding the reviewer's concern about the lack of evidence for receptor expression in LH cells, our new data resolves the apparent discrepancy with the literature. We note that Hollander-Cohen et al. previously identified the pituitary CCK receptor (which they termed *cckbra*, but corresponds to the same *cckbrb* locus we studied) and localized it specifically to FSH cells. Our findings refine this observation rather than contradict it. Our scRNA-seq and FISH/IHC data confirm that *cckbrb* is indeed enriched in the FSH cell lineage, but specifically within the subpopulation that co-expresses LH (Fig. 3d;

Supplementary Fig. S15). Since these cells express high levels of *fshb*, they were classified simply as "FSH cells" in the Hollander-Cohen study. However, our data reveals that these receptor-positive cells also actively produce LH. Therefore, the receptor is indeed present on a specific subset of LH-producing cells (the dual-hormone progenitors), providing the necessary structural basis for the direct regulation of LH by CCK.

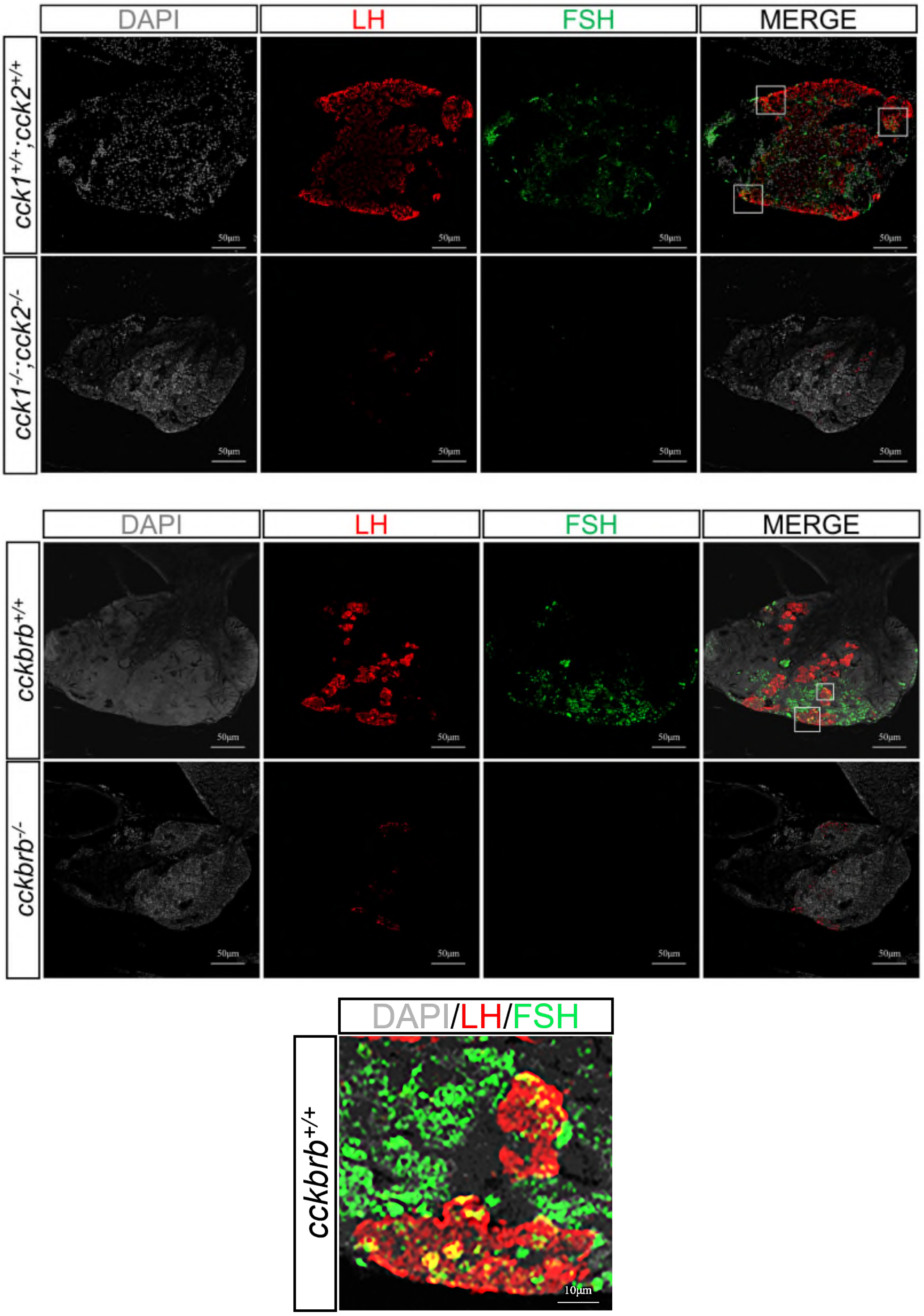

Fig. 3a Representative immunohistochemical staining of LH and FSH proteins in the pituitary of WT, *cck1*$^{-/-}$;*cck2*$^{-/-}$, and *cckbrb*$^{-/-}$ zebrafish.

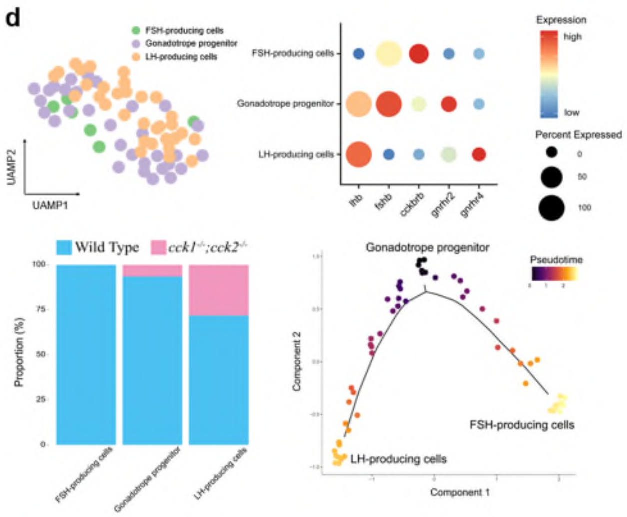

Fig. 3d Sub-clustering and trajectory analysis of the gonadotrope cell population.

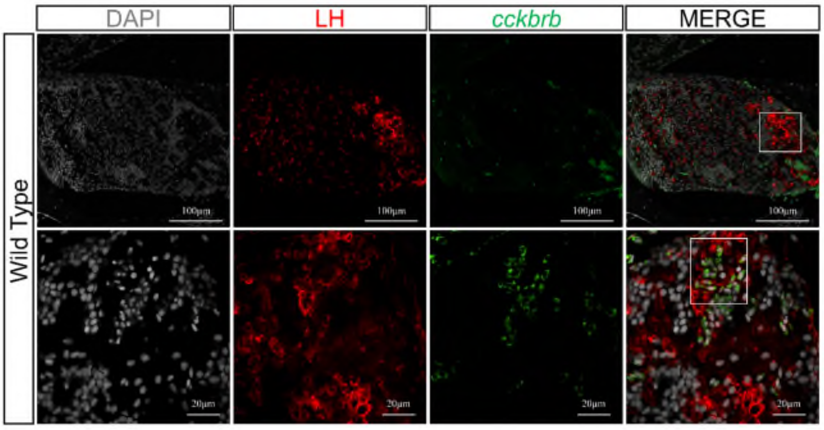

Supplementary Fig. 15 | Co-localization of *cckbrb* mRNA and LH protein in the adult male zebrafish pituitary gland. *cckbrb* mRNA was detected using a fluorescent probe (via Fluorescence *In Situ* Hybridization), and LH protein was visualized via immunohistochemistry (IHC).

Table R1. Single-cell RNA sequencing *lhb* and *fshb* transcript levels in LH and FSH gonadotropes (Tanaka et al., 2024).

[Table Redacted]

[Figure Redacted]

Figure R4. Pituitaries were subjected to double labeling in situ hybridization. *lhb* mRNA (red), *fshb* mRNA (green), merged image to analyze colocalization of *lhb* and *fshb*. (Tanaka et al., 2024).

8. L178-180: Concluding that CCK promotes gonadal maturation and ovulation through its regulation of LH (either directly or indirectly) is premature, as this inference is based solely on their common ability to increase ovulation rates following injection. It is important to note that numerous hormones and factors have demonstrated similar effects, and we cannot conclude that they mediate actions of each other.

Response to Comment 8:

We thank the reviewer for this insightful comment. We fully agree that the shared ability of CCK and LH to induce ovulation, when considered in isolation, is correlative and does not definitively prove that CCK acts exclusively via LH, given the multitude of factors influencing ovulation. In direct response to this point, we have tempered the causal language in the Result and Discussion to avoid overinterpretation.

Revision in the Manuscript:

Result

"Functionally, this dual regulation is physiologically relevant, as injection of either CCK or LH significantly restored ovulation rates in female zebrafish (Fig. 3k)." (Line 202-203)

Discussion

"Functionally, the physiological relevance of this regulation is confirmed by the effective induction of ovulation in female zebrafish following exogenous administration of either CCK or LH."(Line 427-430)

However, we wish to clarify that this functional readout is integrated within a much broader mechanistic framework in our study. Our genetic evidence shows that *cck* and *cckbr* mutants exhibit a severe reduction in pituitary LH content and *lhb* expression (Fig. 3a, b), confirming that endogenous CCK is required for normal LH levels. Furthermore, our mechanistic data demonstrates that CCK stimulates *lhb* transcription both directly (Fig. 3i) and indirectly via hypothalamic *gnrh3* (Fig. 3h). Consequently, the injection experiment (Fig. 3k) serves as a critical functional validation: it demonstrates that restoring CCK signaling—which we have proven drives LH synthesis—is sufficient to trigger the physiological endpoint (ovulation) that is lost in the mutants. Collectively, this multi-layered evidence strongly supports the model that CCK's regulation of the LH axis is a significant contributor to its pro-ovulatory action.

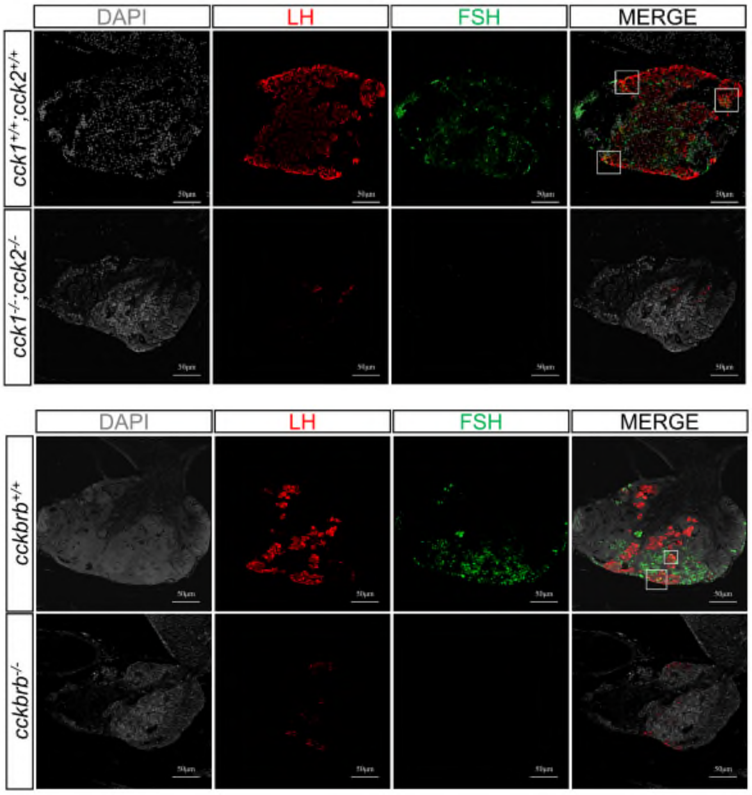

Fig. 3a Representative immunohistochemical staining of LH and FSH proteins in the pituitary of WT, *cck1*$^{-/-}$;*cck2*$^{-/-}$, and *cckbrb*$^{-/-}$ zebrafish.

b

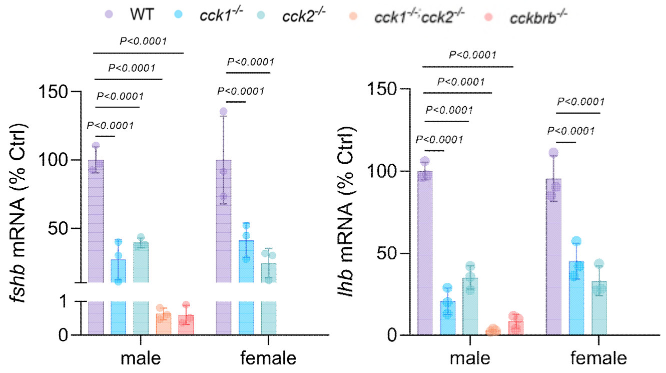

Fig. 3b Relative mRNA expression of *lhb* and *fshb* in pituitary tissue from different knockout models compared to WT. Data are derived from n= 4 independent biological replicates, where each replicate consists of a pool of 3 pituitaries.

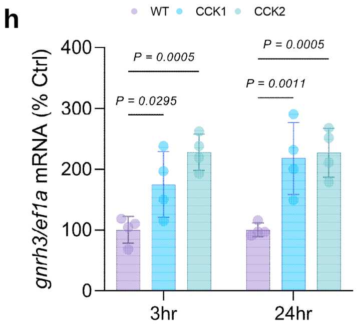

Fig. 3h *gnrh3* mRNA levels in hypothalamic cells treated with CCK1 or CCK2 for 3 hours and 24 hours (*n*= 4).

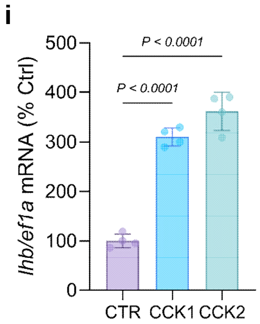

Fig. 3i *lhb* mRNA levels in primary pituitary cells treated with CCK1 or CCK2 for 24 hours.

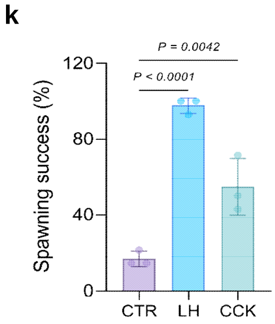

Fig. 3k Functional assessment of ovulation success rate (%) in female zebrafish 24 hours after i.p. injection of LH or CCK (*n*= 14 biologically independent animals from 3 independent experiments).

9. L344-345: To demonstrate that CCK promotes gonadal development through FSH, the best way would be using fshb mutant.

Response to Comment 9:

We thank the reviewer for this excellent suggestion. We fully agree that utilizing an *fshb* mutant is the definitive genetic approach to confirm that CCK promotes gonadal development specifically through FSH signaling. In direct response to this comment, we performed new long-term dietary supplementation experiments using both Wild-type (WT) and *fshb*$^{-/-}$ mutant zebrafish to rigorously test this hypothesis.

To ensure the validity of our approach, we first confirmed that our oral delivery system was effective: measurements taken 1.5 hours post-feeding with a CCK-supplemented diet showed significantly elevated plasma CCK levels and a robust induction of pituitary *fshb* mRNA expression (Supplementary Fig. S9). We then compared the long-term effects of this validated CCK feeding regimen. In wild-type fish, CCK supplementation significantly promoted ovarian and testicular development, evidenced by increased gonadosomatic index (GSI) and advanced histological maturation. Crucially, however, the same CCK treatment failed to rescue the gonadal defects or increase GSI in *fshb*$^{-/-}$ mutants. The complete inability of CCK to promote gonadal development in the absence of FSH provides conclusive genetic proof that FSH is the indispensable downstream effector for this specific function. These new results are presented in Supplementary Fig. S10, strengthening the mechanistic link between CCK and FSH-dependent gametogenesis.

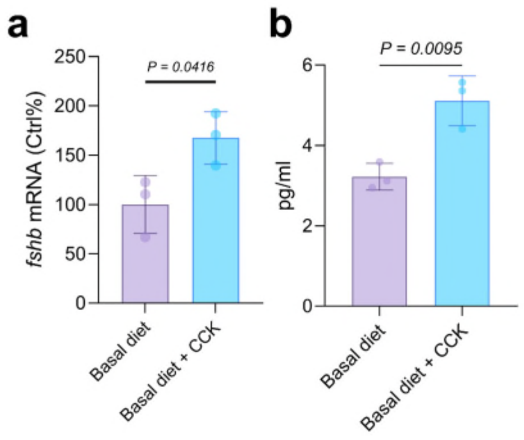

Supplementary Fig. S9 | Confirmation of CCK feeding efficacy and resulting impact on pituitary *fshb* expression. The figure confirms the effective absorption of dietary CCK and its subsequent biological effect, measured 1.5 hours post-feeding with a CCK-supplemented diet versus a normal diet. **a** Relative mRNA expression level of *fshb* in the pituitary gland (determined by qPCR). Data are presented as mean±sem for *n*= 3 independent replicates. Each CCK-fed replicate is a pool of four pituitaries; each normal diet replicate is one pituitary. **b** Plasma CCK concentration (determined by ELISA). Data are presented as mean±sem for *n*= 3 independent replicates. Each replicate is a pool of blood from three individual fish.

Editorial Note: Supplementary Fig. S10a in this Peer Review File is reproduced with permission from BioRender, Created in BioRender. Li, H. (2026) https://BioRender.com/v7qg9n0

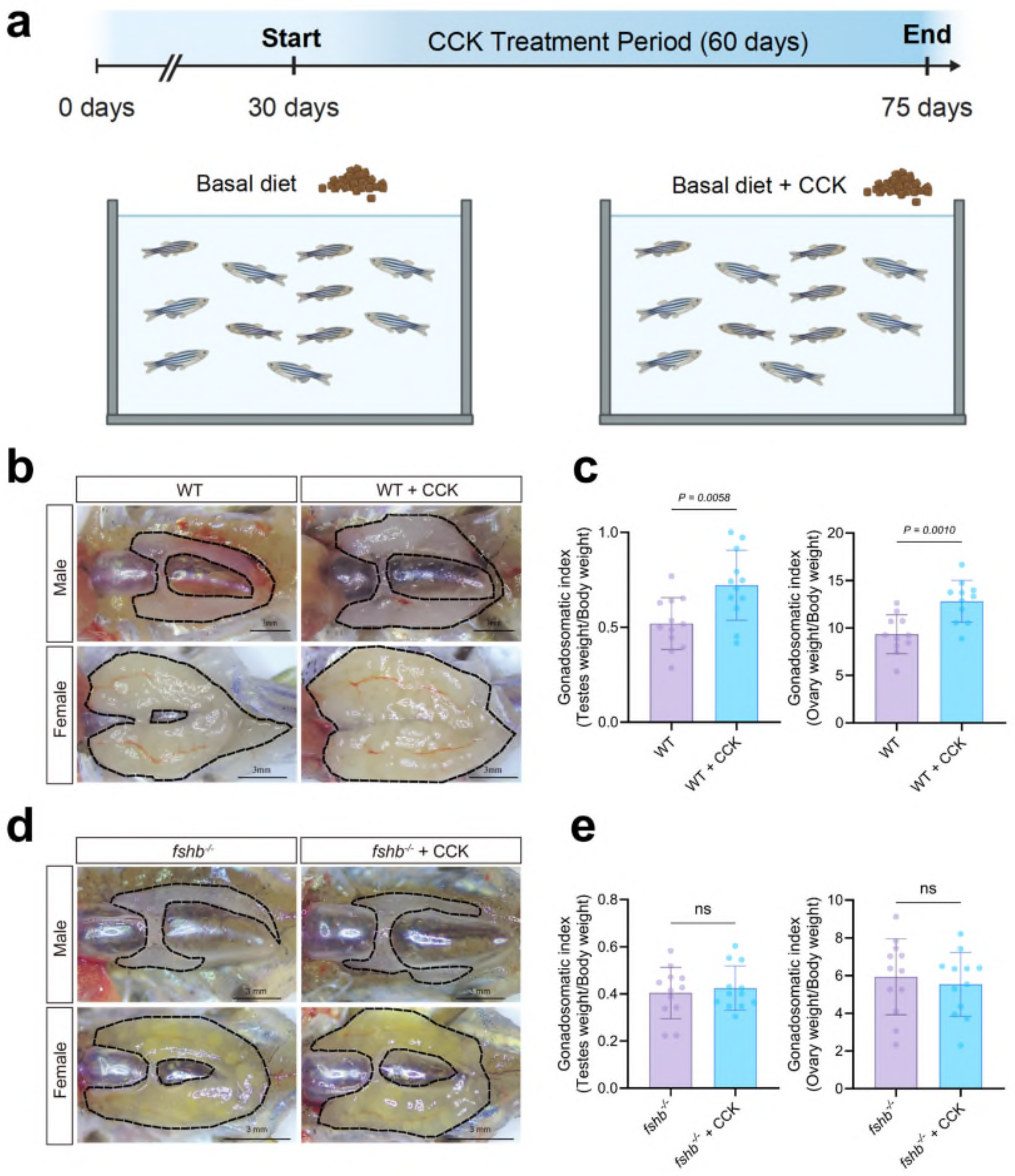

Supplementary Fig. S10 | Effects of CCK-containing diet on gonad development in wild-type and *fshb*$^{-/-}$ zebrafish. a, Schematic diagram of the experimental timeline. Fish were fed the CCK diet for 60 days (from 30 to 75 dpf). **b, d**, Representative images of gonad morphology in Wild-type (WT) (**b**) and *fshb*$^{-/-}$ (**d**) zebrafish after the feeding period. **c, e**, Gonadosomatic Index (GSI) comparison in WT (**c**) and *fshb*$^{-/-}$ (**e**) male and female fish. Data are presented as mean ± s.e.m. for *n*= 12 independent fish per group.

10. L369: This statement "GnRH3 is the primary factor inducing LH expression in zebrafish" may not be true as recent genetic studies have rejected the involvement of GnRH system in controlling gonadotropin biosynthesis.

Response to Comment 10:

We thank the reviewer for this important correction regarding the characterization of GnRH3. We acknowledge that the functional primacy of GnRH in teleost reproduction has been re-evaluated in light of recent genetic studies showing mild phenotypes in knockout models. We agree that our original statement was overly definitive given this context.

Accordingly, we have revised the text in Discussion to more accurately reflect the current consensus. "Additionally, CCK operates via an indirect, GnRH3-mediated route, significantly upregulating hypothalamic *gnrh3* expression "(Line 425-427)

Furthermore, we have integrated this revised view into our proposed model of multimodal control. Rather than claiming GnRH3 is the sole driver, we posit that CCK acts as an "upper-level regulator" that co-activates the canonical GnRH3 pathway alongside a parallel direct pituitary pathway to ensure robust LH synthesis. This nuanced framing is strongly supported by our new functional evidence, which demonstrates that GnRH3 treatment directly induces *lhb* expression in pituitary cells *in vitro* (Supplementary Fig. S13). This confirms that the GnRH3-LH axis remains a functional and potent component of the regulatory network recruited by CCK, even if it operates within a system characterized by evolutionary redundancy.

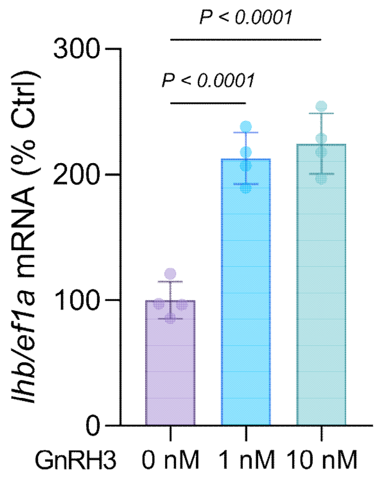

Supplementary Fig. S13 | GnRH3 stimulates *lhb* expression in pituitary cells *in vitro*. qPCR analysis of relative *lhb* mRNA expression in primary cultured zebrafish pituitary cells treated with GnRH3 for 24 hours. Data are presented as mean ± s.d. for *n*= 4 independent biological replicates. Expression levels were normalized to *ef1a*.

Editorial Note: Supplementary Fig. S14a in this Peer Review File is reproduced with permission from BioRender, Created in BioRender. Li, H. (2026) https://BioRender.com/hgl9icn

11. L375-377: The involvement of GnRH3 in CCK actions should be tested in gnrh3 mutant.

Response to Comment 11:

We thank the reviewer for reinforcing this point. This suggestion is closely related to the issue raised in **Comment 6**.

As detailed in our response to Comment 6, we concluded that a stable genetic knockout of *gnrh3* would likely yield ambiguous results due to developmental compensation and the redundancy of the neuroendocrine system (where the direct CCK-LH pathway would mask the loss of the indirect pathway).

To provide a more definitive and mechanistically precise answer, we employed an acute functional blockade strategy. Using a hypothalamic-pituitary co-culture system, we demonstrated that blocking GnRH3 signaling with a neutralizing antibody significantly attenuates CCK-induced *lhb* expression (Supplementary Fig. S14). This result functionally isolates the GnRH3-dependent component of CCK's action, confirming that GnRH3 is indeed a necessary mediator for the full effect of CCK on LH synthesis, without the confounding variables inherent in a permanent knockout model.

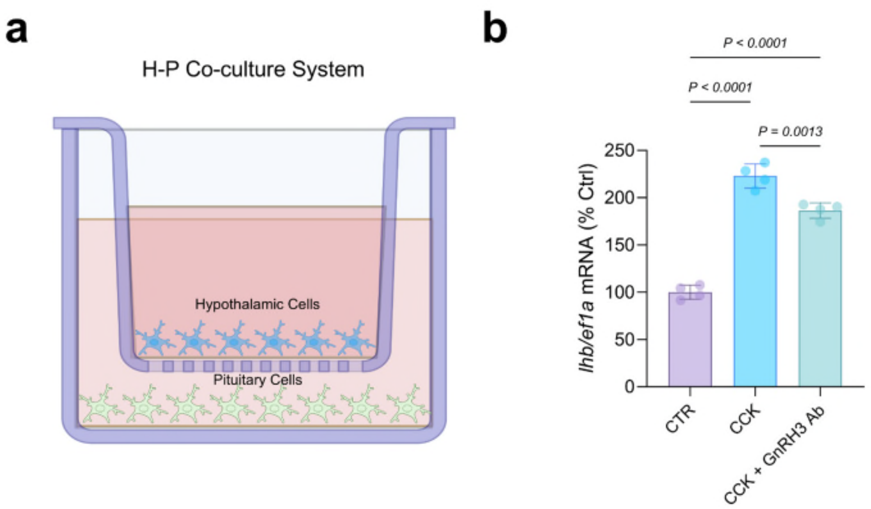

Supplementary Fig. S14 | Hypothalamic-Pituitary (H-P) co-culture system and functional assessment of dual CCK regulation. a Schematic diagram of the H-P co-culture system. Hypothalamic cells are cultured on the transwell insert membrane, and pituitary cells are cultured in the lower chamber, facilitating the transfer of soluble factors. **b** qPCR analysis of *lhb* mRNA expression in pituitary cells from the co-culture system following treatment with Control (Ctrl), CCK only (CCK), and CCK with GnRH3 blocking antibody (CCK + GnRH3 Ab). Data are presented as mean ± s.e.m. (standard error of the mean) for *n*= 4 independent experiments.

12. The existence and mechanism of CCK-FSH and GnRH-LH axes has been reported recently in both zebrafish and medaka. However, there is a complete lack of comparative discussions about these earlier studies, especially the study in medaka story, in the present manuscript.

Response to Comment 12:

We acknowledge that our original discussion did not sufficiently contextualize our findings against these pivotal studies. To address this comprehensively, we have extensively revised the Discussion sections to explicitly cite Hollander-Cohen et al. and Uehara et al. as the foundational studies establishing the CCK-FSH axis. In the revised text, we now clearly delineate between results that corroborate existing models (CCK-FSH) and those that advance the field (CCK-LH and CCK-Gonad/Soma). Furthermore, we have added a dedicated section in the Discussion to position our findings within the current landscape. This section highlights that while the FSH-RH role is conserved, the LH-RH function and the direct, early gonadal regulation we report represent a major expansion of CCK's known neuroendocrine repertoire. We have revised the Discussion section to explicitly compare our work with recent literature and highlight our novel contributions:

Revised Discussion Excerpt 1: Confirmation of FSH-RH Role

"Following gonadal differentiation, zebrafish undergo an extended developmental phase characterized by follicle transition from Stage IB to Stage II, marking female puberty onset[40, 41]. where FSH plays a central role. Our observation that loss of CCK signaling leads to a severe reduction in *fshb* synthesis, which is rescuable by exogenous CCK, aligns robustly with recent breakthroughs in the field. Specifically, seminal studies in medaka[8] and zebrafish[9] have identified CCK as a potent FSH-RH. Our data, derived from comprehensive genetic knockout models, provide independent confirmation of this conserved physiological axis, reinforcing the consensus that CCK directly modulates FSH output to drive gametogenesis in teleosts. Evolutionarily, teleosts exhibit a direct CCK-FSH regulatory axis, wherein FSH-producing gonadotropes express functional CCKRs[8,42,43]. This architecture allows the satiety hormone CCK to directly modulate FSH output, representing a key adaptation in broadcast spawners like zebrafish that require sustained energy investment into oogenesis. In contrast, mammals exhibit a different strategy: FSH and LH are co-produced by the same gonadotropes, which lack CCKRs, implying that FSH regulation in these groups is indirect and likely mediated through upstream hypothalamic signals[44-46]."

Revised Discussion Excerpt 2: Confirmation of LH-RH Role

"Upon completion of gametogenesis, the gonads enter the final maturation stage, during which LH plays a critical role in promoting oocyte maturation and ovulation. In *lhb*$^{-/-}$

zebrafish, oocyte growth proceeds normally, but final maturation and ovulation are significantly impaired[11, 12, 47]. Building upon the established role of CCK as a major FSH-RH, our study reveals a significant expansion of its neuroendocrine repertoire: CCK also functions as a critical regulator of LH in zebrafish. Both *cck1*$^{-/-}$;*cck2*$^{-/-}$ and *cckbrb*$^{-/-}$ mutants exhibited markedly reduced pituitary LH expression, implicating CCK in LH synthesis and secretion. The apparent discrepancy with earlier studies concluding a specific FSH-RH role likely stems from their primary focus on establishing that novel function, and from our identification of a specific cellular substrate: a subpopulation of pituitary gonadotropes that co-express *fshb* and *lhb* transcripts[43] and are enriched for the *cckbrb* receptor, providing a basis for dual regulation.

Mechanistically, CCK regulates LH through a dual pathway. It directly stimulates *lhb* expression in pituitary cells, an action supported by the presence of CCKBRb in cells co-expressing FSH and LH. Additionally, CCK operates via an indirect, GnRH3-mediated route, significantly upregulating hypothalamic *gnrh3* expression. This dual mechanism ensures robust LH output. Functionally, the physiological relevance of this regulation is confirmed by the effective induction of ovulation in female zebrafish following exogenous administration of either CCK or LH. Therefore, our findings extend the existing paradigm, positioning CCK not as a solo FSH-RH, but as a dual gonadotropin-releasing factor that integrates the control of gametogenesis (via FSH) and final maturation/ovulation (via LH), underscoring its role as a master coordinator within the teleost reproductive neuroendocrine system."

13. It is concluded in the manuscript that CCK promotes PGC proliferation and facilitate meiotic division, independently of gonadotropins. However, the study did not provide any information about where the CCK molecule comes from; in particular, the results from the present study clearly show that CCK genes are expressed in multiple organs including the gonads. The working model indicates that CCK comes from the hypothalamus as a neuroendocrine hormone (Figure 6); however, this needs to be validated by evidence.

Response to Comment 13:

We thank the reviewer for this critical observation regarding the anatomical source of CCK. We agree that attributing early gonadal regulation to a hypothalamic neuroendocrine source was premature without spatial evidence. To definitively identify the source of CCK during these early developmental stages, we performed high-resolution immunofluorescence co-staining experiments. Our new data supports a local, paracrine mechanism: at the migration stage (2–5 dpf), CCK protein expression was observed specifically along the migration route of VASA-positive PGCs (Fig. 4d); crucially, at the differentiation stage (14 dpf), CCK was specifically localized within gonadal somatic cells surrounding the germ cells (Fig. 4e). While we also detected expected expression in the intestine (serving as an internal positive control), the specific localization within the gonad provides the structural basis for direct paracrine regulation of germ cells.

Based on these findings, we have revised our working model (Fig. 5i) to accurately reflect these distinct sources. The updated schematic no longer attributes early regulation to the hypothalamus. Instead, it depicts a two-phase control system: (1) Early Stages: Local CCK signaling (derived from gonadal somatic cells and the migratory niche) drives PGC proliferation and meiotic initiation via gonadotropin-independent mechanisms; (2) Late Stages: Hypothalamic/Pituitary CCK signaling drives gonadotropin synthesis and ovulation via gonadotropin-dependent mechanisms. This revision resolves the ambiguity and aligns the model strictly with our experimental evidence.

d

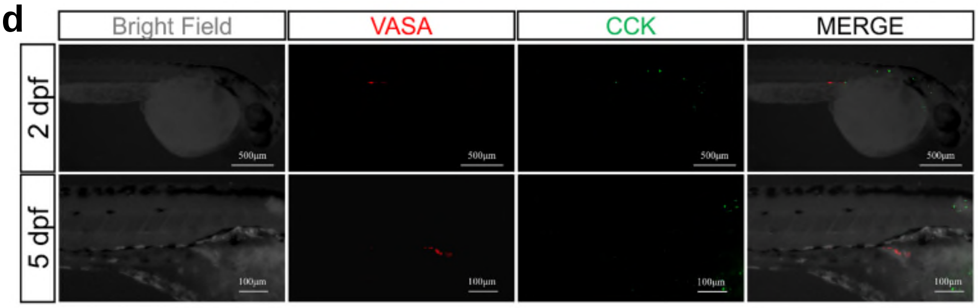

Fig. 4d Whole-mount immunofluorescence co-staining of VASA (germ cell marker, red) and CCK (green) in zebrafish larvae at 2 dpf and 5 dpf. Merged images show the spatial relationship between CCK-expressing cells and migrating PGCs.

Editorial Note: Fig. 5i in this Peer Review File is reproduced with permission from BioRender, Created in BioRender. Li, H. (2026) https://BioRender.com/o68vxgm

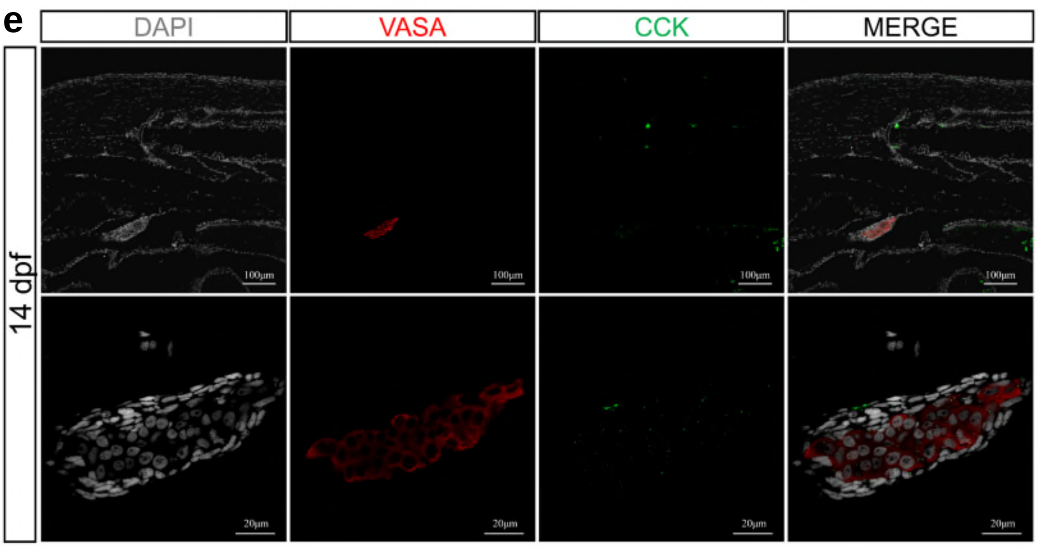

Fig. 4e Immunofluorescence of CCK (green) and VASA (red) in 14 dpf gonads reveals C CK expression in somatic cells surrounding germ cells.

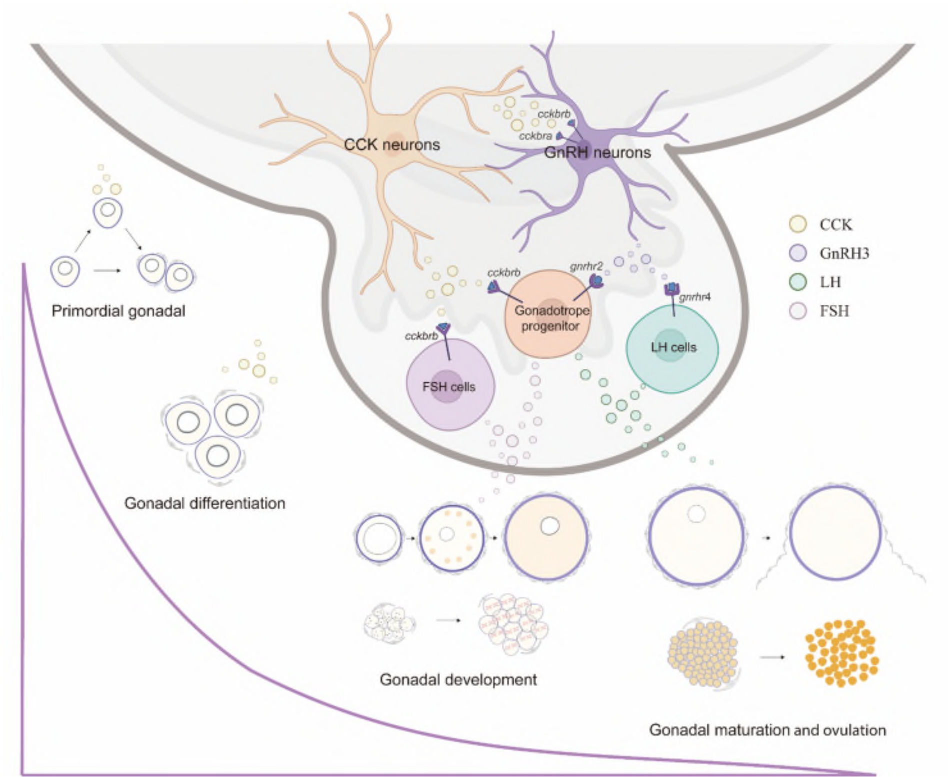

Fig. 5i Schematic diagram depicting the stage-specific regulatory network of CCK signaling during gonadal development. The model illustrates CCK's involvement in primordial germ cell (PGC) proliferation during early formation, the maintenance of somatic cell integrity and meiotic initiation during differentiation, and the regulation of pituitary FSH and LH synthesis (involving both direct pituitary action and indirect hypothalamic *gnrh3* modulation) to support gametogenesis and ovulation.

14. To demonstrate that CCK can directly promote PGC proliferation, the authors need to provide two pieces of critical evidence: first, the expression of CCK receptor(s) in PGCs, and second, in vitro effects of CCK on cultured PGCs. The second one may be technically challenging, but it is worth trying. As a neuroendocrine hormone, CCK may indirectly influence PGC proliferation and migration via other endocrine and paracrine pathways.

Response to Comment 14:

We thank the reviewer for this rigorous suggestion regarding the distinction between direct and indirect effects on PGCs. To address the question of direct action, we performed a comprehensive analysis of the single-cell developmental trajectory dataset from Farrell et al. (2018) (*Science*, DOI: 10.1126/science.aar3131), covering stages from "High" to "6-somite". As shown in Fig. R5, the expression of *cckbrb* within the defined PGC lineage is negligible. Specifically, positive expression was detected in only 3 cells at the 50% epiboly stage and 1 cell at the 90% epiboly stage across the entire dataset. This sporadic and extremely low-level expression strongly argues against a robust direct action of CCK on PGCs during these developmental windows. We also attempted to perform the suggested *in vitro* culture experiment using *piwi1:EGFP* transgenic zebrafish (obtained from the Zebrafish Resource Center) to functionally test this; however, the extremely low number of PGCs prevented the retrieval of sufficient viable cells for a statistically robust proliferation assay, validating the technical challenge noted by the reviewer.

Consequently, our data strongly supports the reviewer's alternative hypothesis that CCK influences PGCs indirectly via the microenvironment. In contrast to the negligible expression in PGCs, we found that CCK is specifically localized to the gonadal somatic cells immediately surrounding the germ cells by 14 dpf (Fig. 4e). Furthermore, *cck* mutants exhibit extensive apoptosis specifically in these somatic cells (Fig. 5f), whereas gonadotropin mutants (which maintain normal PGC numbers) do not show this somatic apoptosis. This indicates that CCK acts on the somatic niche to maintain its integrity, which is the prerequisite for PGC survival and proliferation. We have revised the Discussion to explicitly state that CCK's regulation of PGCs is indirect and mediated by the maintenance of the somatic niche.

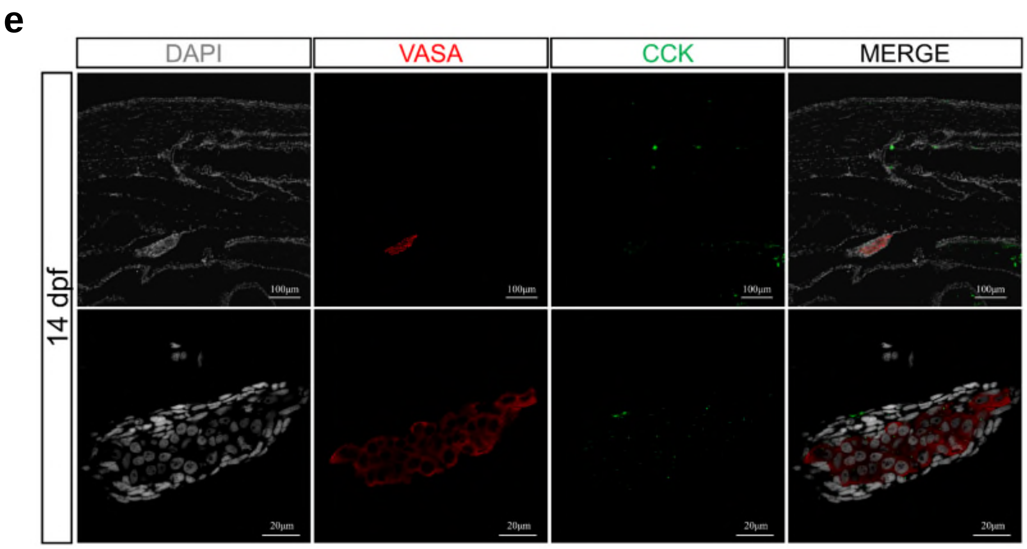

Fig. 4e Immunofluorescence of CCK (green) and VASA (red) in 14 dpf gonads reveals CCK expression in somatic cells surrounding germ cells.

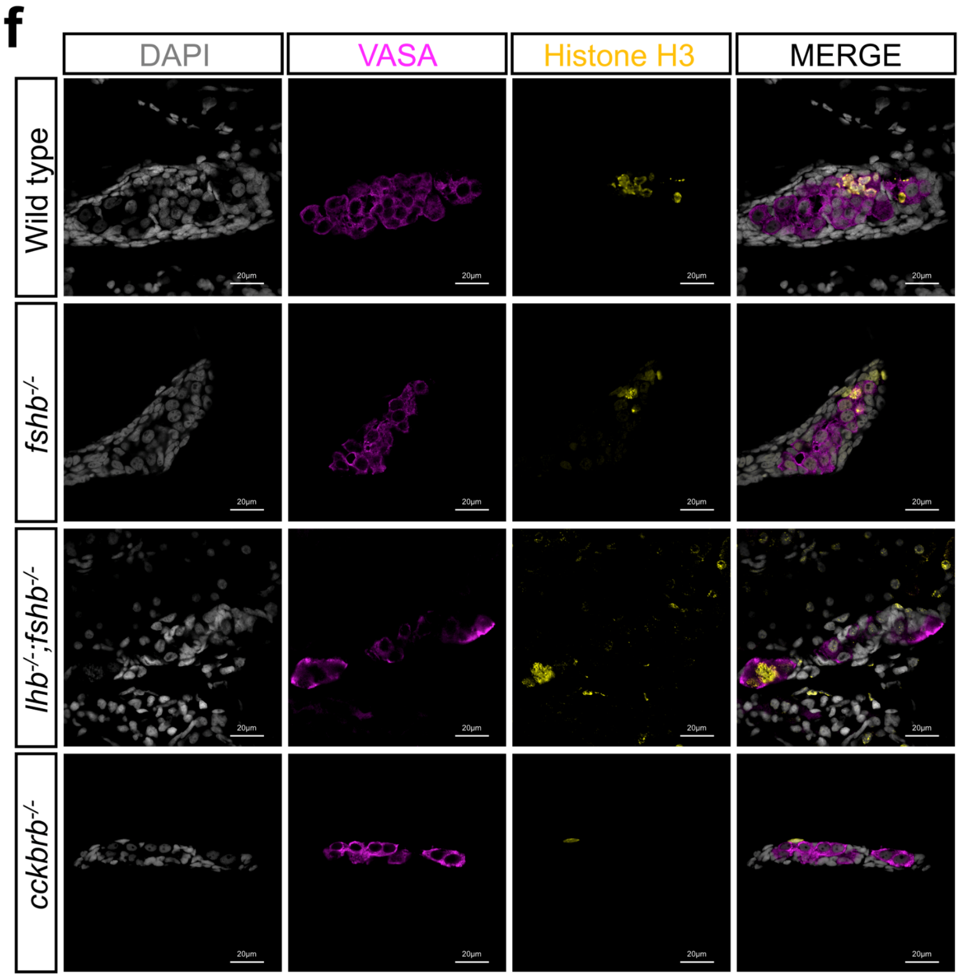

Fig. 4f Immunofluorescence co-expression analysis of germ cells (VASA) and mitotic cells (Histone H3) in gonadal tissue of WT, *fshb*$^{-/-}$, *lhb*$^{-/-}$;*fshb*$^{-/-}$, and *cckbrb*$^{-/-}$ zebrafish at 14 dpf.

[Figure Redacted]

Fig. R5 | Negligible expression of *cckbrb* in the zebrafish PGC lineage during early embryogenesis. a, b Visualization of the single-cell developmental trajectory from Farrell et al. (2018) (Source: https://singlecell.broadinstitute.org/single_cell/study/SCP162). The UMAP plots display cell clusters colored by (**a**) lineage identity (with PGCs indicated) and (**b**) developmental stage (from High to 6-somite). **c**, Expression distribution of *cckbrb* mapped onto the trajectory. The green arrow indicates the PGC cluster. Note that *cckbrb* expression is extremely sparse, detected in only 3 cells at the 50% epiboly stage and 1 cell at the 90% epiboly stage across the entire dataset (Log2 expression values: 1.96, 1.88, 1.54, 0.94). This lack of robust expression in the PGC lineage supports the hypothesis that CCK regulation of PGCs is likely indirect.

15. L382-383: There is no evidence for roles of somatic cell integrity in controlling meiosis.

Response to Comment 15:

We thank the reviewer for this critical comment. We agree that the specific role of gonadal somatic cell integrity in controlling meiosis is a key mechanistic conclusion that requires robust support. To substantiate this link, we provide two lines of compelling new evidence—cellular localization and genetic rescue—that transform this observation from a correlation into a causal mechanism.

Cellular specificity of apoptosis: First, to identify exactly *which* cells were dying, we performed high-resolution co-staining using markers for germ cells (VASA), somatic/Sertoli cells (SOX9a), and apoptosis (TUNEL). As shown in Fig. 5f, *cckbrb* mutants exhibit widespread apoptosis at 14 dpf, the exact developmental window where meiotic initiation fails. Crucially, our detailed analysis reveals that this apoptosis is restricted to the somatic cell population surrounding the germ cells, while the germ cells themselves initially remain viable but arrested. Further characterization at later stages (Fig. 5e) confirms that this cell death specifically affects the interstitial (Leydig) lineage, disrupting the structural and signaling niche required for gametogenesis.

Causal evidence via genetic rescue: To definitively prove that this somatic apoptosis is the *cause* of the meiotic failure (rather than a bystander effect), we performed a functional rescue experiment. We identified that the somatic apoptosis is mediated by the *p53* pathway. By generating *cckbrb*$^{-/-}$;*tp53*$^{-/-}$ double mutants, we successfully blocked this apoptosis. Most importantly, preventing somatic cell death was sufficient to restore meiotic initiation (indicated by the recovery of SYCP3 expression) and rescue gonadal morphology (Fig. 5g, h; Supplementary Fig. 26). This provides direct causal evidence that the integrity of the gonadal somatic niche, maintained by CCK, is an absolute prerequisite for germ cell entry into meiosis. We have revised the Discussion to present this "Somatic Niche Maintenance" model, which is now fully substantiated by our genetic rescue data.

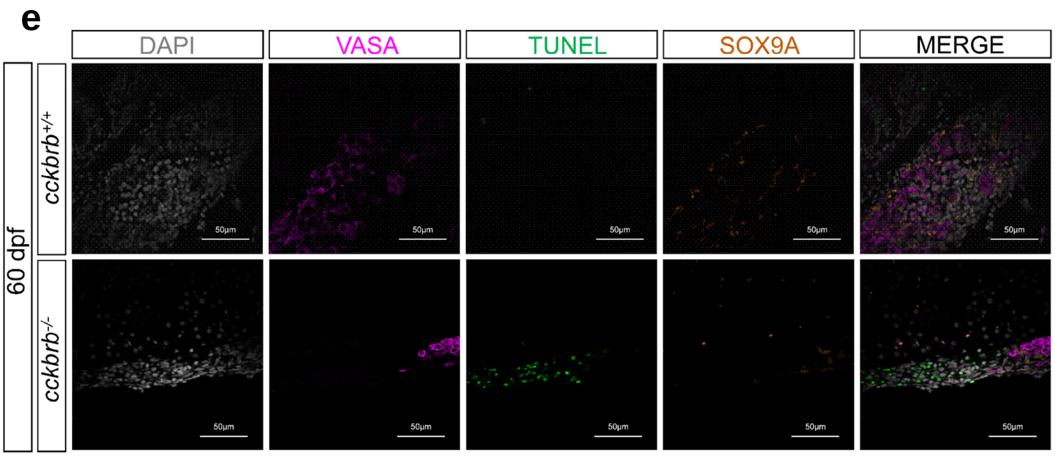

Fig. 5e Co-staining of VASA, TUNEL, and SOX9a in testes of 60 dpf zebrafish.

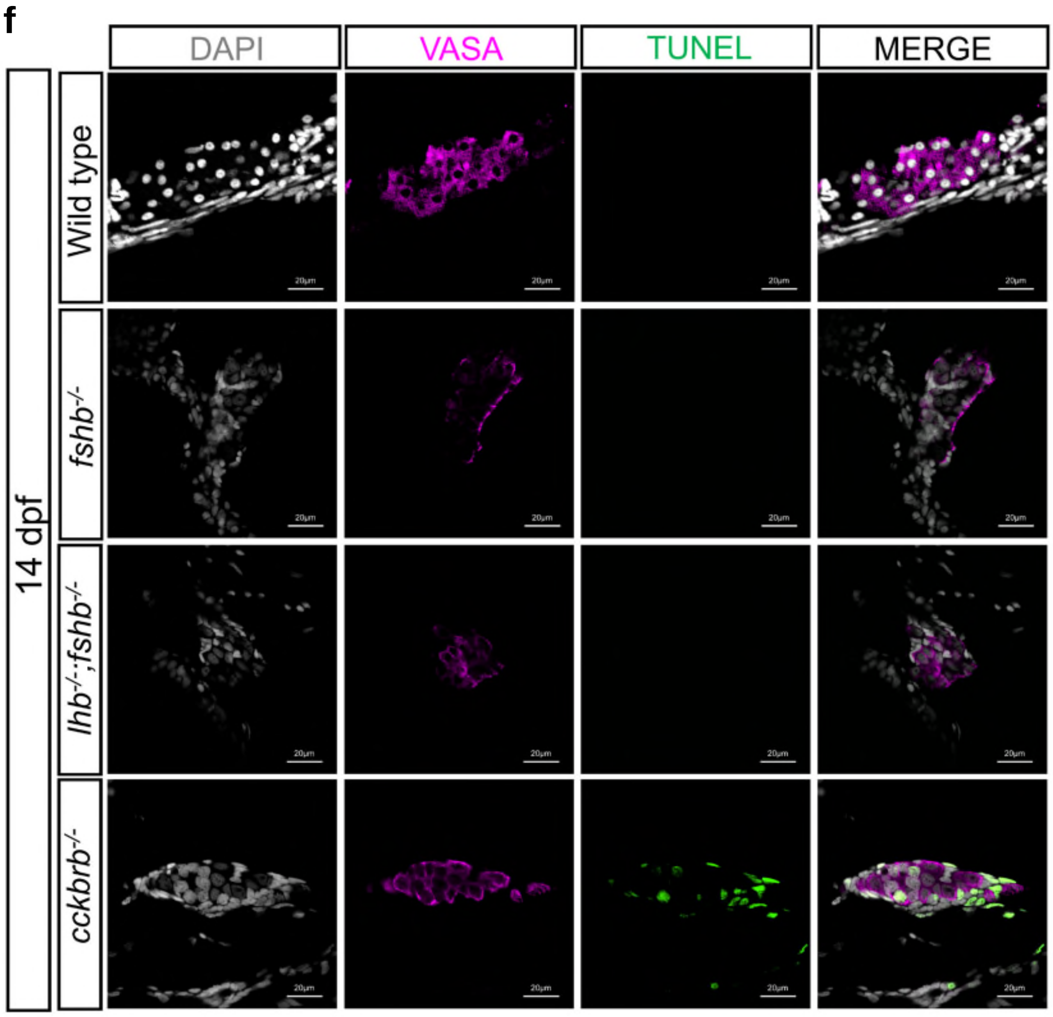

Fig. 5f TUNEL and VASA staining in 14 dpf gonads of WT, *fshb*$^{-/-}$, *lhb*$^{-/-}$;*fshb*$^{-/-}$, and *cckbrb*$^{-/-}$ zebrafish.

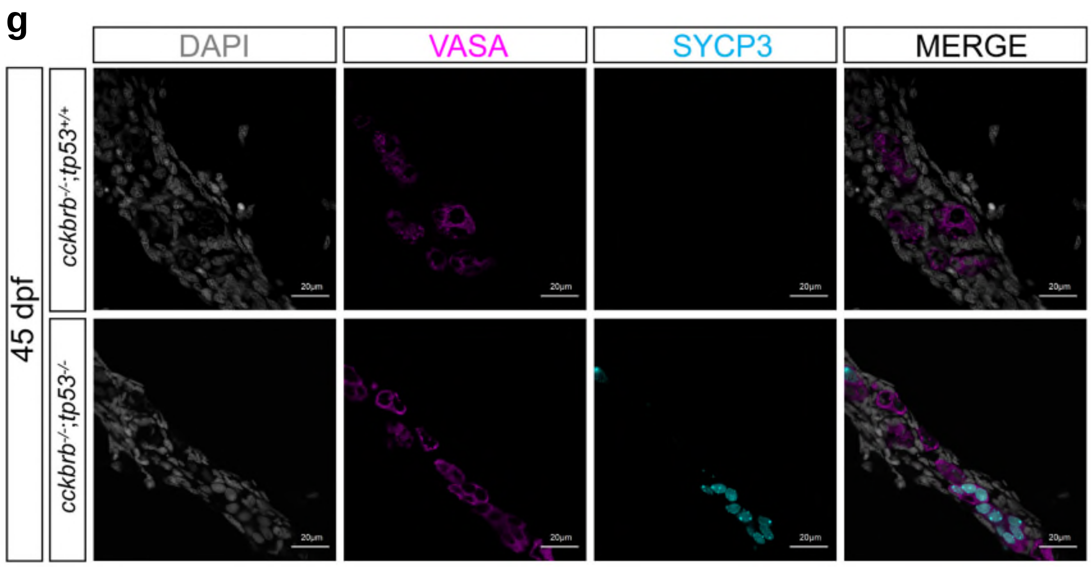

Fig. 5g Immunofluorescence of SYCP3 and VASA in testes at 45 dpf.

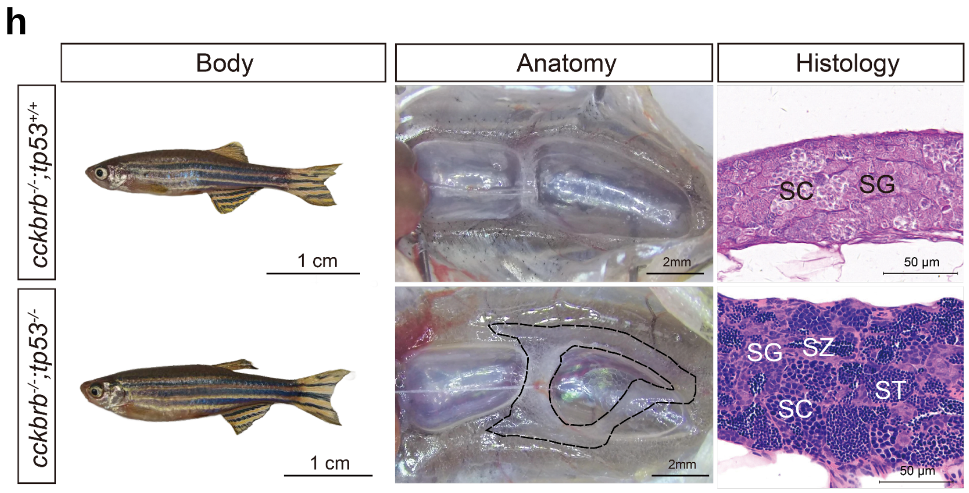

Fig. 5h Gross morphology and histological analysis (H&E) of gonads.

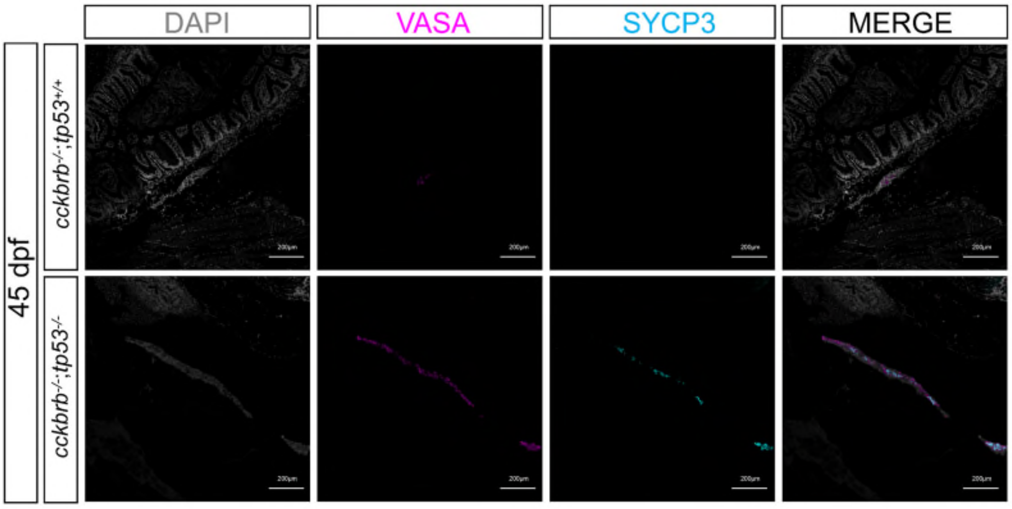

Supplementary Fig. S26 | Analysis of SYCP3 expression in *cckbrb$^{-/-}$;tp53$^{+/+}$* and *cckbrb$^{-/-}$;tp53$^{-/-}$* zebrafish gonads. The figure displays low-magnification Immunofluorescence (IF) staining of SYCP3 protein (a meiotic marker) in gonadal sections. The analysis compares *cckbrb$^{-/-}$;tp53$^{+/+}$*, and *cckbrb$^{-/-}$;tp53$^{-/-}$* zebrafish. Scale bars are indicated on the images.

Other questions:

16. The authors used commercially available antibody to stain for CCK in the hypothalamus. Was the antibody specificity validated by Western blotting?

Response to Comment 16:

We thank the reviewer for this critical question regarding antibody specificity. We fully agree that rigorous validation is essential. In our efforts to address this point via Western blotting, we encountered a technical hurdle specific to the CCK protein: the commercially available antibody is raised against the short, 8-amino-acid CCK-8 peptide. Due to its exceedingly small molecular weight (~1 kDa) and low endogenous levels in tissue lysates, standard Western blotting protocols are often insufficient to resolve a specific band.

Therefore, to provide the most rigorous validation possible, we employed a genetic negative control approach, which is widely considered the "gold standard" for confirming antibody specificity *in situ*. We selected the intestine for this validation assay because it is the site of highest endogenous CCK expression, providing the most stringent test for signal specificity.

As already presented in Fig. 1b of our manuscript, the robust CCK immunoreactive signal present in the Wild-type intestine is completely absent in the *cck1*$^{-/-}$;*cck2*$^{-/-}$ double knockout tissue. This clean genetic validation confirms that the antibody specifically recognizes zebrafish CCK protein and does not exhibit off-target binding, thereby validating its use for identifying CCK-positive neurons in the hypothalamus.

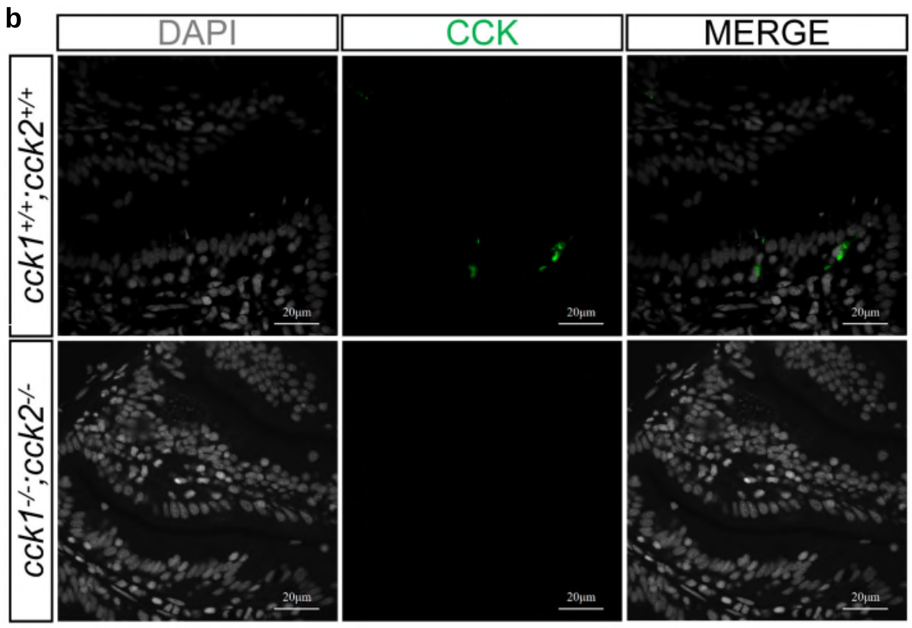

Fig. 1b Immunostaining of CCK protein in the intestine of *cck1*$^{+/+}$;*cck2*$^{+/+}$ and *cck1*$^{-/-}$;*cck2*$^{-/-}$ zebrafish.

17. L108-110: It is stated that CCKBRb exhibited the highest binding affinity for CCK1/2. To evaluate and compare ligand-receptor affinity, one would need to construct a full dose response curve for ED50, which is the critical parameter for comparing binding affinity. However, most dose response curves presented (Fig. 2a) are not complete, making it inappropriate to make conclusions regarding affinity.

Response to Comment 17:

We sincerely thank the reviewer for this critical expert comment regarding the quantitative assessment of ligand-receptor affinity. We fully agree that robust affinity claims must be based on complete, saturated dose-response curves to allow for the precise calculation of EC50 values. We acknowledge that the initial data in Fig. 2a were insufficient for such a definitive conclusion.

In direct response to this point, we performed a completely new set of experiments using a highly sensitive CRE luciferase reporter system (replacing the original NFAT system) to optimize signal detection. We treated cells expressing each zebrafish CCK receptor (CCKAR, CCKBRA, CCKBRb) with a full logarithmic range of CCK1 and CCK2 peptide concentrations.

The results, now presented in the revised Fig. 2a, show complete sigmoidal dose-response curves for the receptor-ligand pairs. Based on these high-quality data, we accurately calculated the EC_{50} values, which are detailed in Supplementary Table S1. These quantitative results robustly support our original observation that CCKBRb exhibits the lowest EC_{50} values (highest affinity) for both CCK1 and CCK2 ligands compared to the other receptor subtypes. We have revised the manuscript text (Results section and Fig. 2a legend) to base our conclusions squarely on these new quantitative EC_{50} comparisons.

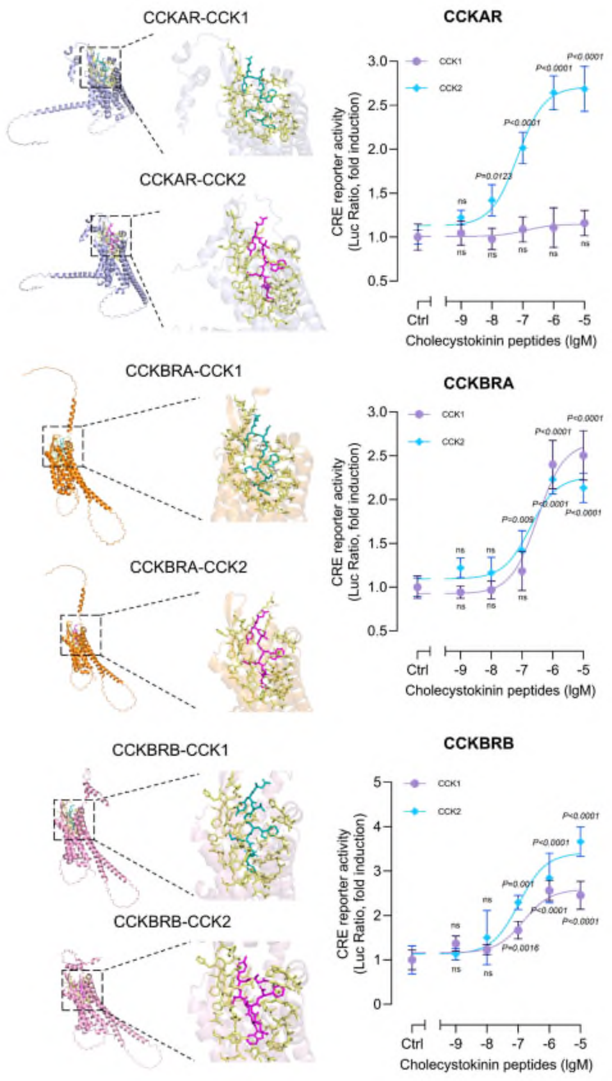

Fig. 2a Ligand-receptor binding analysis. Structural prediction of zebrafish CCK interacting with its receptors (CCKAR, CCKBRA, and CCKBRB) via AlphaFold3 and functional validation using a dual-luciferase reporter assay (*n*= 4).

Supplementary Table S1 | EC_{50} Values for Zebrafish CCK Receptors

Receptor	CCK1 EC_{50} (nM)	CCK2 EC_{50} (nM)
CCKAR	-	68.4 ± 33.1
CCKBRA	240.8 ± 128.1	135.9 ± 108.6
CCKBRB	8.3 ± 5.7	4.9 ± 2.3

The values represent the effective concentration (EC_{50}) of zebrafish CCK1 and CCK2 ligands. Data are presented as the mean ± s.e.m. *n*= 4.

18. L118-119: replace “-“ with commas.

Response to Comment 18:

We thank the reviewer for identifying this error in punctuation. We have replaced the hyphens (“-”) with commas in the revised manuscript.

19. L141-142 and L148: Statistical analysis is needed to make the conclusion “marked reduction”, and “significantly promoted”.

Response to Comment 19:

We thank the reviewer for emphasizing the necessity of robust statistical support for our conclusions. We have addressed this point separately for the single-cell sequencing data and the functional data, as they present different analytical contexts.

Regarding the single-cell RNA-seq data (L141-142): We agree that the term "marked reduction" would ideally be supported by a formal statistical test of cell abundance. However, due to the inherent technical challenge of low cell yield from the *cck1*$^{-/-}$;*cck2*$^{-/-}$ mutant pituitaries, rigorous statistical comparison of cell population sizes is challenging and can be confounded by multiple factors. Therefore, to ensure accuracy and avoid overstatement, we have revised the language from the quantitative "marked reduction" to the more descriptive "clear reduction," which accurately reflects the pronounced visual trend evident in Fig. 3c, d and Supplementary Fig. S7.

Regarding the functional data on ovarian development (L148): We fully agree that the claim "significantly promoted" requires statistical validation. To provide this, we performed an additional quantitative analysis by calculating the Gonadosomatic Index (GSI) for the zebrafish in the dietary CCK supplementation experiment. The results, now included as Supplementary Fig. S10c, demonstrate a statistically significant increase in GSI in the CCK-treated group, which robustly supports our conclusion that CCK promotes ovarian development. We have updated the manuscript text and figures accordingly.

Editorial Note: Supplementary Fig. S10a in this Peer Review File is reproduced with permission from BioRender, Created in BioRender. Li, H. (2026) https://BioRender.com/v7qg9n0

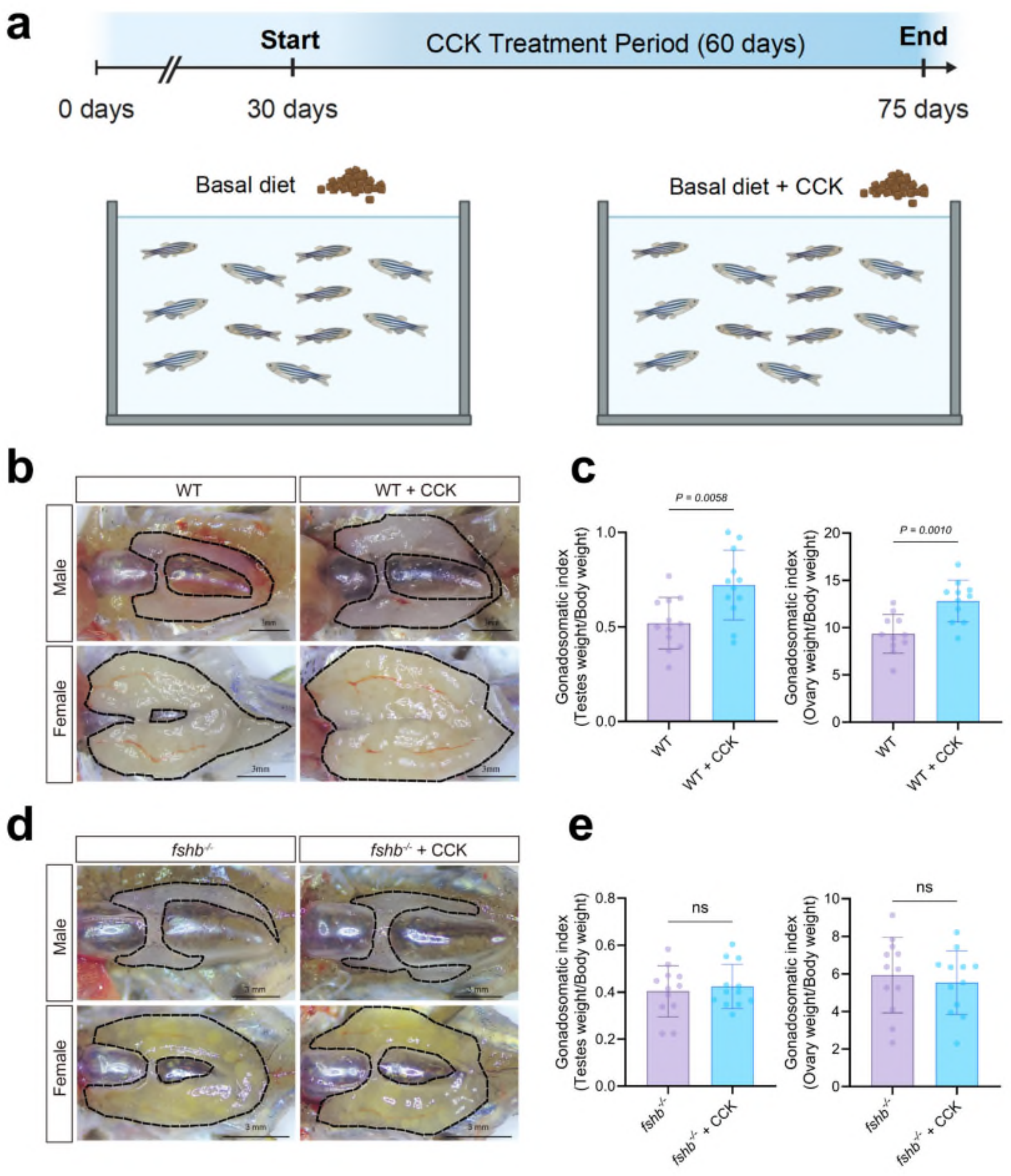

Supplementary Fig. S10 | Effects of CCK-containing diet on gonad development in wild-type and *fshb*$^{-/-}$ zebrafish. a, Schematic diagram of the experimental timeline. Fish were fed the CCK diet for 60 days (from 30 to 75 dpf). **b, d**, Representative images of gonad morphology in Wild-type (WT) (**b**) and *fshb*$^{-/-}$ (**d**) zebrafish after the feeding period. **c, e**, Gonadosomatic Index (GSI) comparison in WT (**c**) and *fshb*$^{-/-}$ (**e**) male and female fish. Data are presented as mean ± s.e.m. for *n*= 12 independent fish per group.

20. L177: What LH was used to induce ovulation? This information is important because it is known that heterologous hormones especially those from mammals may have different receptor specificity and potency in fish species.

Response to Comment 20:

We thank the reviewer for raising this critical point regarding hormone specificity. The source and species origin of the LH used are indeed vital for interpreting the physiological relevance of the ovulation induction experiment.

In our study, the LH administered was recombinant zebrafish Luteinizing Hormone (r-zLh). We specifically selected this homologous protein to ensure high receptor binding affinity and biological activity within the zebrafish model system, thereby avoiding the potential confounding factors of potency and specificity associated with heterologous mammalian hormones.

We have updated the Methods section (Line 658-659) to explicitly state the source, species, and recombinant nature of the LH protein used. We appreciate the reviewer's attention to this important methodological detail.

21. Figure 4b: the label "fshrb-/-" should be "fshb-/-".

Response to Comment 21:

We thank the reviewer for identifying this typographical error. We have corrected the label in Figure 4b from "*fshrb*$^{-/-}$" to the accurate gene name, "*fshb*$^{-/-}$" (Figure 4b).

22. L273: "donadotropin" should be "gonadotropin".

Response to Comment 22:

We appreciate the reviewer pointing out this typographical error. We have corrected "donadotropin" to the accurate term, "gonadotropin", in the revised manuscript.

23. L274: In Figure 1, although the mutant males show deficient testis development, spermatocytes are obvious, indicating meiotic activity. How can the expression of sycp3 be completely missing?

Response to Comment 23:

We thank the reviewer for this insightful observation, which allows us to clarify a crucial aspect of the meiotic arrest phenotype. The reviewer is correct that our histology identifies cells with the morphology of spermatocytes (SC) in the mutants. However, the absence of SYCP3 protein indicates a profound failure at the molecular level of meiosis.

This apparent discrepancy is a recognized hallmark of early meiotic arrest. Cells can be positioned and begin to condense their chromosomes, acquiring a spermatocyte-like morphology, yet fail to execute the core molecular program of meiosis—specifically, the assembly of the synaptonemal complex, for which SYCP3 is an essential structural component.

The phenotype we observe is directly analogous to that reported in *sycp3* null mutants (e.g., Pan et al., 2022), where germ cells also appear as spermatocytes by histology but are molecularly and functionally arrested because they cannot form the synaptonemal complex.

Therefore, our data collectively demonstrate that in the absence of CCK signaling, germ cells initiate differentiation and reach a pre-meiotic/early leptotene stage but cannot progress into active, stable meiosis due to a failure to initiate SYCP3 expression and synaptonemal complex formation. This defines a specific mechanistic role for CCK in activating the core molecular machinery of meiotic prophase I.

24. L279: What does “permanent block” mean? If it indicates no meiotic activity, why are spermatocytes present in the mutant testis (Figure 1)? Without meiosis, gonads should be undifferentiated, then how is sex determined?

Response to Comment 24:

We thank the reviewer for these insightful questions, which allow us to clarify the precise nature of the phenotype and the distinction between sex determination and gametogenesis.

(1) Clarification of "Permanent Block": We apologize for the lack of clarity in our original phrasing. The term "permanent block" was intended to describe the sustained and irreversible arrest of germ cell development specifically during the early stages of meiosis. This prevents the formation of spermatids and spermatozoa, leading to complete infertility. We have revised the text to replace "permanent block" with a more precise description: "confirming a sustained arrest of germ cell differentiation at the pre-meiotic stage" (Line 311-312)

(2) Presence of spermatocytes without active meiosis: The reviewer astutely connects this to Comment 23. As detailed in our previous response, the cells histologically classified as "spermatocytes" in the mutants are likely arrested at a pre-meiotic or very early leptotene-like stage. They have initiated a differentiation pathway that gives them this morphology but have failed molecularly to execute true meiosis, as evidenced by the absence of the synaptonemal complex protein SYCP3. Thus, the presence of these cells is consistent with a meiotic initiation or progression defect, not with successful meiosis.

(3) Sex determination in the absence of meiosis: Our mutants exhibit a 100% male sex ratio and develop male secondary sexual characteristics (e.g., breeding tubercles on fins, Fig. 2d). This unequivocally demonstrates that somatic sex determination has occurred correctly, directing the gonad to become a testis. The subsequent failure in gametogenesis (the meiotic block) is a consequence of the absence of CCK signaling within this now-determined testicular environment. The germ cells are present and begin to differentiate but cannot complete their development.

In summary, CCK is not required for the initial somatic events of male sex determination but is essential for the subsequent completion of germ cell meiosis within the determined testis.

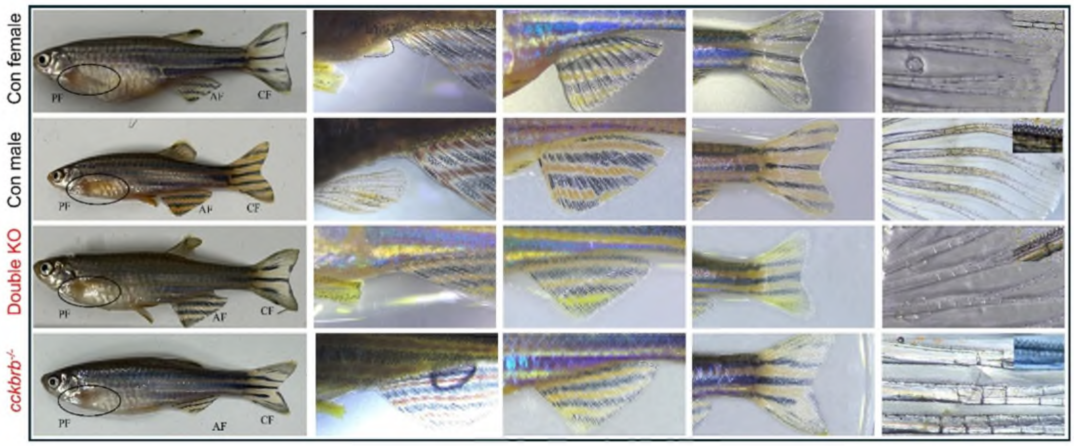

Fig. 2d Analysis of secondary sexual characteristics (SSC) in 4-month-old zebrafish. Representative images comparing the anal fin (AF), caudal fin (CF), and pectoral fin (PF) phenotypes in wild-type *cck1*$^{-/-}$;*cck2*$^{-/-}$ and *cckbrb*$^{-/-}$ zebrafish.

25. L286: Administering CCK through incubation is an interesting method. Any information about how efficiently the peptide is absorbed and which pathway(s) it may follow?

Response to Comment 25:

We thank the reviewer for this important question regarding the delivery efficiency and potential uptake pathways of the CCK peptide via our incubation method. To directly quantify uptake, we performed an Enzyme-Linked Immunosorbent Assay (ELISA) on whole-body lysates of zebrafish larvae following the standard CCK incubation protocol. The results, now presented in Supplementary Fig. S25, demonstrate a significant elevation in internal CCK peptide concentrations in the treated group compared to controls, confirming that the peptide is efficiently absorbed and maintained at detectable levels.

Regarding the specific absorption pathway, while precise pharmacokinetic dissection of dermal versus branchial routes is technically challenging in larvae, trans-epithelial absorption is a well-documented mechanism for peptide uptake in aquatic organisms at this stage. Most critically, the functional rescue of meiosis (Fig. 5d) serves as the ultimate biological validation of this method. The observation that immersion treatment successfully restored SYCP3 expression in mutant gonads confirms that the absorbed CCK is biologically active and capable of reaching target tissues to elicit a specific physiological response. We have updated the Methods section to include the details of this validation experiment.

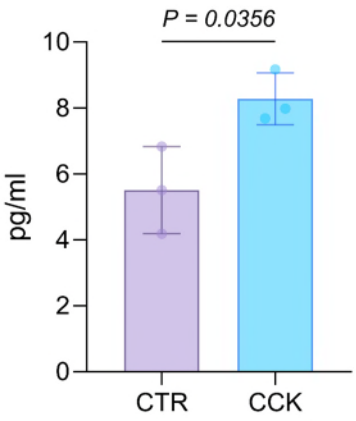

Supplementary Fig. S25 | Quantification of internal CCK concentration in larval zebrafish following waterborne immersion. Internal CCK concentration (pg/ml) was measured by ELISA in 48 hpf zebrafish larvae from Control (CTR) and CCK immersion groups after 1 hour treatment. Data are presented as mean ± s.e.m. for *n*= 3 independent biological replicates. Each replicate is a pooled sample of 20–30 individual larvae.

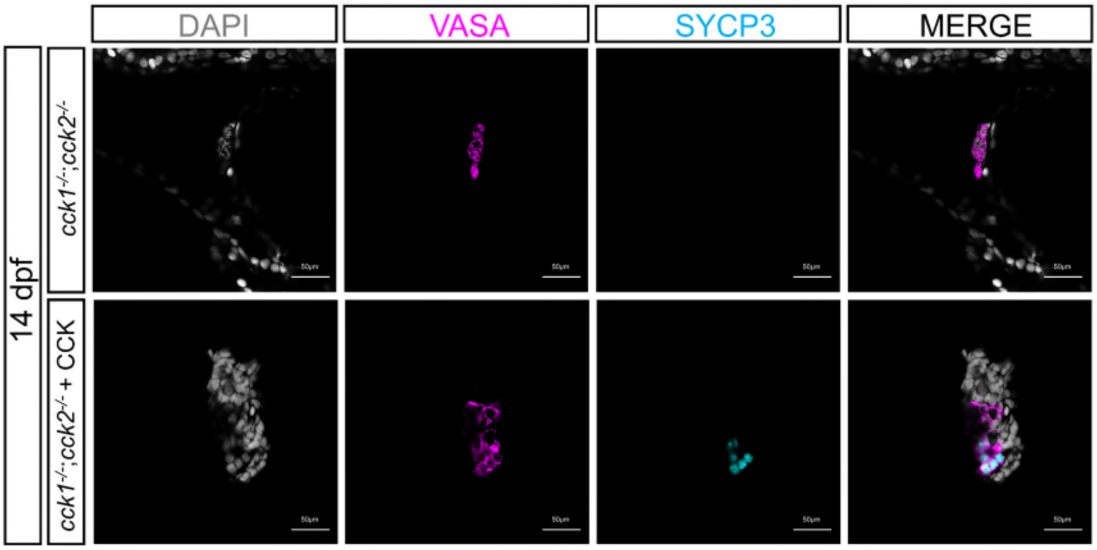

Fig. 5d VASA and SYCP3 co-staining in gonadal sections of 14 dpf *cck1*$^{-/-}$;*cck2*$^{-/-}$ larvae following exogenous CCK immersion treatment starting from fertilization.

26. L409: The manuscript should include comprehensive details about the fshb and lhb mutants generated and utilized in this study. Given that fshb and lhb knockout models have also been reported by several other laboratories, such information would be helpful for comparative analysis, particularly when discrepancies in phenotypes are observed.

Response to Comment 26:

We thank the reviewer for this important suggestion. We fully agree that providing comprehensive details on the genetic models is essential for transparency, reproducibility, and comparative analysis. In response, we have included Supplementary Fig. S17, which provides a full characterization of the *fshb* and *lhb* mutant lines generated for this study. This figure details the CRISPR/Cas9 targeting strategies and genomic lesions, alongside comprehensive morphological and histological analyses of the gonads in single and double mutants. Importantly, the phenotypic outcomes we observed—such as the sterility of *lhb*$^{-/-}$ females and the impaired ovarian development in *fshb*$^{-/-}$ females—are consistent with previous reports (e.g., Zhang et al., 2015; Chu et al., 2015). Providing these detailed molecular and phenotypic data allows for direct comparison with models from other laboratories and helps interpret potential discrepancies across studies. We have also updated the Methods section to reference this supplementary characterization.

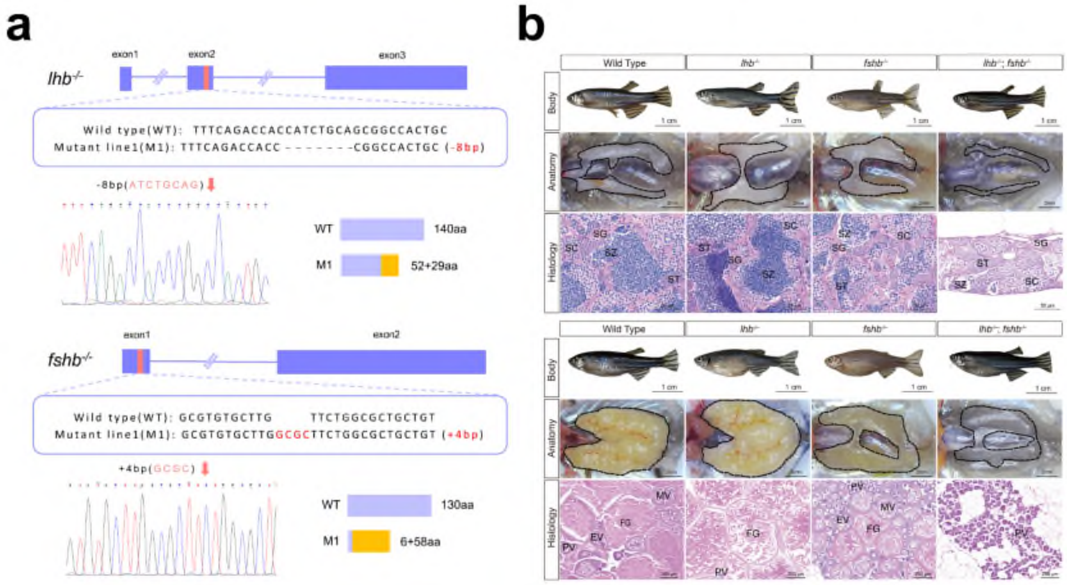

Supplementary Fig. S17 | Morphological and histological analysis of gonads in *lhb* and *fshb* knockout zebrafish models. a Schematic diagram showing the CRISPR/Cas9 targeting locations within the *lhb* and *fshb* genes. **b** Morphological and histological analysis of gonads in *lhb* and *fshb* single and double knockout zebrafish models compared to wild-type (WT) controls. Representative images illustrate the gross morphology and histological sections of the gonads. The developmental stages of oocytes and spermatogenesis are indicated by the following abbreviations: FG, fully mature oocytes; MV, mid-vitellogenic stage; EV, early vitellogenic stage; PV, pre-vitellogenic stage; PG, primary growth stage; SG, spermatogonia; SC, spermatocytes; ST, spermatids; SZ, mature sperm. Scale bars are indicated on the images.

27. L430-431: How was total number of follicles counted for each female?

Response to Comment 27:

We thank the reviewer for requesting clarification on our follicle quantification methodology. The total number of follicles was assessed using the proportional staging analysis method, a standard approach for comparing follicular developmental profiles across treatment groups, as previously described in Mitchell et al. (PNAS, 2020) and other established protocols. Our detailed procedure was as follows: Ovaries were fixed, sectioned at a thickness of 5-8 μm, and stained with Hematoxylin and Eosin. Follicles were then systematically classified into five distinct developmental stages: primary growth (PG), pre-vitellogenic (PV), early-vitellogenic (EV), mid-vitellogenic (MV), and full-grown (FG).

To obtain a representative profile for each ovary while avoiding double-counting, we counted all follicles present in every *n*-th section (e.g., every 5th section) throughout the entire ovary. The results were then expressed as the percentage distribution of follicles across the different developmental stages relative to the total number of follicles counted from that female.

We acknowledge that this proportional method is designed to provide a robust and comparative analysis of the follicular stage composition between experimental groups, rather than to report the absolute, non-redundant number of follicles per ovary. This approach effectively controls for variations in ovary size and focuses on the relative progression of folliculogenesis.

We have revised the Methods section (Lines 480-488) to provide a clearer and more precise description of this proportional counting methodology.

"Quantitative analysis of folliculogenesis was performed using a proportional staging method. To obtain a representative profile, all follicles across a systematic, non-adjacent series of sections (e.g., every 5th section) spanning the entire ovary were counted and classified into five developmental stages: primary growth (PG), pre-vitellogenic (PV), early-vitellogenic (EV), mid-vitellogenic (MV), and full-grown (FG). The results are expressed as the percentage distribution of follicles across these stages and are designed to compare the relative composition of the follicular population between samples, not to report absolute follicle counts."

28. L434: How was area occupied by spermatogonia quantified as they are often scattered individually across testis sections?

Response to Comment 28:

We appreciate this important question regarding the method's precision, especially for scattered cell types like spermatogonia. The quantification of the area occupied by spermatogonia (SG) was performed using morphometric analysis of Hematoxylin and Eosin (H&E) stained testis sections, a method adapted from established zebrafish protocols (Mitchell et al. 2020). This specific quantification involved capturing digital images of representative sections and using image analysis software to manually or semi-automatically outline and cumulatively quantify the total area covered by all cells classified as spermatogonia, accounting for both clustered and individually scattered cells. The final results are consistently presented as the percentage of the total testis area within each examined image, accurately reflecting the relative abundance of spermatogonia against the entire tissue mass. We have ensured the Methods section (L480-488) clearly describes this area-based morphometric quantification approach.

29. L496: Embryos cannot be fertilized.

Response to Comment 29:

We appreciate the reviewer's attention to detail regarding this semantic inaccuracy. We have corrected the wording in the revised manuscript (Line 592-595) for greater precision. The corrected sentence now reads: "Briefly, embryos, generated from *cck1*$^{+/-}$;*cck2*$^{+/-}$ heterozygous crosses, were then incubated in E3 medium containing a CCK cocktail (0.02 µg/mL CCK1 and 0.02 µg/mL CCK2)." This clarifies that the generated embryos were the material being incubated, not the subject of fertilization.

30. L502: Feed supplementation is an interesting approach for peptide delivery. However, this should be validated for the following: (1) doses used; (2) efficiency of absorption; and (3) serum or tissue concentrations.

Response to Comment 30:

We agree with the reviewer that rigorous validation of the peptide delivery system is essential when using feed supplementation. To substantiate the effectiveness and biological relevance of our approach, we performed quantitative analysis addressing the key pharmacokinetic parameters. The doses used were optimized based on preliminary feeding trials. To validate the delivery efficiency and systemic concentration, we quantified plasma CCK levels using ELISA following the feeding regimen. The results, now presented in Supplementary Fig. S9, demonstrate a significant elevation in circulating CCK levels 1.5 hours post-feeding in the treated group compared to controls, confirming that the administered dose is sufficient to achieve systemic absorption. Furthermore, we validated the biological efficacy of the absorbed peptide by measuring the functional outcome in the target organ, observing a robust induction of pituitary *fshb* mRNA expression in the CCK-fed group (Supplementary Fig. S9). The combination of elevated plasma concentrations and specific transcriptional response provides definitive evidence that the fed CCK is effectively absorbed and biologically active. We have ensured that the Methods section and the Supplementary Information now clearly present these data (Line 611-624).

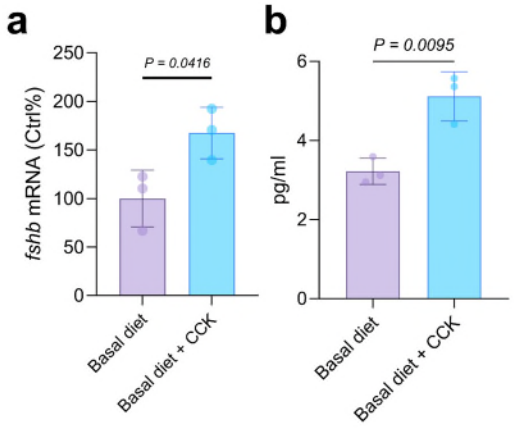

Supplementary Fig. S9 | Confirmation of CCK feeding efficacy and resulting impact on pituitary *fshb* expression. The figure confirms the effective absorption of dietary CCK and its subsequent biological effect, measured 1.5 hours post-feeding with a CCK-supplemented diet versus a normal diet. **a** Relative mRNA expression level of *fshb* in the pituitary gland (determined by qPCR). Data are presented as mean ± s.e.m. for n= 3 independent replicates. Each CCK-fed replicate is a pool of four pituitaries; each normal diet replicate is one pituitary. **b** Plasma CCK concentration (determined by ELISA). Data are presented as mean ± s.e.m. for n= 3 independent replicates. Each replicate is a pool of blood from three individual fish.

31. The manuscript is very well written, clear and easy to follow. It appears that the authors used AI tools to assist in writing, which is perfectly fine and demonstrate the value of AI technology. However, the authors should disclose the use and indicate which tools were used. Also, the authors should try to humanize the final version to remove signs of AI writing, including the words commonly over-used by AI tools, such as Concurrently, Crucially, Notably, etc.

Response to Comment 31:

We sincerely thank the reviewer for the highly positive feedback regarding the clarity and flow of our manuscript and appreciate the insightful comment concerning the use of AI tools. (1) Disclosure: We confirm that we utilized Gemini primarily for language refinement, grammar checks, and improving sentence structure during the drafting process. We have added a disclosure statement in the Acknowledgements section of the revised manuscript to transparently indicate this assistance, in line with the journal's emerging guidelines on AI usage. (2) Humanization/Tone: We fully agree that the final version must reflect the authors' voice. Following the reviewer's suggestion, we have conducted a thorough humanization pass of the entire manuscript, specifically targeting and replacing common overused phrases and transitional words (such as "Concurrently," "Crucially," and "Notably") with more varied, natural-sounding, and direct phrasing. We believe these steps have successfully addressed the reviewer's concerns while maintaining the praised clarity and quality of the writing.

"Author Contributions Statement
The authors acknowledge the use of Gemini (Google) for language editing and polishing of the manuscript."

References

1. Chu, L., Li, J., Liu, Y. & Cheng, C. H. Gonadotropin signaling in zebrafish ovary and testis development: Insights from gene knockout study. *Mol. Endocrinol.* **29**, 1743-1758 (2015).
2. Farrell, J. A., Wang, Y., Riesenfeld, S. J. et al. Single-cell reconstruction of developmental trajectories during zebrafish embryogenesis. *Science*, **360**, eaar3131(2018).
3. Mitchell, K. et al. Targeted mutation of secretogranin-2 disrupts sexual behavior and reproduction in zebrafish. *PNAS*. **117**, 12772-12783 (2020).
4. Pan, Y. J., Tong, S. K., Hsu, C. W., Weng, J. H. & Chung, B. C. Zebrafish Establish Female Germ Cell Identity by Advancing Cell Proliferation and Meiosis. *Front. Cell. Dev. Biol.* **10**, 866267 (2022).
5. Tanaka, S., Yu, Y., Levavi-Sivan, B., Zmora, N. & Zohar, Y. GnRH—gonadotropes interactions revealed by pituitary single-cell transcriptomics in zebrafish. *Endocrinology.* **165**, bqae151 (2024).
6. Trudeau, V. L. & Somoza, G. M. Multimodal hypothalamo-hypophysial communication in the vertebrates. *Gen. Comp. Endocrinol.* **293**, 113475 (2020).
7. Whitlock, K.E., Postlethwait, J. & Ewer, J. Neuroendocrinology of reproduction: Is gonadotropin releasing hormone (GnRH) dispensable. *Front Neuroendocrinol*. **53**, 100738 (2019).
8. Zhang, Z., Lau, S-W., Zhang, L. & Ge, W. Disruption of zebrafish follicle-stimulating hormone receptor (*fshr*) but not luteinizing hormone receptor (*lhcgr*) gene by TALEN leads to failed follicle activation in females followed by sexual reversal to males. *Endocrinology.* **156**, 3747-3762 (2015).

Reviewer #2 (Remarks to the Author):

Summary or Overall assessment

The authors present a comprehensive genetic study demonstrating that the CCK signaling pathway serves as a distinct regulator of FSH and LH expression in teleosts, independent of GnRH—a finding of notable academic value that challenges classical models. To my knowledge, this is the third independent group to validate this concept using a fish model, following studies published in Nature Communications (Shun Kenny Uehara et al., 2024) and eLife (Lian Hollander-Cohen et al., 2024). The consistency of the present findings with prior work further enhances their credibility. Through detailed phenotypic analyses, the authors outline multiple roles of CCK across key stages of gametogenesis, including germ cell proliferation, meiotic initiation, somatic cell survival, sex determination, and the regulation of both FSH and LH. While phenotypic descriptions are thorough, mechanistic insights remain limited, which somewhat constrains the study's depth and broader impact. Nonetheless, given the strength of the dataset and the importance of the topic, the manuscript holds significant potential if mechanistic aspects are further developed. Below are my specific comments on the manuscript.

Response:

We sincerely thank the reviewer for this highly thoughtful and positive assessment of our manuscript. We are greatly encouraged by the recognition of the academic value of our findings and their consistency with recent breakthroughs in the field. We fully agree that providing deeper mechanistic insights is critical to maximizing the study's impact. In strict accordance with your suggestions, we have performed extensive new experiments to decipher the underlying molecular and cellular mechanisms.

Specifically, we have elucidated two core regulatory pathways:

(1) We identified a dual regulatory mechanism where CCK drives LH synthesis through both direct action on a newly identified *fshb/lhb* co-expressing pituitary subpopulation and an indirect hypothalamic *gnrh3*-dependent pathway.

(2) We have further elucidated a core mechanism by which CCK regulates early gonadal development: it maintains gonadal somatic cell survival, thereby indirectly controlling the initiation of germ cell meiosis. This finding was directly validated by our genetic rescue experiment—inhibiting apoptosis through *tp53* knockout in *cckbrb* mutants successfully restore meiotic initiation (indicated by the recovery of SYCP3 expression) and rescue gonadal morphology. This demonstrates that somatic cell integrity is a critical downstream step through which CCK signaling regulates meiotic progression.

We believe these specific mechanistic advances significantly strengthen the depth and impact of the revised manuscript, and we provide a point-by-point response to your detailed comments below.

Major Concerns：

1.In Figure 1c, please indicate the age of the fish used and specify the experimental groups represented by the different colored bars in the quantification plot. While the data can be interpreted by an expert reader, it is essential to include a clear and complete legend to ensure rigorous and reproducible reporting. More broadly, figure legends involving animal experiments should report the animal's age, sample size, data format (e.g., mean ± s.e.m.), and statistical test used to ensure transparency and reproducibility. In addition, this issue is not limited to Figure 1 (Such as Fig. 2 et al). Throughout the manuscript, the age of animals used in all experiments—including histological analyses, immunofluorescence, and other animal-based assays—should be clearly stated.

Response to Comment 1:

We sincerely thank the reviewer for highlighting this critical requirement for rigorous and reproducible reporting. We fully agree that complete and transparent figure legends are essential for the interpretation of the data.

Specific Revision of Figure 1c:

As specifically requested, we have updated the legend for Figure 1c to explicitly state that the analysis was performed on 3-month-old zebrafish and to define the exact sample sizes for quantification (*n*= 6 for ovaries, *n*= 7 for testes). The data are presented as the mean percentage ± s.e.m., and statistical analysis was performed using one-way ANOVA with Dunnett's post-hoc test.

Global Revision of Manuscript:

We have conducted a comprehensive review and revision of all figure legends to ensure this standard is applied throughout the manuscript. For example: **Figure 2b** now specifies the use of 5-month-old zebrafish for gonadal histological analysis and GSI analysis (n=7 for histology, n=15 for GSI). **Figure 2d** specifies the analysis of secondary sexual characteristics in 4-month-old zebrafish. Similar clarifications have been made for all other figures including the age of animals, sample size (n), data format (mean ± s.e.m.), and the specific statistical test used.

We have ensured that the age of the animals, sample size (*n*), data format (e.g., mean pm±s.e.m.), and statistical tests are clearly stated in every figure legend involving animal experiments. Furthermore, these experimental details have been cross-verified with the Methods section to ensure full transparency and reproducibility.

2.The authors report that CCK regulates both FSH and LH, which contrasts with prior studies (Uehara et al., Nature Communications, 2024; Hollander-Cohen et al., eLife, 2024) that concluded CCK specifically regulates FSH, not LH. This discrepancy should be addressed in the discussion with direct comparison and mechanistic context. If confirmed, dual regulation of FSH and LH by CCK would be a significant advance. However, current evidence for LH regulation is preliminary—mainly based on spatial co-expression and indirect induction. Stronger functional validation or clearer discussion of the limitations is needed, especially as this may represent the study's most novel contribution.

Response to Comment 2:

We thank the reviewer for this central comment, which rightly identifies the regulation of LH by CCK as a potentially major novel finding of our study, and for urging stronger validation. We acknowledge the apparent discrepancy with the prior studies of Uehara et al. and Hollander-Cohen et al. and have now substantially strengthened the evidence for dual regulation and provided a mechanistic context for this difference in the revised Discussion.

Revised Discussion Excerpt 1: Confirmation of FSH-RH Role (Line 391-408)

"Following gonadal differentiation, zebrafish undergo an extended developmental phase characterized by follicle transition from Stage IB to Stage II, marking female puberty onset[40, 41]. where FSH plays a central role. Our observation that loss of CCK signaling leads to a severe reduction in *fshb* synthesis, which is rescuable by exogenous CCK, aligns robustly with recent breakthroughs in the field. Specifically, seminal studies in medaka[8] and zebrafish[9] have identified CCK as a potent FSH-RH. Our data, derived from comprehensive genetic knockout models, provide independent confirmation of this conserved physiological axis, reinforcing the consensus that CCK directly modulates FSH output to drive gametogenesis in teleosts. Evolutionarily, teleosts exhibit a direct CCK-FSH regulatory axis, wherein FSH-producing gonadotropes express functional CCKRs[8,42,43]. This architecture allows the satiety hormone CCK to directly modulate FSH output, representing a key adaptation in broadcast spawners like zebrafish that require sustained energy investment into oogenesis. In contrast, mammals exhibit a different strategy: FSH and LH are co-produced by the same gonadotropes, which lack CCKRs, implying that FSH regulation in these groups is indirect and likely mediated through upstream hypothalamic signals[44-46]."

Revised Discussion Excerpt 2: Confirmation of LH-RH Role (Line 409-434)

"Upon completion of gametogenesis, the gonads enter the final maturation stage, during which LH plays a critical role in promoting oocyte maturation and ovulation. In *lhb*$^{-/-}$

zebrafish, oocyte growth proceeds normally, but final maturation and ovulation are significantly impaired[11, 12, 47]. Building upon the established role of CCK as a major FSH-RH, our study reveals a significant expansion of its neuroendocrine repertoire: CCK also functions as a critical regulator of LH in zebrafish. Both *cck1*$^{-/-}$;*cck2*$^{-/-}$ and *cckbrb*$^{-/-}$ mutants exhibited markedly reduced pituitary LH expression, implicating CCK in LH synthesis and secretion. The apparent discrepancy with earlier studies concluding a specific FSH-RH role likely stems from their primary focus on establishing that novel function, and from our identification of a specific cellular substrate: a subpopulation of pituitary gonadotropes that co-express *fshb* and *lhb* transcripts[43] and are enriched for the *cckbrb* receptor, providing a basis for dual regulation.

Mechanistically, CCK regulates LH through a dual pathway. It directly stimulates *lhb* expression in pituitary cells, an action supported by the presence of CCKBRb in cells co-expressing FSH and LH. Additionally, CCK operates via an indirect, GnRH3-mediated route, significantly upregulating hypothalamic *gnrh3* expression. This dual mechanism ensures robust LH output. Functionally, the physiological relevance of this regulation is confirmed by the effective induction of ovulation in female zebrafish following exogenous administration of either CCK or LH. Therefore, our findings extend the existing paradigm, positioning CCK not as a solo FSH-RH, but as a dual gonadotropin-releasing factor that integrates the control of gametogenesis (via FSH) and final maturation/ovulation (via LH), underscoring its role as a master coordinator within the teleost reproductive neuroendocrine system."

3. Line 215-216: The manuscript compares the phenotypes of fshb-/-, lh-/-, and double mutants with those of cck-deficient zebrafish to support the gonadotropin-independent function of CCK. Please clarify whether these lhb/fshb mutants were newly generated in this study. If so, are their phenotypes consistent with previous reports? Additional details on their construction, genotyping, and phenotypic validation would strengthen the interpretation.

Response to Comment 3:

We thank the reviewer for requesting clarification on the *fshb* and *lhb* mutant lines used for comparative analysis. These knockout models were generated in our laboratory for this study. While their observed phenotypes are consistent with previous reports in the literature, we agree that providing construction details is essential for transparency. As fully addressed in our response to Comment 26 (Reviewer 1), we have included comprehensive information including the specific target site, knockout strategy, and schematic diagrams in the Supplementary Fig. S17. This supplementary figure provides the necessary details on their construction, genotyping, and validation to fully support the interpretation of the comparative analyses presented in the manuscript.

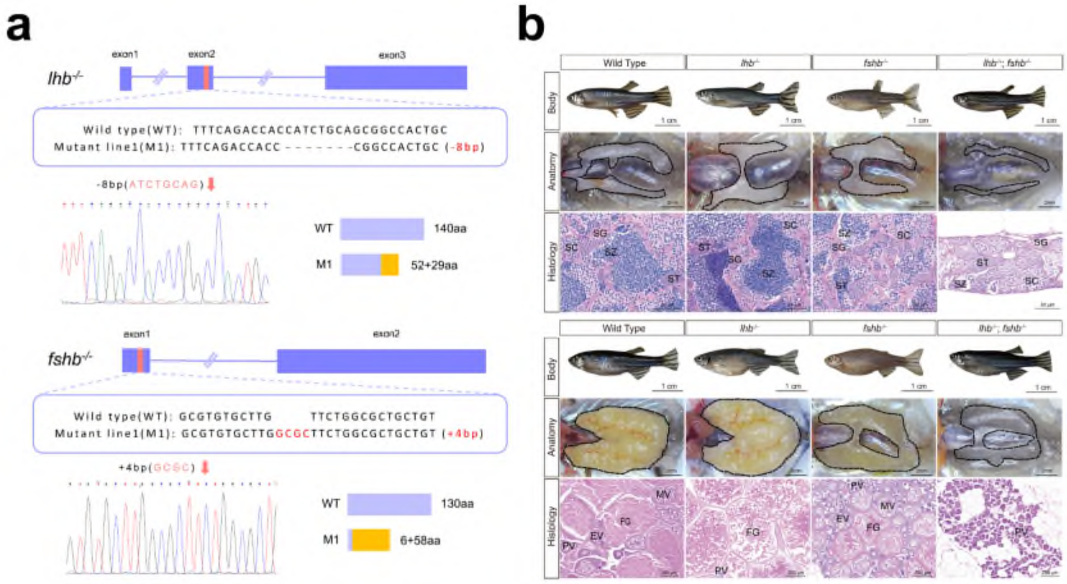

Supplementary Fig. S17 | Morphological and histological analysis of gonads in *lhb* and *fshb* knockout zebrafish models. a Schematic diagram showing the CRISPR/Cas9 targeting locations within the *lhb* and *fshb* genes. **b** Morphological and histological analysis of gonads in *lhb* and *fshb* single and double knockout zebrafish models compared to wild-type (WT) controls. Representative images illustrate the gross morphology and histological sections of the gonads. The developmental stages of oocytes and spermatogenesis are indicated by the following abbreviations: FG, fully mature oocytes; MV, mid-vitellogenic stage; EV, early vitellogenic stage; PV, pre-vitellogenic stage; PG, primary growth stage; SG, spermatogonia; SC, spermatocytes; ST, spermatids; SZ, mature sperm. Scale bars are indicated on the images.

4. Fig 4: The authors assess PGC proliferation at 14 dpf using VASA and H3P staining and report a marked reduction in cckbrb mutants. However, have the authors considered the possibility of developmental delay in mutants, which may confound the assessment of proliferation at a fixed time point? Including a comparison of general developmental timing or discussing this potential bias would be helpful.

Response to Comment 4:

We thank the reviewer for raising this important consideration regarding the potential for a general developmental delay in the mutants, which could confound the interpretation of proliferation defects at a fixed time point. We have carefully addressed this possibility and provide compelling evidence that the reduced PGC proliferation is a specific and sustained defect.

First, we rule out a generalized developmental delay. Contrary to what a developmental lag would predict, our *cck* mutants exhibit normal or even accelerated somatic growth in terms of body length and weight compared to wild-type controls (Fig. 1f). This demonstrates that the observed germ cell phenotype is not secondary to an overall retardation of organismal development.

Second, we demonstrate the defect is persistent, not transient. If the phenotype were merely a delay, one would expect a "catch-up" in proliferation at later developmental stages. To test this, we performed a longitudinal analysis of germ cell proliferation in *cckbrb* mutants at multiple time points: 45, 90, and 180 days post-fertilization (dpf). The results, now included as Supplementary Fig. S20, reveal a persistent and significant reduction in mitotic activity (Histone H3-positive cells) and a failure to expand the germ cell population throughout development into adulthood.

Therefore, the combination of normal somatic growth and a sustained proliferative block across the lifespan confirms that the phenotype represents a specific, long-lasting impairment in germ cell development intrinsic to the loss of CCK signaling, rather than a consequence of general developmental timing.

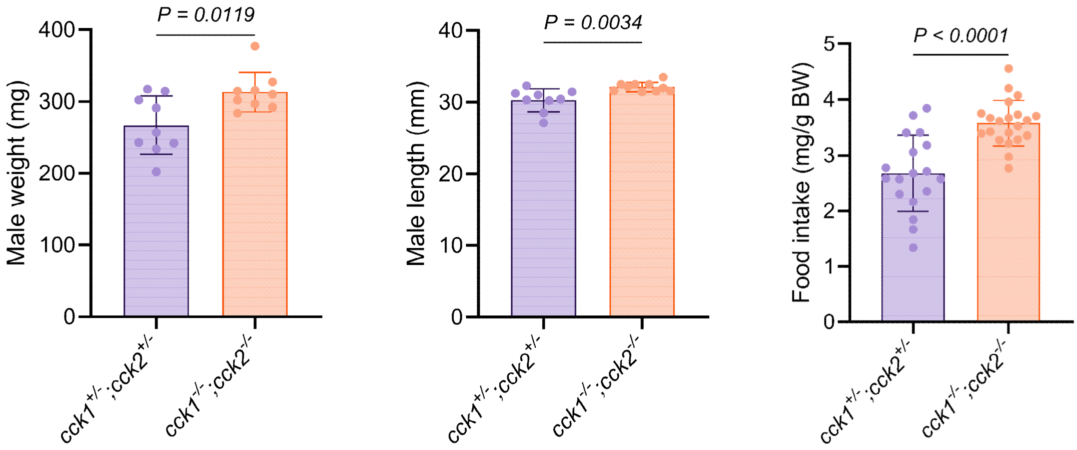

Fig. 1f Comparisons of body weight (*n*= 9), body length (*n*= 9), and food intake (*n*= 18). Data are presented as mean ± s.e.m.

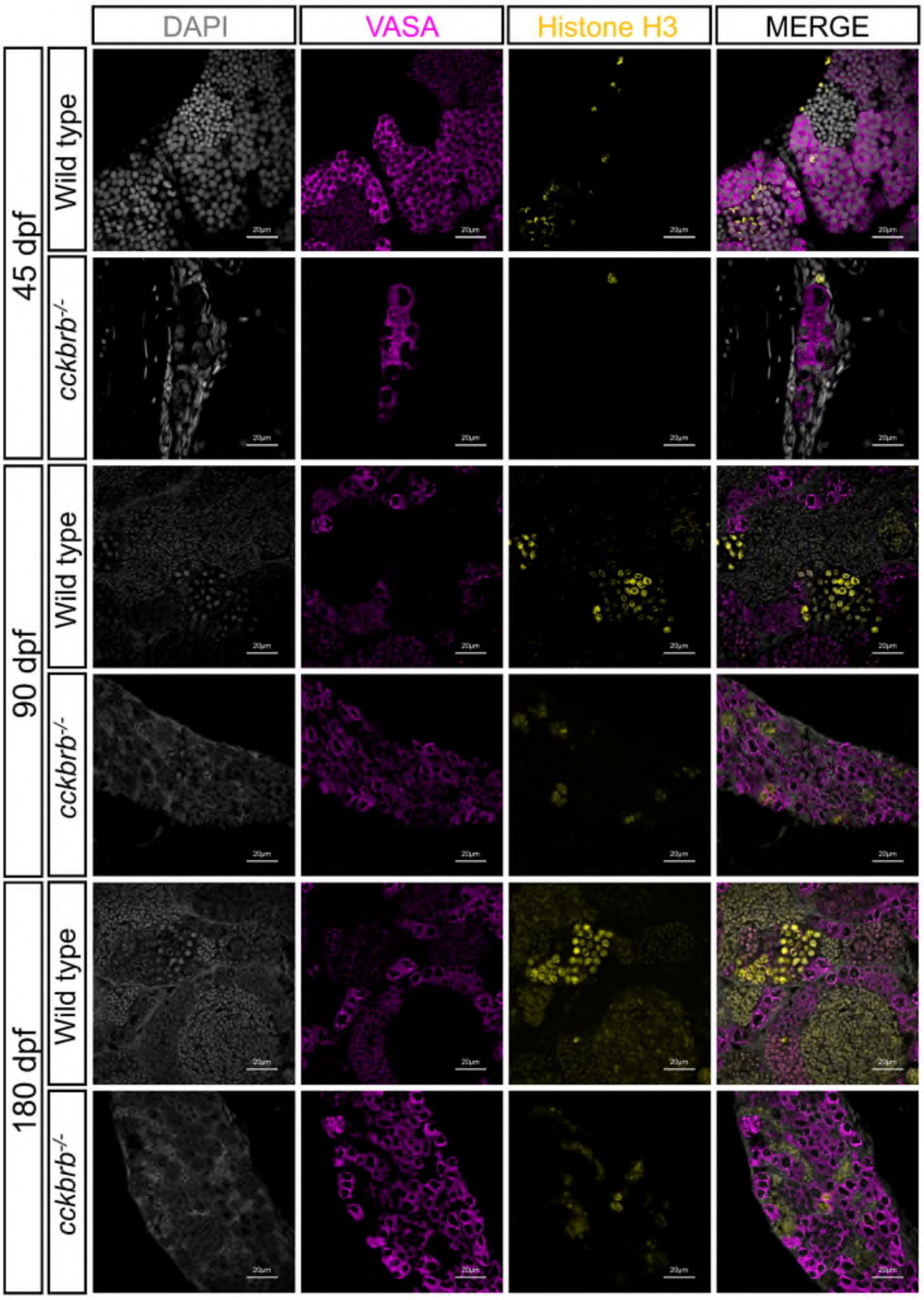

Supplementary Fig. S20 | Immunofluorescence analysis of germ cell proliferation in *cckbrb*$^{-/-}$ zebrafish at different developmental stages. The figure displays Immunofluorescence (IF) co-staining of gonadal sections from Wild-type (WT) and *cckbrb*$^{-/-}$ zebrafish at 45 dpf, 90 dpf, and 180 dpf. The sections were stained for VASA (a germ cell marker) and Histone H3 (H3) (a mitotic cell marker) to assess germ cell proliferation. Scale bars are indicated on the images.

5.The authors attribute the all-male phenotype in CCK pathway mutants to disrupted PGC migration, proliferation, meiosis, and somatic cell survival. However, this remains correlative. In zebrafish, PGC loss may be a consequence—not a cause—of male development, and the possibility that CCK directly affects sex determination cannot be excluded. More critically, this model is inconsistent with findings in medaka: cck2rb knockout medaka still develop females (Uehara et al., Nature Communications, 2024). If CCK directly reduced PGCs, a similar male bias would be expected, which was not observed. This suggests that CCK may not directly control PGC number. The authors should interpret this mechanism more cautiously and consider alternative explanations. Additional molecular evidence demonstrating how CCK directly regulates PGC number—or PGC rescue experiments—would help clarify the causality and strengthen the conclusions.

Response to Comment 5:

We thank the reviewer for this highly insightful and critical comment. We attribute the discrepancy between our all-male zebrafish mutants and the female-retaining medaka mutants (Uehara et al., 2024) to the fundamental disparity in their sex determination systems. Medaka possesses a hard-wired XX/XY genetic sex determination system (Matsuda et al., 2002), which buffers against germ cell fluctuations. In contrast, zebrafish exhibit a highly plastic system where female differentiation strictly relies on a threshold number of germ cells; insufficient PGCs trigger the default male pathway (Moore et al., 2013; Liew et al., 2012). Therefore, the reduction in PGCs caused by CCK loss triggers the default male fate in zebrafish, whereas the genetic switch in medaka overrides this defect. We have expanded the Discussion to explicitly address this species-specific plasticity.

To address the concern regarding causality, we present evidence establishing that the PGC defect precedes sex differentiation and is driven by a somatic mechanism. We observed significant PGC migration defects at 24 hpf (Fig. 4a) and proliferation arrest/somatic apoptosis at 14 dpf (Fig. 4f, 5f). Since somatic masculinization typically commences around 20–25 dpf, these early defects confirm that PGC loss is the cause (upstream driver) rather than the consequence. Mechanistically, as detailed in our response to Reviewer 1 (Comment 14), our single-cell analysis revealed negligible *cckbrb* expression in PGCs (Fig. R5), supporting an indirect, somatic cell-mediated mechanism. Instead, CCK maintains the gonadal somatic niche (Fig. 4e), and its loss leads to massive somatic apoptosis (Fig. 5f). Crucially, inhibiting this apoptosis via *tp53* knockout rescues both somatic cell survival and the initiation of meiosis in germ cells (Fig. 5g, h). This genetic rescue experiment provides direct causal proof that CCK promotes germ cell development by maintaining the integrity of the somatic niche.

In summary, the all-male phenotype in zebrafish CCK mutants results from an early, somatic cell-mediated failure of the gonadal niche, which leads to PGC deficiency and thereby triggers the germ cell-dependent default male pathway. This mechanism is fully consistent with the plastic sex determination system of zebrafish and is distinct from the outcome in genetically determined medaka. Our model, supported by temporal and genetic rescue evidence, clarifies that CCK acts as a master regulator of the somatic niche, indirectly ensuring germ cell survival and guiding sexual fate.

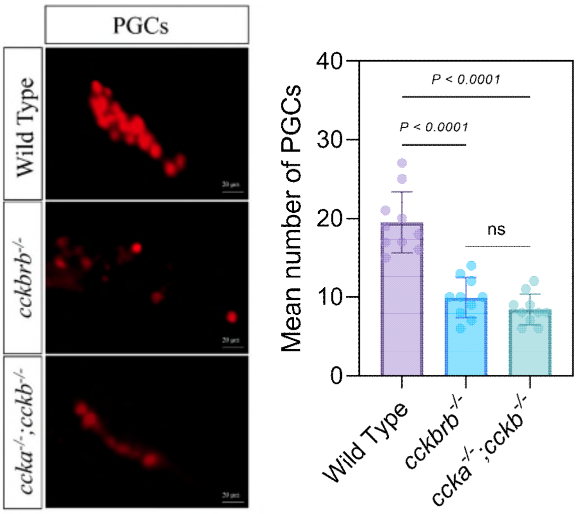

Fig. 4a Representative images and quantification of PGCs in Wild-type (WT), *cckbrb*$^{-/-}$, and *cck1*$^{-/-}$; *cck2*$^{-/-}$ embryos.

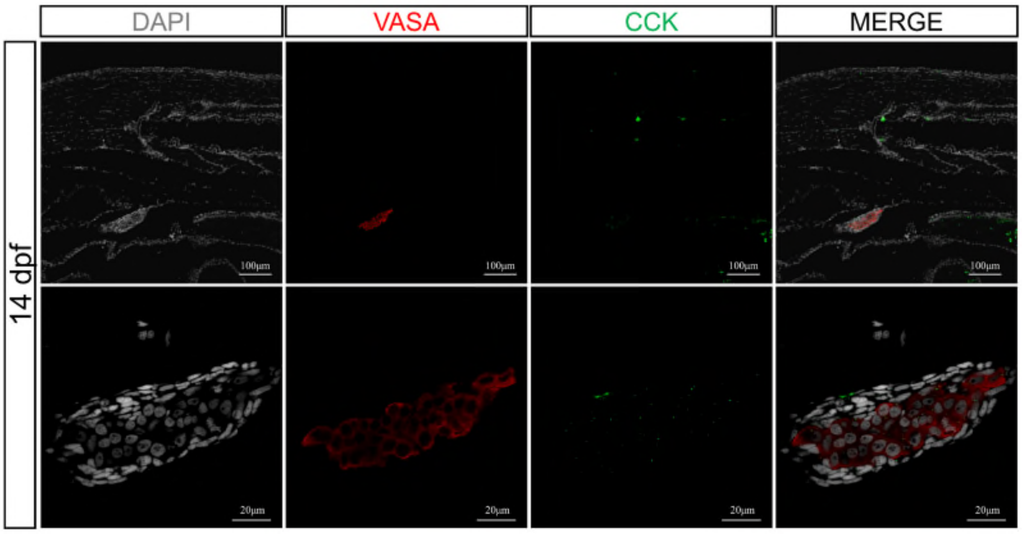

Fig. 4e Immunofluorescence of CCK (green) and VASA (red) in 14 dpf gonads reveals CCK expression in somatic cells surrounding germ cells.

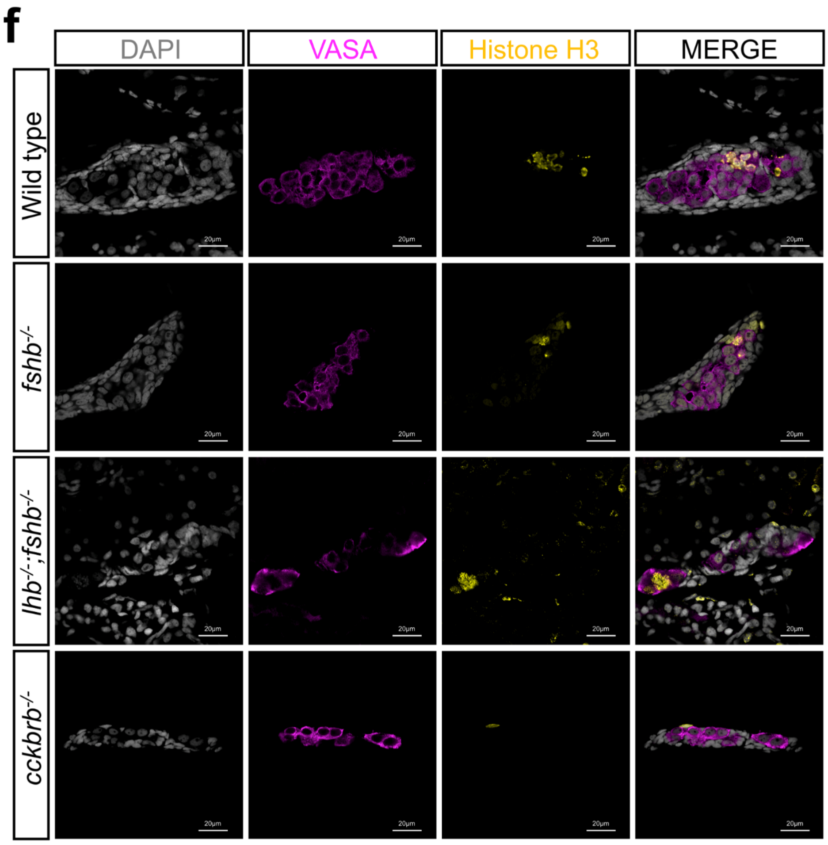

Fig. 4f Immunofluorescence co-expression analysis of germ cells (VASA) and mitotic cells (Histone H3) in gonadal tissue of WT, *fshb*-/-, *lhb*-/-;*fshb*-/-, and *cckbrb*-/- zebrafish at 14 dpf.

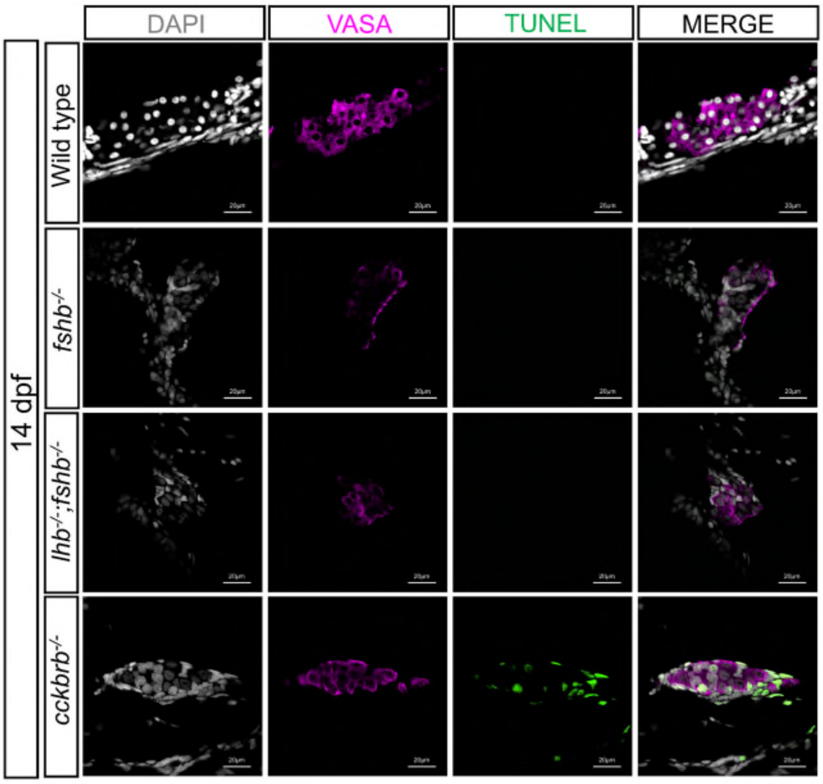

Fig. 5f TUNEL and VASA staining in 14 dpf gonads of WT, *fshb*-/-, *lhb*-/-;*fshb*-/-, and *cckbrb*-/- zebrafish.

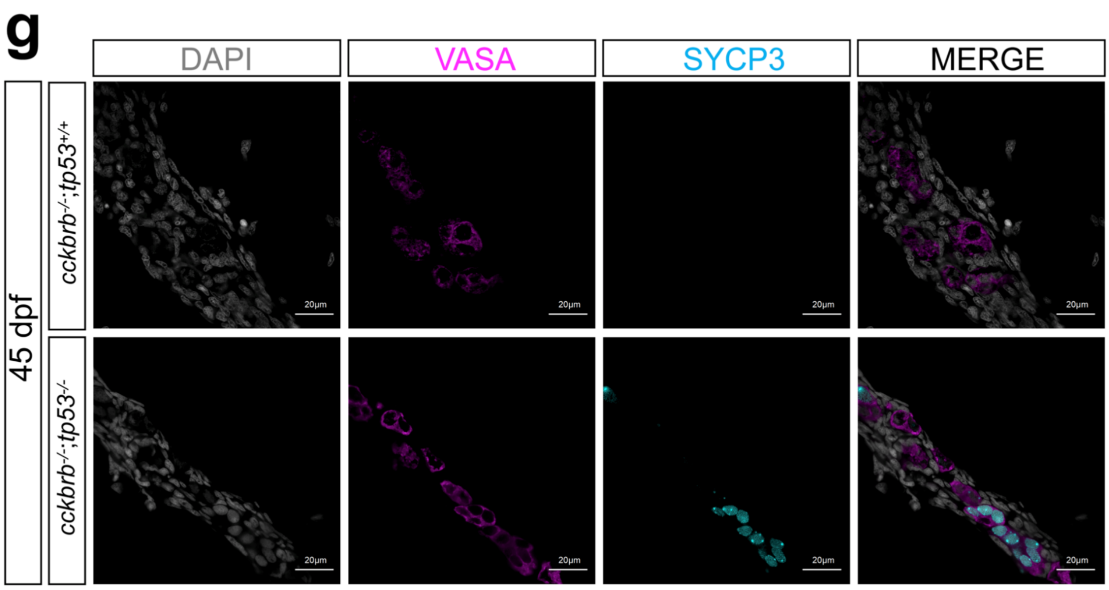

Fig. 5g Immunofluorescence of SYCP3 and VASA in testes at 45 dpf.

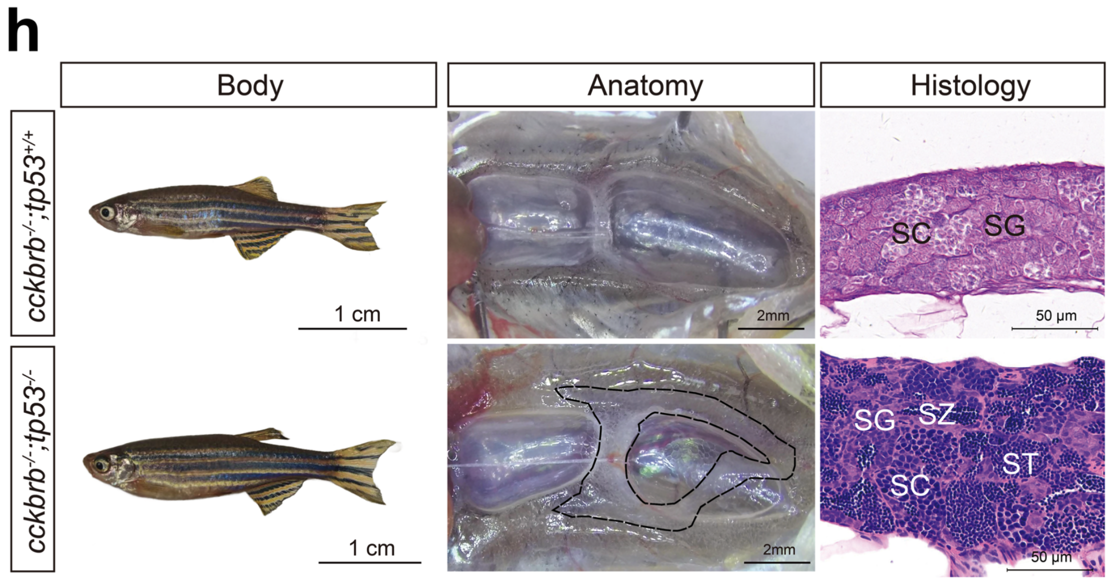

Fig. 5h Gross morphology and histological analysis (H&E) of gonads.

[Figure Redacted]

Fig. R5 | Negligible expression of *cckbrb* in the zebrafish PGC lineage during early embryogenesis. a, b Visualization of the single-cell developmental trajectory from Farrell et al. (2018) (Source: https://singlecell.broadinstitute.org/single_cell/study/SCP162). The UMAP plots display cell clusters colored by (**a**) lineage identity (with PGCs indicated) and (**b**) developmental stage (from High to 6-somite). **c**, Expression distribution of *cckbrb* mapped onto the trajectory. The green arrow indicates the PGC cluster. Note that *cckbrb* expression is extremely sparse, detected in only 3 cells at the 50% epiboly stage and 1 cell at the 90% epiboly stage across the entire dataset (Log2 expression values: 1.96, 1.88, 1.54, 0.94). This lack of robust expression in the PGC lineage supports the hypothesis that CCK regulation of PGCs is likely indirect.

6. The authors propose a central role for CCK in integrating metabolic and reproductive regulation in zebrafish. However, the potential species specificity, crosstalk with other metabolic regulators (e.g., NPY, ghrelin), and indirect effects through altered energy status remain underexplored. The discussion would benefit from acknowledging these limitations and outlining future directions, such as investigating feedback mechanisms or inter-organ communication pathways.

Response to Comment 6:

We sincerely thank the reviewer for highlighting the necessity of placing CCK's role within the broader metabolic context. We fully agree that providing empirical evidence for metabolic integration is crucial to substantiate our "Master Coordinator" model. To address this, we performed extensive new studies focused on the Glucose-Insulin-CCK Gonadal Axis, the results of which decisively support CCK's central integrating role. We found that a high-carbohydrate (HG) diet significantly increased both blood glucose levels and brain *cck1/cck2* expression, correlating with enhanced gonadal development in wild-type fish (Fig. R6A, B). Crucially, this pro-gonadal effect is dependent on CCK, as the HG diet failed to rescue the severe gonadal atrophy in *cck1*$^{-/-}$;*cck2*$^{-/-}$ double mutants (Fig. R6C). To elucidate the upstream mechanism, we demonstrated that glucose injection upregulates brain CCK expression (Fig. R7A) and that insulin receptors (*insra/insrb*) are specifically enriched in hypothalamic CCK neurons (Fig. R7B). Furthermore, glucose stimulation directly upregulates *cck* in isolated hypothalamic cells (Fig. R7C), and *insra/insrb* knockout zebrafish exhibit reduced *cck* expression and a male-biased sex ratio (Fig. R7D), mirroring the *cck* mutant phenotype. These findings validate a Glucose-Insulin-CCK-Gonad axis.

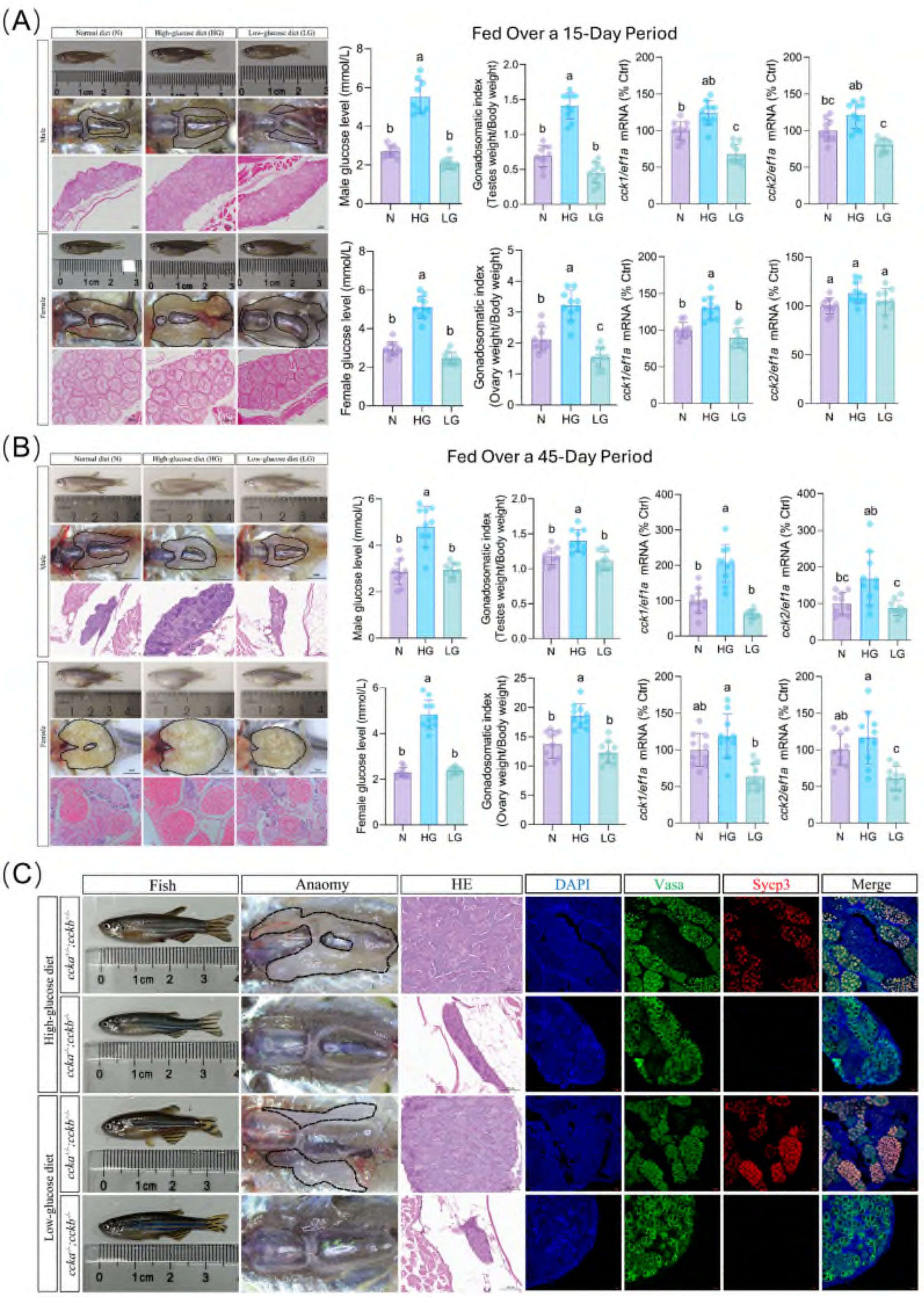

Fig.R6 Effects of high-carbohydrate diet on gonadal development and brain CCK expression in zebrafish. (A) Gonadal developmental status and brain *cck1* and *cck2* expression levels in zebrafish at 45 days post-fertilization (dpf), after feeding diets with varying starch contents for 15 days. (B) Gonadal developmental status and brain *cck1* and *cck2* expression levels in zebrafish at 45 dpf, after feeding diets with varying starch contents for 45 days. (C) Comparative analysis of gonadal development (HE staining and immunofluorescence staining) in 45 dpf *cck1*$^{+/-}$;*cck2*$^{+/-}$ and *cck1*$^{-/-}$;*cck2*$^{-/-}$ zebrafish fed high-glucose (HG) or low-glucose (LG) diets for 45 days. Scale bars are indicated.

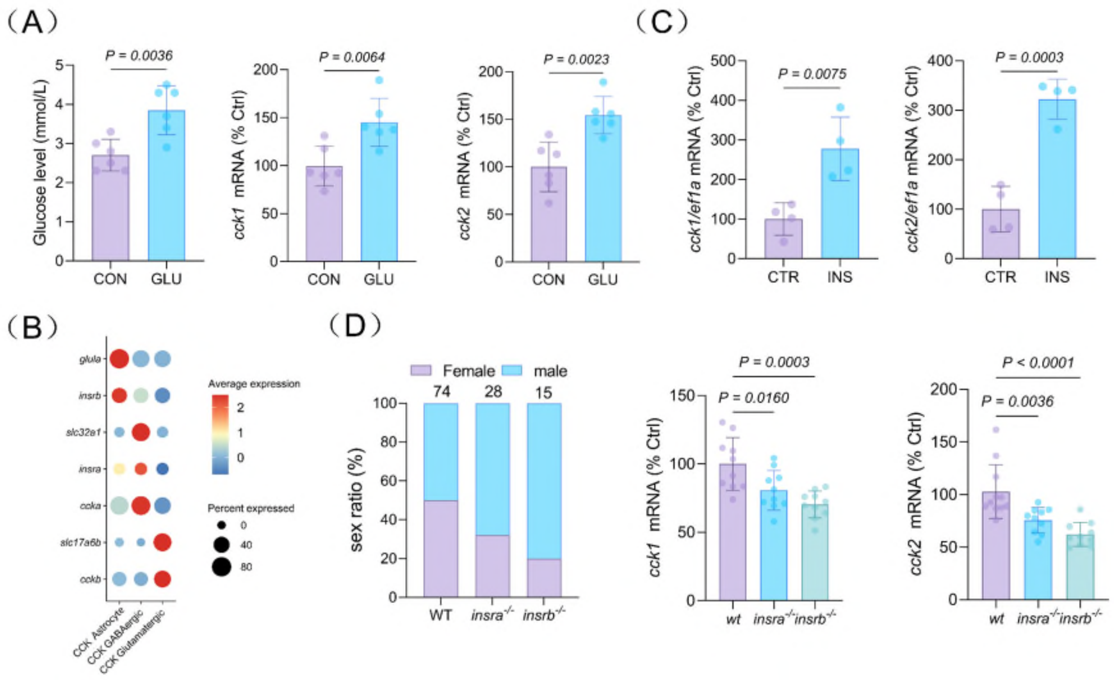

Fig. R7 Mechanisms by which glucose metabolism regulates zebrafish CCK expression via insulin receptors *insra* and *insrb*. (A) Blood glucose levels and brain *cck1* and *cck2* gene expression changes at 3 hours post-intraperitoneal glucose injection in zebrafish. Data are presented as mean ± SEM. (B) Single-cell transcriptomic analysis revealing the expression patterns of *insra* and *insrb* genes in hypothalamic CCK neurons of zebrafish. (C) Changes in *cck1* and *cck2* mRNA expression levels in cultured zebrafish hypothalamic cells after glucose treatment for 3 hours. Data are presented as mean ± SEM. (D) Sex ratio analysis and brain *cck1* and *cck2* gene expression levels in *insra* and *insrb* knockout zebrafish models. Data are presented as mean ± SEM.

Minor Comments：

7. Line 114-116 (Fig 2d): The authors show that male double mutants exhibit normal secondary sexual characteristics, an important observation suggesting intact androgen signaling. This finding effectively rules out androgen deficiency as a cause of reproductive failure. Given that fish reproductive behavior is jointly regulated by LH and androgens, this result provides a critical foundation for interpreting subsequent behavioral and hormonal defects. However, the manuscript neither states the rationale behind this experiment nor discusses its biological relevance. As a result, the data are presented in isolation, making it difficult for readers—especially those outside the field—to appreciate its relevance. I recommend that the authors provide background context, clarify the intent of the experiment, and discuss its implications within the framework of sex hormone signaling and reproductive behavior.

Response to Comment 7:

We thank the reviewer for this insightful comment regarding the interpretation of secondary sexual characteristics (SSCs). We agree that the observation of normal SSCs (Fig. 2d) is a critical finding that initially suggested intact androgen signaling, and we acknowledge that its presentation in isolation limited the reader's understanding of its full biological relevance. The primary rationale for analyzing SSCs was to determine if the reproductive failure in our mutants stemmed from incomplete sex differentiation (intersex/feminization) or a specific functional deficit in established males. The presence of normal tubercles and fin coloration confirmed that the mutants are phenotypically male, narrowing the cause of infertility to behavioral or physiological dysfunction. However, as the reviewer astutely noted, this created a paradox: the normal morphology implies intact androgen signaling, yet the complete loss of courtship behavior—a process driven by high androgen levels—suggests a deficiency.

To resolve this and clarify the biological implications within the framework of sex hormone signaling, we performed two decisive new experiments. First, contrary to the inference drawn solely from SSCs, our direct metabolomic quantification revealed that testicular testosterone and androstenedione levels are significantly reduced in *cckbrb*$^{-/-}$ mutants compared to wild-type controls (Supplementary Fig. S5). Second, to determine if this quantitative reduction was functionally relevant to the behavioral defect, we treated *cckbrb*$^{-/-}$ mutants with exogenous testosterone. Crucially, this treatment completely rescued the impaired courtship behaviors (Supplementary Fig. S6).

These combined findings provide a sophisticated nuance to our understanding of androgen signaling in teleosts: they suggest that the threshold of androgen signaling

required to maintain morphological SSCs is lower than that required to drive complex courtship behaviors. Therefore, while basal androgen levels in the mutants are sufficient for somatic masculinization, CCK signaling is essential for maintaining the high physiological levels necessary for reproductive behavior. We have revised the manuscript to explicitly state this rationale and discuss these findings, moving beyond the initial observation of SSCs to a verified model of androgen insufficiency-driven behavioral failure.

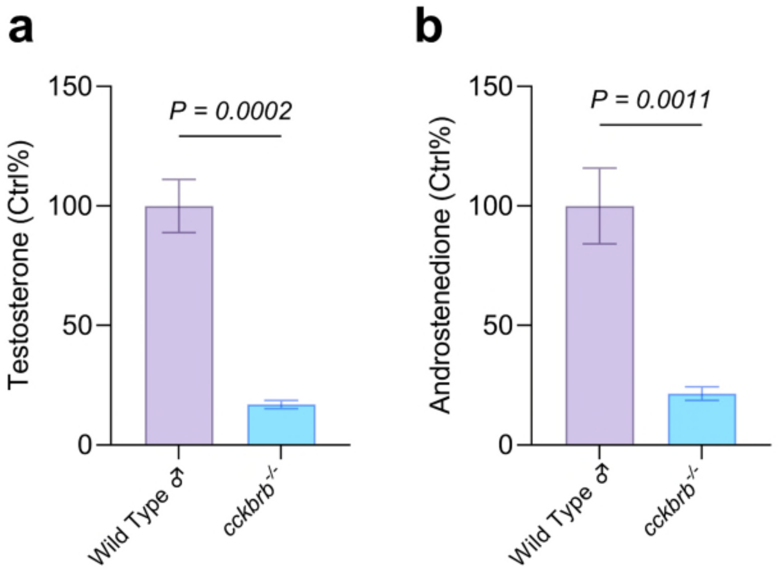

Supplemental Fig. S5 | Testicular androgen levels in *cckbrb*$^{-/-}$ zebrafish. The levels of key androgen hormones were quantified in the testes of *cckbrb*$^{-/-}$ and wild-type (WT) male zebrafish. **a**, Relative levels of testosterone in WT and *cckbrb*$^{-/-}$ testes. **b**, Relative levels of androstenedione in WT and *cckbrb*$^{-/-}$ testes. Androgen quantification was performed using metabolomic analysis. Data were normalized to the mean value of the Wild-type (WT) male testes group. Data are presented as the mean ± s.d. (standard deviation) for n= 3 independent biological replicates. Each biological replicate represents a pooled sample composed of the testes from ten individual male zebrafish.

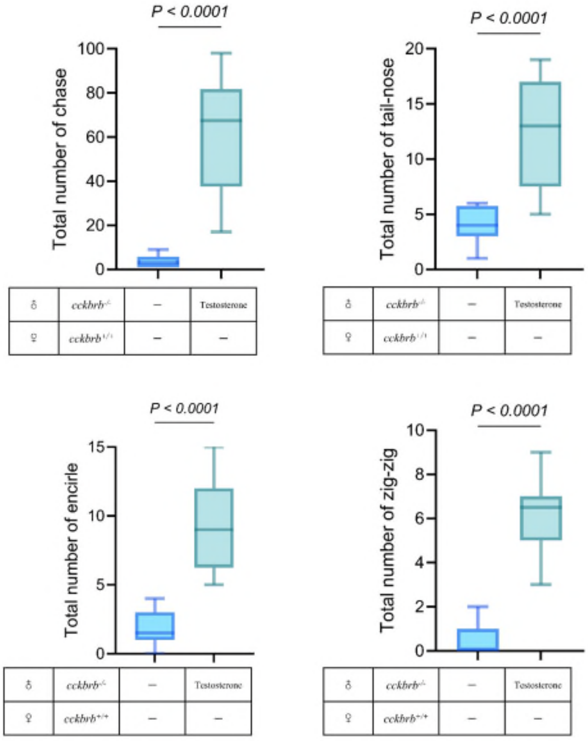

Supplemental Fig. S6 | Testosterone immersion rescues the impaired courtship behaviors in *cckbrb*$^{-/-}$ zebrafish. The effect of exogenous testosterone on male courtship behavior was assessed in wild-type (WT) and *cckbrb*$^{-/-}$ zebrafish. Male fish were treated with testosterone immersion prior to behavioral assessment. The figure shows the frequency of each courtship behavior (Chase, Tail-nose, Encircle and Zig-zag) performed by males of different genotypes and treatments during a 10-minute observation period. Data are presented using box plots, where the central line indicates the median, the box boundaries represent the interquartile range (IQR), and the whiskers denote the minimum and maximum (min-max) values. The sample size for each group is n= 24 independent pairings.

8. Line 199: Please note that there should be a space between numerical values and units (e.g., “5 dpf” instead of “5dpf”). This formatting issue appears multiple times throughout the manuscript, such as in Line 199. A thorough check and correction are recommended to ensure consistency and adherence to journal style.

Response to Comment 8:

We thank the reviewer for pointing out this critical formatting error. We acknowledge that the lack of a space between numerical values and units (e.g., “5dpf”) appeared frequently throughout the manuscript. We have performed a thorough, manuscript-wide check and corrected all instances of this formatting error to ensure consistency and strict adherence to the journal's style requirements.

9. Fig. 3: Again, for panels j-m in, each should include its own legend indicating the group represented by each bar, even if the groups are the same. Currently, only panel j provides this information. Similar omissions are found elsewhere in the manuscript. Please ensure all figure panels are self-contained, with clear labeling of group identities, to allow interpretation without cross-referencing.

Response to Comment 9:

We appreciate the reviewer for reiterating this important formatting requirement. We apologize for the omission of necessary, independent legends in Figure 3 (panels j-m) and elsewhere. We have thoroughly reviewed and corrected all figures in the revised manuscript to ensure that each panel is self-contained and includes its own clear, explicit legend identifying the experimental groups represented by each bar, thus allowing interpretation without cross-referencing.

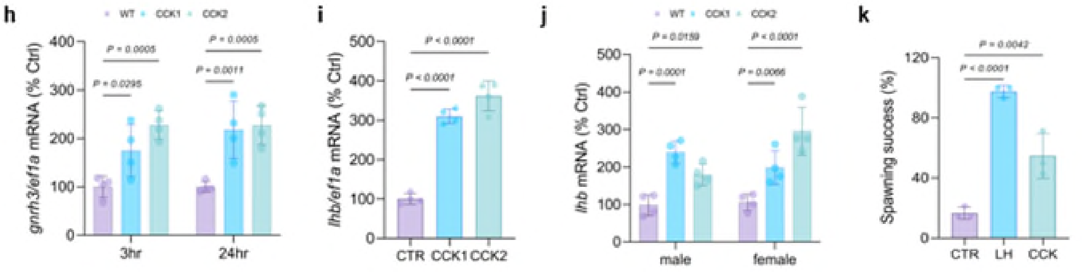

Fig. 3 h *gnrh3* mRNA levels in hypothalamic cells treated with CCK1 or CCK2 for 3 hours and 24 hours (*n*= 4). **i, j** Regulation of *lhb* expression. **i** *lhb* mRNA levels in primary pituitary cells treated with CCK1 or CCK2 for 24 hours. Data represent *n*= 4 independent biological replicates (each consisting of a pool of 3 pituitaries). **j** Pituitary *lhb* mRNA levels measured 24 hours after i.p. injection of CCK1 or CCK2. (Note: Assuming j follows the same pooling strategy as e; if so: Data represent *n*= 4 independent biological replicates, each consisting of a pool of 3 pituitaries). **k** Functional assessment of ovulation success rate (%) in female zebrafish 24 hours after i.p. injection of LH or CCK (*n*= 14 biologically independent animals from 3 independent experiments). Data are presented as mean ± s.e.m.

10. Fig. 3: Although the manuscript does not explicitly define "N" and "n", it appears that "n" refers to the number of biological replicates per experiment and "N" denotes the number of independent experimental repeats. However, there seems to be inconsistency in this usage. For instance, in Figure 3b, "N = 3, n = 4" is stated, yet only three data points are shown. In contrast, Figure 3l uses the same notation ("N = 3, n = 4"), but displays four data points. This discrepancy suggests inconsistent interpretation or presentation. Please clarify and, if necessary, correct the description to ensure accuracy and consistency.

Response to Comment 10:

We sincerely thank the reviewer for identifying this inconsistency and the potential for confusion regarding our statistical notation. We agree that the previous usage of "*N*" and "*n*" was ambiguous and inconsistent with the data points displayed. To ensure accuracy and consistency throughout the manuscript, we have standardized our reporting. We now exclusively use the lowercase "*n*" to denote the number of independent biological replicates used for statistical analysis. Where applicable, we also explicitly describe the sample composition (e.g., "each replicate consists of a pool of 3 pituitaries") to provide full transparency regarding the experimental design. We have corrected the legend for Figure 3 to align perfectly with the data points presented in the graphs.

Figure 3 | CCK regulates the synthesis and function of pituitary FSH and LH in zebrafish. a Representative immunohistochemical staining of LH and FSH proteins in the pituitary of WT, *cck1*$^{-/-}$;*cck2*$^{-/-}$, and *cckbrb*$^{-/-}$ zebrafish. **b** Relative mRNA expression of *lhb* and *fshb* in pituitary tissue from different knockout models compared to WT. Data are derived from *n*= 4 independent biological replicates, where each replicate consists of a pool of 3 pituitaries. **c** Single-cell RNA sequencing (scRNA-seq) visualization of zebrafish pituitary cell clusters. **d** Sub-clustering and trajectory analysis of the gonadotrope cell population. **e, f** Regulation of *fshb* expression. **e** Pituitary *fshb* mRNA levels measured 24 hours after intraperitoneal (i.p.) injection of CCK1 or CCK2. Data represent *n*= 4 independent biological replicates (each consisting of a pool of 3 pituitaries). **f** *fshb* mRNA levels in primary pituitary cells treated with CCK1 or CCK2 for 24 hours (*n*= 4). **g, h** Analysis of the upstream hypothalamic *gnrh3* response. **g** Hypothalamic *gnrh3* expression in CCK ligand and receptor knockout models (*n*= 6). **h** *gnrh3* mRNA levels in hypothalamic cells treated with CCK1 or CCK2 for 3 hours and 24 hours (*n*= 4). **i, j** Regulation of *lhb* expression. **i** *lhb* mRNA levels in primary pituitary cells treated with CCK1 or CCK2 for 24 hours. Data represent *n*= 4 independent biological replicates (each consisting of a pool of 3 pituitaries). **j** Pituitary *lhb* mRNA levels measured 24 hours after i.p. injection of CCK1 or CCK2. (Note: Assuming j follows the same pooling strategy as e; if so: Data represent *n*= 4 independent biological replicates, each consisting of a pool of 3 pituitaries). **k** Functional assessment of ovulation success rate (%) in female zebrafish 24 hours after i.p. injection of LH or CCK (*n*= 14 biologically independent animals from 3 independent experiments). Data are presented as mean ± s.e.m.

11. Fig 3i: The labels "40X" and "100X" refer to objective magnifications and are not typically included directly in figure panels. Relative image size should instead be indicated using clearly visible scale bars. In the current manuscript, the scale bars in several histological and immunofluorescence images (e.g., H&E and IF) are too small to be easily read. Please enlarge the scale bars to a legible size and ensure they are clearly visible across all relevant figures.

Response to Comment 11:

We thank the reviewer for this helpful suggestion regarding image presentation. We have removed the specific panel mentioned from the revised manuscript. Furthermore, in accordance with the reviewer's advice, we have removed all "40X/100X" magnification labels and ensured that clearly legible scale bars are present in all histological and immunofluorescence images (e.g., H&E and IF).

As these corrections were applied across the entire manuscript, we have provided representative examples below to demonstrate the improved legibility of the scale bars. Please refer to the revised manuscript for the complete set of updated figures.

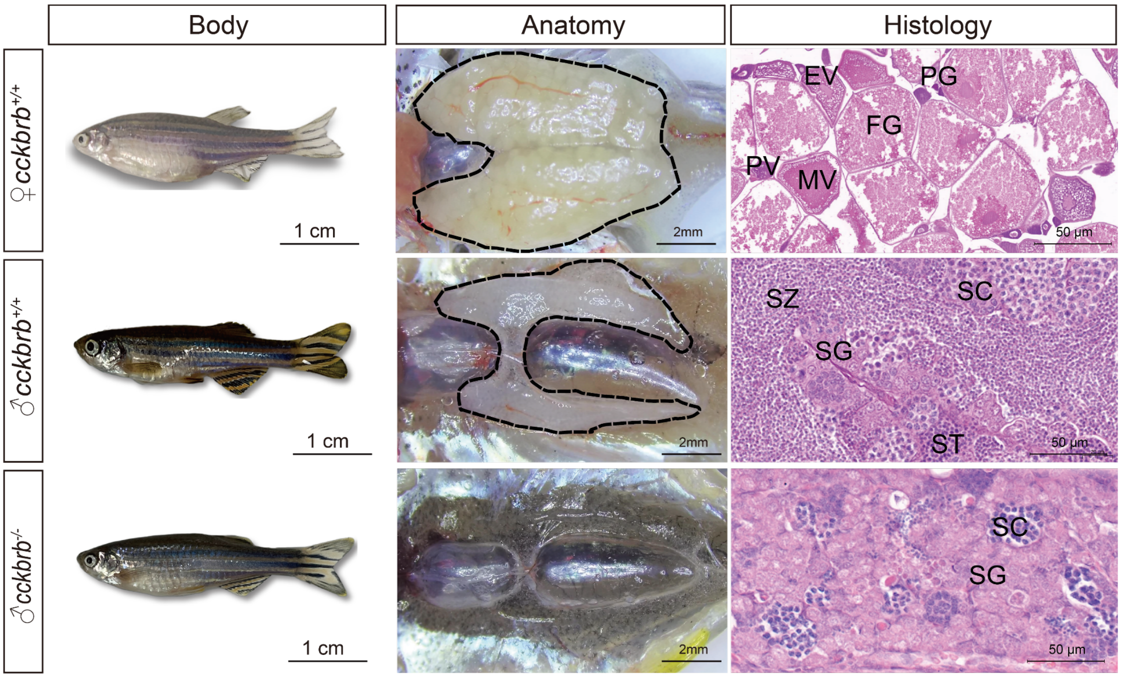

Fig. 3b Gonadal morphology and histology in 5-month-old *cckbrb*$^{+/+}$ and *cckbrb*$^{-/-}$ zebrafish.

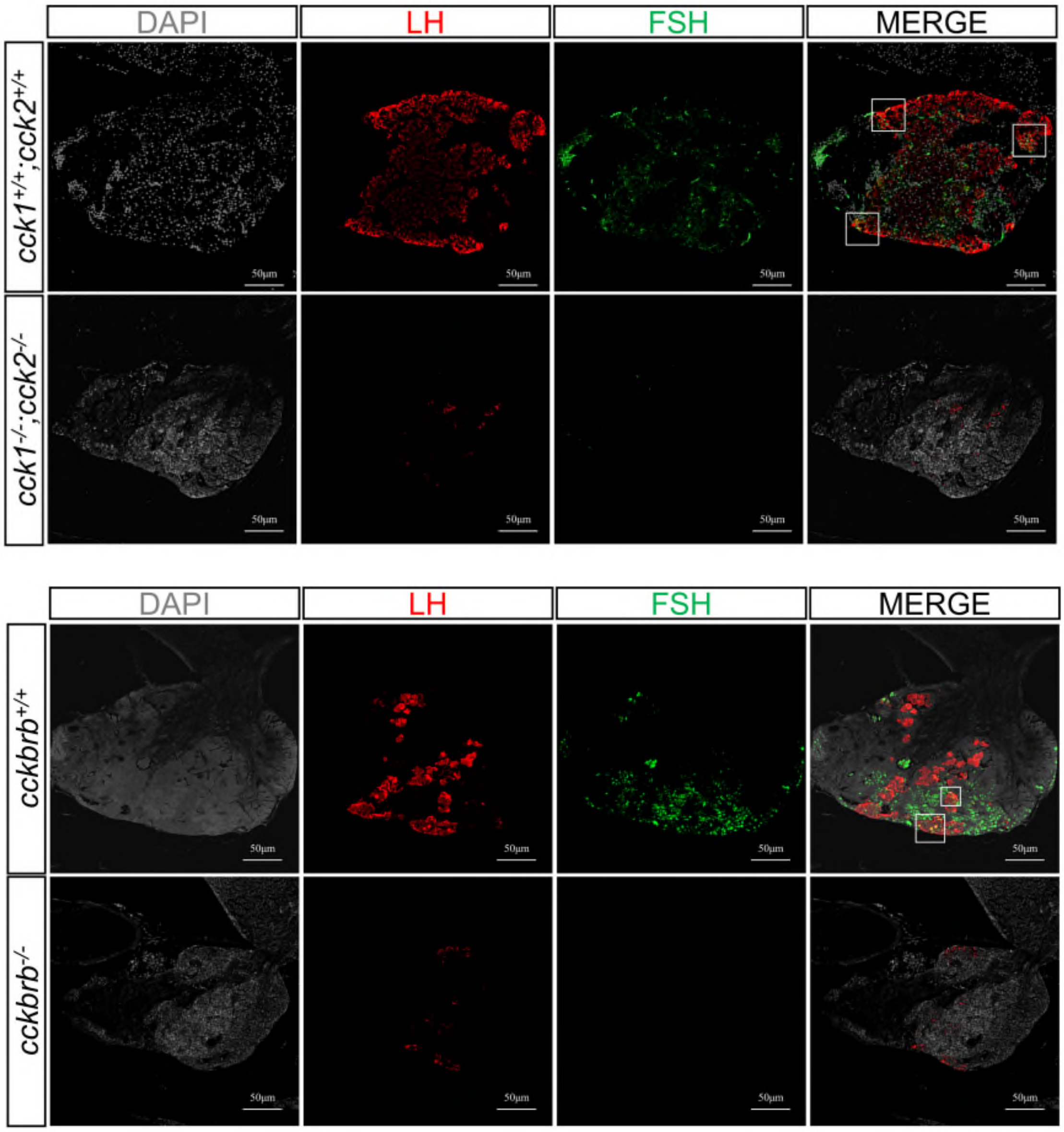

Fig. 3a Representative immunohistochemical staining of LH and FSH proteins in the pituitary of WT, *cck1*$^{-/-}$;*cck2*$^{-/-}$, and *cckbrb*$^{-/-}$ zebrafish.

12. Immunofluorescence (IF) results should be quantified and supported with statistical analysis, particularly in figures such as Fig. S12, S13, and 4c. Quantification would enhance the rigor and interpretability of the data.

Response to Comment 12:

We agree with the reviewer that quantification is essential to ensure data rigor and interpretability. To address this, we adopted a strategy that prioritizes the most accurate quantification method available for each developmental stage.

Quantitative Validation for Juvenile and Adult Stages:

For the analysis of specific protein markers in later developmental stages, we utilized Western Blotting (WB) instead of IF intensity analysis. WB is widely regarded as the "gold standard" for protein quantification due to its superior specificity and semi-quantitative capability.

Histone H3 (Proliferation): We performed WB analysis on gonads from *cckbrb*$^{-/-}$ mutants and WT controls at 45, 90, and 180 dpf. The results show a consistent reduction in Histone H3 levels in mutants, confirming the proliferation defect (**Supplementary Fig. S21**).

SYCP3 (Meiosis): We quantified SYCP3 expression in *fshb*$^{-/-}$, *lhb*$^{-/-}$;*fshb*$^{-/-}$, and *cckbrb*$^{-/-}$ zebrafish at 45 dpf via WB. This analysis confirms the significant reduction of meiotic markers in these lines compared to WT (**Supplementary Fig. S23**).

Technical Limitations at 14 dpf (Fig. 4c):

Regarding the early larval stage (14 dpf) shown in Fig. 4c, we attempted to perform WB validation but encountered insurmountable technical limitations. At 14 dpf, the primordial gonads are microscopic and cannot be feasibly dissected from the larvae. Furthermore, using whole-larvae lysates is not viable because the target protein (Histone H3) is ubiquitous; the overwhelming signal from somatic tissues would mask the specific signal from the nascent gonads, rendering the results uninterpretable.

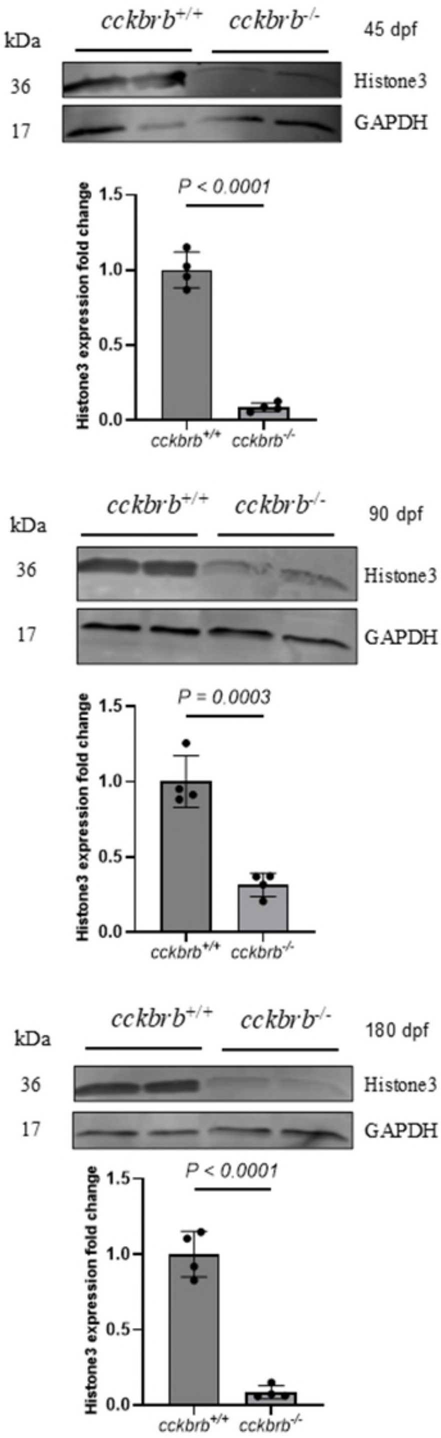

Supplementary Fig. S21 | Representative western blots showing the expression levels of Histone H3 in gonads from Wild-type (WT) and *cckbrb*$^{-/-}$ zebrafish at 45, 90, and 180 days post fertilization (dpf). GAPDH served as a loading control. The experiment was repeated three times independently with similar results. Source data are provided as a Source Data file.

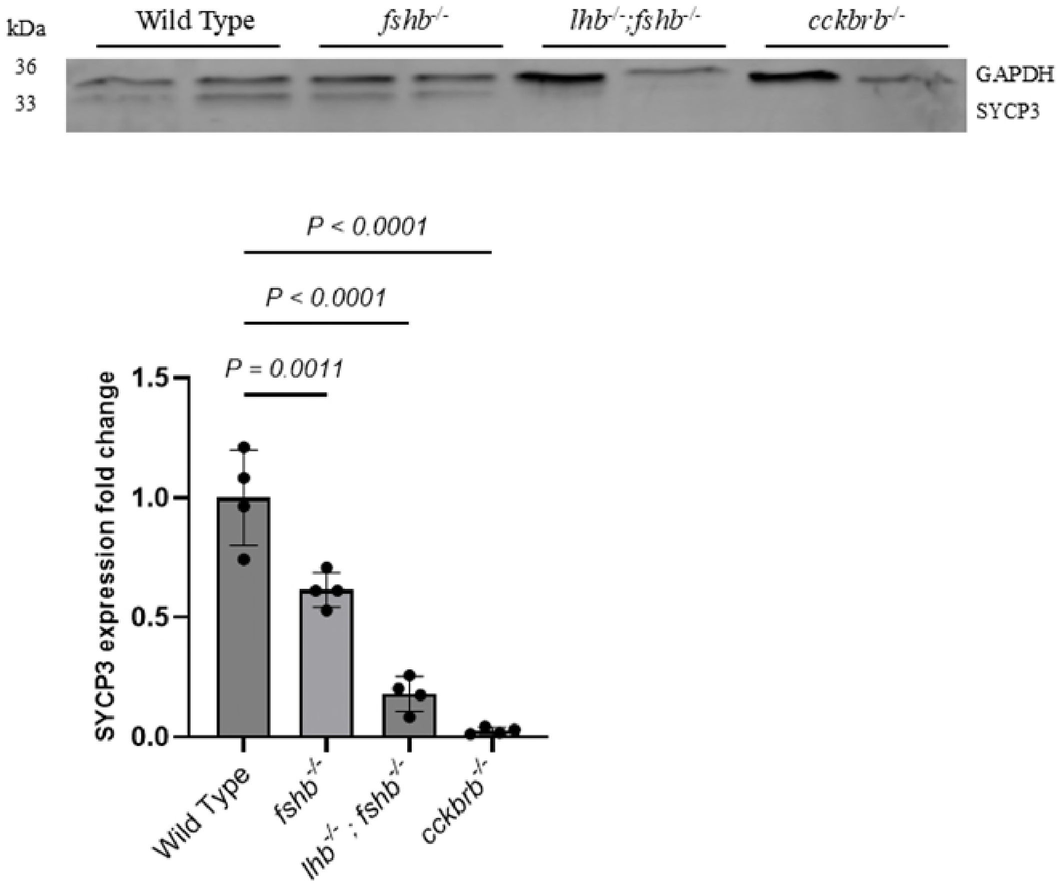

Supplementary Fig. S23 | Western blot analysis of the meiotic marker SYCP3 in WT, *fshb*$^{-/-}$, *lhb*$^{-/-}$;*fshb*$^{-/-}$, and *cckbrb*$^{-/-}$ zebrafish. Representative western blots showing SYCP3 protein expression in gonads from WT, *fshb*$^{-/-}$, *lhb*$^{-/-}$;*fshb*$^{-/-}$, and *cckbrb*$^{-/-}$ zebrafish zebrafish at 45 days post fertilization (dpf). GAPDH served as a loading control. The experiment was repeated three times independently with similar results. Source data are provided as a Source Data file.

13. Line 235 and 472: The abbreviation "hpf" (hours post-fertilization) is defined more than once in the manuscript. Please retain the definition only at its first occurrence and use the abbreviation consistently thereafter to avoid redundancy.

Response to Comment 13:

We appreciate the reviewer identifying this redundancy in abbreviation definition. We have performed a thorough, manuscript-wide check and correction to ensure that the definition of hpf (hours post-fertilization) is retained only at its first occurrence and that the abbreviation is used consistently throughout the remainder of the manuscript.

Editorial Note: Supplemental Fig. S4a in this Peer Review File is reproduced with permission from BioRender, Created in BioRender. Li, H. (2026) https://BioRender.com/qm1v0y9

14. Line 442-451: The study reports impaired mating behavior in cck mutants but does not clarify whether their general locomotor activity is intact. It would strengthen the interpretation to confirm that reduced mating behavior is not due to non-specific motor deficits.

Response to Comment 14:

We thank the reviewer for raising this important point. To definitively rule out the possibility that the impaired mating behavior observed in our mutants was due to non-specific motor deficits, we performed a quantitative assessment of general locomotor activity. We evaluated the swimming activity of wild-type and mutant zebrafish (*cck1*$^{-/-}$;*cck2*$^{-/-}$, and *cckbrb*$^{-/-}$) under a feeding stimulus. The total swimming distance was measured and analyzed using tracking software. The results, now presented in Supplementary Fig. S4, show no significant difference in locomotor activity between the mutant and control groups. This finding confirms that the profound reduction in courtship behaviors is not attributable to a general impairment in motor function or swimming capability, thereby strengthening the interpretation that the behavioral deficit is specific to the reproductive context and is a direct consequence of disrupted CCK signaling.

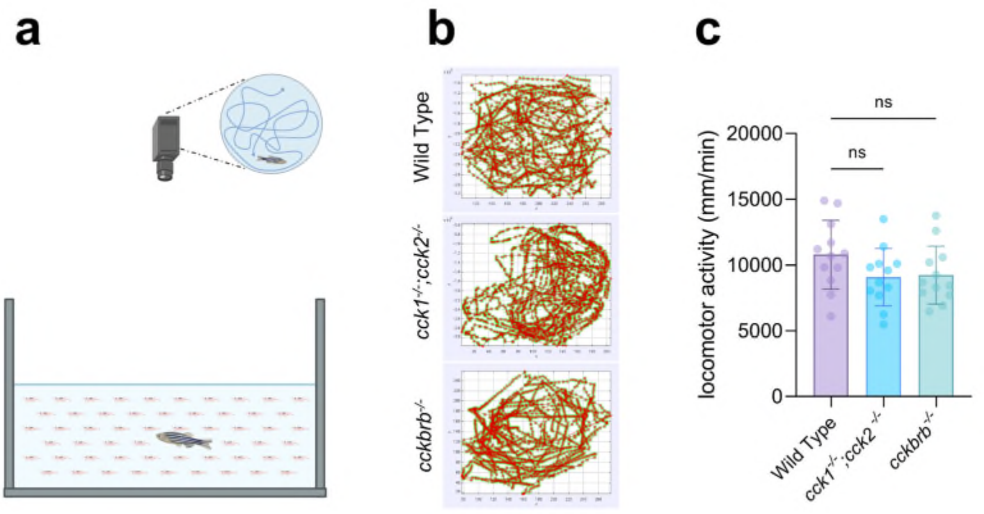

Supplemental Fig. S4 | Locomotor activity driven by feeding stimulus in in *cck1*$^{-/-}$;*cck2*$^{-/-}$ and *cckbrb*$^{-/-}$ zebrafish. a Experimental scheme. **b** Representative swimming traces of wild-type, *cck1*$^{-/-}$;*cck2*$^{-/-}$ and *cckbrb*$^{-/-}$ zebrafish. **c** Quantification of feeding-driven locomotor activity. Data are presented as mean ± s.e.m. for *n*= 12 independent fish per group.

References

1. Farrell, J. A., Wang, Y., Riesenfeld, S. J. et al. Single-cell reconstruction of developmental trajectories during zebrafish embryogenesis. *Science*, **360**, eaar3131(2018).
2. Liew W C, Bartfai R, Lim Z, et al. Polygenic sex determination system in zebrafish. PloS one, 2012, 7(4): e34397.
3. Matsuda, M., Nagahama, Y., Shinomiya, A. et al. DMY is a Y-specific DM-domain gene required for male development in the medaka fish. Nature, 2002, 417, 559–563.
4. Moore E C, Roberts R B. Polygenic sex determination. Current Biology, 2013, 23(12): R510-R512.
5. Tanaka, S., Yu, Y., Levavi-Sivan, B., Zmora, N. & Zohar, Y. GnRH—gonadotropes interactions revealed by pituitary single-cell transcriptomics in zebrafish. *Endocrinology.* **165**, bqae151 (2024).
6. Uehara, S. K. et al. Identification of the FSH-RH as the other gonadotropin-releasing hormone. *Nat. Commun.* **15**, 5342 (2024).

Reviewer #3 (Remarks to the Author):

In this manuscript, the authors provide a detailed analysis of the effects of knocking out the CCK system on reproductive functions in zebrafish. While Hollander-Cohen previously performed knockouts in zebrafish, their study focused only on CCK receptors. In contrast, this manuscript includes ligand knockouts and thus provides a more detailed analysis of the effects on FSH.

In addition, the manuscript proposes a completely new perspective in which CCK regulates reproduction via gonadotropin-independent pathways. However, this analysis is limited to knockouts and single-cell RNA-seq expression profiling, with only minimal IHC, and does not investigate which CCK neurons act on which cell expressing CCK receptor. As such, the conclusion that hypothalamic CCK directly affects the gonads, as depicted in the schematic, is not supported by the evidence.

Furthermore, the authors' claim that CCK regulates LH contradicts previously published findings, but the experimental evidence provided in this manuscript is insufficient to support this assertion.

In conclusion, the reviewer considers that the manuscript lacks sufficient evidence to support many of its claims. Specifically, the main story relies on whole-body knockouts, which could have multiple indirect effects, and co-expression results from single-cell RNA-seq, which are not definitive. Without more detailed morphological analyses or in vitro physiological experiments, these conclusions cannot be drawn. To make definitive claims from single-cell RNA-seq, reproducibility should be confirmed using more conclusive methods such as double in situ hybridization. Single-cell RNA-seq does not necessarily reflect in vivo expression accurately, and using it alone to support conclusions risks error.

Response to Comment 1:

We sincerely thank the reviewer for their thorough and constructive critique. We acknowledge the initial concerns regarding the mechanistic depth of our study and the reliance on correlative data from whole-body knockouts and scRNA-seq. In direct response, we have undertaken an extensive series of new experiments designed to transform key correlative observations into causally supported conclusions. The revisions below address each major concern with decisive new evidence.

(1). Upgrading methodological rigor and externally validating key cell populations

The reviewer rightly emphasized that scRNA-seq findings require orthogonal validation. We have moved beyond correlation by supplementing our data with direct morphological evidence and leveraging strong external consensus.

Definitive receptor localization: For all critical receptor localizations (e.g., CCKBRb on LH-producing cells), we now provide high-resolution dual FISH/IHC data, offering direct *in situ* visual proof (Supplementary Fig. S15).

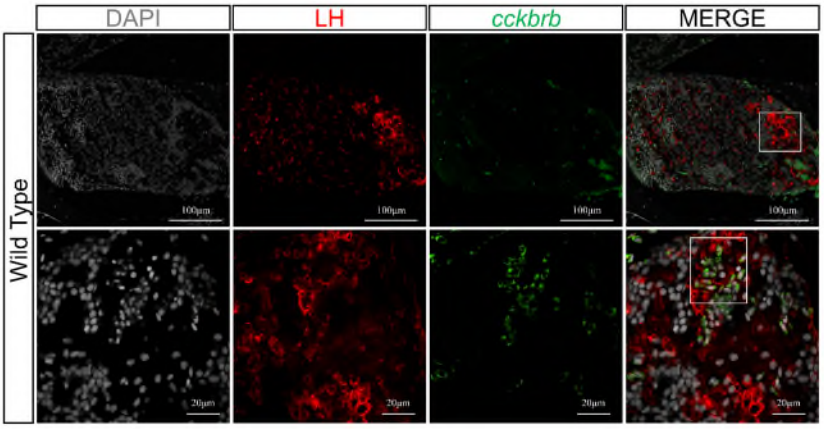

Supplementary Fig. S15 | Co-localization of *cckbrb* mRNA and LH protein in the adult male zebrafish pituitary gland. *cckbrb* mRNA was detected using a fluorescent probe (via Fluorescence *In Situ* Hybridization), and LH protein was visualized via immunohistochemistry (IHC).

External validation of dual-expressing gonadotropes: The existence of the fshb+/lhb+ cell population is no longer solely our scRNA-seq finding. It is now strongly corroborated by independent, recent studies. Sakura Tanaka et al. (2024) confirmed this population in zebrafish using both scRNA-seq and protein co-localization. Furthermore, quantitative FACS data from Hollander-Cohen et al. (2021) reported a substantial proportion (7.1%) of gonadotropes co-expressing LH and FSH, underscoring its physiological relevance. Furthermore, we confirmed this co-expression at the protein level using immunohistochemistry, providing strong external validation for the phenomenon. These optimized images unambiguously demonstrate the co-localization of LH and FSH proteins *in vivo* (Fig. 3a).

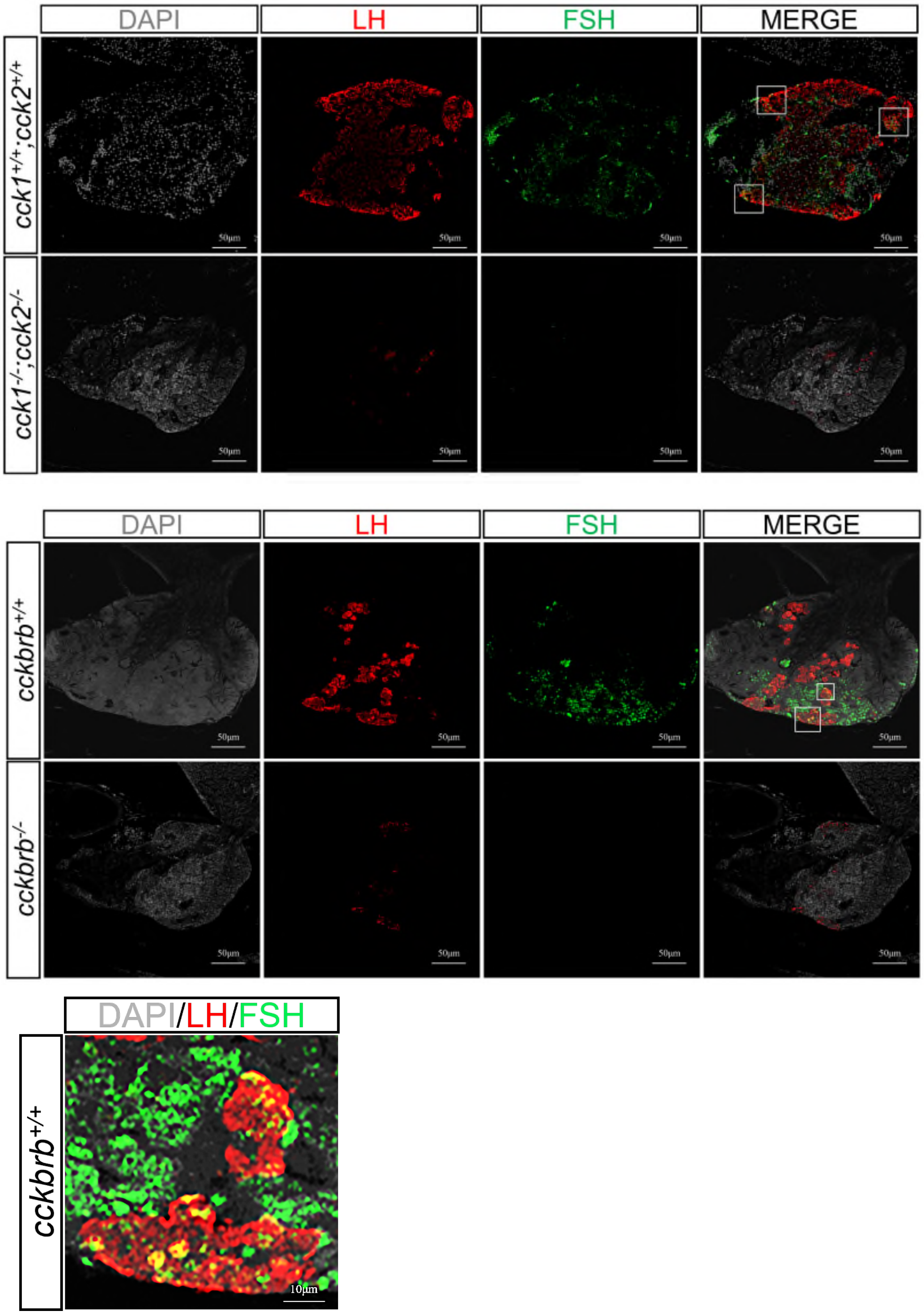

Fig. 3a Representative immunohistochemical staining of LH and FSH proteins in the pituitary of WT, *cck1*$^{-/-}$;*cck2*$^{-/-}$, and *cckbrb*$^{-/-}$ zebrafish.

(2). Providing mechanistic proof for LH regulation

To resolve the discrepancy with previous studies and prove CCK's role in LH regulation, we provide a multi-tiered functional validation that isolates the effect from systemic confounders.

Direct pituitary action: Our *in vitro* primary pituitary cell culture experiments demonstrate that CCK directly stimulates *lhb* transcription (Fig. 3i), isolating a direct effect and ruling out indirect systemic feedback. We note that Hollander-Cohen et al. previously identified the pituitary CCK receptor (which they termed *cckbra*, but corresponds to the same *cckbrb* locus we studied) and localized it specifically to FSH cells. Our findings refine this observation rather than contradict it. Our scRNA-seq and FISH/IHC data confirm that *cckbrb* is indeed enriched in the FSH cell lineage, but specifically within the subpopulation that co-expresses LH (Fig. 3d; Supplementary Fig. S15). Since these cells express high levels of *fshb*, they were classified simply as "FSH cells" in the Hollander-Cohen study. However, our data reveals that these receptor-positive cells also actively produce LH. Therefore, the receptor is indeed present on a specific subset of LH-producing cells (the dual-hormone progenitors), providing the necessary structural basis for the direct regulation of LH by CCK.

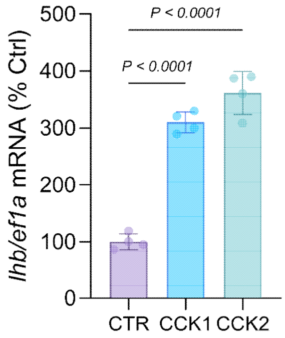

Fig. 3i. qPCR analysis of *lhb* gene expression in pituitary cells treated with CCK1 or CCK2 for 24 hours (*n*= 4)

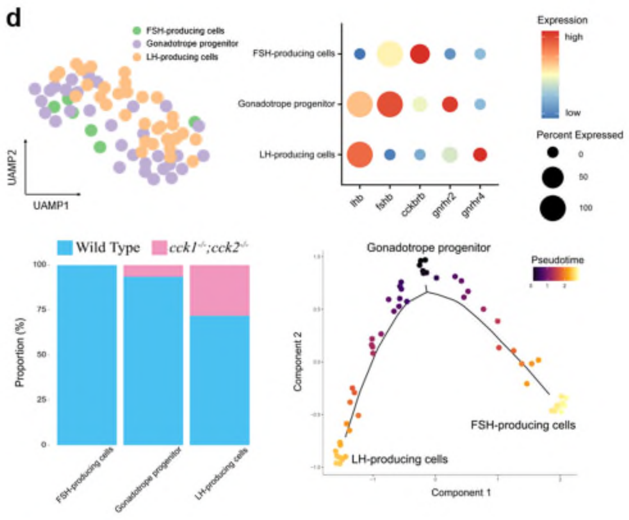

Fig. 3d Sub-clustering and trajectory analysis of the gonadotrope cell population.

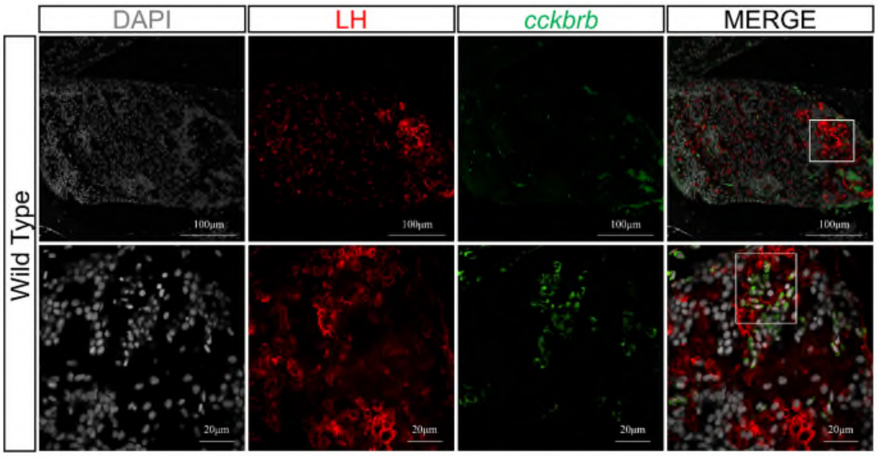

Supplementay Fig. S15 | Co-localization of *cckbrb* mRNA and LH protein in the adult male zebrafish pituitary gland. *cckbrb* mRNA was detected using a fluorescent probe (via Fluorescence In Situ Hybridization), and LH protein was visualized via immunohistochemistry (IHC).

Editorial Note: Supplementary Fig. S14a in this Peer Review File is reproduced with permission from BioRender, Created in BioRender. Li, H. (2026) https://BioRender.com/hgl9icn

Functional validation of the GnRH3 pathway: Using a novel co-culture system with a GnRH3-neutralizing antibody, we show that the CCK-induced *lhb* expression is significantly attenuated (Supplementary Fig. S14). This provides direct functional evidence for the physiological relevance of the CCK-GnRH3-LH axis.

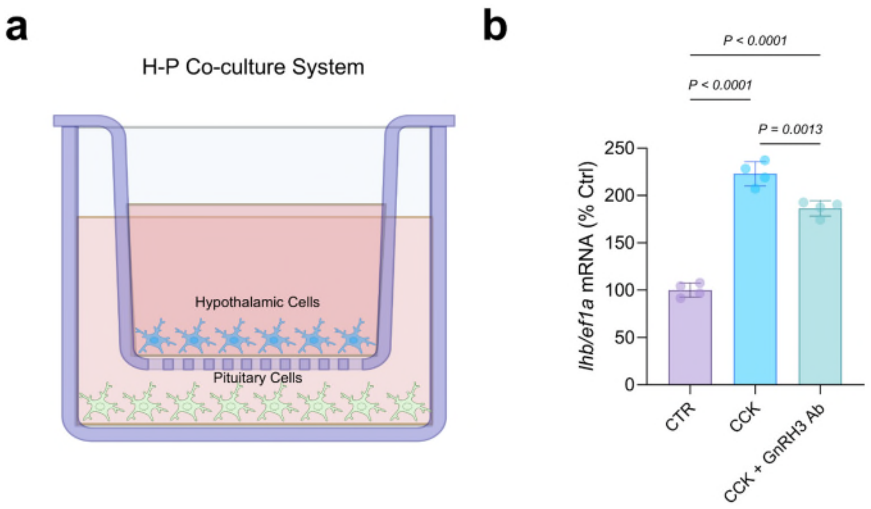

Supplementary Fig. S14 | Hypothalamic-Pituitary (H-P) co-culture system and functional assessment of dual CCK regulation. a Schematic diagram of the H-P co-culture system. Hypothalamic cells are cultured on the transwell insert membrane, and pituitary cells are cultured in the lower chamber, facilitating the transfer of soluble factors. **b** qPCR analysis of *lhb* mRNA expression in pituitary cells from the co-culture system following treatment with Control (Ctrl), CCK only (CCK), and CCK with GnRH3 blocking antibody (CCK + GnRH3 Ab). Data are presented as mean ± s.e.m. (standard error of the mean) for *n*= 4 independent experiments.

(3). Substantiating the gonadotropin-independent pathway and correcting the model

We agree that the model of hypothalamic CCK directly acting on the gonad required clarification and substantiation. Our new data elucidate the mechanism and precise source of CCK.

Causal link in somatic cell survival: We identified that CCK loss triggers gonadal somatic cell apoptosis. Most critically, genetic knockout of *tp53* rescued both somatic cell apoptosis and meiotic initiation in *cckbrb* mutants (Fig. 5g, 5h), providing powerful causal evidence for this gonadotropin-independent function.

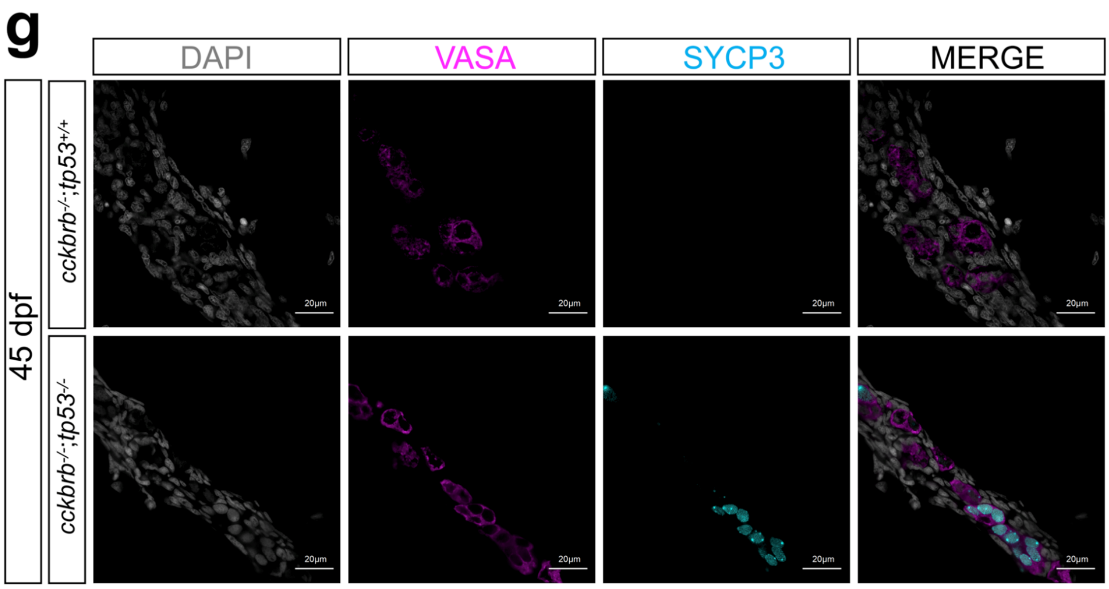

Fig. 5g Immunofluorescence of SYCP3 and VASA in testes at 45 dpf.

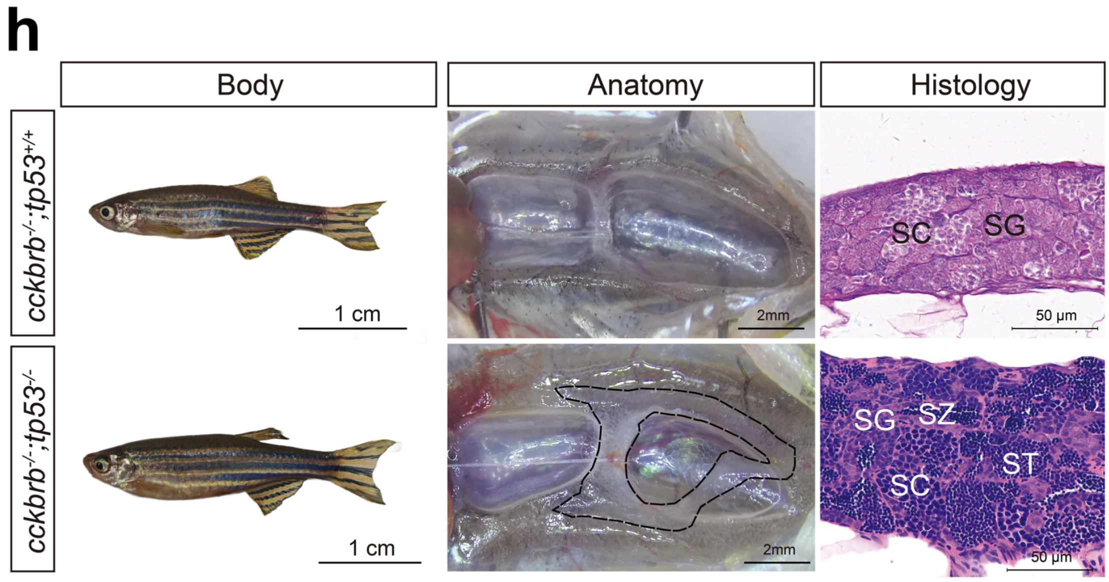

Fig. 5h Gross morphology and histological analysis (H&E) of gonads.

Precise source of CCK for early gonadal action: In response to the valid criticism regarding the schematic, spatial expression analysis revealed that during early development, the CCK influencing the gonad originates from local tissue, not the hypothalamus (Fig. 4d,e; Fig. R5). We have therefore revised our working model (Fig. 5i) to accurately distinguish between CCK's neuroendocrine role (hypothalamus-pituitary) and its local paracrine role (local source-gonad).

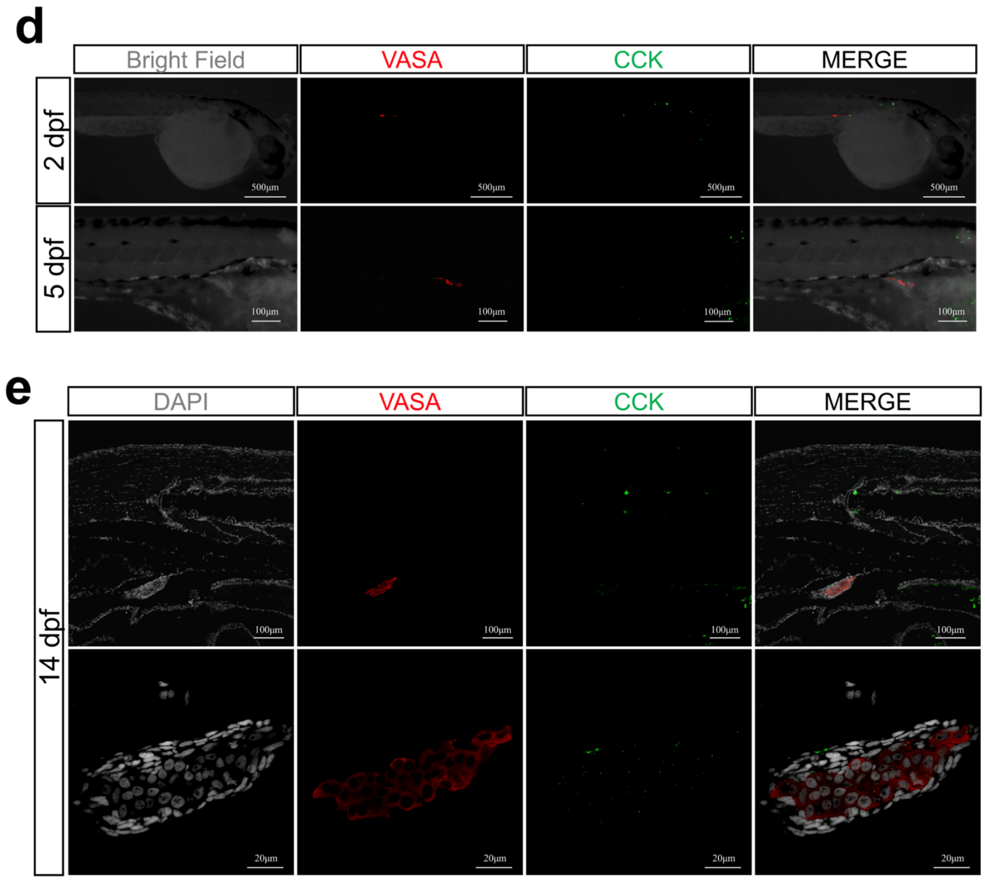

Fig. 4. (**d)** Whole-mount immunofluorescence co-staining of VASA (germ cell marker, red) and CCK (green) in zebrafish larvae at 2 dpf and 5 dpf. Merged images show the spatial relationship between CCK-expressing cells and migrating PGCs. (**e)** Immunofluorescence of CCK (green) and VASA (red) in 14 dpf gonads reveals CCK expression in somatic cells surrounding germ cells.

Editorial Note: Fig. 5i in this Peer Review File is reproduced with permission from BioRender, Created in BioRender. Li, H. (2026) https://BioRender.com/o68vxgm

[Figure Redacted]

Fig. R5 | Negligible expression of *cckbrb* in the zebrafish PGC lineage during early embryogenesis. a, b Visualization of the single-cell developmental trajectory from Farrell et al. (2018) (Source: https://singlecell.broadinstitute.org/single_cell/study/SCP162). The UMAP plots display cell clusters colored by (**a**) lineage identity (with PGCs indicated) and (**b**) developmental stage (from High to 6-somite). **c**, Expression distribution of *cckbrb* mapped onto the trajectory. The green arrow indicates the PGC cluster. Note that *cckbrb* expression is extremely sparse, detected in only 3 cells at the 50% epiboly stage and 1 c ell at the 90% epiboly stage across the entire dataset (Log2 expression values: 1.96, 1.88 , 1.54, 0.94). This lack of robust expression in the PGC lineage supports the hypothesis t hat CCK regulation of PGCs is likely indirect.

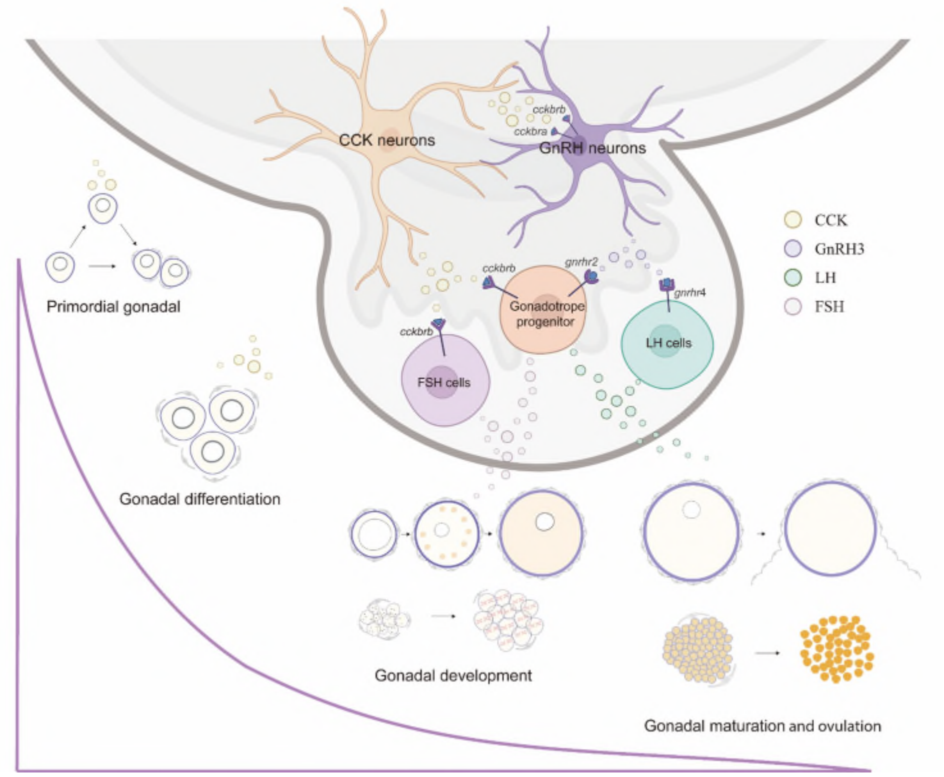

Fig. 5i Schematic diagram depicting the stage-specific regulatory network of CCK signaling during gonadal development. The model illustrates CCK's involvement in primordial germ cell (PGC) proliferation during early formation, the maintenance of somatic cell integrity and meiotic initiation during differentiation, and the regulation of pituitary FSH and LH synthesis (involving both direct pituitary action and indirect hypothalamic *gnrh3* modulation) to support gametogenesis and ovulation.

Technical issues:

• All RNA co-localization is discussed based solely on single-cell RNA-seq data. This can be misleading, as dissociation alters expression profiles and may not reflect physiological conditions. For example, in Figure 3a, histological data show distinct localization of LH and FSH cells, but single-cell RNA-seq suggests substantial co-localization. As reported in medaka (Fontaine et al., 2020, J. Endocrinol.), dissociation changes lh and fsh expression profiles. Thus, double in situ hybridization or other methods are needed to confirm these results. The cells labeled as "progenitors" in Figure 3d may not be progenitors, but rather artifacts caused by dissociation for single-cell RNA-seq. The authors should carefully compare these data with double-label histology results and consider that in vivo bihormonal cells are likely very rare.

Response to Comment 2:

We sincerely thank the reviewer for this rigorous technical comment. We fully agree that single-cell RNA-seq data, which requires tissue dissociation, can introduce artifacts and does not always reflect the native physiological state. The potential for dissociation-induced stress to alter gene expression profiles (as noted in medaka by Fontaine et al., 2020) is a valid concern that requires definitive validation. To address this and rule out the possibility of scRNA-seq artifacts, we have performed the requested validation using in situ methods that preserve tissue integrity:

Protein-Level Confirmation *In Vivo* (Ruling out Artifacts): We performed double immunofluorescence staining on intact pituitary sections. As shown in the revised Fig. 3a, high-resolution confocal microscopy unambiguously reveals a subpopulation of gonadotropes that co-express both FSH and LH proteins. Since this experiment was performed on fixed tissue without dissociation, it confirms that the co-expression is a genuine physiological phenomenon and not a transcriptomic artifact induced by cell isolation.

Independent External Validation: The existence of this specific dual-hormone population has been independently confirmed by a very recent study. Tanaka et al. (Endocrinology, 2024) utilized both scRNA-seq and immunohistochemistry to identify and validate a "progonadotrope" population in zebrafish that co-expresses FSH and LH (Table R1; Fig. R4). This external validation from a separate laboratory strongly suggests that these cells are a conserved feature of the zebrafish pituitary rather than a technique-specific artifact.

Identity of "Progenitors": Regarding the "progenitor" classification in Fig. 3d**,** our pseudotime trajectory analysis places these co-expressing cells specifically at the

developmental branch point preceding the differentiation of distinct FSH and LH lineages. While we acknowledge they may represent a transitional state, the combination of their stemness gene expression profile (Supplementary Fig. S8) and their position in the trajectory supports their identity as a precursor pool.

Conclusion: By combining our new protein-level evidence with independent literature support, we have robustly confirmed that the FSH/LH co-expressing cells are bona fide physiological entities. We have revised the manuscript to include these validation data and to discuss the potential for dissociation effects more carefully, citing the relevant literature.

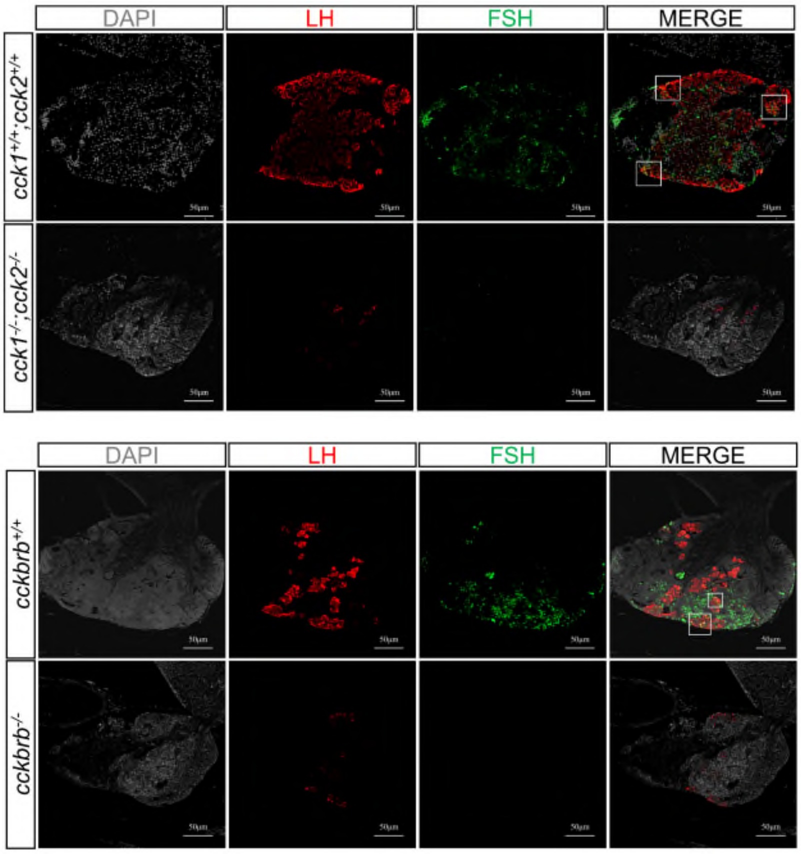

Fig. 3a Representative immunohistochemical staining of LH and FSH proteins in the pituitary of WT, *cck1*$^{-/-}$;*cck2*$^{-/-}$, and *cckbrb*$^{-/-}$ zebrafish.

Table R1. Single-cell RNA sequencing *lhb* and *fshb* transcript levels in LH and FSH gonadotropes (Tanaka et al., 2024).

[Table Redacted]

[Figure Redacted]

Figure R4. Pituitaries were subjected to double labeling in situ hybridization. *lhb* mRNA (red), *fshb* mRNA (green), merged image to analyze colocalization of *lhb* and *fshb*. (Tanaka et al., 2024).

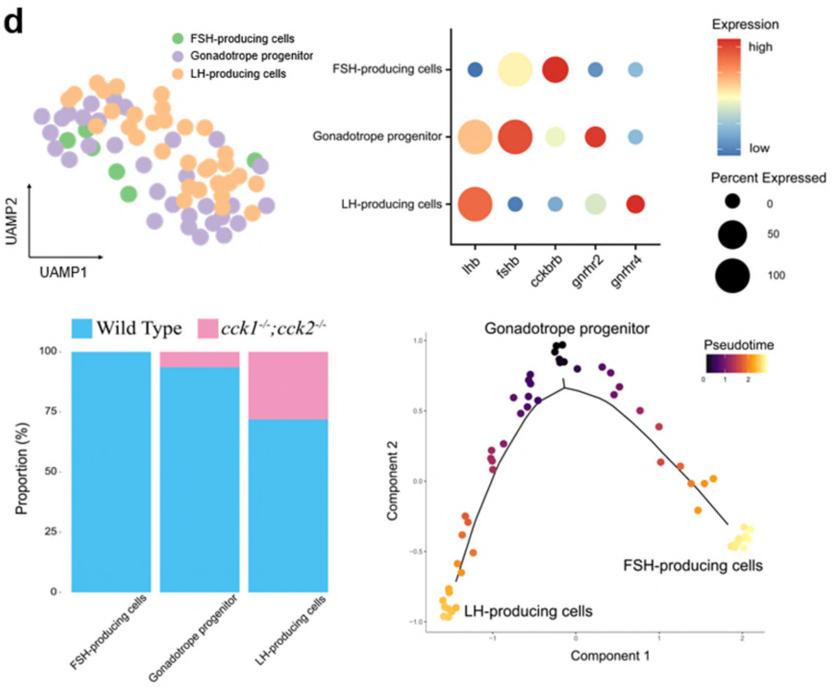

Fig. 3d Sub-clustering and trajectory analysis of the gonadotrope cell population.

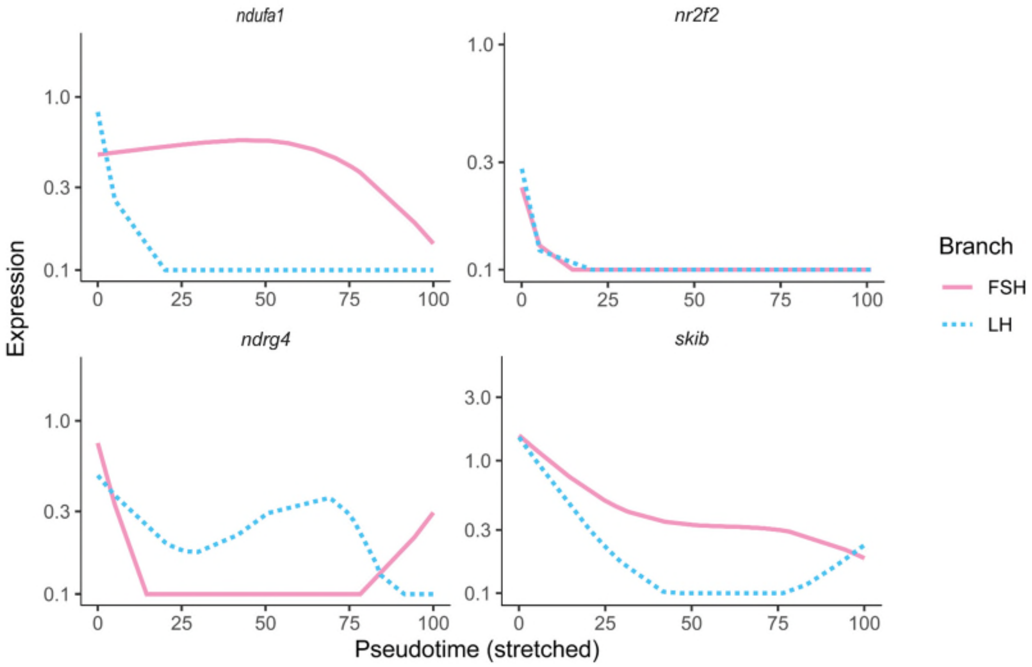

Supplementary Fig. S8 Dynamics of stemness gene expression during gonadotrope differentiation. The figure shows the relative expression levels of the stemness-related genes *ndufa1*, *nr2f2*, *ndrg4*, and *skib* across the pseudotime trajectory of gonadotrope differentiation, determined by pseudotime analysis. The two trajectories are shown as FSH (pink solid line) and LH (blue dotted line) differentiation branches.

Comments on CCK regulation of LH and GnRH:

• The claim that CCK regulates LH contradicts previous experiments in the same zebrafish line (Hollander-Cohen, 2024, eLife), where LH cells did not express cckbr, and Ca^{2+} imaging or release experiments showed no significant effect.

• Knockouts reduce FSH and consequently gonadal estrogen, which could indirectly attenuate the GnRH-LH pathway. Gonadal estrogen is established to stimulate this pathway (Huggard-Nelson et al., 2002; Li et al., 2018). Uehara et al., 2024 observed similar LH reductions in intact fish in medaka, while knockout of cckbr in gonadectomized fish did not affect LH. If the authors claim that CCK regulates LH, they must show this effect persists in gonadectomized fish.

• The assertion that GnRH neurons express cckbr is based on hypothalamic single-cell RNA-seq, but the main population of GnRH3 neurons resides in the POA. Even if double in situ hybridization confirmed co-localization, simply having receptors does not establish functional importance; targeted KO or electrophysiological analysis of GnRH3 neurons would be required.

• The claim that CCK injection induces ovulation likely reflects indirect effects via FSH-stimulated estrogen, rather than a direct action on the gonads, and cannot be stated as "CCK facilitates gonadal maturation and ovulation via its dual regulation (direct and indirect) of LH synthesis and secretion (Line 179-180)." These conclusions should be led by more careful observations.

Response to Comment 3:

We thank the reviewer for these rigorous and insightful comments regarding the regulation of LH and GnRH. We address these critical points with new data and clarifications below:

(1). Discrepancy with Hollander-Cohen et al. (2024): The reviewer correctly notes that Hollander-Cohen et al. did not detect *cckbr* in LH cells. We attribute this discrepancy to the resolution of cell population identity. As detailed in our response to Reviewer 2 (Comment 7), our scRNA-seq and new high-resolution FISH/IHC data reveal that *cckbrb* is expressed specifically in a subpopulation of dual-hormone gonadotropes (Fig. 3d; Supplementary Fig. S15). These cells were likely classified as "FSH cells" in previous studies due to high *fshb* expression, masking the receptor's presence in the LH-producing lineage. We have further validated the existence of these dual-hormone cells using protein-level co-localization (New Fig. 3a), consistent with recent findings by Tanaka et al. (2024).

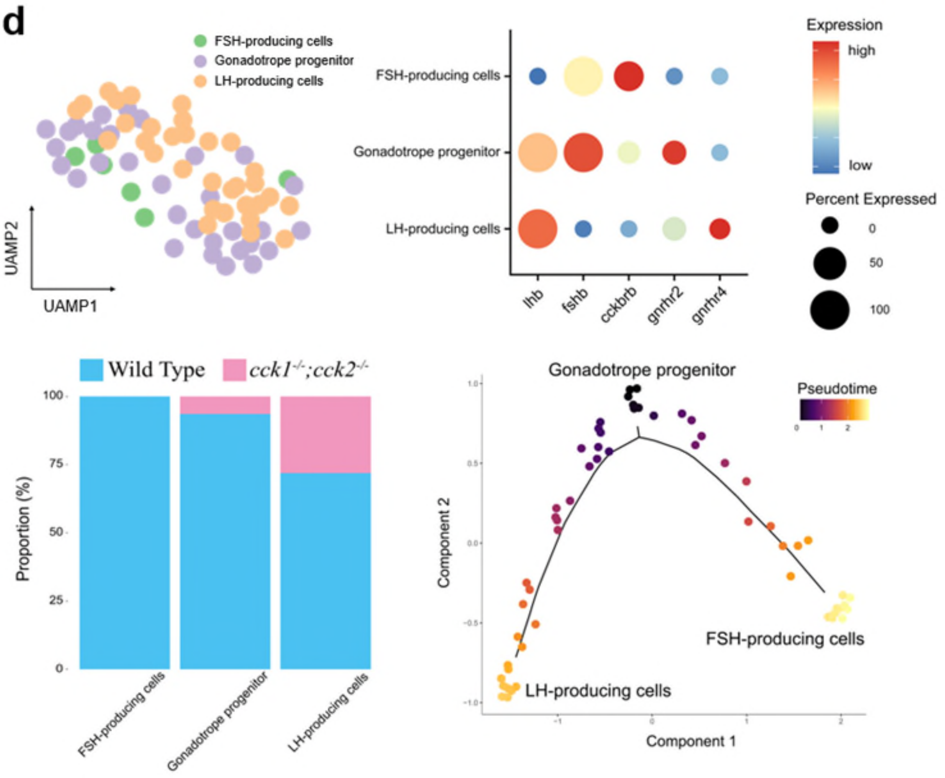

Fig. 3d Sub-clustering and trajectory analysis of the gonadotrope cell population.

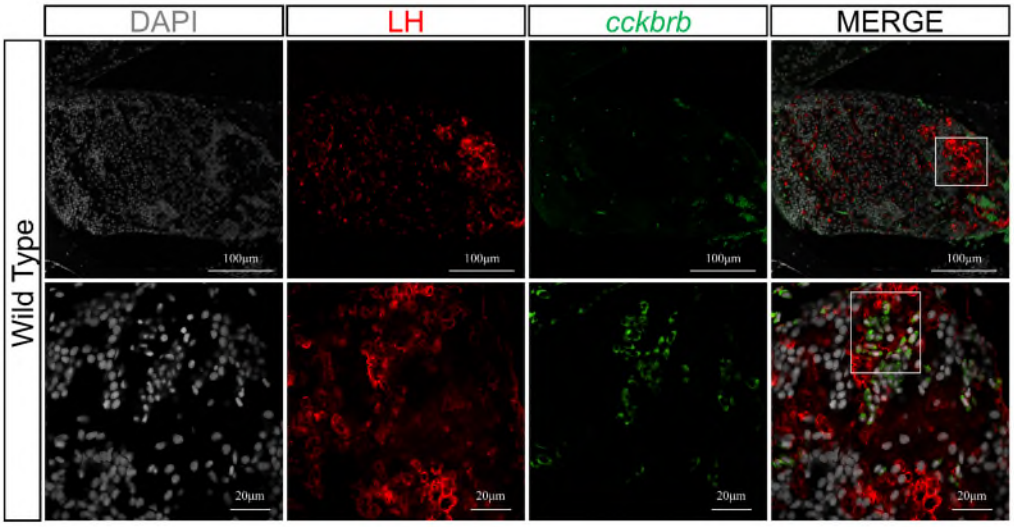

Supplementary Fig. S15 | Co-localization of *cckbrb* mRNA and LH protein in the adult male zebrafish pituitary gland. *cckbrb* mRNA was detected using a fluorescent probe (via Fluorescence *In Situ* Hybridization), and LH protein was visualized via immunohistochemistry (IHC).

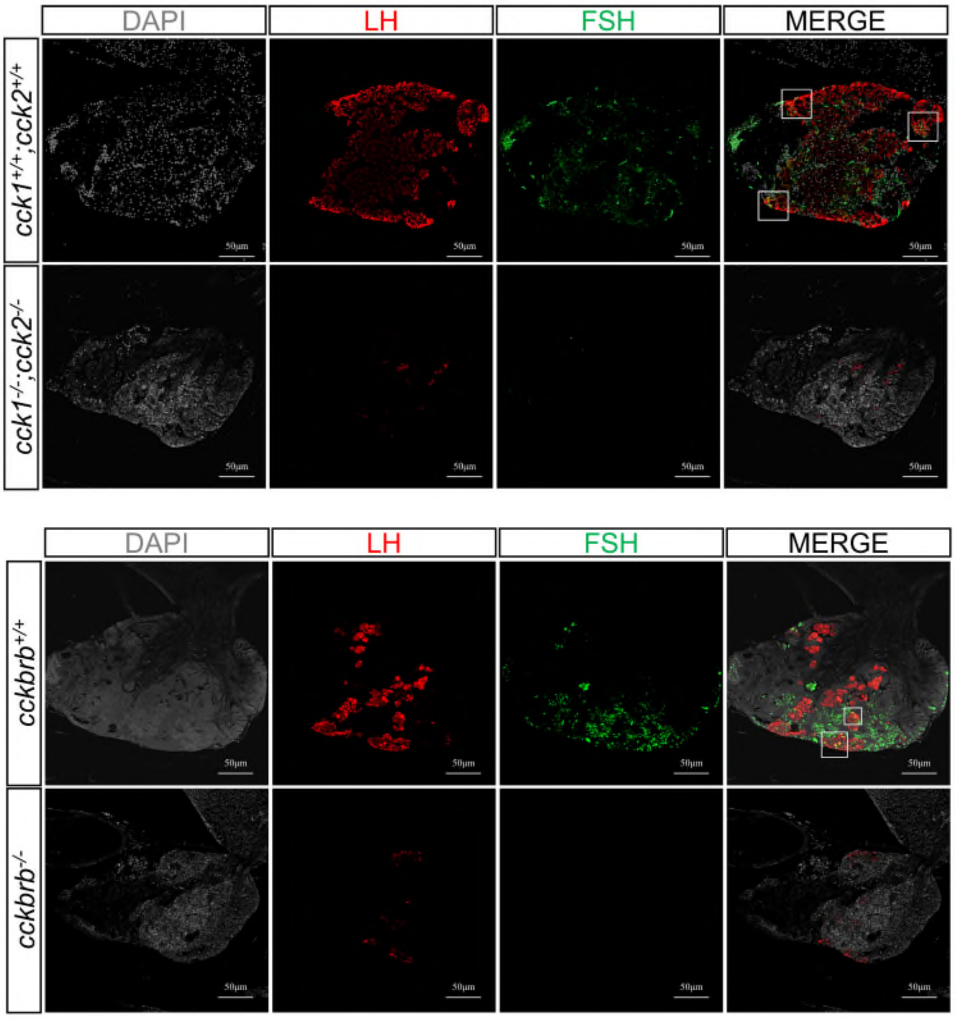

Fig. 3a Representative immunohistochemical staining of LH and FSH proteins in the pituitary of WT, *cck1*$^{-/-}$;*cck2*$^{-/-}$, and *cckbrb*$^{-/-}$ zebrafish.

(2). Direct regulation vs. Secondary estrogen effects (Gonadectomy Issue): We acknowledge the reviewer's valid concern that LH reduction could be secondary to low gonadal estrogen (as seen in medaka). While we did not perform *in vivo* gonadectomy, we addressed this logic using a functional equivalent: primary pituitary cell culture. In this *in vitro* system, pituitary cells are isolated from any gonadal influence (i.e., no systemic estrogen feedback). We found that CCK treatment robustly upregulates *lhb* expression in these cultured cells (Fig. 3i). This demonstrates that the pituitary is capable of responding to CCK directly in the absence of the gonad, ruling out the possibility that the regulation is *solely* driven by secondary ovarian estrogen failure.

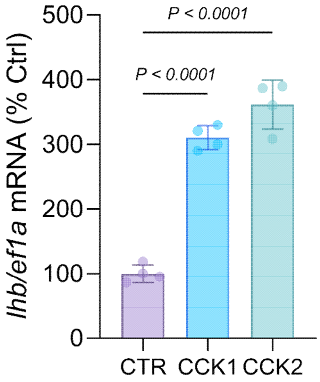

Fig. 3i. qPCR analysis of *lhb* gene expression in pituitary cells treated with CCK1 or CCK2 for 24 hours (*n*= 4)

(3). GnRH3 regulation and functional importance: Regarding the location and function of GnRH3 neurons:

Anatomy: We acknowledge the POA distribution. Our "hypothalamic" dissections for scRNA-seq and culture typically encompass the preoptic area-hypothalamic continuum.

Functional validation: To move beyond simple receptor expression, we performed functional assays. We show that CCK treatment significantly upregulates *gnrh3* in isolated hypothalamic/POA cells (Fig. 3h).

Necessity: Most importantly, to establish functional importance, we used a GnRH3-neutralizing antibody in a hypothalamic-pituitary co-culture system. Blocking GnRH3 signaling significantly attenuated (though did not abolish) the CCK-induced *lhb* response (Supplementary Fig. S14). This provides direct functional evidence that the GnRH3 pathway is a necessary component of CCK's action on LH, complementing the direct pituitary mechanism.

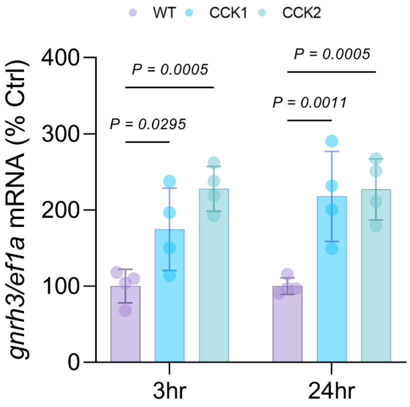

Fig. 3h *gnrh3* mRNA levels in hypothalamic cells treated with CCK1 or CCK2 for 3 hours and 24 hours (*n*= 4).

Editorial Note: Supplementary Fig. S14a in this Peer Review File is reproduced with permission from BioRender, Created in BioRender. Li, H. (2026) https://BioRender.com/hgl9icn

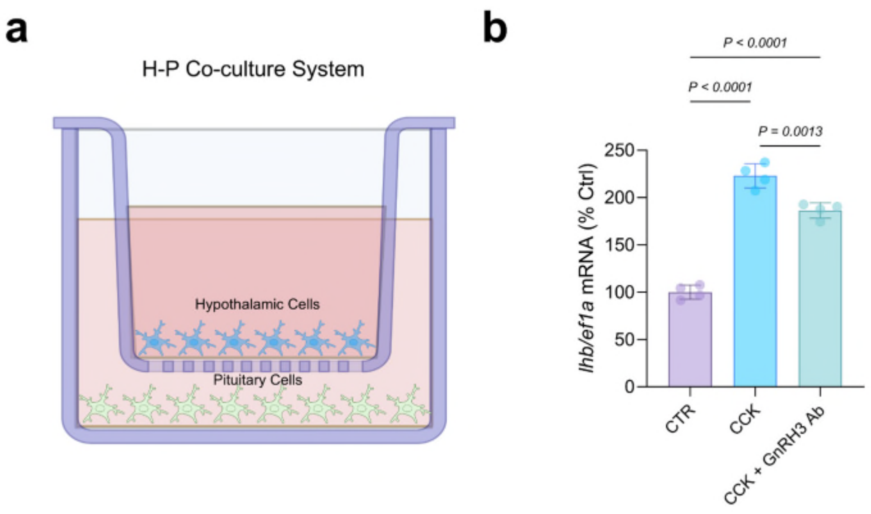

Supplementary Fig. S14 Hypothalamic-Pituitary (H-P) co-culture system and functional assessment of dual CCK regulation. a Schematic diagram of the H-P co-culture system. Hypothalamic cells are cultured on the transwell insert membrane, and pituitary cells are cultured in the lower chamber, facilitating the transfer of soluble factors. **b** qPCR analysis of *lhb* mRNA expression in pituitary cells from the co-culture system following treatment with Control (Ctrl), CCK only (CCK), and CCK with GnRH3 blocking antibody (CCK + GnRH3 Ab). Data are presented as mean ± s.e.m. (standard error of the mean) for *n*= 4 independent experiments.

(4). Interpretation of ovulation: We agree with the reviewer that injection outcomes can be multifactorial. As noted in our response to Reviewer 1, we have tempered our conclusion to state that the pro-ovulatory effect is "consistent with" CCK's regulation of LH.

“Functionally, this dual regulation is physiologically relevant, as injection of either CCK or LH significantly restored ovulation rates in female zebrafish (Fig. 3k).” (Line 202-203)

References

1. Farrell, J. A., Wang, Y., Riesenfeld, S. J. et al. Single-cell reconstruction of developmental trajectories during zebrafish embryogenesis. *Science*, **360**, eaar3131(2018).
2. Tanaka, S., Yu, Y., Levavi-Sivan, B., Zmora, N. & Zohar, Y. GnRH—gonadotropes interactions revealed by pituitary single-cell transcriptomics in zebrafish. *Endocrinology.* **165**, bqae151 (2024).

Responses to the Comments from the Reviewers

Cholecystokinin coordinates gonadotropin-dependent and independent pathways to orchestrate zebrafish gonadal development

Reviewer #1 (Remarks to the Author):

The authors have responded to all of my questions and concerns and have extensively revised the manuscript with addition of a substantial amount of new data. This reviewer appreciates the considerable effort involved. However, some questions and concerns remain and should be further addressed or clarified.

Response:

We sincerely thank the reviewer for their positive assessment of our revision efforts and for acknowledging the substantial amount of new data added to the manuscript. We have carefully considered the remaining questions and concerns raised below and have provided point-by-point responses to further clarify these issues and enhance the rigor of our study.

1. The authors tested the effects of FSH and GH on the expression of lhb for possible paracrine regulation in the pituitary. This is good. However, what FSH and GH were used in the experiment? Recombinant proteins from zebrafish or other species? If the hormones are heterologous, then the authors need to be cautious in interpreting the data as hormones from different species may not act as specifically as homologous ones. The manuscript also mentioned using recombinant zebrafish LH obtained from a commercial company (Beijing VJTBio). However, no information was given about how the recombinant hormone is produced and how the activity is validated. Also, no information about this hormone is available at the company's website. The authors should add this information in the manuscript.

Response to Comment 1:

We thank the reviewer for highlighting the importance of reagent transparency and species specificity. We explicitly confirm that all hormones used in this study (FSH, LH, and GH) were recombinant zebrafish proteins, rather than heterologous proteins, ensuring the physiological relevance of our findings. We have updated the "Materials and Methods" section with the specific production and validation details below.

Regarding the gonadotropins, the recombinant zebrafish LH and FSH proteins were custom-synthesized by Beijing VJTBio. To preserve biological activity dependent on post-translational modifications, these proteins were produced using a eukaryotic expression system rather than bacteria. Their purity was confirmed by SDS-PAGE, showing single bands at the expected molecular weights (Fig. R1). Furthermore, their biological activities were rigorously validated: recombinant LH effectively induced ovulation *in vivo* (Fig. 3k in the manuscript), while recombinant FSH significantly stimulated the expression of the steroidogenic enzyme *cyp19a1a* in the ovary. Specifically, intraperitoneal injection of rzfFSH (200 ng/g body weight; *n*= 6) significantly upregulated ovarian *cyp19a1a* mRNA levels at 24 hours post-injection (Fig. R2).

For the recombinant zebrafish GH, the protein was custom-synthesized by GenScript

and was also produced using a eukaryotic expression system to ensure proper protein folding. The identity and purity of the rzfGH were verified via SDS-PAGE (Fig. R1). Its biological activity was confirmed *in vivo*. Intraperitoneal injection of rzfGH (100 ng/g body weight; *n*= 6) resulted in a significant upregulation of insulin-like growth factor 1 (*igf1*) mRNA expression in the liver at 24 hours post-injection compared to saline-injected controls (Fig. R3), confirming its capacity to activate the hepatic GH/IGF signaling axis.

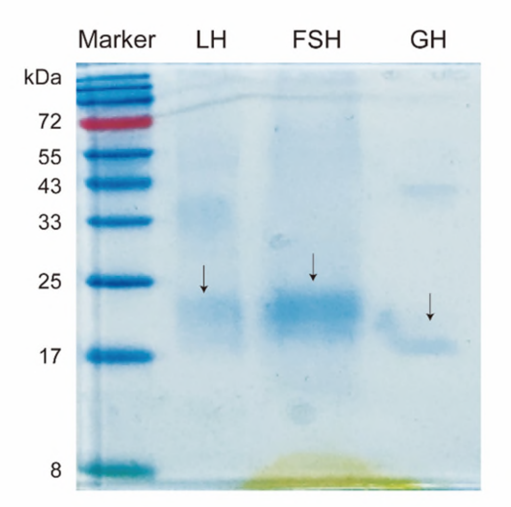

Fig. R1 | SDS-PAGE validation of recombinant zebrafish hormones. Purified recombinant zebrafish LH, FSH, and GH proteins were resolved by SDS-PAGE and stained with Coomassie Blue.

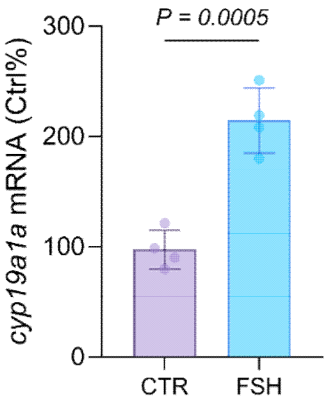

Fig. R2 | Biological activity validation of recombinant zebrafish FSH (rzfFSH). Adult female zebrafish were intraperitoneally injected with rzfFSH (200 ng/g body weight) or saline control (*n*= 4 fish per group). Ovarian tissues were collected 24 hours post-injection. The mRNA expression levels of the steroidogenic gene *cyp19a1a* were quantified by qPCR. Data are presented as mean ± s.e.m.

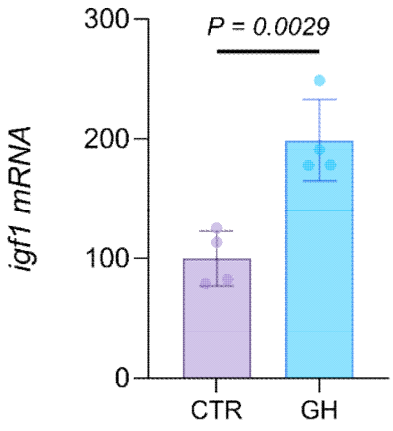

Fig. R3 | Biological activity validation of recombinant zebrafish GH (rzfGH). Adult zebrafish were intraperitoneally injected with rzfGH (100 ng/g body weight) or saline control (*n*= 4 fish per group). Liver tissues were collected 24 hours post-injection to assess the activation of the GH/IGF signaling axis. The mRNA expression levels of *igf1* were quantified by qPCR. Data are presented as mean ± s.e.m.

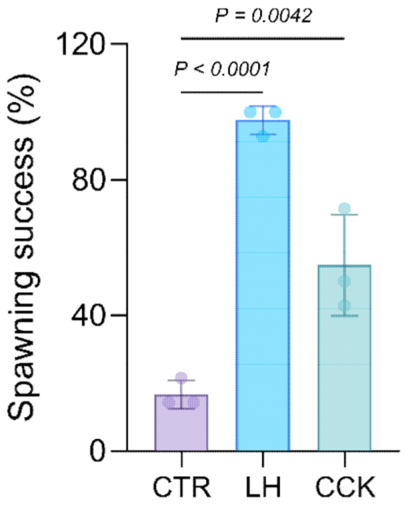

Fig. 3k Functional assessment of ovulation success rate (%) in female zebrafish 24 hours after i.p. injection of LH or CCK (*n*= 14 biologically independent animals from 3 independent experiments). Data are presented as mean ± s.e.m.

2. "Unlike mammals, teleosts lack a median eminence. Instead, hypothalamic neurons directly innervate the pituitary, enabling multiple neuropeptides to directly regulate gonadotropes." This statement is questionable as direct innervation is not the condition for multiple neuropeptides to directly regulate gonadotropes. The median eminence allows more neuropeptides or neurotransmitters to directly regulate pituitary cells.

Response to Comment 2:

We sincerely appreciate the reviewer for correcting our statement regarding the comparative neuroanatomy of the median eminence. We fully accept the critique that direct innervation is not a prerequisite for multi-peptide regulation, and we agree that the mammalian median eminence system effectively facilitates complex regulation by allowing numerous factors to reach the pituitary via the portal circulation.

Our previous phrasing conflated anatomical delivery methods with the capacity for multi-factorial control, which was an error in logic. Our intended argument was to highlight the functional redundancy inherent in the teleost system. The absence of a severe phenotype in GnRH-deficient models is likely due to the molecular diversity of the gonadotropes themselves—specifically, their expression of receptors for multiple neuroendocrine factors (including CCK, Neurokinin B, and Secretogranin-2).

Action Taken:

We have revised the Introduction (Lines 41–45) to remove the inaccurate causal link between direct innervation and multi-peptide regulation. The updated text now emphasizes that the anatomical arrangement in teleosts facilitates a multi-factorial control system without making flawed comparisons to the mammalian median eminence.

Revised Text (Lines 41–45):

"...Although luteinizing hormone-releasing hormone (LH-RH), discovered in 1971, is well-established as the main regulator of gonadotropin release in mammals and most vertebrates[15], this model has been challenged in teleosts. In teleosts, hypothalamic neurons directly innervate the pituitary pars distalis. This anatomical arrangement facilitates the direct delivery of diverse neuroendocrine factors to gonadotropes, which express a repertoire of receptors to integrate these signals. Consistent with this multi-factorial control system, in GnRH-deficient models like medaka and zebrafish, FSH synthesis and function remain largely unaffected..."

3. Figure S13 examined the effect of GnRH3 on lhb expression. Why not looking at fshb expression as well in the samples?

Response to Comment 3:

We thank the reviewer for this constructive suggestion. We agree that examining *fshb* expression provides a more comprehensive view of GnRH3 regulation. We have performed the qPCR analysis on the same primary pituitary cell samples. The results showed that GnRH3 treatment significantly upregulated *fshb* mRNA expression. However, notably, the magnitude of this induction was much less pronounced compared to *lhb*. This differential responsiveness supports the view that while GnRH3 can modulate FSH (likely via the bi-hormonal subpopulation), it functions primarily as the dominant regulator for LH. We have included this new dataset in the revised manuscript as Supplementary Fig. S13b (the original LH data is now panel a).

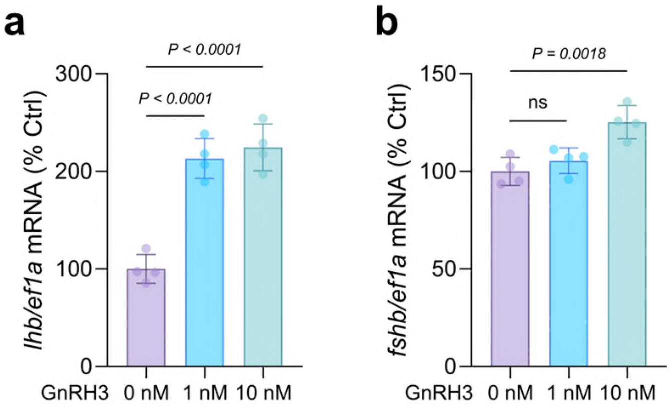

Supplementary Fig. S13 | Effects of GnRH3 on gonadotropin expression in pituitary cells *in vitro*. a qPCR analysis of relative *lhb* mRNA expression in primary cultured zebrafish pituitary cells treated with graded concentrations of GnRH3 (0, 1, and 10 nM) for 24 hours. **b** qPCR analysis of relative *fshb* mRNA expression under the same experimental conditions. Data are presented as mean ± s.d. for *n*= 4 independent biological replicates. Expression levels were normalized to *ef1a*.

4. Regarding the use of a gnrh3 knockout to demonstrate the indirect CCK–GnRH3–LH pathway, the authors tried to argue against this approach by invoking possible developmental compensation. This is not a convincing argument. While developmental compensation can occur in stable mutant lines, genetic loss-of-function is still the most appropriate and direct way to test this pathway. By the same logic, the authors would not have performed the experiment using fshb-/- mutant to demonstrate FSH involvement in CCK action. Therefore, developmental compensation alone is insufficient justification to dismiss the gnrh3 knockout approach. It is understandable that gnrh3 mutant line was not available and generating it would take a long time, longer than the time allowed for the revision. This explanation would be good and reasonable enough.

Response to Comment 4:

We sincerely appreciate the reviewer's candid and constructive feedback. We fully accept the reviewer's point that our previous argument regarding developmental compensation was inconsistent, particularly given our successful use of the *fshb* mutant. We agree that genetic loss-of-function is the gold standard for verifying such pathways, and "developmental compensation" alone should not preclude the use of *gnrh3* mutants.

As the reviewer kindly acknowledged, the primary constraint preventing us from performing this experiment is the unavailability of a *gnrh3* mutant line in our laboratory. Generating a stable homozygous mutant line in zebrafish typically requires at least two to three generations (approximately 6–8 months), which extends significantly beyond the time frame allowed for this revision. We are grateful for the reviewer's understanding of this practical limitation.

To address the mechanism of the CCK–GnRH3–LH pathway in the absence of a mutant, we utilized an immunological neutralization strategy using a specific affinity-purified anti-GnRH3 antibody. In our hypothalamic-pituitary co-culture system, the addition of this neutralizing antibody effectively abolished the CCK-induced upregulation of *lhb* expression (Fig. S14). We believe this functional data provides robust evidence for the proposed pathway within the constraints of the current study.

5. The authors performed immunohistochemical staining for LH and FSH. However, the antibodies used were raised by the same group before against grass carp hormones. The specificity of these antibodies in zebrafish is questionable and needs to be validated.

Response to Comment 5:

We verify that the antibodies used were rabbit polyclonal anti-grass carp LH and rat polyclonal anti-grass carp FSH, custom-synthesized by Dia-An (Wuhan). Although these antibodies were raised against grass carp proteins, the amino acid sequences are highly conserved between grass carp and zebrafish. To empirically validate their specificity for zebrafish targets, we performed two complementary experiments.

First, Western blot analysis (Fig. R4) using the purified recombinant zebrafish proteins (described in Comment 1) confirmed that the antibodies specifically recognize zebrafish LH and FSH, detecting single distinct bands at the expected molecular weights without non-specific background. Second, to verify specificity in situ, we analyzed the double immunofluorescence data presented in Fig. 3a of the manuscript. Consistent with recent studies in teleosts showing both distinct and overlapping gonadotroph populations, our staining confirmed the presence of distinct LH- and FSH-expressing cells alongside a subset of bi-hormonal cells. Crucially, the observation of clear single-positive populations for each hormone confirms that the antibodies do not cross-react; otherwise, ubiquitous colocalization would be observed.

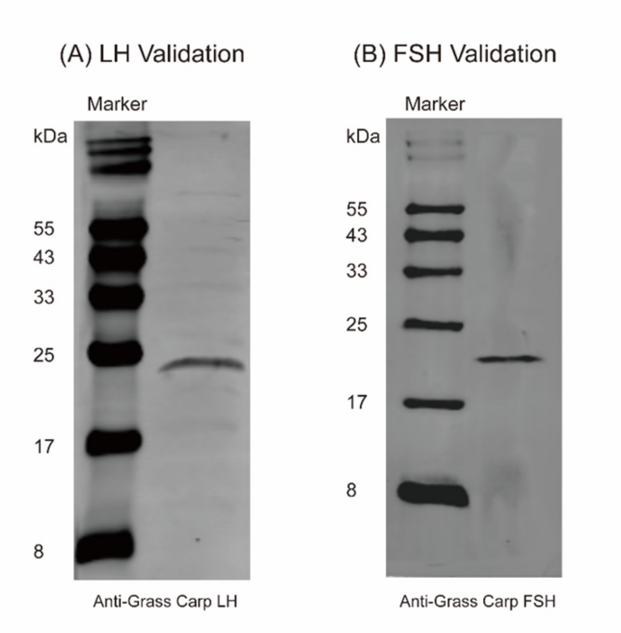

Fig. R4 | Western blot analysis of recombinant zebrafish LH and FSH proteins. (A) Immunoblotting of recombinant zebrafish LH protein probed with the rabbit anti-grass carp LH antibody. (B) Immunoblotting of recombinant zebrafish FSH protein probed with the rat anti-grass carp FSH antibody.

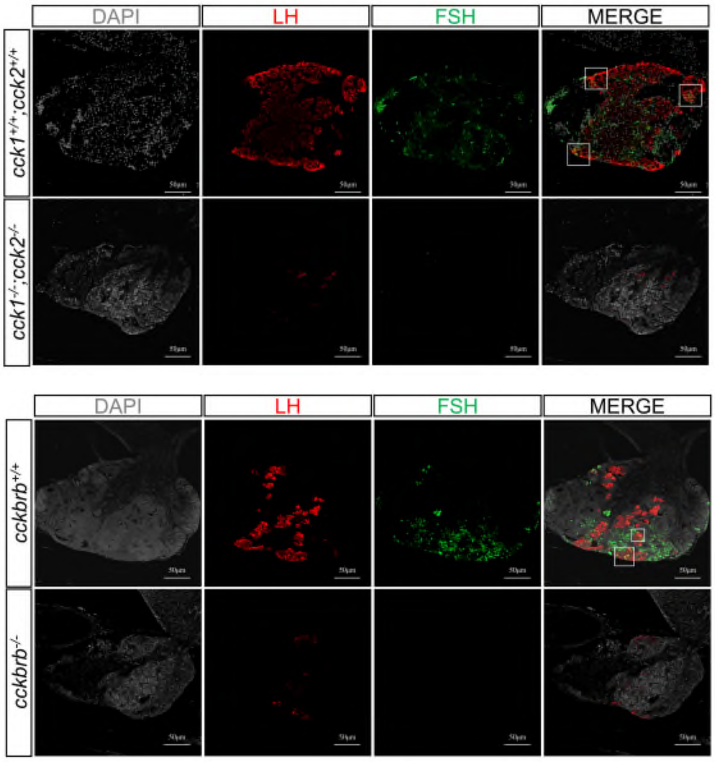

Fig. 3a Representative immunohistochemical staining of LH and FSH proteins in the pituitary of WT, *cck1*$^{-/-}$;*cck2*$^{-/-}$, and *cckbrb*$^{-/-}$ zebrafish.

Reviewer #2 (Remarks to the Author):

The authors have responded to all of my questions and suggestions and have supplemented the manuscript with a substantial amount of new experimental data. Overall, the revised version represents a marked improvement over the original submission. This manuscript provides a comprehensive and technically impressive analysis of the role of CCK signaling in zebrafish gonadal development. By integrating multiple genetic models and functional assays, the study substantially advances our understanding of CCK as a regulator of both gonadotropin-dependent and -independent processes during early germline development. The overall framework and conclusions are of clear interest for clarifying the physiological roles of CCK in teleost reproduction.

1. However, several questions and concerns still remain and need to be further addressed or clarified.I do not question the reported reduction in PGC number and the all-male phenotype in CCK mutants. However, PGC number and migration defects occur at early embryonic stages, whereas the somatic cell apoptosis proposed as a mechanism is observed mainly after ~14 dpf, making a direct causal link unclear. How CCK regulates PGC number during early development therefore remains unresolved.

2. Related to the above point, the authors show that tp53 knockout rescues the meiotic defects caused by loss of CCK (this genetic rescue is very elegant), and the mechanism is attributed to suppression of somatic cell apoptosis. However, the manuscript does not report the sex composition of the cck;tp53 double mutants. The data presented indicate restoration of spermatogenesis, but female development does not appear to be recovered. If the all-male phenotype in CCK mutants is primarily driven by somatic apoptosis, why is female fate not restored when apoptosis is rescued? This suggests that meiotic rescue and sexual fate determination may be separable processes, and further implies that CCK's effects on early PGC behavior cannot be fully explained by somatic apoptosis alone.

Response to Reviewer #2 (including Comments 1 & 2):

We sincerely thank the reviewer for their positive assessment and for raising this critical point regarding the causal link between early PGC defects and late somatic apoptosis. We fully accept the reviewer's insightful critique. We agree that while we initially conflated these events, the data from our *cckbrb;tp53* double mutants actually reveal a fascinating biological distinction between sex determination and fertility.

As the reviewer correctly pointed out, *tp53* deficiency successfully rescued somatic cell survival and restored spermatogenesis (fertility), yet it failed to restore the female sex ratio (the rescued cohort remained all-male). This result is pivotal. It supports a "Two-Phase Model" of CCK regulation:

Phase I (Embryonic): The reduction in PGC numbers occurs early (0-10 dpf) and is tp53-independent. This initial germ cell depletion likely drops below the threshold required for female differentiation, thereby irreversibly locking the gonad into a male trajectory.

Phase II (Larval/Juvenile): The subsequent somatic cell apoptosis occurs later (14-45 dpf) and is *tp53*-dependent. This apoptosis is what drives the massive germ cell loss and ultimate sterility.

Therefore, by rescuing the apoptosis (Phase II) without fixing the PGC deficit (Phase I), we generated "Fertile Males." This elegantly demonstrates that CCK's role in early PGC proliferation (sex determination) is mechanistically distinct from its role in maintaining somatic integrity (fertility).

Action Taken:

We have revised the Discussion section (Lines 390–400) to explicitly incorporate this "Two-Phase" model. The updated text clarifies that while somatic apoptosis drives the sterility/meiotic arrest, it is the upstream, gonadotropin-independent PGC deficiency that likely dictates the male-biased sex determination.

Revised Text (Lines 390–400):

"...Concurrently, gonadal somatic cells (GSCs), which support meiosis through nutrients provision and regulatory signals, underwent extensive apoptosis at 14 and 45 dpf. Given that GSC depletion disrupts meiosis and gametogenesis, this phenotype provides a mechanistic basis for the observed arrest. This causality is further substantiated by our genetic rescue data: *cckbrb*$^{-/-}$;*tp53*$^{-/-}$ double mutants exhibited suppressed apoptosis and restored spermatogenesis (fertility). However, remarkably, female individuals were not observed in the rescued cohort. This dissociation supports a "two-phase" model: the initial reduction in PGC numbers (Phase I) likely locks the gonad into a male trajectory—a defect that *tp53* deletion cannot reverse—whereas the subsequent somatic apoptosis (Phase II) drives the meiotic arrest and sterility. Thus, while *tp53* deficiency rescues gonadal integrity and fertility, it is insufficient to overturn the sex determination bias established by early PGC loss. Crucially, neither somatic apoptosis nor meiotic arrest occurred in *fshb*$^{-/-}$ or *fshb*$^{-/-}$;*lhb*$^{-/-}$ mutants..."

Reviewer #3 (Remarks to the Author):

The reviewer would like to acknowledge the considerable effort made by the authors in adding a large number of experiments within a relatively short revision period. These new data are valuable in that they move the study away from the original, incorrect hypothesis that hypothalamic CCK simultaneously regulates both the pituitary and the gonads, and instead support an independent regulation model, which is likely a more plausible conclusion. This shift demonstrates that the additional experiments have generated important information.

On the other hand, this shift completely changed the concept, and the title "Cholecystokinin integrates gonadotropin-dependent and -independent pathways to orchestrate zebrafish gonadal development" cannot be applied.

Also, even with many additional experiments, the evidence remains insufficient to overturn previously published studies showing little or no direct effect of CCK on LH regulation, and the newly proposed model of local regulation of CCK in the gonads is still not fully supported. In addition, for several of the newly added experiments, validation of the experimental systems and key antibody appears insufficient, leaving uncertainties in data interpretation. Please refer to the specific comments below.

To "Response to Comment 1:"

>We acknowledge the initial concerns regarding the mechanistic depth of our study and the reliance on correlative data from whole-body knockouts and scRNA-seq. In direct response, we have undertaken an extensive series of new experiments designed to transform key correlative observations into causally supported conclusions. The revisions below address each major concern with decisive new evidence.

>(1). Upgrading methodological rigor and externally validating key cell populations

The authors state that cckbrb is expressed in LH cells with the additional data of double labeling of cckbrb mRNA and LH protein. However, when viewed at low magnification, this co-localization is very minor (Supplementary Fig. S15). Even if present, the degree of co-localization is extremely low, which is in clear contrast to the situation in FSH cells, where cckbrb expression is almost complete, as reported previously (Hollander-Cohen et al., 2024; Uehara et al., 2024). Also, if CCK indeed exerts a direct effect on LH, responses comparable to those observed for FSH should have been detected in LH in Hollander-Cohen et al. (2024) (see absence of effect in Hollander-Cohen et al., 2024, Figure4). The present study does not provide sufficient data to overturn the clear difference in responsiveness between FSH and LH reported in prior studies.

Therefore, based on previous studies and the data presented here, the current model implying that CCK acts on FSH and LH in a similar manner is not objectively supported..

>(2). Providing mechanistic proof for LH regulation

In your data, only a very small subset of LH cells is reported to express cckbrb. Given this, how can the authors explain a nearly threefold increase in overall LH expression? It is difficult to reconcile these two observations.

Importantly, cckbrb is a receptor that can signal through both Gs and Gq pathways. Therefore, if CCK truly increases LH expression under physiological conditions via a Gs-mediated mechanism, it should also activate Gq signaling and consequently elevate intracellular Ca^{2+} levels. However, as noted above, in freshly prepared pituitary tissues, LH cells show little to no response to CCK peptide (Hollander-Cohen et al., 2024). In other words, the results reported by Hollander-Cohen et al. indicate that, under physiological conditions, CCK does not directly act on LH cells, and likewise do not support a direct increase in LH expression under physiological conditions.

This interpretation is also consistent with the low degree of cckbrb co-localization in LH cells observed in the present study.

As mentioned in my previous comments, considering the findings of Romain et al. (2020), it is well established that culturing pituitary cells alters FSH and LH expression patterns, often leading to increased co-expression of FSH and LH. Thus, the authors may be observing the effects of CCK under these non-physiological, culture-specific conditions.

Unless the authors adequately explain why Hollander-Cohen et al. failed to observe Calcium responses in LH cells upon CCK application, this hypothesis cannot revise the current understanding.

>Functional validation of the GnRH3 pathway:

I acknowledge to establish this new experiment (Supplementary Fig.S14) . However, the experimental design relies on the use of antibodies, and the appropriate control in this case should be normal serum. Without such a control, it is not possible to exclude the possibility that other components present in the serum contributed to the observed reduction in LH levels. Therefore, the experimental system itself requires reconsideration.

In addition, adding immunized rabbit serum to the culture medium at a dilution of 1:500 is too much and may introduce unintended side effects, and these potential confounding effects should be carefully considered and controlled for.

More broadly, for all experiments added during the revision that rely on antibodies, proper validation of the antibodies used is essential. For example, it should be demonstrated whether the effect of GnRH can be abolished by this antibody. Without such validation, the specificity and physiological relevance of the observed effects remain uncertain.

>Precise source of CCK for early gonadal action: In response to the valid criticism regarding the schematic, spatial expression analysis revealed that during early development, the CCK influencing the gonad originates from local tissue, not the hypothalamus (Fig. 4d,e; Fig. R5). We have therefore revised our working model (Fig. 5i) to accurately distinguish between CCK's neuroendocrine role (hypothalamus-pituitary) and its local paracrine role (local source-gonad).

This is a very reasonable set of data. If feasible, demonstrating whether this local source is truly important through a tissue- or cell-type–specific knockout would be necessary to reach a solid conclusion..

>This lack of robust expression in the PGC lineage supports the hypothesis that CCK regulation of PGCs is likely indirect.

Which cell types mediate this effect? At a minimum, tissue-level in situ hybridization is required to address this point.

Overall, in the original version, a working hypothesis was proposed without sufficient evidence, namely that hypothalamic CCK is required for PGC proliferation, and this hypothesis was subsequently reversed in the revision. I believe that interpreting what is not directly observed and elevating such interpretations into hypotheses is potentially dangerous. In this regard, it is appropriate that the authors did not retain the original conclusion in the revised version. In this sense, the all conclusions, including the title and abstract should be carefully reviewed.

To “Response to Comment 3:”

>(1). Discrepancy with Hollander-Cohen et al. (2024):

As stated above, the fact that little to no response is observed in LH:GCaMP recordings is a serious concern. Even if a small response were detected in a very limited subset of LH cells, it would not be reasonable to discuss this effect as operating in parallel with the robust action of CCK on FSH, nor to draw the two as equivalent pathways in a schematic model. Such a representation does not reflect the relative strength or reliability of the underlying data.

[Concerns about the newly added data]

(1) In Fig. S9, it is unclear how the peptide is taken up when delivered through food because peptides are digested in the stomach or gut. Moreover, the reported

increase in mRNA expression within 1.5 hours appears unusual and requires further explanation. Given the small sample size (n = 3), it is questionable whether the observed difference is sufficiently robust and clear to support a firm conclusion.

(2) In addition, it is not specified which CCK peptide sequence was used in these experiments. The peptide sequence is not described anywhere in the manuscript and should be explicitly provided.

(3) Information regarding the antibodies and related reagents is insufficient, and their validation is inadequate. For both the neutralizing antibody and the antibodies used for staining, a dilution of 1:500 appears excessively concentrated. Moreover, beyond the statement that the antibodies were "custom-synthesized by Dia-An, Wuhan," no additional information is provided. As a result, the specificity of these antibodies remains unclear.

(4) In Fig. S11, the authors refer to the staining as GnRH immunohistochemistry, but the anatomical location of the signal is unclear. Moreover, GnRH3 neurons are not expected to be present in such large numbers, raising the possibility that the signal represents something else. This raises concerns about whether a specific antibody was used at an appropriate concentration.

Please refer to Spicer et al. (2016) for the expected distribution and characteristics of GnRH3 neurons. https://doi.org/10.1371/journal.pone.0158141.

(5) In lines 587–589, the manuscript states:

"The anti-GnRH3, anti-LH, and anti-FSH antibodies were custom synthesized by Dia-An (Wuhan) following the method of Feng et al. (24) and validated in previous studies (10)."

However, reference 24 describes the generation of a GnRH3 antibody, whereas reference 10 does not provide validation of these antibodies, nor does it clearly describe their origin or specificity. Therefore, the claim that these antibodies were "validated in previous studies" is not supported by the cited references. Moreover, the wording suggests that the antibodies were newly generated for the present study, which is inconsistent with the statement that they have already been validated. As a result, both the provenance and specificity of these antibodies remain unclear.

Response to Reviewer #3:

We wish to express our sincere gratitude to the reviewer for their rigorous and insightful assessment of our manuscript. We deeply appreciate the reviewer's recognition of the "considerable effort" we invested in the revision and the value of the new experimental data.

We fully accept the reviewer's critical comment that the additional data have fundamentally shifted our understanding from the original hypothesis to a more accurate "independent regulation" model. We agree that the neuroendocrine (pituitary) and paracrine (gonadal) actions of CCK operate via distinct pathways.

Below, we provide a point-by-point response to every specific comment raised.

1. Response to "Title and Concept Shift"

Comment:

The reviewer noted that the title "integrates" is no longer applicable given the shift to an independent regulation model.

Response:

We fully agree. To accurately reflect this distinction while highlighting that these pathways work in concert to achieve gonadal development, we have modified the title to:

"Cholecystokinin coordinates gonadotropin-dependent and -independent pathways to orchestrate zebrafish gonadal development"

We believe "coordinates" better captures the physiological reality: while the pathways are mechanistically distinct and spatially separated, CCK signaling acts as a synchronizing factor to ensure both germ cell establishment and hormonal support.

2. Response to "Mechanistic Proof for LH Regulation"

Response to LH Regulation & Hollander-Cohen Discrepancy:

Comment:

The reviewer questioned the physiological relevance of CCK on LH, citing low cckbrb co-localization and the lack of robust Ca^{2+} response in Hollander-Cohen et al. (2024). They also suggested that our in vitro results might be culture artifacts (altered expression patterns)

Response:

Regarding Hollander-Cohen et al. (2024): We respectfully note that Hollander-Cohen et al. actually reported that CCK *does* elicit a calcium response in LH cells, albeit "weaker" and "varied widely" (20%-100% of cells responding). This perfectly aligns with the well-established existence of a bi-hormonal subpopulation (cells co-expressing FSH and LH) in teleosts. We propose that the responses observed in our study are driven by CCK acting on these *cckbrb*-positive bi-hormonal cells.

Regarding Culture Artifacts: We acknowledge the reviewer's concern that primary culture conditions can influence gene expression patterns. However, we respectfully submit that our conclusion is not based solely on *in vitro* data. The most critical evidence comes from our *in vivo* genetic models: *cckbrb*$^{-/-}$ mutants exhibit a marked reduction in pituitary *lhb* mRNA expression in the intact animal. This *in vivo* phenotype definitively proves that CCK signaling is physiologically required for maintaining basal LH synthesis. If the CCK-LH regulation were merely a culture artifact, the loss of the CCK receptor *in vivo* would not result in such a profound suppression of *lhb* levels. Our *in vitro* data merely serves to dissect the mechanism (direct vs. indirect) of this established *in vivo* phenomenon.

Response to Mechanistic Proof for LH Regulation (Explaining the 3-fold Increase *in vitro*):

Comment:

The reviewer rightly questioned how a "very small subset" of cckbrb-expressing LH cells could sustain a "nearly threefold increase" in total LH expression in primary pituitary culture.

Response:

We appreciate the reviewer’s keen observation regarding the quantitative discrepancy between the sparse receptor distribution and the robust transcriptional response. We propose that the observed 3-fold increase in our *in vitro* system is driven by two synergistic factors, one of which aligns with the reviewer's own insight:

High Transcriptional Amplitude in Target Cells: While the *cckbrb*-positive subpopulation (bi-hormonal cells) is numerically small, the activation of the Gq-calcium signaling pathway in these cells is likely highly potent. A massive transcriptional induction (e.g., >20-fold) within a small specific subset of cells can mathematically result in a significant (3-fold) increase in the total mRNA pool of the entire culture.

Culture-Induced Cellular Plasticity (As noted by the reviewer): Importantly, we agree with the reviewer’s suggestion that "culturing pituitary cells alters FSH and LH expression patterns, often leading to increased co-expression." It is highly probable that in our primary culture system, the population of bi-hormonal (FSH+LH / CCK-responsive) cells expands or becomes more sensitive compared to the *in vivo* state. This culture-specific enrichment of the responsive population provides a logical explanation for why the direct CCK-induced *lhb* upregulation is more pronounced *in vitro* (3-fold) than might be predicted solely from *in situ* staining of fixed tissue.

Conclusion: Therefore, the *in vitro* data accurately reflects the intrinsic capability of CCK to drive *lhb* expression directly in responsive gonadotropes. However, we fully agree that *in vivo*, this direct pathway operates in concert with the powerful indirect GnRH3 pathway to achieve full physiological regulation.

3. Response to Antibody Specificity & Validation (Crucial Revisions)

Comment:

The reviewer raised concerns about antibody validation, specifically: (1) lack of normal serum control for functional assays; (2) 1:500 dilution being "too much"; (3) inaccurate citations (Ref 10 & 24) regarding validation; (4) GnRH3 signal abundance in POA.

Response:

We take these concerns regarding experimental rigor very seriously. We have addressed them as follows:

Antibody Validation (Western Blot & Pre-absorption): We explicitly verify that the antibodies used were Rat polyclonal anti-GnRH3, Rabbit polyclonal anti-LH, and Rat polyclonal anti-FSH. We validated them via:

Western Blot (Fig. R4): Using purified recombinant zebrafish proteins, confirming specific bands at expected molecular weights.

Antigen Pre-absorption (Fig. R5): For GnRH3, pre-incubating the antibody with synthetic peptide completely abolished the IHC signals, definitively proving that the staining is specific to the antigen.

Control for Functional Assays (Response to "Serum Control"): The reviewer correctly pointed out that normal serum is the appropriate control for whole serum antibodies. However, we clarify that all custom antibodies used in functional blocking experiments were affinity-purified, not whole serum. Therefore, the vehicle (PBS) is the appropriate control, as non-specific serum proteins are absent. This eliminates the confounding effects of serum components.

Response to "1:500 Dilution is too high": We apologize for the confusion. The 1:500 dilution refers to the affinity-purified antibody stock, not raw serum. For affinity-purified antibodies, a working dilution of 1:500 is a standard and appropriate concentration for IHC and neutralization assays, ensuring specific binding without the background associated with high-concentration raw serum.

Correction of Citations (Ref 10 & 24): We sincerely apologize for the citation error pointed out by the reviewer. We acknowledge that the previous references did not provide the specific validation for these custom antibodies. We have removed the inaccurate citations and instead included the direct validation data (Western Blot and Pre-absorption tests) in the revised manuscript and Supplementary Information to rigorously demonstrate specificity.

Response to GnRH3 Anatomical Distribution: Regarding the signal density in Fig. S11 compared to Spicer et al. (2016): Our imaging focused on the projection fibers in the hypothalamus. The apparent "large number" reflects the antibody's high sensitivity in detecting the dense fiber network. Most importantly, the pre-absorption test (Fig. R5) confirms that these signals are specific GnRH3 antigens, ruling out non-specific background.

4. Response to Peptide Experiments (Uptake & Sequence)

Comment:

The reviewer questioned the uptake mechanism (digestion, 1.5h timing) and requested peptide sequences.

Response:

Peptide Details: Per the reviewer's request, we have updated the Methods section (Lines 556–558) to explicitly provide the sequences of the synthesized zebrafish non-sulfated octapeptides used in this study. Importantly, both peptides were amidated at the C-terminus to ensure biological stability and activity:

CCK1 peptide: DYLGWMDF-NH_2

CCK2 peptide: DYVGWMDF-NH_2

Mechanism of Uptake (Agastric Physiology): regarding the concern about digestion, we emphasize that zebrafish are agastric (lacking a functional stomach) and its associated acidic environment. This unique physiological feature minimizes the proteolytic degradation that typically occurs in mammals. Furthermore, the CCK analog used is a short octapeptide (8 amino acids), which facilitates rapid transepithelial passage.

Timing (1.5h) & Direct Evidence: To address the concern about the 1.5-hour timeframe, we provided direct evidence via ELISA (Fig. S9b). The data shows a significant elevation in plasma CCK levels at 1.5 hours post-feeding, confirming that the peptide successfully entered the systemic circulation within this window. This timeframe is consistent with the rapid kinetics of absorption followed by the induction of immediate-early gene transcription in the pituitary.

Robustness of Sample Size (*n*= 3): Regarding the sample size, we clarify that the "*n*= 3" refers to three independent biological replicates, not technical replicates. Crucially, to ensure robustness and reduce individual variance, each biological replicate was a pooled sample derived from multiple animals (4 pituitaries per pool for qPCR; blood from 3 fish per pool for ELISA). This pooling strategy is a standard practice in zebrafish endocrinology to average out individual heterogeneity, ensuring that the observed statistically significant difference reflects a genuine biological effect rather than noise.

5. Response to "Local Source" & PGC Regulation

Comment:

The reviewer accepted the "local source" data but asked which cell type mediates the PGC effect (suggesting it is likely indirect).

Response:

We appreciate the reviewer's positive comment on our revised spatial expression data. Regarding the PGC regulation:

Indirect Mechanism: We agree with the reviewer that the regulation of PGCs is likely indirect/paracrine, given the lack of robust *cckbrb* expression directly on PGCs.

Local Source Identification: Our ISH data (Fig. 4d,e) identifies the surrounding somatic cells in the gonadal primordium as the source of CCK. We propose that local CCK acts on these somatic cells (which express *cckbrb*) to secrete downstream factors that support PGC proliferation. This "somatic-to-germline" paracrine support is a classic mechanism in gonadal development. While a tissue-specific knockout would be the definitive proof, the Editor has kindly exempted this long-term experiment from the current revision. We believe the combination of spatial mapping and phenotypic rescue data strongly supports this local paracrine model.

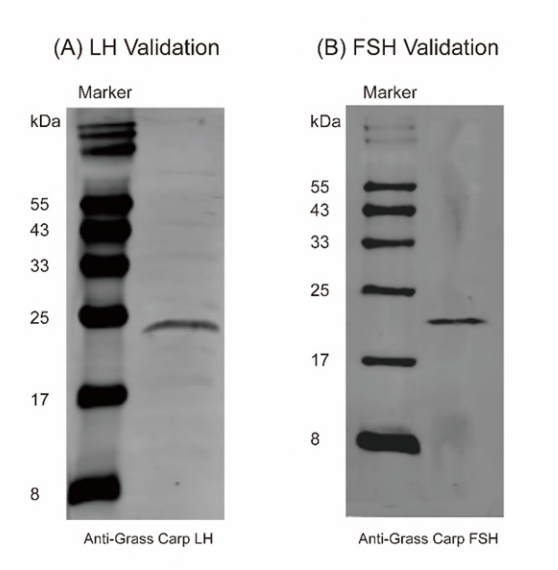

Fig. R4 | Western blot analysis of recombinant zebrafish LH and FSH proteins. (A) Immunoblotting of recombinant zebrafish LH protein probed with the rabbit anti-grass carp LH antibody. (B) Immunoblotting of recombinant zebrafish FSH protein probed with the rat anti-grass carp FSH antibody.

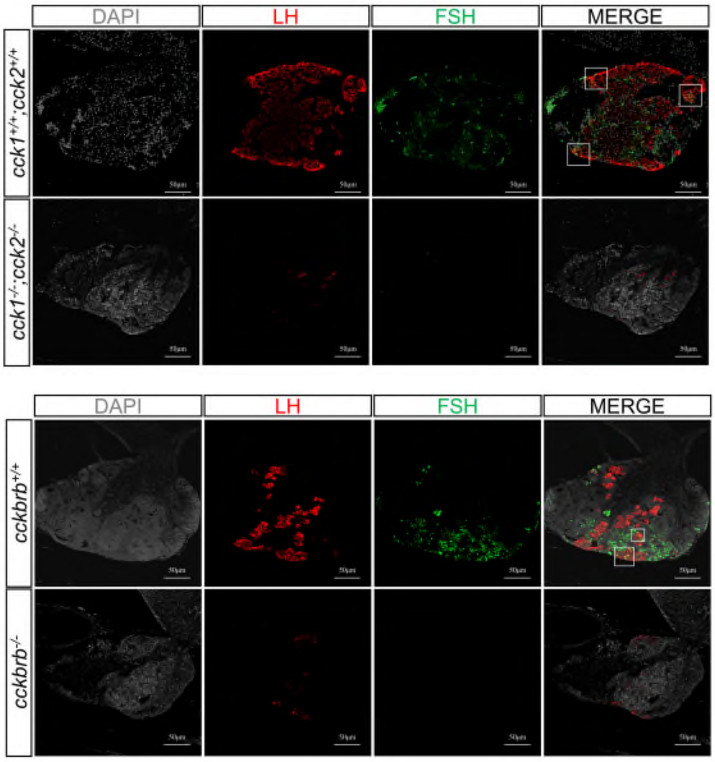

Fig. 3a Representative immunohistochemical staining of LH and FSH proteins in the pituitary of WT, *cck1*$^{-/-}$;*cck2*$^{-/-}$, and *cckbrb*$^{-/-}$ zebrafish.

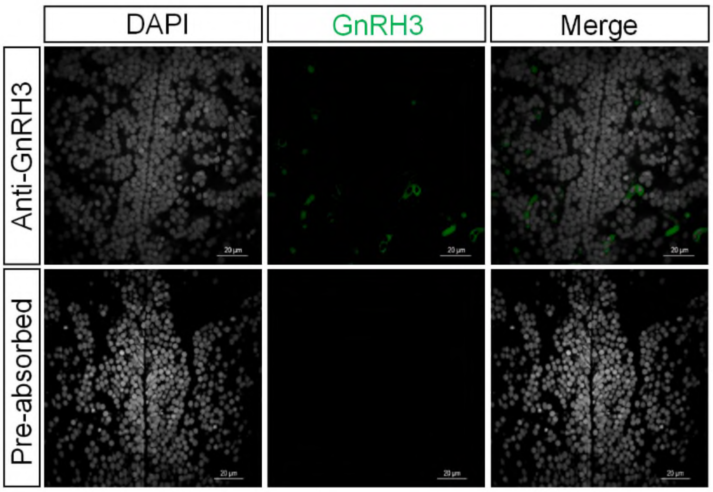

Fig. R5 | Validation of GnRH3 antibody specificity via antigen pre-absorption. Immunohistochemical staining of the zebrafish hypothalamus using anti-GnRH3 antibody compared with staining using the antibody pre-absorbed with synthetic GnRH3 peptide.

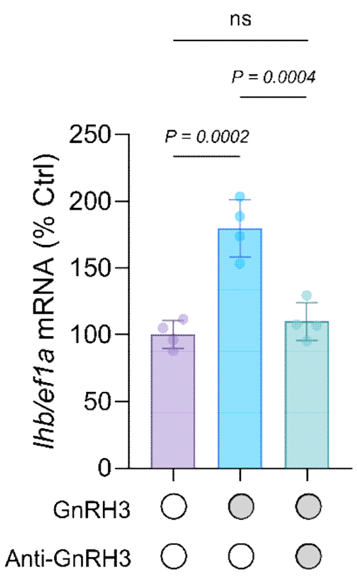

Fig. R6 | Functional neutralization of GnRH3 bioactivity by anti-GnRH3 antibody *in vitro*. Relative mRNA expression of *lhb* in primary zebrafish pituitary cells treated with synthetic GnRH3 peptide alone or pre-incubated with anti-GnRH3 antibody. Data are presented as mean ± s.d.

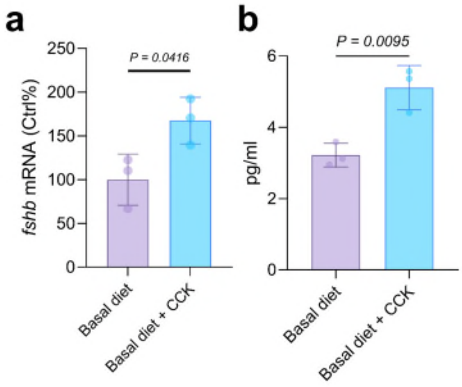

Supplementary Fig. S9 | Confirmation of CCK feeding efficacy and resulting impact on pituitary *fshb* expression. The figure confirms the effective absorption of dietary CCK and its subsequent biological effect, measured 1.5 hours post-feeding with a CCK-supplemented diet versus a normal diet. **a** Relative mRNA expression level of *fshb* in the pituitary gland (determined by qPCR). Data are presented as mean ± s.e.m. for *n*= 3 independent replicates. Each CCK-fed replicate is a pool of four pituitaries; each normal diet replicate is one pituitary. **b** Plasma CCK concentration (determined by ELISA). Data are presented as mean ± s.e.m. for *n*= 3 independent replicates. Each replicate is a pool of blood from three individual fish.

Responses to the Comments from the Reviewers

Cholecystokinin coordinates gonadotropin-dependent and independent pathways to orchestrate zebrafish gonadal development

Reviewer #2 (Remarks to the Author):

The authors have satisfactorily addressed all of my previous concerns. The revised manuscript shows clear improvements in logic, rigor, and clarity of presentation. Congratulations to the authors on this solid work, and I recommend publication of this manuscript in Nature Communications.

Response to Reviewer #2 :

We sincerely thank Reviewer #2 for their highly positive feedback, their recognition of the extensive improvements made during the revision process, and their strong recommendation for publication. We deeply appreciate the time and expertise they have invested in helping us improve this manuscript.

Reviewer #3 (Remarks to the Author):

Through the course of the discussion with the reviewers, it has become clear that the current dataset does not provide decisive evidence supporting a strong direct action on LH. In particular, no critical data have been presented in the revised manuscript that would overturn the previous Ca2+ imaging findings, in which no clear response was observed in LH cells.

The rationale underlying the authors' initial conclusion that CCK functions as an LH-releasing hormone, based on culture and knockout experiments, is addressed in detail in the specific comments. However, it is also evident that, throughout the revision process, the authors' interpretation has shifted from asserting a "direct action on LH" toward acknowledging the existence of indirect effects. This shift itself represents the difficulty of positioning CCK as a strong direct regulatory axis for LH.

Crucially, it must be emphasized that the GnRH peptide—the established LH-releasing hormone (LH-RH)—demonstrates a robust induction of both Ca2+ transients and lhb expression in LH cells in zebrafish and other teleosts. While CCK fulfills these criteria for the FSH system, the evidence presented for the LH system remains fundamentally different and insufficient to warrant the designation of "LH-RH."

In light of these considerations, I strongly recommend that the interpretation regarding LH, as well as the overall description throughout the manuscript, be revised in a direction strictly supported by the data or previous studies.

Please see the specific comments below for detailed explanations and suggestions.

>1. Response to "Title and Concept Shift"

>Previous Comment:

>The reviewer noted that the title "integrates" is no longer applicable given the shift to an independent regulation model.

> Authors' Response:

>We fully agree. To accurately reflect this distinction while highlighting that these pathways work in concert to achieve gonadal development, we have modified the title to:

>"Cholecystokinin coordinates gonadotropin-dependent and -independent pathways to orchestrate zebrafish gonadal development"

>We believe "coordinates" better captures the physiological reality: while the pathways are mechanistically distinct and spatially separated, CCK signaling acts as a synchronizing factor to ensure both germ cell establishment and hormonal support.

Reviewer's Comment:

The authors state that they "fully agree" with the concern regarding the independent nature of the pathways; however, no substantive conceptual revision is evident. The revised title replaces "integrate" with "coordinate" and "orchestrate," yet all three terms presuppose a single unified regulatory system—the very premise that has been questioned.

More fundamentally, these terms imply the existence of a central regulatory entity or master-switch mechanism. In contrast, the present study identifies two physiologically distinct systems that utilize

the same signaling molecule:

• Hypothalamic CCK acting on the pituitary (gonadotropin-dependent)

• Gonadal CCK acting locally within the gonad (gonadotropin-independent)

Using the term "coordinate" in this context is conceptually problematic. By analogy, acetylcholine is utilized by both parasympathetic neurons regulating cardiac function and motor neurons controlling skeletal muscle contraction. However, no physiologist would describe these distinct systems as being "orchestrated" by acetylcholine merely because they share a common neurotransmitter. They represent independent physiological processes initiated by different neuronal populations under different functional contexts.

Similarly, gonadal growth during early development mediated by local CCK signaling and hypothalamic CCK stimulation of the pituitary during reproductive activation represent distinct physiological events. The mere use of the same signaling molecule does not justify framing them as components of a single coordinated regulatory system.

Unless you can provide evidence of a shared upstream trigger that simultaneously regulates both the hypothalamic and gonadal CCK pools to synchronize these pathways, the terms "coordinate" and "orchestrate" are inappropriate.

The title must be revised to accurately reflect these parallel, independent pathways, such as: "Hypothalamic and peripheral cholecystokinin stimulate zebrafish gonadal development through gonadotropin-dependent and -independent pathways."

>2. Response to "Mechanistic Proof for LH Regulation"

>Response to LH Regulation & Hollander-Cohen Discrepancy:

>Previous Comment:

>The reviewer questioned the physiological relevance of CCK on LH, citing low cckbrb co-localization and the lack of robust Ca2+ response in Hollander-Cohen et al. (2024). They also suggested that our in vitro results might be culture artifacts (altered expression patterns)

> Authors' Response:

>Regarding Hollander-Cohen et al. (2024): We respectfully note that Hollander-Cohen et al. actually reported that CCK does elicit a calcium response in LH cells, albeit "weaker" and "varied widely" (20%-100% of cells responding). This perfectly aligns with the well-established existence of a bi-hormonal subpopulation (cells co-expressing FSH and LH) in teleosts. We propose that the responses observed in our study are driven by CCK acting on these cckbrb-positive bi-hormonal cells.

>Regarding Culture Artifacts: We acknowledge the reviewer's concern that primary culture conditions can influence gene expression patterns. However, we respectfully submit that our conclusion is not based solely on in vitro data. The most critical evidence comes from our in vivo genetic models: cckbrb-/- mutants exhibit a marked reduction in pituitary lhb mRNA expression in

the intact animal. This in vivo phenotype definitively proves that CCK signaling is physiologically required for maintaining basal LH synthesis. If the CCK-LH regulation were merely a culture artifact, the loss of the CCK receptor in vivo would not result in such a profound suppression of lhb levels. Our in vitro data merely serves to dissect the mechanism (direct vs. indirect) of this established in vivo phenomenon.

Reviewer's Comment:

The concerns regarding the interpretation of the in vivo data were already raised in the initial review, but are reiterated here for clarity. It is indeed true that lhb expression was reduced in vivo. However, it is important to emphasize that these experiments were conducted in intact animals. In teleosts, gonadal estrogen is widely accepted to stimulate the GnRH–LH axis (Huggard-Nelson et al., 2002; Li et al., 2018). Therefore, it is entirely plausible that disruption of the CCK system reduces FSH levels, leading to impaired gonadal function, which in turn secondarily suppresses LH expression.

Moreover, as the authors themselves show, CCK knockout produces more severe defects in gonadal development than FSH knockout. Such profound gonadal impairment would itself be expected to reduce LH levels. In Fig. 1C, the ccka^{-}/$^{-}$;cckb^{-}/$^{-}$ animals are all male and exhibit markedly deficient testicular development and spermatogenesis. These severe gonadal abnormalities are sufficient to account for reduced LH expression. Therefore, the results obtained in ccka^{-}/$^{-}$;cckb^{-}/$^{-}$ animals cannot be interpreted as evidence that CCK directly regulates LH.

Consistent with this interpretation, in medaka, CCK receptor knockout reduces LH expression in intact animals, but this reduction is not observed in ovariectomized individuals (Uehara et al., 2024; Fig. 1i vs. Fig. 1l). This clearly indicates the presence of a gonad-dependent indirect mechanism. In the zebrafish in vivo data cited in the present manuscript, the possibility of such gonad-dependent indirect effects has not been excluded. By observing the medaka findings, an indirect interpretation appears more likely.

On the Interpretation of the Bi-hormonal Cell Data

The authors argue that the response is limited to a small subpopulation of bi-hormonal cells and suggest that either extreme upregulation within a small number of cells or culture-related effects may account for the "profound" change in whole-pituitary lhb expression. However, drawing the conclusion that CCK strongly regulates LH under such unstable and artificial conditions represents a logical leap (related to the next question).

In the "culture conditions" where LH expression increases, the proportion of bi-hormonal cells (which express cckbr) is also elevated. Stimulation of LH expression in these bi-hormonal cells in vitro does not necessarily imply that such regulation occurs in vivo, where these cells constitute a very minor population. Furthermore, because the authors interpret bi-hormonal cells as progenitor-

like cells, it is inappropriate to designate CCK as an "LH-RH" based on effects observed in this limited and potentially transitional cell population.

Inconsistency with Previous Zebrafish Studies

A more critical issue is the insufficient discussion of consistency with existing literature. In zebrafish, Hollander-Cohen et al. reported using Ca2+ imaging that CCK robustly increases Ca2+ signals in FSH cells, whereas no significant response was observed in LH cells. This finding indicates that, at least in zebrafish, the primary direct target of CCK is the FSH system.

To argue that CCK functions as both FSH-RH and LH-RH on the basis of an undetectable effect on a minor FSH/LH co-expressing progenitor subpopulation is not justified. If the effect on LH cells cannot be detected by Ca2+ imaging and is confined to a minor progenitor-like population, it cannot reasonably be equated with the clearly demonstrated FSH-releasing activity.

Although the present manuscript concludes that CCK directly regulates LH, it does not clearly explain how these findings reconcile with previous zebrafish studies. Without resolving inconsistencies across studies—including those conducted in the same species—it is premature to conclude a direct action on LH. Furthermore, increasing in culture condition does not mean "releasing activity".

Required Discussion Points

At a minimum, the authors should explicitly discuss:

- *The zebrafish Ca2+ imaging data*
- *The medaka ovariectomy experiments*

and carefully consider the possibility that the in vitro system does not fully reflect physiological conditions, as well as the likelihood that the observed effects on LH represent indirect mechanisms in a broad sense.

While the authors demonstrate the presence of CCK receptors in LH-expressing cells, these receptors are restricted to a minor bi-hormonal population, and this limitation must be interpreted cautiously.

At present, the only consistently and robustly demonstrated effect is the direct action on the FSH system. In contrast, for the LH system, the available evidence is insufficient to conclusively support a direct regulatory role, and this distinction should be clearly articulated in the manuscript.

>Response to Mechanistic Proof for LH Regulation (Explaining the 3-fold Increase in vitro):

>Previous Comment:

>The reviewer rightly questioned how a "very small subset" of cckbrb-expressing LH cells could sustain a "nearly threefold increase" in total LH expression in primary pituitary culture.

>Authors' Response:

>We appreciate the reviewer's keen observation regarding the quantitative discrepancy between the sparse receptor distribution and the robust transcriptional response. We propose that the observed 3-fold increase in our in vitro system is driven by two synergistic factors, one of which aligns with the reviewer's own insight:

>High Transcriptional Amplitude in Target Cells: While the cckbrb-positive subpopulation (bi-hormonal cells) is numerically small, the activation of the Gq-calcium signaling pathway in these cells is likely highly potent. A massive transcriptional induction (e.g., >20-fold) within a small specific subset of cells can mathematically result in a significant (3-fold) increase in the total mRNA pool of the entire culture.

>Culture-Induced Cellular Plasticity (As noted by the reviewer): Importantly, we agree with the reviewer's suggestion that "culturing pituitary cells alters FSH and LH expression patterns, often leading to increased co-expression." It is highly probable that in our primary culture system, the population of bi-hormonal (FSH+LH / CCK-responsive) cells expands or becomes more sensitive compared to the in vivo state. This culture-specific enrichment of the responsive population provides a logical explanation for why the direct CCK-induced lhb upregulation is more pronounced in vitro (3-fold) than might be predicted solely from in situ staining of fixed tissue.

>Conclusion: Therefore, the in vitro data accurately reflects the intrinsic capability of CCK to drive lhb expression directly in responsive gonadotropes. However, we fully agree that in vivo, this direct pathway operates in concert with the powerful indirect GnRH3 pathway to achieve full physiological regulation.

Reviewer's Comment:

Thank you for your reconsideration and detailed responses. The authors propose two hypotheses to explain the pronounced induction of lhb observed in vitro:

(1) high-amplitude transcriptional induction in a small subset of cells, and

(2) increased cellular plasticity under culture conditions.

These are important considerations and should be explicitly incorporated into the main text. In particular, the possibility that culture-specific changes in cellular composition influenced the results must be clearly distinguished from interpretations of in vivo physiology.

Furthermore, the authors now state that, in vivo, CCK acts in coordination with a "powerful indirect GnRH3 pathway." However, given this clarification, the current description of CCK as "acting as a luteinizing-hormone-releasing hormone (LH-RH)" creates an internal inconsistency with the authors' own revised interpretation.

At present, the data suggests at most the possibility or capacity for a direct action on LH. This is qualitatively different from the well-established and robust direct regulatory axis demonstrated for FSH. It would therefore be inappropriate to position LH regulation at the same conceptual level as FSH control. I strongly recommend revising the use of the term "LH-RH" and clearly distinguishing between limited direct effects and indirect pathways.

In Hollander-Cohen, GnRH action on LH cells is clearly shown in Ca2+ imaging; this is "LH-RH".

On the Proposed CCK → GnRH3 Pathway

The assertion of a "powerful indirect GnRH3 pathway" is based primarily on the culture experiment shown in Fig. S14. However, GnRH3 blockade explains only a small fraction (approximately ~20%) of the CCK-induced increase in LH expression. This is fundamentally different from the data in Fig. R6, where exogenous GnRH3-induced responses are almost completely abolished by GnRH3 antibody blockade.

These results indicate that the CCK → GnRH3 pathway accounts for only a minor component of the observed LH induction. Indeed, the majority of the LH increase persists even when GnRH3 signaling is blocked. When considered together with the authors' own acknowledgment that long-term culture markedly increases the proportion of bi-hormonal cells, the most parsimonious interpretation is that the observed LH upregulation largely reflects culture-induced changes in cellular composition rather than a dominant physiological CCK → GnRH3 axis.

Moreover, this proposed pathway is supported by a single experimental approach and lacks independent validation, such as receptor co-localization by in situ hybridization. The evidentiary support is therefore limited. At this stage, positioning the CCK → GnRH3 pathway as a major regulatory axis leaves substantial room for alternative interpretations.

Conclusion and Recommendation

Taken together, the current description of CCK as "acting as a luteinizing-hormone-releasing hormone (LH-RH)" exceeds what is supported by the presented data and lacks appropriate caution. The data demonstrate, at most, the possibility of a direct influence on LH. This does not yet warrant placing LH regulation on the same level as the more robustly supported FSH regulatory axis.

The conclusions that are clearly supported by the present study are:

- *Hypothalamic CCK is involved in FSH regulation.*
- *CCK may influence germ cell proliferation at the gonadal level.*

These findings are substantial and valuable in their own right.

However, the proposed LH regulatory mechanism would represent a revision of the existing framework in the field, and the current evidence is insufficient to justify such a strong claim. Drawing definitive conclusions without adequate empirical support is premature.

Accordingly, I strongly recommend a substantial tone-down of the conclusions regarding LH regulation, along with explicit acknowledgment of culture-specific effects and unverified pathways as limitations of the study.

Revisions Required to Ensure Consistency with the Consensus Reached During the Review Process
Several sections of the manuscript deviate from the interpretation that has been refined and clarified through the review process. These should be revised accordingly.

Line 8
"orchestrating" → "stimulating"
The term "orchestrating" implies a central and integrative regulatory role that exceeds the scope of the current evidence. A more neutral term such as "stimulating" is appropriate.

Line 9
"(1) Acting as a luteinizing-hormone-releasing hormone (LH-RH), CCK directly activates pituitary lhb transcription via CCKBRb and indirectly enhances hypothalamic gnrh3 expression to regulate LH synthesis and secretion."
As discussed throughout the review process, the evidence supporting CCK action on LH is not comparable in strength to that for the FSH system (or GnRH action on LH). Based on the current data, it is not appropriate to position CCK as a luteinizing-hormone-releasing hormone (LH-RH). The present wording conveys a stronger conclusion than is warranted by the available evidence.
The increase in lhb expression observed under culture conditions may reflect culture-specific changes in cellular composition, including expansion of bi-hormonal cells. Therefore, extrapolating these findings to in vivo physiology requires caution.
Furthermore, it is well established that changes in gonadal function can secondarily influence LH expression. The in vivo data presented here do not allow a clear distinction between direct pituitary effects and indirect gonad-mediated mechanisms.
Accordingly, this statement should either be deleted or revised in a substantially more conservative manner. For example:
"CCK may influence LH synthesis through indirect mechanisms."
This wording remains within the limits of the current data and avoids overstating a direct regulatory role.

Line 19
The terms "central integrator" and "synchronizing" imply unified central control by a single CCK system. However, the present study suggests that hypothalamic and peripheral CCK may regulate

reproductive function through distinct pathways. Thus, describing CCK as a "central integrator" is inconsistent with both the current data and the interpretive consensus reached during review.
I recommend revising to:
"Hypothalamic and peripheral CCK cells regulate gonadal function via gonadotropin-dependent and -independent pathways, respectively."

Line 23
Keyword: "LH-releasing hormone" should be deleted.

Lines 50–53
The statement that CCK action on LH "has not been previously explored" is inconsistent with existing literature. Indirect effects of CCK on LH via the gonad have already been discussed in Uehara et al., 2024. Moreover, Hollander-Cohen et al., 2024 reported LH-related analyses in cckr knockout zebrafish, including Ca2+ imaging in LH cells. Therefore, it is inappropriate to suggest that LH regulation has not been examined.
However, understanding of endogenous CCK ligands remains limited. Ligand-level experimental validation has thus far been performed only in medaka, whereas zebrafish studies have primarily focused on receptor-level analyses. It remains unclear whether CCK itself or other peptides such as gastrin serve as the physiologically relevant endogenous ligand(s) in zebrafish.
The current findings are significant in demonstrating that CCK functions as a ligand in zebrafish. Therefore, I recommend reframing the question as follows:
"However, while ligand-level analyses have been performed in medaka, studies in zebrafish have primarily focused on receptor activation and knockout models, and the identity of the physiologically relevant endogenous ligand(s) within the CCK/gastrin family remains unclear. Therefore, a broader consensus regarding CCK regulation of gonadotropins across teleost species has yet to be established."

Line 79
"Finally, CCK acts as a LH-RH to induce oocyte maturation and ovulation."
This statement again designates CCK as LH-RH. Given the high likelihood of indirect mechanisms, this exceeds the scope of the evidence and should be deleted.

Lines 430–447
This paragraph contains interpretations that deviate from the consensus established during review. In particular, describing CCK as a "dual gonadotropin-releasing factor" or "master coordinator" represents a conceptual model that is not supported by the current data.

I strongly recommend deletion of this entire paragraph.
Instead, the discussion should acknowledge that the apparent stimulation of LH may reflect secondary effects mediated through FSH and gonadal function, and should be interpreted cautiously within that framework.

Lines 455–458
"Finally, during the final maturation and ovulation phase, CCK enhances LH release via dual hypothalamic (gnrh3 upregulation) and pituitary (lhb induction) mechanisms to facilitate ovulation."
This statement is not consistent with the consensus reached during review. Neither prior studies nor the present study demonstrate that CCK increases LH specifically during the ovulation phase. This constitutes speculation beyond the presented data and should be deleted.

Lines 458–460
"Thus, CCK emerges as a central integrator of metabolic and reproductive signals, synchronizing gonadotropin-independent and -dependent pathways across gonadal ontogeny."
The terms "central integrator" and "synchronizing" overstate the conclusions relative to the evidence. A more neutral and accurate description would be:
"Thus, hypothalamic and peripheral CCK can act through gonadotropin-dependent and -independent pathways, respectively, in teleost reproduction."

Author's response

>3. Response to Antibody Specificity & Validation (Crucial Revisions)

>Control for Functional Assays (Response to "Serum Control"): The reviewer correctly pointed out that normal serum is the appropriate control for whole serum antibodies. However, we clarify that all custom antibodies used in functional blocking experiments were affinity-purified, not whole serum. Therefore, the vehicle (PBS) is the appropriate control, as non-specific serum proteins are absent. This eliminates the confounding effects of serum components.

Reviewer's Comment:
In the GnRH antibody experiment, only the GnRH-Ab group involved immunoglobulin purified from serum, which inevitably contains contaminating serum-derived components and, at minimum, non-specific IgG fractions. This introduces a potential confounding factor that is not present in the other experimental groups.
Ideally, the experiment should have included a control antibody preparation purified from serum in the same manner (e.g., non-immune IgG or pre-immune serum processed identically), in order to

control for possible effects of co-purified serum proteins or IgG itself. Without such a control, it cannot be excluded that part of the observed effect reflects non-specific influences of serum-derived components rather than specific GnRH neutralization.

Therefore, this methodological limitation should be explicitly described in the manuscript, and the interpretation of the GnRH blockade results should be presented with appropriate caution.

Response to Reviewer #3:

We wish to express our deepest gratitude to Reviewer #3 for their rigorous, insightful, and highly constructive evaluation of our revised manuscript. We deeply appreciate the significant time and physiological expertise the reviewer has invested, which has undeniably refined our conceptual framework and improved the scientific accuracy of our paper. e fully agree with the reviewer’s central premise: our data robustly supports the direct regulation of FSH by CCK, whereas the evidence for LH regulation points toward a much more complex mechanism. We acknowledge and accept the reviewer's insightful critique that the in vivo suppression of LH could be heavily compounded by indirect gonadal feedback (due to impaired gonadal development), and that our robust in vitro results may be amplified by culture-induced cellular plasticity (expansion of the bi-hormonal cell subpopulation).In this final revision, we have fully embraced the "independent pathways" model advocated by the reviewer. We recognize that positioning CCK's action on LH at the same canonical level as its control over FSH is not justified by the current evidence. Consequently, we have completely removed the definitive designation of CCK as an "LH-RH" throughout the entire manuscript. Instead, we have adopted a more precise and cautious terminology, describing CCK as possessing "LH-releasing capacity" and contributing to the LH regulatory network through complex, potentially indirect mechanisms. Furthermore, per the reviewer's specific instructions, we have incorporated all "Required Discussion Points" into the main text. We now explicitly discuss the potential for indirect gonadal feedback in vivo, acknowledge the expansion of bi-hormonal cells as a culture-induced caveat in vitro, and have reconciled our findings with previous Ca^{2+} imaging literature (Hollander-Cohen et al.).By making these substantial textual modifications-while retaining the objective observation that CCK does possess the intrinsic capacity to stimulate *lhb* in isolated environments-we believe the manuscript now presents a highly rigorous, balanced, and scientifically accurate conclusion that aligns perfectly with the consensus reached during this review process. Below, we provide a detailed, point-by-point response outlining the specific changes made to the manuscript..

1. Response to "Title and Concept Shift"

Comment:

The reviewer noted that the terms "coordinate" and "orchestrate" in the title imply a single unified regulatory system or master-switch mechanism, and suggested revising the title to explicitly reflect parallel, independent pathways.

Response:

We sincerely appreciate the reviewer’s rigorous conceptual analysis. We fully agree with the reviewer's physiological assessment that the hypothalamic CCK-pituitary axis and the gonadal CCK paracrine system operate via mechanistically distinct and spatially separated pathways.

Following the guidance of the Monitoring Editor, we have retained the term "coordinates" in the title, as we use it in a broader functional sense-highlighting that these two distinct systems temporally align to ensure successful gonadal development. However, to fully respect the reviewer's highly accurate point regarding physiological independence,

we have carefully reviewed the main text to ensure that we do not mistakenly describe CCK as a single "master-switch." We have explicitly emphasized the parallel and distinct nature of these pathways in the Discussion section, ensuring our conceptual framework aligns with the reviewer's insights.

2. Response to " Mechanistic Proof for LH Regulation" and Consistency with Literature

Comment:

The reviewer raised critical concerns regarding the physiological relevance of CCK's direct action on LH. Specifically, the reviewer pointed out that: (1) The in vivo reduction of LH in mutants might be a secondary effect of gonadal impairment (loss of positive feedback). (2) The robust in vitro response might be amplified by culture-induced cellular plasticity (expansion of bi-hormonal cells). (3) Previous Ca2+ imaging studies (Hollander-Cohen et al.) showed weak/no responses in LH cells compared to FSH cells.

Response:

We deeply appreciate the reviewer’s profound physiological insight regarding the complexity of LH regulation and its consistency with existing literature. We fully concur that indirect mechanisms heavily influence the *in vivo* outcomes; specifically, the severe gonadal impairment in *cckbrb*$^{-/-}$ mutants undoubtedly disrupts the gonadal-estrogen positive feedback loop, which likely contributes to the secondary suppression of *lhb* expression. We also acknowledge the reviewer's valid point that long-term culture may increase cellular plasticity and expand the bi-hormonal subpopulation, making the response more pronounced *in vitro* than *in vivo*. However, we respectfully submit that precisely because the *in vivo* environment is confounded by this gonadal feedback, our primary pituitary culture system remains essential to uncouple these variables. The robust upregulation of *lhb* in isolated pituitary cells provides valid mechanistic proof that CCK possesses the intrinsic capacity to act directly on responsive target cells, even if its physiological dominance *in vivo* is shared with indirect pathways. Furthermore, this mechanism perfectly aligns with the Ca^{2+} imaging data from Hollander-Cohen et al., who reported that while CCK universally activates FSH cells, its responses in LH cells were "weaker" and "varied widely." This variability strongly supports our model that CCK does not universally activate all LH gonadotropes, but specifically targets the numerically smaller bi-hormonal subpopulation. To ensure our manuscript reflects this nuanced consensus and strictly adheres to your "Required Discussion Points", we have incorporated a dedicated discussion in the revised text. We now explicitly discuss the Hollander-Cohen Ca^{2+} imaging data, acknowledge that the profound in vivo suppression of LH could be partially attributed to indirect gonad-mediated mechanisms (loss of feedback), and note the limitation that *in vitro* culture conditions may amplify cellular plasticity. By removing the absolute "LH-RH" designation but retaining the evidence of CCK's "intrinsic LH-releasing capacity," we believe the revised manuscript presents a highly rigorous and balanced conclusion supported by both our *in vitro* data and the existing literature.

3. Revisions Required to Ensure Consistency (Line-by-Line Modifications)

Comment:

The reviewer provided specific line-by-line recommendations to tone down the conclusions regarding LH regulation, remove terms like "master coordinator" or "LH-RH," and ensure the manuscript reflects the consensus reached during the review process.

Response:

We deeply appreciate the reviewer’s meticulous guidance on the manuscript's text. We have fully accepted these recommendations to ensure our claims do not exceed the presented evidence. The following specific revisions have been made:

Line 8: Changed "orchestrating" to "stimulating" as requested. (**Line 22**)

Line 9: We have removed the definitive "LH-RH" claim. The sentence has been revised to: "CCK directly activates pituitary *lhb* transcription via CCKBRb in a specific bi-hormonal subpopulation, and indirectly enhances hypothalamic *gnrh3* expression to regulate LH synthesis and secretion." (**Line 23-26**)

Line 19: We replaced the "central integrator" concept with your suggested text: " Collectively, hypothalamic and peripheral CCK cells regulate gonadal function through gonadotropin-dependent and gonadotropin-independent pathways, respectively, thereby coordinating gonadal development from germline establishment to maturation." (**Line 35-38**)

Line 23: Deleted the keyword "LH-releasing hormone", and added the keyword “gonadotropin-independent pathway” (**Line 40-41**)

Lines 50–53: We have adopted your exact suggested phrasing to accurately reflect the literature: "However, while ligand-level analyses have been performed in medaka[8], studies in zebrafish have primarily focused on receptor activation and knockout models[9]. The identity of the physiologically relevant endogenous ligand(s) within the CCK/gastrin family remains unclear, and whether CCK itself or other peptides such as gastrin serve as the physiologically relevant endogenous ligand(s) in zebrafish has not been fully determined. Therefore, a broader consensus regarding CCK regulation of gonadotropins across teleost species has yet to be established." (**Line 67-74**)

Line 79: We deleted the statement designating CCK as an LH-RH during ovulation. (**Line 100**)

Lines 430-447: As requested, we have deleted the entire paragraph describing CCK as a "dual gonadotropin-releasing factor" and "master coordinator". We have replaced it with a cautious discussion acknowledging that the in vivo stimulation of LH may reflect secondary effects mediated through gonadal function, and in vitro results may be influenced by cellular plasticity.

“The apparent discrepancy with earlier studies emphasizing a specific FSH-RH role may reflect differences in experimental focus and interpretation. Our data show that CCKBRb is enriched in a subpopulation of pituitary gonadotropes co-expressing *fshb* and *lhb* transcripts[43], suggesting that CCK may directly influence *lhb* expression in a subset of responsive cells. This interpretation aligns with previous calcium imaging studies in zebrafish, which reported that CCK elicits robust calcium responses in FSH cells, whereas responses in LH cells were described as ‘weaker’ and ‘varied widely’ (20%–100% of cells responding)[9]. Such variability supports our model that CCK does not universally activate all LH gonadotropes, but instead targets a specific subpopulation—likely the bi-hormonal (FSH/LH co-expressing) cells that also express *cckbrb*. In our primary pituitary culture system, the expansion of this bi-hormonal subpopulation under culture conditions may amplify the observed *lhb* response, offering a plausible explanation for the discrepancy between the robust *in vitro* induction and the more variable *in vivo* calcium signals. Meanwhile, the marked *in vivo* suppression of LH-related phenotypes may partially result from indirect effects due to impaired gonadal function and loss of gonadal feedback. Furthermore, while primary pituitary culture demonstrates that CCK has the intrinsic capacity to stimulate *lhb* expression, the magnitude of this response *in vitro* may be influenced by culture-induced cellular plasticity. Together, these findings support a cautious interpretation: CCK contributes to LH regulation, but the extent of its physiological role *in vivo* remains to be fully clarified.” (**Line 364-386**)

Lines 455-458 We deleted the statement claiming CCK enhances LH specifically during the ovulation phase. (**Line 394**)

Lines 458-460: We revised the final conclusion to your suggested neutral description: "Thus, hypothalamic and peripheral CCK can act through gonadotropin-dependent and -independent pathways, respectively, in teleost reproduction." (**Line 394-396**)

4. Response to Antibody Specificity & Validation (Methodological Limitation)

Comment:

The reviewer correctly pointed out that for the GnRH antibody experiment, relying solely on a vehicle (PBS) control is insufficient. Because the affinity-purified antibody originates from serum, a non-immune IgG control processed identically is necessary to definitively rule out non-specific effects of co-purified serum components. The reviewer requested this limitation be explicitly described in the manuscript.

Response:

Our Response: We completely agree with the reviewer's rigorous methodological critique. We recognize that without a parallel non-immune IgG control processed in the exact same manner, we cannot absolutely exclude the potential non-specific influences of co-purified immunoglobulins or serum-derived components.

To ensure our data is interpreted with the appropriate scientific caution requested, we have added an explicit acknowledgment of this methodological limitation to the manuscript.

Revisions in the Manuscript: We have added the following statement to the relevant section (Methods): " Hypothalamic-Pituitary (H-P) Co-culture and Antibody Blocking: *While the functional blockade of GnRH3 was performed using an affinity-purified antibody compared against a vehicle (PBS) control, we acknowledge that the absence of an identically processed non-immune IgG control limits our ability to completely exclude potential non-specific effects of co-purified serum components. Therefore, the interpretation of the GnRH blockade results should be approached with appropriate caution."*(**Line 514-519**)
